# Genomics of Sable (*Martes zibellina)* × Pine Marten (*Martes martes*) Hybridization

**DOI:** 10.1093/gbe/evag018

**Published:** 2026-03-05

**Authors:** Andrey A Tomarovsky, Azamat A Totikov, Tatiana M Bulyonkova, Polina L Perelman, Alexei V Abramov, Natalia A Serdyukova, Aliya R Yakupova, Dmitry Prokopov, Violetta R Beklemisheva, Mikkel-Holger S Sinding, Guzel Davletshina, Maria Pobedintseva, Ksenia Krasheninnikova, Daniel W Foerster, Anna S Mukhacheva, Alexandra Mironova, Michail Sidorov, Wenhui Nie, Jinhuan Wang, Svetlana A Romanenko, Anastasiya A Proskuryakova, Malcolm Ferguson-Smith, Fengtang Yang, Nikolay Cherkasov, Elena Balanovskaya, M Thomas P Gilbert, Innokentiy M Okhlopkov, Anna Zhuk, Alexander S Graphodatsky, Roger Powell, Klaus-Peter Koepfli, Sergei Kliver

**Affiliations:** Laboratory of Diversity and Evolution of Genomes, Institute of Molecular and Cellular Biology SB RAS, Novosibirsk 630090, Russia; Department of Natural Sciences, Novosibirsk State University, Novosibirsk 630090, Russia; Laboratory of Diversity and Evolution of Genomes, Institute of Molecular and Cellular Biology SB RAS, Novosibirsk 630090, Russia; Department of Natural Sciences, Novosibirsk State University, Novosibirsk 630090, Russia; Laboratory of System Dynamics, A. P. Ershov Institute of Informatics Systems, 630090 Novosibirsk, Pr. Lavrentieva, 6, Russia; Laboratory of Diversity and Evolution of Genomes, Institute of Molecular and Cellular Biology SB RAS, Novosibirsk 630090, Russia; Laboratory for Theriology, Zoological Institute RAS, St. Petersburg 199034, Russia; Laboratory of Diversity and Evolution of Genomes, Institute of Molecular and Cellular Biology SB RAS, Novosibirsk 630090, Russia; Division of Evolutionary Biology, Ludwig-Maximilians-Universität, Planegg 82152, Germany; Microevolution and Biodiversity, Max Planck Institute for Biological Intelligence, Seewiesen 82319, Germany; Centre for Haemato-Oncology, Barts Cancer Institute, Queen Mary University of London, London, UK; QMUL Centre for Epigenetics, Queen Mary University of London, London, UK; Laboratory of Diversity and Evolution of Genomes, Institute of Molecular and Cellular Biology SB RAS, Novosibirsk 630090, Russia; Center for Evolutionary Hologenomics, The Globe Institute, The University of Copenhagen, Copenhagen, Denmark; Department of Birds of Mammals, Greenland Institute of Natural Resources, Nuuk, Greenland; Laboratory of Diversity and Evolution of Genomes, Institute of Molecular and Cellular Biology SB RAS, Novosibirsk 630090, Russia; Laboratory of Diversity and Evolution of Genomes, Institute of Molecular and Cellular Biology SB RAS, Novosibirsk 630090, Russia; Independent Researcher, Wellcome Trust Genome Campus, Hinxton, Saffron Walden CB10 1RQ, UK; Leibniz Institute for Zoo and Wildlife Research (IZW), Berlin 10315, Germany; Sikhote-Alin Biosphere Zapovednik, Ternei 692150, Russia; Laboratoire de Physiologie Cellulaire and Végétale, Univ. Grenoble Alpes/CNRS/CEA/INRA/IRIG, Grenoble, France; Institute of Biological Problems of Cryolithozone SB RAS, Yakutsk 677000, Russia; State Key Laboratory of Genetic Resources and Evolution, Kunming Institute of Zoology, Chinese Academy of Sciences, Kunming 650223, China; State Key Laboratory of Genetic Resources and Evolution, Kunming Institute of Zoology, Chinese Academy of Sciences, Kunming 650223, China; Laboratory of Diversity and Evolution of Genomes, Institute of Molecular and Cellular Biology SB RAS, Novosibirsk 630090, Russia; Laboratory of Diversity and Evolution of Genomes, Institute of Molecular and Cellular Biology SB RAS, Novosibirsk 630090, Russia; Cambridge Resource Centre for Comparative Genomics, Department of Veterinary Medicine, University of Cambridge, Cambridge CB3 OES, UK; School of Life Sciences and Medicine, Shandong University of Technology, Zibo, China; Vavilov Institute of General Genetics, Moscow, Russia; Laboratory of Human Population Genetics, Research Centre for Medical Genetics, Moscow 115522, Russia; Center for Evolutionary Hologenomics, The Globe Institute, The University of Copenhagen, Copenhagen 1353, Denmark; University Museum, NTNU, Trondheim, Norway; Institute of Biological Problems of Cryolithozone SB RAS, Yakutsk 677000, Russia; Institute of Applied Computer Science, ITMO University, St. Petersburg 197101, Russia; Laboratory of Amyloid Biology, St. Petersburg State University, St. Petersburg 199034, Russia; Laboratory of Diversity and Evolution of Genomes, Institute of Molecular and Cellular Biology SB RAS, Novosibirsk 630090, Russia; North Carolina State University; Smithsonian-Mason School of Conservation, Front Royal, VA 22630, USA; Center for Evolutionary Hologenomics, The Globe Institute, The University of Copenhagen, Copenhagen 1353, Denmark

**Keywords:** interspecific hybridization, introgression, species divergence, genetic diversity, conservation genomics, Mustelidae

## Abstract

The sable (*Martes zibellina*) and pine marten (*Martes martes*) are two Palearctic mustelids with long-recognized hybrids (kidases), whose fertility was controversial for years. Early genetic studies confirmed the existence of hybrids beyond F1, but limited marker resolution prevented detailed characterization of hybrid ancestry. Both species were hunted for centuries, but anthropogenic pressures during the 20th-century caused severe bottlenecks in the sable. Hunting bans and large-scale reintroduction programs restored sable populations across much of its range, including the sympatric zone, potentially affecting hybridization. We resequenced 30 individuals from most of the sables’ range and the Eastern part of pine marten's. Among samples, we found a broad spectrum of hybrid types with mosaic recombinant chromosomes that confirm hybrid fertility and indicate crossover is not suppressed in kidases. This necessitates re-evaluation of previous research, as we detected notable discrepancies between short tandem repeat-based ancestry and whole-genome analysis. We revealed mitochondrial DNA introgression from sables into most pine martens, indicating displacement of native pine marten mitochondrial sequences. Pine marten heterozygosity is relatively low (∼0.5 to 0.6 hetSNPs/kbp), while sable's diversity (∼1.5 to 1.8 hetSNPs/kbp) is unexpectedly high given its demographic history, likely reflecting successful reintroduction programs. We dated species divergence at 1.52 [confidence interval (CI): 1.05 to 2.06] Mya, and identified candidate genes potentially associated with hybrid fertility issues. This study is the first to elucidate marten hybridization at the whole-genome level, opening new research directions for understanding hybridization among Holarctic martens, the genetic consequences of reintroduction programs, and comparative adaptomics.

Significance Interspecific hybridization challenges fundamental concepts of species boundaries and has critical implications for evolutionary biology and biodiversity conservation. Within the Mustelidae family, the sable (*Martes zibellina*) and the pine marten (*Martes martes*) form one of the best-documented natural hybrid systems among mammals, providing a unique opportunity to explore the genomic background of hybridization. Using whole-genome resequencing of both pure species and hybrids, we identified a broad spectrum of hybrid genotypes characterized by mosaic recombinant chromosomes, providing clear evidence of hybrid fertility. We detected displacement of native mitochondrial DNA and revealed inconsistencies between morphological, phenotypic, and genomic classifications, and striking differences in genetic diversity between the two species. These findings highlight how hybridization can reshape genomes and species boundaries, emphasizing its fundamental role in evolution and its importance for effective biodiversity conservation.

## Introduction

**Fig. 1.**
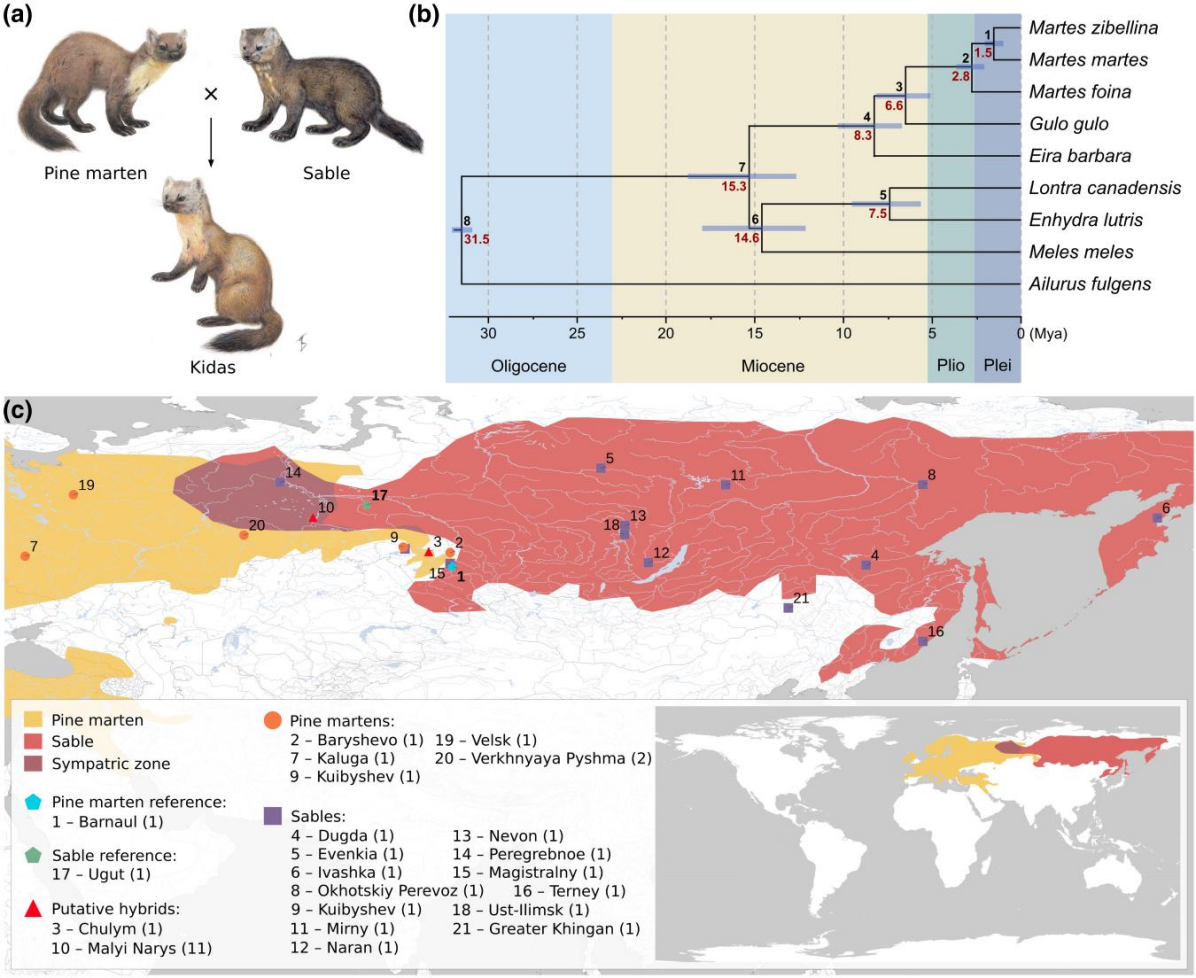
Sable (*M. zibellina*), pine marten (*M. martes*), kidas (*M. martes* × *M. zibellina*), and their ranges. (a) The sable (*M. zibellina*), the pine marten (*M. martes*), and the kidas (*M. martes* × *M. zibellina*). Illustrations by T. M. Bulyonkova; (b) time-calibrated phylogenetic tree based on the independent molecular clock model. Branch lengths are proportional to time, with divergence dates shown in millions of years. For each node, the node ID is displayed above the branch, and the estimated divergence time (Mya) is shown below. Node bars indicate the 95% confidence intervals (CIs) for node age uncertainty. Geological epochs: Plio—Pliocene, Plei—Pleistocene. The tree topology was confirmed by ML, Bayesian, and ML + ASTRAL methods. (c) Geographic ranges of the sable and the pine marten along with the locations of samples used in this study. The pine marten range is shown in yellow, the range of the sable in red, and the sympatric zone in maroon. Markers show sampling locations (the number of samples is indicated in parentheses in the legend). Location codes correspond to the column “Point on map” in Table S1. Small map in the bottom right corner shows full (uncut) areas from the IUCN Red List of Threatened Species (2024-2; Herrero et al. 2015; Monakhov 2015a).

Interspecific hybridization has played a significant role in the evolution of many mammalian lineages, including the Mustelidae family—a diverse group that encompasses ferrets, weasels, otters, badgers, and martens (Rozhnov et al. 2013; Colella et al. 2018, 2021; Kinoshita et al. 2019; Etherington et al. 2022). The sable (*Martes zibellina*) and pine marten (*Martes martes*) are two closely related but distinct species within the genus *Martes* whose ranges partially overlap across Eurasia. In the sympatric zone, hybrids (Fig. 1a) known as kidas (or kidus, Russian кидас/кидус) naturally occur, with mentions of them dating back to the 18th–19th centuries (Pavlinin 1963). According to data from the International Union for Conservation of Nature's Red List of Threatened Species, this zone forms a wide band stretching from northwest to southeast (∼1,900 km by diagonal) across several regions of Russia: from the Komi Republic in the northwestern Urals through Khanty-Mansi Autonomous Okrug—Yugra, Tyumen Oblast, Omsk Oblast, Tomsk Oblast, and Novosibirsk Oblast (Herrero et al. 2015; Monakhov 2015a).

Although kidases have long been recognized in the fur industry, their official taxonomic and hybrid status has remained controversial for years. Morphologically, they exhibit a broad range of phenotypes, combining traits of both parental species (see details in SM section S1.1). Yurgenson (1947) considered kidases to be interspecific hybrids, whereas Pavlinin (1963) challenged this view, suggesting that most individuals identified as kidases were actually highly variable sables or martens, with their morphological deviations arising within each species rather than as a result of hybridization (Yurgenson 1947; Pavlinin 1963). Grakov (1976) showed that in captivity, kidases were produced from male sables and female martens, whereas male martens and female sables yielded no offspring in the experiments (Grakov 1976). The backcrossing and intercrossing of kidases had limited success, causing some authors to claim that male F1 hybrids were infertile, thus limiting the introgression in the wild (Portnova 1941; Grakov 1974, 1981, 1993). However, these studies were based on a small number of crossing attempts, which involved few individuals (e.g. ∼10 in the Grakov 1976 study), of unknown or poorly described ancestry. It is unclear whether the origin or features of the particular individuals affected the results or not. Nonetheless, based on these reports, some researchers hypothesized that the hybrid breakdown between the sable and the pine marten acts as a reproductive barrier, limiting its further expansion within each species’ range (Kassal and Sidorov 2013).

Sable and pine marten have similar karyotypes (2*n* = 38), as do other closely related species from the subgenus *Martes* (Koepfli et al. 2008; Law et al. 2018; Hassanin et al. 2021): the Pacific marten (*M. caurina*), American marten (*M. americana*), Japanese marten (*M. melampus*), and the stone marten (*M. foina*). In particular, their chromosomes share similar morphologies, G-banding patterns, and low heterochromatin content (Hsu and Benirschke 1971; Graphodatsky et al. 2020; Beklemisheva et al. 2023). A recent comparative chromosome painting study confirmed a one-to-one synteny and absence of large rearrangements among chromosomes of the sable, pine marten, and stone marten, but highlighted differences in macrosatellites that form heterochromatin blocks (Beklemisheva et al. 2023). These findings suggest that no cytogenetic barriers prevent hybridization between these species.

Early genetic studies of sable-pine marten hybridization based on mitochondrial DNA (mtDNA) fragments revealed bidirectional introgression between *M. zibellina* and *M. martes* in sympatric populations of the Northern Urals (Rozhnov et al. 2010). Importantly, species-specific mtDNA haplotypes often do not correspond to phenotypic species assignments, even when external morphological traits appear unambiguous (Rozhnov et al. 2010). The distinctiveness of sable and pine marten mitotypes has been clearly established (Rozhnov et al. 2010; Kassal and Sidorov 2013), and the detection of pine marten individuals carrying sable mtDNA—despite earlier assumptions that such introgression was impossible based on results from captive breeding experiments—provided compelling evidence of natural hybridization (Rozhnov et al. 2010). These findings were supported by further reports of mtDNA introgression between the two species (Davison et al. 2001; Pishchulina 2013), suggesting the fertility of at least female hybrids (Pishchulina 2013). Later, the classification of hybrid individuals using microsatellite loci was conducted in several studies, confirming the persistence of hybridization within the zone of sympatry (Pishchulina 2013; Rozhnov et al. 2013; Modorov et al. 2020; Ranyuk et al. 2025). However, only a small number (up to 11) of microsatellites [short tandem repeats (STRs)] was used, and their distribution on chromosomes was unknown. To date, despite numerous studies based on morphological traits and molecular markers, the hybridization between sables and pine martens has not been studied at the whole-genome level. The key reason lies in the absence of high-quality genome assemblies not only for the genus *Martes*, but also for the Guloninae lineage within the family Mustelidae. Until 2021, only a highly fragmented assembly of the wolverine (*Gulo gulo*) was available (Ekblom et al. 2018). In recent years, assemblies of several marten species have appeared (O’Brien and Januszczak 2024), including those generated with long-read and Hi-C sequencing (Liu et al. 2020; Tomarovsky et al. 2025b).

Human activities have significantly influenced the interaction between sables and pine martens. Historically, both sable and pine marten furs were considered luxury goods and served as high-value natural currency for centuries. At the turn of the 20th century, the value of sable fur peaked due to a combination of declining harvest numbers and intense demand from the fashion industries in both Europe and North America. By 1925, prices soared to as much as $6,000 (12,000 rubles) per pelt—an equivalent of 8 kg/18 lbs of gold at the time (Shadiul and Vashukevich 2020). Overhunting for several centuries, beginning as early as the 17th century (Timofeev and Nadeev 1955), resulted in severe population declines, range reduction, and fragmentation, ultimately leading to a complete harvesting ban imposed in 1935 to 1940 (Mikhel 1938; Romanov 1941). Finally, a large-scale reintroduction program was implemented 1939 to 1941 and 1945 to 1970, with more than 18,000 individuals released throughout the original range of the sable (Timofeev and Nadeev 1955; Bobrov et al. 2008; Monakhov 2015b). However, harvesting for fur was not the only factor that affected the ranges and population dynamics of the two species. Most of the pine marten's range overlaps with areas of relatively high human population density, where agricultural development and degradation of core forested habitats may have exerted significant pressure on populations (Twining et al. 2020). Therefore, the diversity and population structures of both species were affected by anthropogenic activities, which must be taken into account while studying the hybridization between them.

In this work, we present the first detailed analysis of interspecific hybridization between sables and pine martens. We resequenced 30 individuals of nonhybrids, as well as putative hybrids from the zone of sympatry. We performed detailed analyses of ancestry using multiple approaches, assessed genetic diversity and its spatial distribution, and integrated our results with previous studies. We also revealed the genomic legacy of the sable reintroduction program and assessed its results. Finally, we dated the divergence between sable and pine marten and revealed previously unknown genomic features in the two species.

## Results

### Resequencing Data and Choice of Reference

We resequenced twelve sables, six pine martens, and twelve putative hybrids from the sable-pine marten sympatric zone (Fig. 1c, Table S1). Combined with data from previously sequenced sable (sample CHN; Liu et al. 2020) and two (sable and pine marten) reference individuals (Tomarovsky et al. 2025b), our final dataset reached 33 samples. The analysis of 23-mer distributions (Fig. S1) showed no significant differences in genome size among the pine martens, sables, and putative hybrids. We observed the smallest genome sizes for the samples T72 (2.39 Gbp), 10xmzib, and T18 (2.40 Gbp), and the largest for S50 (2.54 Gbp), respectively (Table S2). Sample T87 showed an additional k-mer peak (at a half coverage) on the distribution, indicating a high heterozygosity level.

To choose a proper reference assembly (the sable, the pine marten or sister to them the stone marten) for read mapping, we reconstructed (details in SM sections S2.3 to S2.4) a species chronogram (Fig. 1b) based on the published genome assemblies, which included red panda and five more mustelid species to cover known reliable fossil-based calibration points (Table S3). According to our results, the stone marten diverged from the sable-pine marten lineage at ∼2.8 (CI: 2.11 to 3.66) Mya (Fig. 1b), which is nearly twice older than the age of the sable-pine marten's most recent common ancestor (MRCA), 1.52 (CI: 1.05 to 2.06) Mya. Because of this relatively large genetic distance, using the stone marten assembly as a reference may lower the mapping rate, reduce alignment precision, and introduce biases in downstream analyses that are difficult to detect. For this reason, we performed all analyses twice, using both sable and pine marten assemblies as references. We obtained consistent results using these two references. Here, we report only results obtained using the sable assembly; the detailed comparison with the results for the pine marten assembly is provided in SM (section S2.5).

### Genetic Structure and Ancestry

To explore genetic structure and ancestry proportions, we conducted principal component analysis (PCA) and global (whole genome) and local (independent ADMIXTURE runs in 1 Mbp sliding windows with 100 kbp step) ancestry analyses using the resequencing data. We further analyzed ancestry using known STR loci and mitochondrial genomes, connecting our work with previous studies that applied these markers or characters.

#### PCA and Ancestry According to WGS Data

**Fig. 2.**
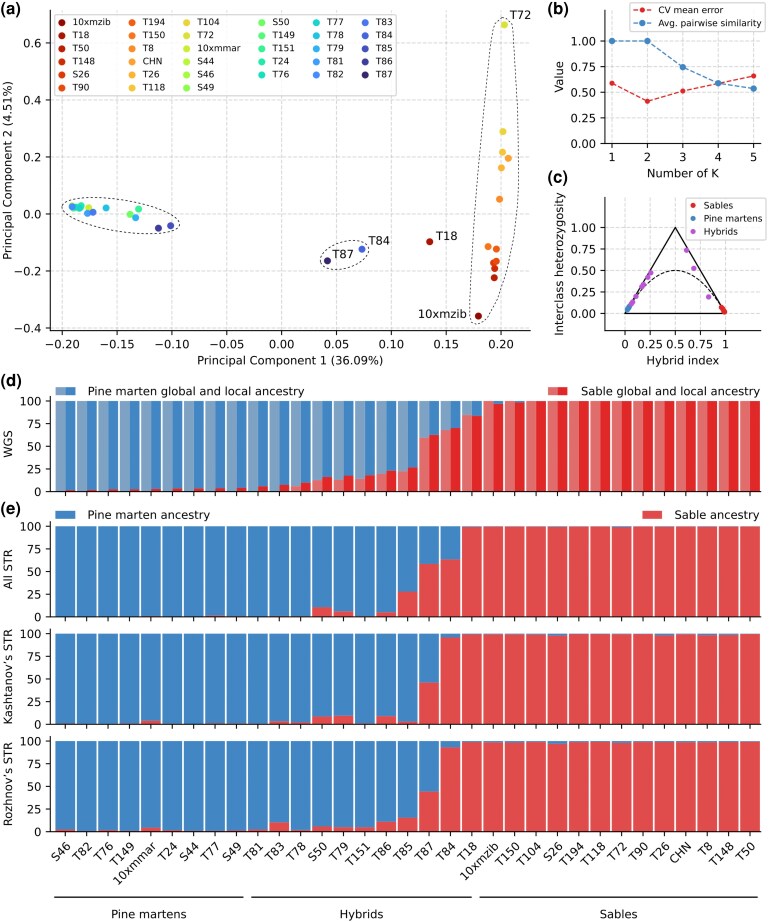
Principal component analysis and ancestry proportions of pine martens, sables, and putative hybrids. (a) PCA plot based on 1,719,592 SNPs obtained from autosomes and the pseudoautosomal region (PAR). The dashed ellipses indicate clusters of pure pine martens and hybrids with a small fraction of sable (left), pure sables (right), and hybrids close to F1 (middle). Sample T18 was located outside of the clusters. The percentage of variance explained by the corresponding component is indicated in parentheses; (b) Cross-validation error (red) and average pairwise similarity (blue) based on global ancestry. (c) Triangle plot with allele frequency difference threshold (δ) of 0.75. (d) Global (left half) and local ancestry (right half) based on the WGS data. Samples are labeled along the *X*-axis, while the *Y*-axis represents the mean probability of ancestry. (e) Ancestry analysis for three sets of STR loci. All STRs—combined set of all mapped STRs loci, Rozhnov's STRs—mapped markers from (Rozhnov et al. 2013), Kashtanov's STRs—markers from (Kashtanov et al. 2022).

PCA revealed two distinct clusters, distinguishing sable (right) and pine marten (left) samples, with samples T87 and T84 located between these clusters, elucidating them as potential F1-like hybrids (Fig. 2a, Fig. S2). We also note two samples with a specific location on the plot. The first, T18, is located between the two hybrid samples and the sable cluster, whereas the second, T72, is part of the sable cluster, but is an outlier located at the top right corner of the plot. We consider principal component 1 (PC1, 36.09% of the variance) to be associated with interspecies differences, whereas PC2 (4.51%) is associated with variation within the sable. Interestingly, the distribution of sables along PC2 partly follows a longitudinal pattern: the further east the sample was gathered, the higher it is located on the figure. This pattern is consistent for samples collected from Far East of Russia (T72, T104, T118, T26), as well as the CHN sample from China, (PC2 > 0), but the remaining samples (PC2 < 0), from Central Siberia and Western Yakutia (samples T150, T194, T90, S26, 10xmzib, T148, T50), are mixed. The relationship between the hybrid index and interclass heterozygosity (allele frequency difference threshold δ = 0.75; 297,989 ancestry-informative markers) showed a pattern highly consistent with the PCA analysis (Fig. 2c). Samples from the pine marten cluster are distributed along the left side of the triangle (blue and purple dots), whereas the sable samples (red dots) form a dense cluster in the right corner. As expected, the samples T84 and T87 (the purple dots on the right side) are located closer to the top corner, corresponding to a position of F1-like hybrids, and T18 is between them and the sables.

Global and local ancestry analyses (Fig. 2d) supported two clusters (K = 2) based on cross-validation error assessment (Fig. 2b). Global analyses revealed admixture in 9 samples (including those initially classified as pure species): T18, S50, T151, T78, T79, T84, T85, T86, and T87, with admixture levels ranging from 6% (T78) to 40.4% (T87), while remaining samples showed no signs of introgression. Local analysis detected admixture in two additional samples (T81 and T83) at levels of 5.92% and 7.48%, respectively (Table S4), with accuracy confirmed by notable hybrid and sable components in the heterozygosity analysis (Fig. S3). We found admixture signals between 1% and 5% in several other samples, including the reference genome individuals of both species: 10xmzib, 10xmmar, T150, S44, S46, S49, T149, T24, T76, T77, and T82, while remaining samples showed only small introgression traces (<1%). Using a 5% threshold to distinguish pure species ancestry from hybrid/introgressed individuals, we divided samples into three groups: pure sables (13 individuals: 10xmzib, S26, T8, T26, T50, T72, T90, T104, T118, T148, T150, T194, and CHN), pure pine martens (9 individuals: 10xmmar, S44, S46, S49, T149, T24, T76, T77, and T82), and hybrid individuals with ≥5% introgression (11 individuals). The third group was further subdivided into F1-like (T87, 35% to 50% introgression), backcross-like (S50, T79, T151, T86, T85, T84, T18, 15% to 35% introgression), and atypical sables or martens (T81, T83, T78, 5% to 15% introgression).

We verified PCA and ancestry analyses using HyDe, F3, and D statistics using the three-group classification of resequenced individuals based on the local ancestry results (File S1). Group HyDe test revealed a significant level of hybridization with γ = 0.17, (Z-score = 103.35, *P*-value << 0.01). Individual tests of the samples from the hybrid group resulted in a broad range of γ values: from 0.02 (T81) to 0.67 (T18). Samples T84 and T87 were the closest to the 50/50 ratio, with γ of 0.48 and 0.41 (Z-scores of 140.92 and 148.94). The F3 metric calculated for the hybrid group (−0.21, *Z*-score = −75.5) showed a pattern similar to the HyDe results. Individual F3 tests showed statistically significant admixture (|*Z*-score| > 3) in all hybrid samples, with four samples exhibiting particularly extreme *Z*-score: T87 (−63.2), T85 (−33.8), T84 (−42), and T86 (−28.2). Values from D statistics were concordant and confirmed gene flow (|*Z*-score| > 3) into hybrid individuals from both sable and pine marten.

#### STR Markers: Localization and Ancestry

Using an *in silico* polymerase chain reaction (PCR) approach, we identified the locations of previously described STR loci for various Mustelidae species (Davis and Strobeck 1998; Fleming et al. 1999; Domingo-Roura 2002; Vincent et al. 2003; Basto et al. 2010; Natali et al. 2010), and checked if it was possible to (1) genotype them in our samples and (2) if so, to evaluate their performance in detecting admixture. Out of a total of 79 markers, only 36 and 44 loci were suitable for STR-genotyping from our reads (150 bp) using the sable and pine marten references, respectively (File S2). In addition to the full set of STR markers, we also used two subsets called the Kashtanov's (Kashtanov et al. 2022) subset and the Rozhnov's (Rozhnov et al. 2013) subset (see SM section S3.2 for details). The full set of markers maps to all chromosomes except chr11, chr16, and chrX (Fig. S4a) with a mean density of 1.9 STRs/chr (STR markers per chromosome). Rozhnov's and Kashtanov's subsets have significantly lower coverage: the first maps to only 6 out of 19 chromosomes (0.4 STRs/chr, Fig. S4b), while the second maps to 11 out of 19 (0.7 STRs/chr, Fig. S4c), respectively.

Ancestry analysis showed a notable difference between sets for the classification of some hybrid martens (Fig. 2e). For example, sample T85 was shown to have 27.8% of sable according to the full set of STRs, but for the Kashtanov and Rozhnov subsets, the values are significantly lower—2.9% and 15.5%, respectively. Moreover, sample T84 showed a ∼30% difference between the full set (63.4% of the sample) and the two subsets (95.8% and 93.1%). For the rest of the hybrid martens, the difference is not as dramatic, but higher than that for other samples. Compared to whole-genome sequencing analysis, STR-based results for hybrid samples revealed a significant underestimation of the admixture.

#### Mitochondrial Genome Sequences

**Fig. 3.**
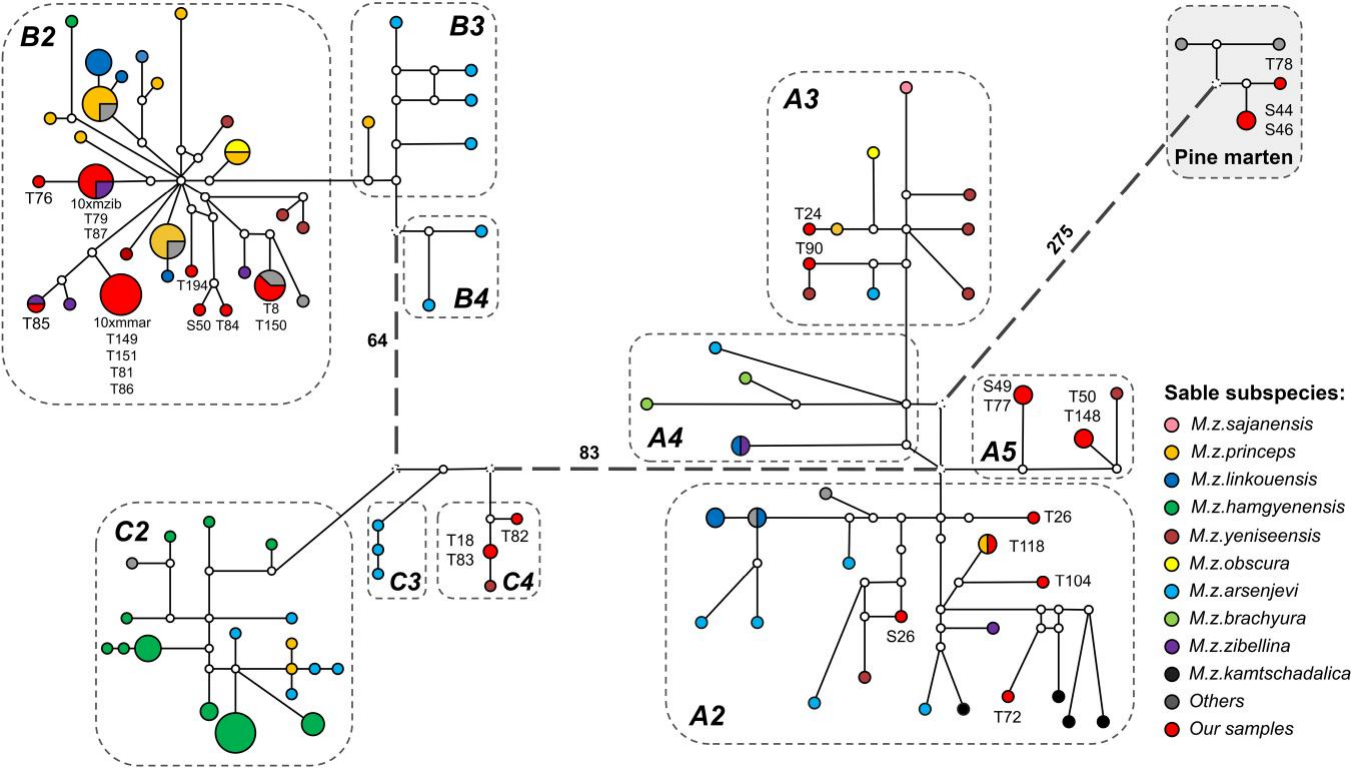
Median-joining network of mitogenomes haplotypes. The sizes of the circles are proportional to the haplotype frequency, and the distances between haplotypes are relative to the number of substitutions between haplotypes. Sable subspecies are denoted by different colors (see legend). Substitutions between the three main clades (A, B, and C) are shown as a number. *M. foina* (NC_020643.1) was used as an outgroup (not shown). See File S4 for a raw haplotype network with all distances labeled. Note that the haplotype network does not contain clades A1, B1, and C1. These clades, previously reported in (Li et al. 2021) were split as nonmonophyletic into A3, A4, A5, B3, B4, C3, and C4, respectively.

A previous mtDNA-based phylogeographic study of martens (Li et al. 2021) was focused on sables (∼100 individuals) and included only a few pine martens as an outgroup. We expanded this dataset by adding our 32 samples and mtDNA sequences assembled from published raw reads (Liu et al. 2020; Manakhov et al. 2021), resulting in 140 total samples (File S3). The addition of new data improved coverage of previously under-sampled lineages within the C1 and A1 clades (Fig. 3, Fig. S5). We reconstructed a median-joining haplotype network (Fig. 3) and maximum likelihood phylogenetic tree (Fig. S5) using this dataset. The obtained topologies of both the network and tree include three major clades containing sables (A, B, and C), along with an outgroup consisting of pine martens, which agrees with previously reported results (Li et al. 2021). However, some of the minor clades (A1, B1, and C1) are nonmonophyletic, and we propose to divide them into smaller subgroups. Clade A1 was split into three subgroups (A3, A4, and A5) while clades B1 and C1 were each split into two subgroups (Fig. 3, Fig. S5): B3, B4, C3, and C4, respectively. We found no clear correlation between sable haplogroups and designated subspecies (inferred from the sampling location). Among our samples, only T72 clustered with its formal subspecies *M. z. kamtschadalica*. Finally, we note that only three of our samples (two pure pine martens S44, S46, and atypical pine martenT78) had pine marten mtDNA. The remaining samples, even those identified as pine martens according to the WGS ancestry, have sable mtDNA representing different haplogroups.

### Heterozygosity

**Fig. 4.**
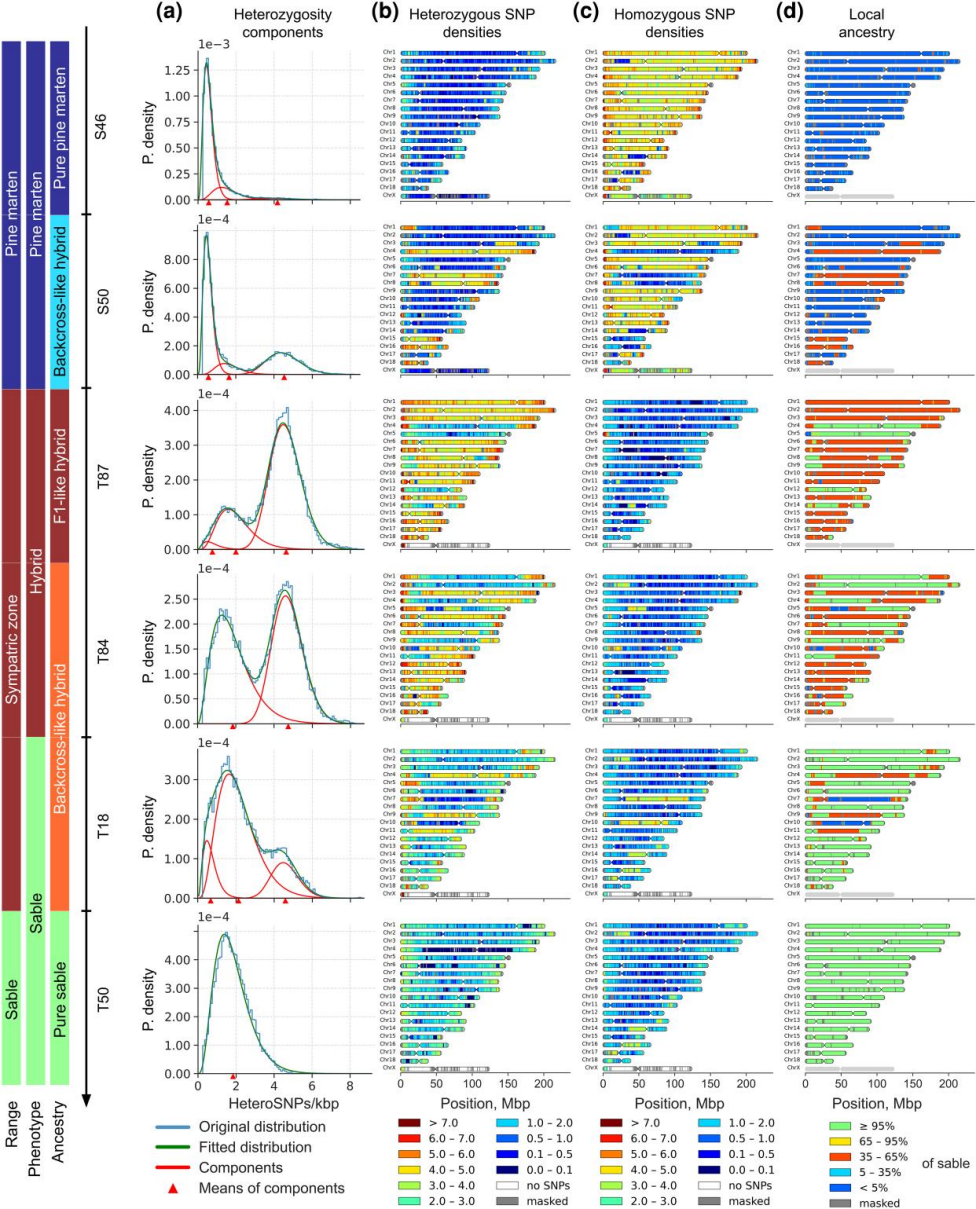
Transition from the pine marten to the sable across the sympatric zone (representative samples only). (a) Probability density of heterozygous SNPs/kbp in 1 Mbp windows with a 100 kbp step and their fitted components. Blue—original distribution, green—fitted distribution, red—distribution of components with their means (red triangles); (b,c) Distribution of heterozygous and homozygous (different from reference) SNP density along chromosomes, respectively. SNPs were counted in 1 Mbp windows with a 100 kbp step and scaled to SNPs/kbp (represented by the color scale on the bottom, ranging from dark blue (extremely low density) to brown (very high density), or from 0 to 0.1 to >7 SNPs/kbp, respectively). Distributions of the remaining samples are presented in File S6; (d) Local ancestry (% of sable) on chromosomes (green—sable, blue—pine marten, red—hybrid) of 1 Mbp sliding windows with 100 kbp step (chrX excluded). Local ancestry for the other samples is presented in the File S7.

The total number of heterozygous SNPs (hetSNPs) varied greatly among our samples: from 1.86 million (10xmmar) to 8.88 million (T87) hetSNPs (Table S5). By analyzing the distributions of hetSNPs (counted in 1 Mbp windows with 100 kbp steps), we detected four major modes of heterozygosity, present in various combinations in different individuals (Fig. 4a, Fig. S6). By fitting a linear combination of negative binomial distributions to our data (see SM sections S2.6 and S3.3 for the details), we resolved components of heterozygosity for all individuals and estimated corresponding mean values (Figs. S3, S10, and File S5). The first component (R) at 0 hetSNPs/kbp corresponds to regions with low heterozygosity (potential segments of runs of homozygosity, RoH) and is found in multiple samples (Fig. 4a). The second component (P), with a median value of 0.57 hetSNPs/kbp is a characteristic feature of samples initially classified as pine martens, but is also present in sympatric martens. The third component (S, median value 1.71 hetSNPs/kbp) was detected in all sable samples and the putative hybrid martens T84 (comparable level to a priori sables) and T87 (significantly lower level). The last component (H, median value 4.52 hetSNPs/kbp) is mostly associated with the putative hybrids (T78, T79, T85, T86, T84, T87) and shows a dramatic expression in samples T84 and T87. However, H is also present in several individuals initially described as sables (T18) and pine martens (S50 and T151), but later identified as backcross-like hybrids by the local ancestry analysis. Densities of the hetSNPs along the chromosomes indicate that windows belonging to a particular component form long continuous runs, which is consistent with the local ancestry (Fig. 4, File S6). Finally, the dramatic difference in heterozygosity between the sable and pine marten is statistically significant. We confirmed it for both the median heterozygosity (1.71 vs 0.62 hetSNPs/kbp, one-sided Mann–Whitney *P*-value ≤ 0.00005) and the corresponding heterozygosity components (mean *P* = 0.57 vs. mean S = 1.9 hetSNPs/kbp, Mann–Whitney *P*-value ≤ 0.00005).

### Runs of Homozygosity (RoH)

**Fig. 5.**
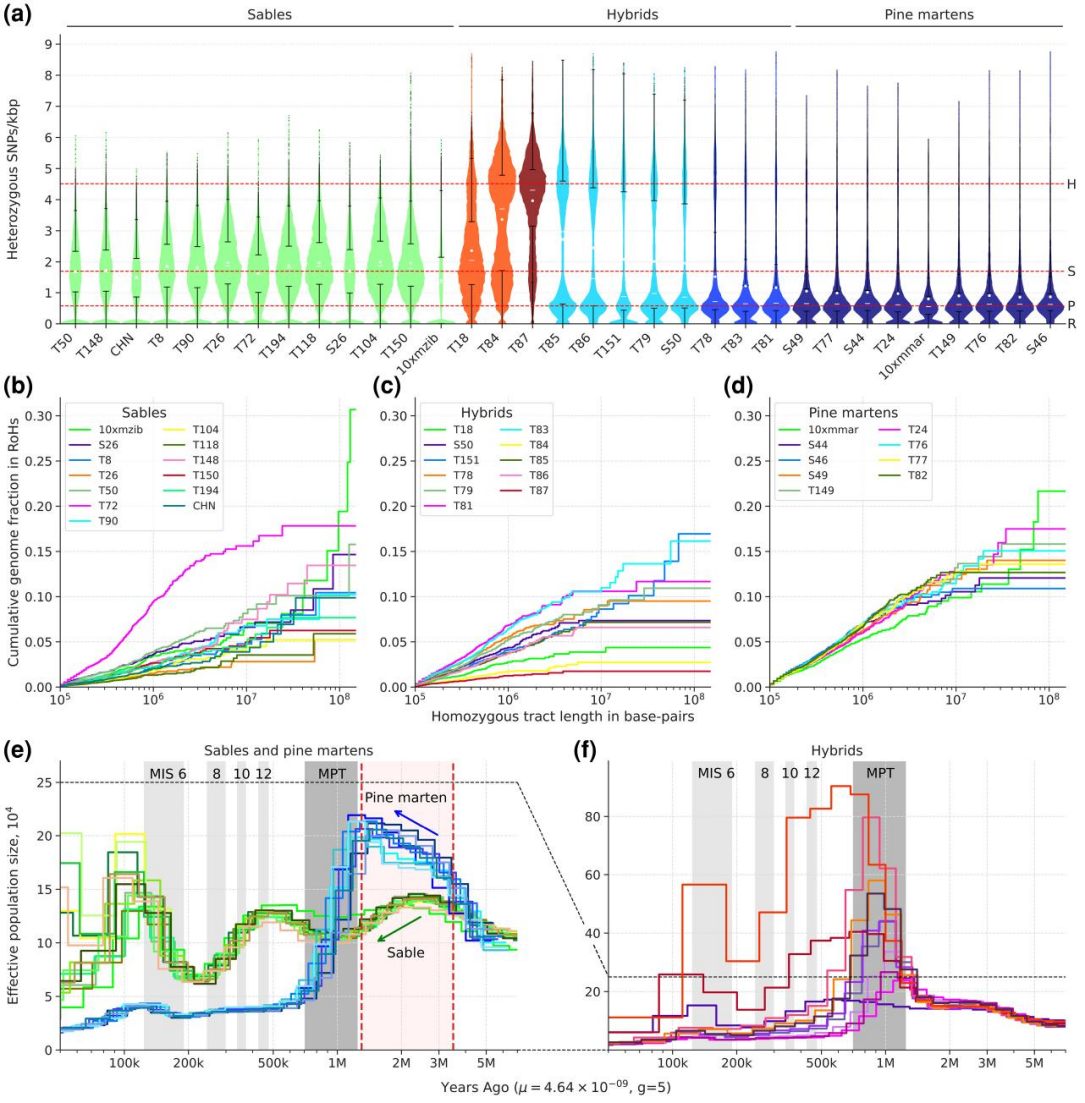
Heterozygosity, runs of homozygosity and demography. (a) Distribution of mean heterozygosity (SNP only) of the individuals counted in sliding windows of 1 Mbp with 100 kbp step size. The samples are colored according to the local ancestry: green and dark blue—pure sables and pure pine martens, respectively; blue—atypical pine martens; orange and light blue—backcross-like hybrids, respectively; and dark red—F1-like hybrids. Each distribution was scaled with respect to the *x*-axis. Horizontal red lines indicate three levels of heterozygosity (0.57, 1.71, and 4.52 hetSNPs/kbp) corresponding to the median values (among the samples) of means for pine marten (P), sable (S), hybrid (H), and RoH (R) components. Note that pane A does not display violin plots or other kernel-density representations, but rather histograms smoothed by using the bin midpoints. The bin width was set to 0.1 hetSNPs/kbp. For better resolution of the ultra-low heterozygous parts of the distributions, see Fig. S8a; (b–d) Cumulative distribution plots of RoH for the pine martens (b), the hybrids (c), and the sables (d). Homozygous tract lengths and cumulative genome fraction in RoH are represented on *X* and *Y* axes, respectively. Tracts are ordered from shortest to longest. X chromosomes were excluded from all samples. Location of the RoHs on chromosomes is presented in the File S8; (e and f) Demographic history of the sables (e, green curves) and the pine martens (e, blue curves), and the hybrids (f). Mutation rate (*μ*) = 4.64 × 10^−9^. Generation time (g) = 5. MIS—Marine Isotope Stages (only four even, i.e. cold, stages are highlighted): 6 (130 to 191 kya), 8 (243 to 300 kya), 10 (337 to 374 kya), 12 (424 to 478 kya); MPT—mid-pleistocene transition (0.7 to 1.25 Mya). Red dashed lines denote the period preceding species divergence (1.3 to 3.5 Mya). The blue and green arrows indicate the directions of demographic trajectory changes during this pre-divergence period for the pine marten and the sable, respectively. The X chromosome was excluded from the analysis.

Among the samples, we observed significant variation in both the distribution and size of RoH, which we categorized as ultra long (L ≥ 10 Mbp), long (10 Mbp > L ≥1 Mbp), and short (L < 1 Mbp; Fig. S7, Table S6). Two obvious outliers emerged based on the fraction of the genome occupied by ultra long RoH: samples 10xmzib (24.1%) and 10xmmar (11.8%), with some of 10xmzib's ultra long RoH exceeding 100 Mbp in length and nearly completely covering several chromosomes (chr3, chr6, chr11, and chr12), while in 10xmmar only chr3 exhibited a similar pattern (File S8). Cumulative distributions of RoHs (Fig. 5b–d) followed different trajectories between species, with pine martens showing higher, convex or linear patterns, while sables displayed lower, concave distributions, indicating that short and long RoH (<10 Mbp) occupy smaller genomic fractions in sables than pine martens (two-sided Mann–Whitney test, *P*-value = 0.0011), though no significant differences were detected for ultra long RoH (*P*-value = 0.12) or all RoH combined (*P*-value = 0.07). Pure sable T72 from the Kamchatka Peninsula showed a pine marten-like trajectory despite being a pure sable, while hybrid samples exhibited lower RoH numbers compared to pure species with varied trajectories depending on ancestry rates, particularly samples T87 and T84, which had significantly fewer RoH (130 and 166) with shorter total lengths (39.01 and 61.14 Mbp). The multiple shorter RoHs and low heterozygosity in the pine marten suggest a lower long-term effective population size (Ne), whereas the longer RoHs and higher heterozygosity in the sable imply a larger long-term Ne but with evidence for recent inbreeding.

### Demographic History

The pure sables and the pure pine martens as well as the hybrids, showed distinct demographic trajectories (Fig. 5e and f). The sables (Fig. 5e) demonstrated an oscillating curve with three peaks at ∼2.5 (CI: 1.57 to 3.94), ∼0.5 (CI: 0.31 to 0.79), and ∼0.1 (CI: 0.06 to 0.15) Mya. In contrast, the pine martens (Fig. 5e) showed completely different curves: starting around 5 (3.15; 7.89) Mya, their numbers increased and peaked at ∼1.3 (CI: 0.82 to 2.05) Mya. This was followed by a severe bottleneck overlapping with the MPT, when N_e_ decreased five-fold at ∼0.7 (CI: 0.44 to 1.1) Mya. Since then, the effective population size of the pine martens has been slowly decreasing, with a small peak at ∼125 (CI: 79 to 197) kya. The hybrids (Fig. 5f) revealed an interesting pattern: at ∼1.5 (CI: 0.94 to 2.37) Mya their trajectories curve steeply upward. We found that the strength (height of the peak) of this artificial “population explosion” strongly correlates (Kendall's τ = 0.67, *P*-value = 0.003; Spearman's ρ = 0.76, *P*-value = 0.006; Pearson's *r* = 0.8, *P*-value = 0.003) with the introgression level in the hybrids (Fig. S8). Finally, we note that until ∼3.5 (CI: 2.20 to 5.52) Mya all samples share nearly the same and growing trajectory (Fig. 5e, zoomed on Fig. S9).

### Fertility Related Genes

Previously reported synteny analysis of four marten species revealed and confirmed (using cytogenetic data) an inversion on chr11 between the sable and the pine marten (Tomarovsky et al. 2025b). This inversion encompasses 11.5 Mbp and occupies nearly the whole p-arm of the chromosome. Chromosomal inversions in a heterozygous state are known to alter the functional regulation of gene expression and to suppress recombination within the inverted region (so-called crossover suppression), leading to the gradual accumulation of differences between species (Berdan et al. 2023). To test this hypothesis, we analyzed the distribution of F_st_ values (the pure sables vs. pure pine martens) in sliding windows of 1 Mbp with 100 kbp step across the whole genome.

The F_st_ analysis (Fig. S10a) showed a high level of genetic differentiation between the two species, with a mean value of 0.68. We detected 14 loci (1.1 to 2.7 Mbp, Table S7) consisting of windows with high F_st_ values (≥0.9) and predominantly gene-rich (detailed analysis in SM section S2.11). However, none of them was located within the p-arm of chr11. The mean F_st_ of this region (0.74) was only slightly higher than the whole genome mean. To mitigate the effect of position on the chromosome, we compared the p-arm of chr11 with regions of similar architecture: comparable-sized p-arms of chr12, chr13, chr15, and chr18 (10 to 18 Mbp). We found that F_st_ for p-arm of chr11 is statistically significantly higher (corrected *P*-values << 0.001) than for four other chromosomes (0.74 vs. 0.53 to 0.58, Fig. S10c, Table S8). This result suggests that the inverted region has already accumulated a notable additional amount of substitutions. Within the inversion we found only 31 genes (File S9). Gene ontology (GO) analysis showed no statistically significant enrichment of GO terms, but manual examination revealed two reproduction-related genes (*SPMIP7* and *ZPBP*). Both genes are located ∼1.5 Mbp from the inversion breakpoint, and the chromosomal rearrangement shifted them from a peritelomeric position in the pine marten and stone marten (representing the ancestral state) to a pericentromeric position in the sable.

## Discussion

### Mosaic Recombinant Chromosomes in Hybrids

**Fig. 6.**
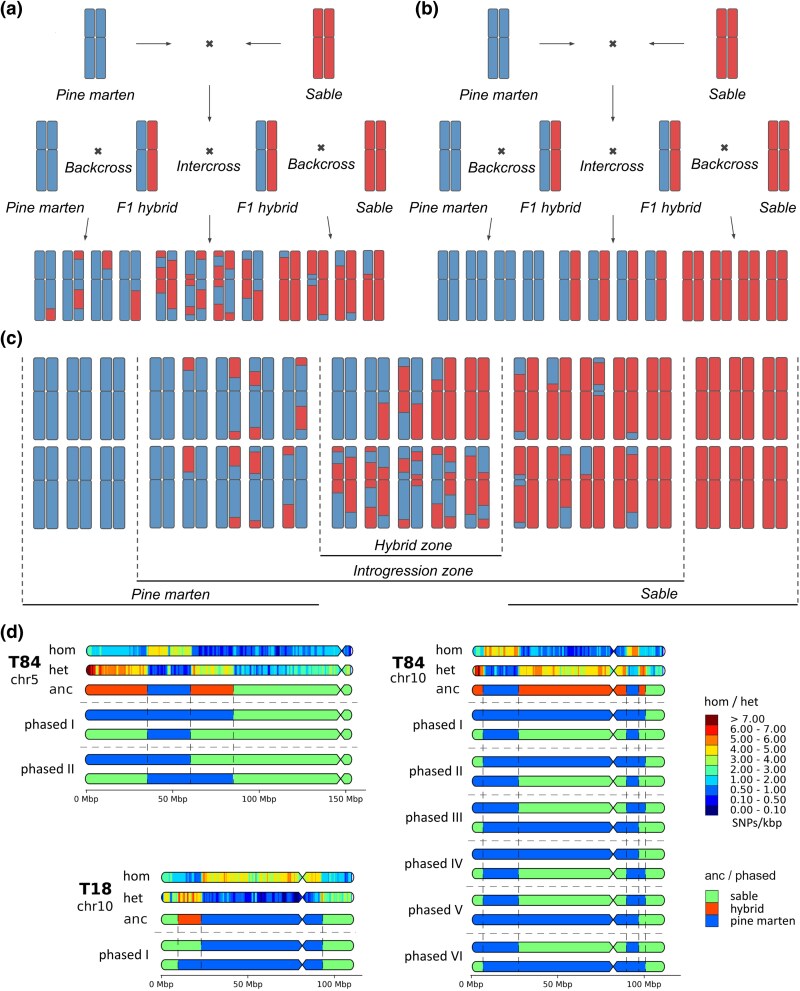
Mosaic recombinant chromosomes in context of hybridization and introgression. (a) Case of unsuppressed crossing over between homologous chromosomes of different origin; (b) hypothetical case of completely suppressed crossing over; (c) gradient of admixture (continuum border between species) between zone of sympatry and regions of pure species in case of unsuppressed crossing over; (d) mosaic recombinant chromosomes and possible variant of their phasing. Bold T** indicates the sample ID. *hom/het****—***density of homozygous and heterozygous SNPs, *anc/phased*—ancestry and phasing, respectively. SNPs were counted in 1 Mbp windows with 100 kbp step size and scaled to SNPs/kbp (represented by the color scale on the bottom, ranging from dark blue (extremely low density of hom/het SNPs) to brown (very high density of hom/het SNPs), with 0 to 0.1 and ≥7 hom/het SNPs/kbp, respectively; anc—local ancestry (% of sable) on chromosomes of 1 Mbp sliding windows with 100 kbp step; phased—possible variants of chromosome phasing.

Our analyses identified 11 hybrids using a 5% introgression threshold to distinguish individuals with pure species ancestry from those with hybrid or introgressed genomes. In our hybrid samples, we observed multiple cases of mosaic recombinant chromosomes (Fig. 6d). If crossing over was completely suppressed, we would expect sable and pine marten chromosomes to remain intact and separate in descendants (Fig. 6b), but our findings support a different model involving active recombination (Fig. 6a). Because of the nature of our dataset (short read paired-end Illumina resequencing only), we lack long-range information to phase the genetic variants. However, we can take into account the distribution of the ancestry along chromosomes and propose possible variants of the large-scale phasing (Fig. 6d). For example, the ancestry along chr 10 of backcross-like hybrid T18 suggests only a single variant of the phasing (phased I), whereas for chr 5 and chr10 of backcross-like hybrid T84, there are two (phased I and II) and six (phased I–VI) possible options, respectively. Possible phasing patterns for selected chromosomes indicate that both homologs in some chromosome pairs are recombinant and consist of multiple segments, indicating that multiple crossover events have occurred during hybrid formation (Fig. 6d). Such patterns can only arise through multiple successive crosses involving various combinations of pure species and different hybrid types, and only if crossing over is not completely suppressed. Our results suggest that the sympatric zone functions as a hybridization hotspot with introgression events radiating outward in all geographic directions (Fig. 6c, Fig. S11). We observed a continuum border between the species (Harrison and Larson 2014), characterized by a gradual transition from pine marten in the west to sable in the east (Fig. 4). Due to the limited number of samples (for species with such large ranges), we cannot accurately define the borders of the introgression zone. The eastern border is more clearly delimited due to the documented ongoing historical expansion of the pine marten range eastwards, which passes roughly along the eastern edge of the Ob River basin; in the west, it possibly expands past the known sympatry zone in the pre-Urals, i.e., to the west of the Komi Republic, Permskiy Kray, and Sverdlovsk Oblast.

### Genetic Ancestry and Discordance Between Different Datatypes

**Fig. 7.**
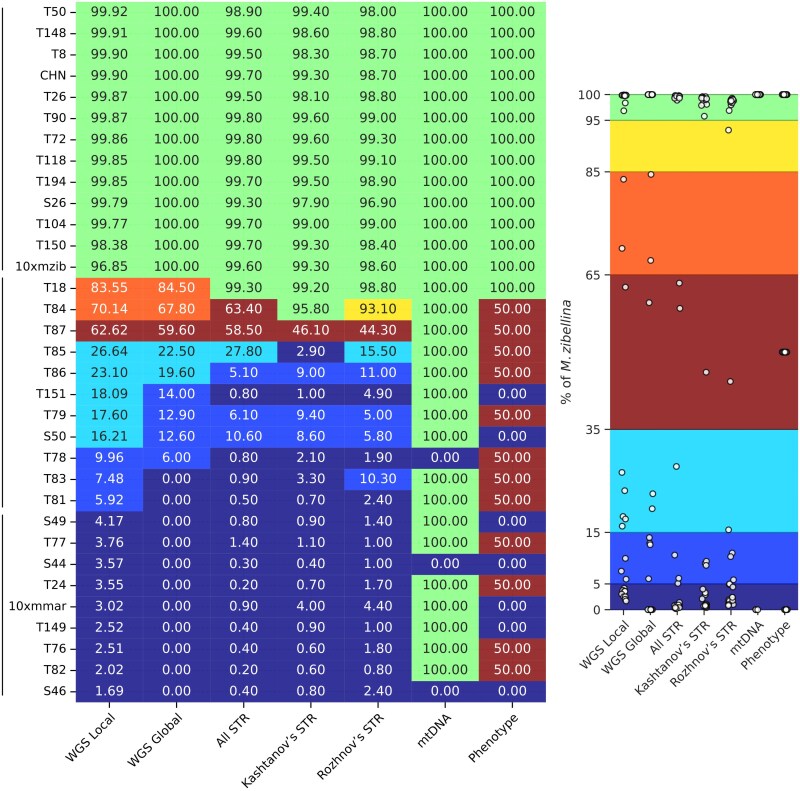
Discordance in ancestry estimates of sable × pine marten hybrids between different analytical methods and datasets. The numbers present a percentage of sable ancestry, i.e. 100% indicates a pure sable, whereas 0% indicates a pure pine marten. Abbreviations of methods and data used to calculate ancestry: WGS Local—WGS data and the local approach, WGS Global—WGS data and the global approach, All STR—complete set of all mapped STR markers, Kashtanov's STR—STR markers from (Kashtanov et al. 2022), Rozhnov's STR—STR markers from (Rozhnov et al. 2013), mtDNA—the origin of mitochondrial DNA, and Phenotype—simple phenotypic traits: tail length, fur length and quality, throat patch size, respectively.

We identified the ancestry of the sampled individuals using global and local ancestry analyses. The latter analysis successfully detected low (<7%) rates of admixture (confirmed by the heterozygosity components plus F3- and D-statistics), which the global analysis failed to detect. Our results showed that the classification of putative hybrids based on phenotypic traits was notably imprecise (Fig. 7). Moreover, even the morphometric character Δ (see SM sections S1.1 and S2.7), previously proposed as a reliable metric for distinguishing sables and pine martens (Monakhov 2021), was heavily biased toward pine marten values in putative kidases and therefore proved uninformative for hybrid verification.

The unsuppressed (at least partially) crossing over in sable × pine marten hybrids revealed in this study suggests that accurate ancestry identification requires, if not WGS data, then at least thousands of markers uniformly distributed over all chromosomes. However, there are multiple studies of hybridization between sables and pine martens, as well as on the intraspecific diversity of sables, which were performed using various sets of STRs and mitochondrial markers (Rozhnov et al. 2010, 2013; Andrianov et al. 2012; Kashtanov et al. 2015, 2022; Li et al. 2021). Moreover, such studies continue to be published, e.g. (Ranyuk et al. 2025). Application of known mustelid STR markers to classify samples in this study revealed significant discordance with WGS-based analysis for hybrid samples, primarily underestimating ancestry levels (Fig. 7). This result is expected given the limited number of STR markers, which fail to provide complete chromosomal coverage even when combined (Fig. S4). STR markers can only be considered reliable when crossing over is completely suppressed and proven coverage of all chromosomes is achieved, suggesting that previous assessments require reevaluation (Rozhnov et al. 2010, 2013; Ranyuk et al. 2025). Mitochondrial DNA obviously cannot be used for classification of hybrids, but in the case of our samples, it does not even correlate with the nuclear genome for pure martens (Fig. 7). Therefore, mtDNA of the sable and marten, and, probably, of other actively hybridizing mustelid species, should be used with precautions even in phylogenetic studies.

### Legacy of the Sable Reintroduction Program

**Fig. 8.**
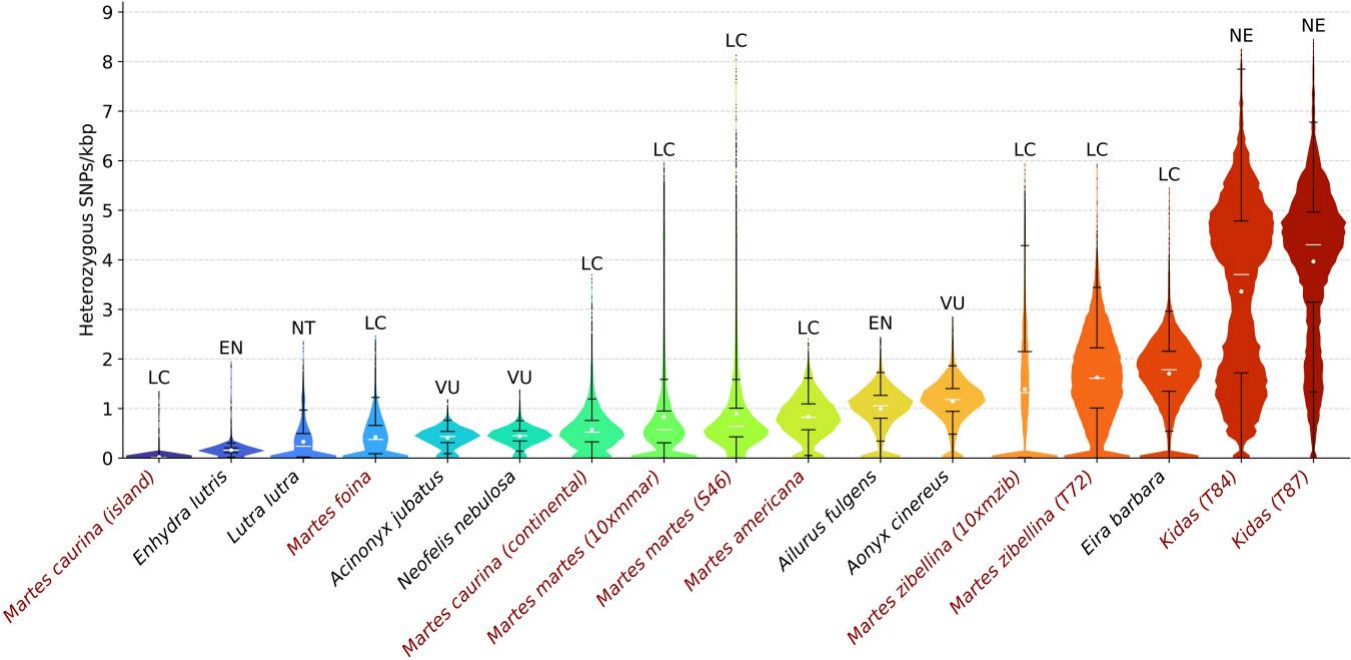
Distribution of the heterozygous SNP density in mustelids and three well-known in conservation genetics species: cheetah (*Acinonyx jubatus*), clouded leopard (*Neofelis nebulosa*), and red panda (*Ailurus fulgens*). The SNP density analysis was performed using exactly the same pipeline, tools, and settings for all species present in the figure. Distributions were counted in 1 Mbp sliding windows with 100 kbp step. Mean values are marked with white dots, medians are represented by white lines. Color gradient indicates level of heterozygosity, ranging from lower (blue) to higher (red) values. Marten species (including hybrids) are highlighted in red. American marten (*Martes americana*; SRR11575352) and Pacific marten (*Martes caurina*; SRR11575348 (island) and SRR11575351 (continental)) samples were obtained from Colella et al. (2021); *M. zibellina* genome assembly was used as a reference. Distributions for other species were obtained from (Totikov et al. 2021; Derežanin et al. 2022; Tomarovsky et al. 2025a). Abbreviations for Global conservation status according to IUCN Red List data: LC—Least Concern, NT—Near Threatened, VU—Vulnerable, EN—Endangered, NE—Not Evaluated.

We found that the sable exhibits unexpectedly high heterozygosity (1.5 to 1.8 hetSNPs/kbp) given its documented history of population declines due to fur harvesting. It is comparable to the heterozygosity (1.78 hetSNPs/kbp) of the tayra (*Eira barbara*), a neotropical gulonine that experiences some pressure from humans, but was never extensively hunted (Derežanin et al. 2022). Moreover, it is approximately three times higher than in the pine marten (Fig. 8). The concave сumulative RoH trajectories of pure sable samples, except one, are also very different from those of the pure pine martens (Fig. 5b and d). We suspect that interbreeding with reintroduced sable individuals, mostly from the Barguzin (*M. z. princeps*) and Vitim (*M. z. vitimensis*) subspecies (Timofeev and Nadeev 1955; Monakhov 2011), may have introduced new genetic diversity, increasing genetic variation, thereby reducing the probability of consanguinity, which in turn could explain the low RoH content.

Among the sables we examined, the pure sable T72 (∼1.6 hetSNPs/kbp) from Kamchatka is worth highlighting, as it has a cumulative RoH trajectory very different from that of other sables (Fig. 5b), but similar to that of the pine martens (Fig. 5d). The Kamchatkan population remained relatively stable throughout the bottlenecks and was used to restock some areas in Eastern Siberia (Magadan Oblast, parts of Yakutia, etc.) where the sable had nearly disappeared (Sablina 1958). This supports the hypothesis that Kamchatka may still harbor an autochthonous population not affected by admixture with other subspecies, consistent with previous studies that have highlighted distinctive features of Kamchatka sables (Malyarchuk et al. 2010, 2014; Kinoshita et al. 2015; Li et al. 2021). Following this hypothesis, the similar heterozygosity of T72 and sables from the Western and Central Siberia, regions where sables experienced severe population declines due to overharvesting, suggests that these results reflect the success of the reintroduction programs. However, our hypothesis should be interpreted with caution, as we have sequenced a limited number of sables, and only one of them belongs to the Kamchatka population.

### Displacement of the Pine Marten mtDNA

Surprisingly, only two pure pine martens (S44 and S46) and only one hybrid (T78) have pine marten mtDNA (Fig. 3). Moreover, our pure pine martens have mtDNA from four different sable clades: A3, A5, B2, and C4 (Fig. 3, Fig. S5), which implies that interspecific mtDNA introgression has occurred multiple times. Such a phenomenon might be explained by the biological and behavioral features of these species. Sable is hypothesized to be better adapted to life in deep snow, and several morphological traits, such as its shorter tail, thicker fur, thicker-furred soles, and better-developed strips of bristly fur on the feet (“snowshoes”), are indirect evidence of this (Timofeev and Nadeev 1955). In regions with harsh, snowy winters, such as the sympatric zone, pine martens may be vulnerable in direct confrontations with sables, particularly smaller females. Some sources claim sables are more aggressive to conspecifics than pine martens, with cases of intraspecific killings being exceptionally rare in pine martens but common in sables. In fact, sables cannibalizing trapped conspecifics is a common occurrence and has been attributed to territorial behavior rather than hunger-motivated carnivory (Buskirk et al. 1994). Therefore, female pine martens might be casualties of interspecific competition in the sympatric zone. Also, it implies that sable mtDNA might have spread far West of the sympatric zone and that pine marten mtDNA should only be occasionally found in the East. Despite the limited sample size of our dataset, we see such a trend in our results: two martens (the pure pine marten S49 and the backcross-like hybrid S50) collected in the far West have sable mtDNA, and no specimen collected in the East has pine marten mtDNA.

### Ancient Roots of Low Heterozygosity in the Pine Marten

Surprisingly, we found that the pine marten is significantly less heterozygous than the sable (Fig. 5a, Fig. S10). Its median heterozygosity of ∼0.5 to 0.6 hetSNPs/kbp (Fig. 5a, Fig. S11) is smaller than in some endangered species, for example, the red panda (1.05 hetSNPs/kbp) and Asian small-clawed otter (1.18 hetSNPs/kbp; Fig. 8). It is only 40% higher than the heterozygosity of such iconic species of conservation biology and genomics as the cheetah (0.44 hetSNPs/kbp) and clouded leopard (0.45 hetSNPs/kbp). Our demographic reconstruction (Fig. 5e) suggests that it is a consequence of the severe population decline during the MPT (0.7 to 1.25 Mya) and, therefore, is an ancient trait of the species rather than a modern feature. This is also consistent with the presence of multiple, but short RoHs. Given its range, census numbers, and no reports of inbreeding depression, we consider it a successful species, which probably purged most of the recessive lethal alleles from its genome, similar to the Channel Island fox (*Urocyon littoralis*; Robinson et al. 2016, 2018). An expanded dataset and further study are necessary to confirm this hypothesis. Moreover, all our conclusions and interpretations apply specifically to Eastern pine marten populations, while the dynamics of Western populations remain poorly understood.

### Demography and Speciation

The trajectories of effective population size of sables and pine martens are markedly different (Fig. 5e), although they show similar curves before 3.5 Mya (Fig. S9). We hypothesize that the significant increase in N_e_ observed in pine martens (1.3 to 3.5 Mya, Fig. 5e) was associated with the colonization of new territories by ancestral pine martens, supported by the strong unidirectional gene flow from the “mainland” area inhabited by the shared ancestors with the sable. The subsequent decrease of the pine marten's N_e_ during the MPT (0.7 to 1.25 Mya, can be considered not only as a potential result of climatic changes, but also indicative of isolation from ancestral sables, as the N_e_ of sables drops negligibly around the same time period (Fig. 5e). Isolation in different refugia during the Pleistocene glaciation(s; Ruiz-González et al. 2013) may have served as a driver of speciation that severed the gene flow between the two diverging species. We speculate on associated hypothetical migration routes in SM (section S2.10).

The fossil calibrated tree (Fig. 1b) estimated the split between the sable and pine marten at 1.52 Mya (CI: 1.05–2.06 Mya), which substantially overlaps with the dramatic drop of the pine marten's N_e_ at 1.3 Mya (CI: 0.82–2.05 Mya). This divergence has been estimated in at least four previous genetic studies (Table S9; Koepfli et al. 2008; Li et al. 2014a; Law et al. 2018; Hassanin et al. 2021). Hassanin et al. (2021) and Li et al. (2014a) used complete mitochondrial genomes (∼16 kbp), Koepfli et al. (2008) analyzed 21 nuclear gene segments and 1 mitochondrial gene (∼12 kbp), while Law et al. (2018) employed 4 mitochondrial genes and 42 nuclear gene segments (∼35 kbp). Most of these studies agree that the MRCA of sable and pine marten existed ∼1.1 Mya (CI: ∼0.6–1.6 Mya), with the exception of Li et al. (2014), who estimated substantially younger divergence of 0.68 Mya (CI: 0.54–0.84 Mya). Interestingly, for the MRCA of all three marten species (including the stone marten, *Martes foina*), the outlier dating estimate belongs to Hassanin et al. (2021) (5.1 Mya, CI unavailable), whereas the other studies estimated it no older than 2.93 Mya (Li et al., CI: 2.35–3.54 Mya) or even younger, at 2.8 Mya (Koepfli et al., CI: 1.9 to 3.8 Mya) or 2.56 Mya (Law et al., CI: 1.93–3.29 Mya). Our mean estimate of 2.83 Mya and the associated confidence intervals (CI: 2.11–3.66 Mya) are consistent with these values. In contrast to the earlier studies, our phylogenetic analyses employed significantly more sites: ∼100 × more sites (1.38 Mbp) than the mtDNA genome-based works and ∼40 × more than the study by Law et al. (2018). Moreover, we utilized only clockwise sites—fourfold-degenerate sites from the third codon positions of single-copy genes. Another potential source of discrepancy may be incomplete lineage sorting or ancient hybridization with the lineages leading to American and/or Japanese martens, which could have affected mtDNA and the small set of nuclear genes used by Law et al. (2018).

### Candidate Genes Related to the Fertility Issues

We found that two fertility-related genes, *SPMIP7* and *ZPBP*, are located within previously described and cytogenetically confirmed 11.5 Mbp inversion between the sable and pine marten encompassing the p-arm of chr 11 (Tomarovsky et al. 2025b). *SPMIP7* (Spermatozoon Microtubule Inner Protein 7) is essential for normal spermatogenesis (Leung et al. 2023), while *ZPBP* (Zona Pellucida Binding Protein) functions in both spermatogenesis and sperm-egg binding (Lin et al. 2007). In humans, mutations in *ZPBP* are associated with spermatogenic failure-66 (SPGF66) disorder and male infertility due to globozoospermia (Oud et al. 2020). Inversions of such size are also known to result in the accumulation of multiple substitutions (Wellenreuther and Bernatchez 2018) due to completely suppressed recombination, as their length is insufficient to form the characteristic inverted loop during meiosis (Anton et al. 2005). The notably higher Fst values on the p-arm of chr 11 relative to those on other chromosomes (see Results) support this, although the estimates may be influenced by differences in within-species diversities (Charlesworth 1998; Cruickshank and Hahn 2014). Nevertheless, the chromosomal environment of *SPMIP7* and *ZPBP* differs between the two species, which may influence their regulation (Runemark et al. 2025) and could potentially contribute to the reported fertility issues in kidases.

## Conclusion

We conducted a thorough genomic investigation into the hybridization between the sable and pine marten; however, many of the patterns revealed warrant further study. The first set of new questions is directly related to the hybrids. We observed bidirectional introgression, including the substitution of marten mtDNA by sable mtDNA in many samples extending far beyond the sympatric zone. This suggests that current species boundaries may be obsolete and require reevaluation. The notable discrepancy between our results and previous mtDNA-based divergence dating necessitates an investigation into potential ancient gene flow from other species. Such introgression, for example, from a closely related *M. americana* and *M. caurina* hybrid system, may bias estimates. However, such a complex interspecies study should be studied using a pangenome approach instead of the usual linear reference(s). In our study, we performed all the analyses twice, using both sable and pine marten genome assemblies as references, to confirm the absence of the reference-related biases. The pangenome methodology is free from such issues. Second, the lower heterozygosity in the pine marten is surprising, as from the history of both species, we expected the opposite pattern. Our samples cover only the eastern part of the range, so the diversity of western populations remains unclear. Our dataset, while spanning much of both species’ ranges, included only 33 individuals; thus, for such widely distributed species, this study should be considered a pilot investigation. Moreover, it is still unclear how the population declines and one of the largest reintroduction programs in history has affected the subspecies and population structure, and whether the diversity was indeed restored or not. Our findings provide reasons for cautious optimism, but the heterozygosity of sables before the population declines of the 19th and 20th centuries remains unknown. To address these questions, a broader sampling and sequencing of hundreds of individuals, including museum and ancient samples, is required. Finally, we have taken the initial step in investigating the fertility issues in hybrids and identified two candidate reproduction-related genes (*SPMIP7* and *ZPBP*). Future investigations will necessitate detailed gene sequence analysis, transcriptomic, and breeding experiments. Our work thus establishes a foundation for at least four research directions and highlights the appropriate methodologies that can be used to pursue them.

## Materials and Methods

### Samples, DNA Extraction and Sequencing

For whole genome resequencing, we used previously collected tissue samples from the Novosibirsk Cell Line Collection located at the Institute of Molecular and Cellular Biology, Siberian Branch of the Russian Academy of Sciences (IMCB SB RAS). The initial classification (pure/putative hybrid) of samples (Table S1) was based on the descriptions of simple morphological traits distinguishing sables, pine martens, and kidases: tail length, fur length and quality, and throat patch size (Kassal and Sidorov 2013). DNA extraction was performed using the standard phenol-chloroform protocol (Sambrook and Russell 2006). The libraries for resequencing were constructed using the TruSeq DNA PCR-Free kit (Illumina, Inc, San Diego, CA, USA). All prepared libraries were sequenced with paired-end 150 bp reads on the Illumina NovaSeq 6000 or Illumina HiSeq X Ten platforms. All manipulations with the samples were performed according to the permission of IMCB Ethical Committee № 01/21 issued on January 26, 2021.

### Data Quality Control, Filtration and Genome Size Estimation

Our dataset consisted of 33 samples, including two previously sequenced martens (Tomarovsky et al. 2025b). Published samples (10xmmar (SRR22412800) and 10xmzib (SRR22412799)) were sequenced using 10X Genomics linked reads, and therefore included barcoding at 5′ ends of the forward reads, which we trimmed using EMerAld (EMA) v0.6.2 (Shajii et al. 2017) before processing. Initial quality control of raw and filtered data was performed using FastQC v0.11.9 (Andrews 2010). Trimming of Illumina adapters and filtering based on read quality was performed in two stages with initial 23-mer-based trimming of large adapter fragments using Cookiecutter v1 (Starostina et al. 2015), followed by trimming of small fragments and quality filtering using Trimmomatic v0.36 (Bolger et al. 2014) with the following parameters: “ILLUMINACLIP:TruSeq2-PE.fa:2:30:10:1 SLIDINGWINDOW:8:20 MINLEN:50.” Distributions of 23-mers after read filtration were counted using Jellyfish v2.3.0 (Marçais and Kingsford 2011) with the parameters “-m 23 -s 30G” for *jellyfish count* and “-l 1 -h 100000000” for *jellyfish histo*. Then, we visualized the distributions using KrATER v2.6.1 (Kliver et al. 2017) with the parameters “-m 23 -u 1.” Genome size estimation and coverage based on paired-end reads was performed using KrATER, incorporating GenomeScope2 v2.0.1 (Ranallo-Benavidez et al. 2020) in diploid mode with a k-mer length of 23. To achieve equal coverage and maximize similarity among all samples, we downsampled them to 22 × coverage using the *reformat.sh* script with the “samplerate=” parameter from BBmap v38.96 (Bushnell 2014). The downsampling fraction was calculated as the ratio of the desired coverage to the initial coverage of the sample.

### Phylogenetic Analyses

For estimation of divergence times we reconstructed a phylogenetic tree using the BuscoClade pipeline v1.7 (https://github.com/tomarovsky/BuscoClade), based on a multiple codon alignment of conserved single-copy orthologous gene coding sequences (BUSCOs) and genome assemblies of nine species: the sable, the pine marten, the stone marten, the wolverine, the tayra, the Canadian river otter, the sea otter, the European badger and the red panda (Dudchenko et al. 2017; Hu et al. 2017; Jones et al. 2017; Derežanin et al. 2022; Lok et al. 2022; Newman et al. 2022; A. Tomarovsky et al. 2025a; Tomarovsky et al. 2025b). The selection of species and details on the reconstruction procedure are provided in sections 2.4 and 3.1 of SM. Divergence times were dated using the MCMCTree tool from PAML v4.10.7 (Yang 2007) and the fossil-based calibrations listed in Table S3 (Stach 1959; Wolsan 1990; Wang et al. 2005; Salesa et al. 2013; Samuels and Cavin 2013; Li et al. 2014a; Law et al. 2018; Marciszak et al. 2024). First, we extracted 4-fold degenerate sites (1,384,523 bp) from the concatenated alignment used to reconstruct the tree. Next, we performed analyses twice (according to the recommendations from authors of the MCMCTree tool) for each of the three molecular clock types (global, correlated, and independent) with 2,200,000 MCMC generations. The first 200,000 generations were discarded as burn-in. We found no discordance between the two runs for each of the clock model analyses. We verified satisfactory convergence of parameters using Tracer v1.7 (Rambaut et al. 2018) for each of the runs. Visualization of the dated phylogenetic tree was performed using FigTree v1.4.4 (Rambaut 2018). All phylogenetic trees generated in this study are included in File S10.

### Reference Genome Assemblies, Alignments, Coverage and PAR Identification

Filtered reads from the 33 samples were aligned to the reference assemblies of both sable (10xmzib, GCA_040938815.1) and pine marten (10xmmar, GCA_040938825.1; Tomarovsky et al. 2025b) using BWA v0.7.11 (Li and Durbin 2009) with default parameters. All the downstream analyses were performed twice for each of the references and compared. Read processing (pairing, sorting, quality filtering, marking duplicates, and indexing the alignments) was performed using Samtools v1.15.1 (Li et al. 2009). Per-base genome coverage was calculated using Mosdepth v0.3.0 (Pedersen and Quinlan 2018) with the parameter “–mapq 10.” Individual genome coverage masks for each sample were obtained based on Mosdepth assessments using the *generate_mask_from_coverage_bed.py* script from the MAVR v0.97 package (https://github.com/mahajrod/MAVR) with the parameters “-x 2.5 -n 0.33,” corresponding to coverage thresholds of >250% and <33% of the median genome coverage. To set the correct ploidy for the X chromosome during variant calling, we identified the coordinates of the PAR in male samples based on individual coverage masks using the *pseudoautosomal_region.py* script from the Biocrutch v1.0 package (https://github.com/tomarovsky/Biocrutch). The algorithm is described in the SM section S3.4.

### Variant Calling and RoHs

Variant calling for all samples was performed using Bcftools v1.15.1 (Li 2011) with parameters: “-d 250 -q 30 -Q 30 –adjust-MQ 50 -a Ad,INFO/Ad,ADF,INFO/ADF,ADR,INFO/ADR,DP,SP,SCR,INFO/SCR -O u” for *bcftools mpileup* and “–group-samples - -m -O u -v -f GQ,GP” for *bcftools call*. Low-quality genetic variants were removed using the *bcftools filter* with the following thresholds (“*QUAL < 20.0 || (FORMAT/SP*  *>*  *60.0 | FORMAT/DP*  *<*  *5.0 | FORMAT/GQ*  *<*  *20.0)*”), and genotypes failing the FORMAT-level criteria were set to missing using the “*–set-GTs .*” option. The PAR coordinates were specified for variant calling via the “–ploidy-file” parameter. The genetic variants in regions with too high and too low coverage were removed using the unified mask (described below) and BEDTools v2.31.0 (Quinlan and Hall 2010) with the default parameters. The filtered and masked genetic variants for each sample were divided into heterozygous and homozygous single-nucleotide polymorphisms (SNPs) and insertions/deletions (indels) using the bcftools filter with the parameters “-i ‘TYPE=″snp″',” “-i ‘TYPE=″indel″'”, “-i ‘FMT/GT=″het″'” and “-i ‘FMT/GT=″hom″'.” All subsequent analyses were performed using only filtered and masked SNPs, which were counted in overlapping 1 Mbp sliding windows with a 100 kbp step size. As some homologous scaffolds in the genome assemblies had different orientations, we inverted corresponding pine marten chromosomes. In such cases, recalculation of SNP coordinates was performed using Picard v.2.27.4 LiftoverVcf (Anon 2019). The visualization of SNP density heatmaps was performed using the *draw_variant_window_densities.py* from the MACE v1.1.32 package (Kliver 2024) with additional masking of windows with ≥75 kbp, i.e. >7.5% of 1 Mbp windows, of high or low coverage bases according to the unified mask (described in the next section).

RoHs were identified based on the previously calculated heterozygous SNP densities in overlapping 100 kbp sliding windows with a 10 kbp step size. The algorithm was implemented using the *get_ROH_regions.py* script from the Biocrutch v1.0 package (https://github.com/tomarovsky/Biocrutch), which filters windows by level of heterozygosity and merges them into a final RoH file. A window was considered to be a RoH if its heterozygosity didn’t exceed 0.05 hetSNP/kbp. For the justification of the threshold, see SM Section S2.5 and Fig. S8a. Windows with intermediate heterozygosity (between 0.05 and 0.1 hetSNP/kbp) were also treated as RoH windows, but only if there were no more than five such windows in a row. Windows with higher heterozygosity (greater than 0.1 hetSNP/kbp) were excluded. Next, the filtered windows were merged together if the distance between them (between the end of the previous window and the start of the next one) was less than half of the window size. The X chromosome was excluded from the RoH analysis. Visualization of RoH distribution along the chromosomal scaffolds was performed using the *draw_features.py* script from the MACE v1.1.32 package (Kliver 2024).

### Classification of Individuals and Introgression Analysis

As input for population structure and ancestry analysis, we used the filtered SNPs as described in the previous section. However, instead of individual per-sample masks, we generated a unified mask based on pairwise intersections of individual per-sample coverage masks (see *Alignments, coverage, and PAR identification*) using BEDTools v2.31.0 (Quinlan and Hall 2010) followed by merging of all intersections. The resulting mask included genomic regions with excessively high (>250%) or excessively low (<33%) coverage in at least two samples. Filtered and masked SNP data were further filtered using PLINK v1.9 (Purcell et al. 2007). This involved filtering by a minimum minor allele frequency of 0.03 (“–maf 0.03”) and retaining only variants with a genotyping rate of at least 95% across all samples (“–geno 0.05”). Next, we pruned SNPs with a high level of pairwise linkage disequilibrium (LD) in sliding windows of 50 SNPs with a step size of 10 SNPs using an r^2^ threshold of 0.2 (“–indep-pairwise 50 10 0.2”). To analyze introgression and hybridization, we performed multiple types of analyses and tests. PCA was performed using PLINK (“–pca” option; Purcell et al. 2007). Genome-wide ancestry (global ancestry) was performed using ADMIXTURE v1.3.0 (Alexander et al. 2009) for K values (number of populations) ranging from 2 to 5, with three replicates each. We chose the K with the minimal CV mean error (calculated by ADMIXTURE) and maximal average pairwise similarity. For K = 2, we performed a local ancestry analysis to identify introgressed regions in the putative hybrid samples. The reference genome was split into 1 Mbp sliding windows with a 100 kbp step size, and ancestry analysis was performed independently for each window. The visualization of introgressed regions along the chromosomal scaffolds was performed using the *draw_variant_window_densities.py* script from the MACE v1.1.32 package (Kliver 2024). To identify Ancestry-Informative Markers (AIMs), calculate interclass heterozygosity, and generate triangle plots, we used the triangulaR package v1.0 (“diff=0.75”; Wiens et al. 2025).

To statistically assess hybridization and gene flow, we repeated the variant calling, this time including *Martes foina* (NCBI SRA ID SRR22412409), which was used as an outgroup in subsequent analyses. SNP filtering, masking, and pruning were performed following the same procedure described in the *Variant calling and runs of homozygosity* section. Hybridization detection was performed using HyDe v.0.4.3 (Blischak et al. 2018) with default parameters for the pure sables and the pure pine martens as parental groups (P1 and P2, respectively) and hybrids as a target. The tests were performed for both all hybrids together as a group and each individual separately. To detect introgression and gene flow, we calculated F3- and D–statistics using AdmixTools v.7.0.2 (Patterson et al. 2012) with default parameters. First, we performed F3 tests for all possible permutations of source and target groups for the hybrids (together as a group) and the pure species. Next, we designated the pure sables as the source 1, the pure pine martens as source 2, and tested all the individuals (both hybrids and the pure species) separately. For the D-statistics, *M. foina* was designated as Z (outgroup) for all tests. Like for F3-statistics, we first analyzed all permutations of the pure species and hybrids as W, X, and Y groups. For individual-level tests, a target individual was always used as W, whereas the pure sables and the pure pine martens were first used as X and Y, and then rearranged (File S1). For each analysis, when a pure individual was tested, it was excluded from the corresponding source pure species group.

### STR-typing and STR-based Ancestry Analysis

Primers from 79 previously identified STR loci (File S11) were compiled from the literature for eight distinct mustelid species—European pine marten (*M. martes*), stone marten (*M. foina*), American marten (*M. americana*), wolverine (*Gulo gulo*), American badger (*Taxidea taxus*), European badger (*Meles meles*), American mink (*Neogale vison*), and ermine (*Mustela erminea*; Davis and Strobeck 1998; Fleming et al. 1999; Domingo-Roura 2002; Vincent et al. 2003; Basto et al. 2010; Natali et al. 2010). Next, an *in silico* PCR with selected primers was performed for the reference assemblies of the pine marten and sable using Simulate_PCR 1.2 (Gardner and Slezak 2014). No more than four mismatches between the target sequence and each primer were allowed. The amplicon length was limited to 50–1,000 bp. Further filtration of the amplicons was performed as previously described (Totikov et al. 2021). Finally, remaining markers were manually investigated in both genome assemblies for repeat motif, repeat length, and number of repeats. The loci with STRs longer than 100 bp, with 2 or more STRs, or without STRs were excluded from all the downstream analyses as well as STRs that were X-linked (SM section 3.2).

The STR genotyping methodology is described in detail in SM section S3.5. Initially, for each sample and STR locus, we extracted reads aligned to it, including 1,000 bp flanking regions, using Samtools v1.19.2. We then verified pairing with Bazam v1.0.1, remapped them to the reference genome using BWA v0.7.17, and performed indel-aware realignment using IndelRealigner from GATK v3.7. (McKenna et al. 2010; Sadedin and Oshlack 2019). Next, we performed STR genotyping using hipSTR v0.6.2 (Willems et al. 2017). In the next stage, in addition to the full set of STR loci, we created two subsets with intersections between the full STR set and the STR sets used in Rozhnov et al. (Rozhnov et al. 2013) and Kashtanov et al. (Kashtanov et al. 2022; File S2). Downstream analyses were performed for all three STR sets. Finally, we performed the STR-based ancestry analysis using STRUCTURE v2.3.4 and Clumpp v1.1.2 (Pritchard et al. 2000; Jakobsson and Rosenberg 2007). All stages were integrated in the SnakeSTR v1.0 pipeline (https://github.com/mahajrod/snakeSTR).

### Demographic Reconstruction

To infer the historical dynamics of the effective population size (N_e_) for each species, we used the Pairwise Sequentially Markovian Coalescent software package (PSMC) v0.6.5 with the parameters “-N25 -t15 -r5 -p “4 + 25*2 + 4 + 6″” (Li and Durbin 2011). The consensus diploid sequences (input for PSMC) were created using Samtools v0.1.19 (Li et al. 2009) with the alignment quality parameter “-C 50” for *samtools mpileup* and the variant calling parameter “-c” for *bcftools view*. Diploid consensus sequences were generated using *vcfutils.pl vcf2fq* from Samtools, with minimum (“-d”) and maximum (“-D”) coverage thresholds for each sample. These thresholds were defined as one-third and 2.5 times the median genome coverage of a sample, respectively. Positions with coverage values outside this range were excluded. Fasta-like input files were generated using *fq2psmcfa* with a minimum base quality threshold set to “-q20.” PSMC bootstrap replicates for each sample were created by segmenting the sequences using *splitfa* and performing 100 bootstrap replicates (“-b 100”). Generation time (g) was set to 5 years (Colella et al. 2021), and mutation rate (*μ*) to 4.64 × 10^−9^ (Bergeron et al. 2023). Values of 2.94 × 10^−9^ and 7.37 × 10^−9^ were used as the lower and upper limits of the confidence interval (CI) for *μ*, respectively (Bergeron et al. 2023).

### Mitochondrial Genome Assemblies and Analysis

Complete mitochondrial DNA (mtDNA) genomes were assembled using MitoZ v2.3 (Meng et al. 2019) with the parameters “all –genetic_code 2 –clade Chordata –insert_size 350 –requiring_taxa ‘Mammalia'.” We also extracted and assembled four mtDNA genomes of sable from reads previously published by Liu et al. (PRJNA495455; Liu et al. 2020) and Manakhov et al. (SRR13213810, SRR13213811, SRR13213812; Manakhov et al. 2021). Before assembly, all samples were downsampled to 20,000,000 reads using the *reformat.sh* script with the “samplereadstarget=” parameter from BBmap v38.96 (Bushnell 2014). All publicly available mtDNA genomes for sables and pine martens from the NCBI database were included in the analysis using the query “(‘Martes zibellina’[Orgn] OR ‘Martes martes’[Orgn]) AND (mitochondrial OR mitochondrion) AND 10000:20000[SLEN]” (Yu et al. 2011; Xu et al. 2012; Malyarchuk et al. 2014; Li et al. 2014b, 2021; Hua et al. 2017). To ensure that all the sequences begin at the tRNA-Phe start codon (nucleotide position 1) and have the same orientation, a manual preprocessing was performed: sequences were reverse-complemented and/or adjusted as necessary. A total of 140 mtDNA genomes were used in the analysis (File S3).

The mtDNA genome sequences were aligned using MAFFT v7.490 (Katoh et al. 2002) with default parameters, followed by the filtration of the hypervariable regions of the control region and other poorly aligned regions using TrimAl v1.2rev59 (Capella-Gutiérrez et al. 2009) with the parameters “-automated1 -nogaps.” The multiple sequence alignment length before and after filtering was 17,326 bp and 15,701 bp, respectively. Next, we reconstructed a maximum likelihood tree using IQ-TREE v2.2.0 (automatic model selection, 1000 ultra-fast bootstrap replicates). *M. foina* (NC_020643.1) was used as an outgroup to root the tree. The filtered mtDNA sequences were clustered using CD-HIT v4.8.1 (Fu et al. 2012) with options “-c 1.0 -s 1.0 -S -aL 1.0 -aS 1.0” to count identical sequences and assign haplotype IDs. Next, a haplotype network was constructed and visualized using the median joining approach implemented in PopArt v1.7 (Leigh and Bryant 2015). The input file for PopArt is provided in File S12.

### Fst and Tajima's D

Fst and Tajima's D statistics were calculated using masked SNPs (unified mask) and Vcftools v0.1.16 and VCF-kit v0.2.9 (Danecek et al. 2011; Cook and Andersen 2017), respectively, in 1 Mbp sliding windows with a 100 kbp step size, using the sable genome assembly as a reference. The comparison was performed between pure sables and pure pine martens (maximum 5% of the introgression according to the local ancestry analysis of WGS data). Genome windows containing more than 607,592 bp (see SM section S3.6 for detailed description of the threshold selection) repetitive sequences were excluded from the downstream analyses.

## Supplementary Material

evag018_Supplementary_Data

## Data Availability

Resequencing data are available from BioProject: PRJNA1102534. Mitochondrial genomes generated in this study are available at the following accession numbers: PP934007-PP934038. Mitochondrial genomes assembled from publicly available resequencing data are available in the GenBank Third Party Annotation database under the following accession numbers: PP960514-PP960517.
