# Correction to: Genomics of Sable (*Martes zibellina)* × Pine Marten (*Martes martes*) Hybridization

**DOI:** 10.1093/gbe/evag189

**Published:** 2026-07-27

This is a correction to: Andrey A Tomarovsky, Azamat A Totikov, Tatiana M Bulyonkova, Polina L Perelman, Alexei V Abramov, Natalia A Serdyukova, Aliya R Yakupova, Dmitry Prokopov, Violetta R Beklemisheva, Mikkel-Holger S Sinding, Guzel Davletshina, Maria Pobedintseva, Ksenia Krasheninnikova, Daniel W Foerster, Anna S Mukhacheva, Alexandra Mironova, Michail Sidorov, Wenhui Nie, Jinhuan Wang, Svetlana A Romanenko, Anastasiya A Proskuryakova, Malcolm Ferguson-Smith, Fengtang Yang, Nikolay Cherkasov, Elena Balanovskaya, M Thomas P Gilbert, Innokentiy M Okhlopkov, Anna Zhuk, Alexander S Graphodatsky, Roger Powell, Klaus-Peter Koepfli, Sergei Kliver, Genomics of Sable (*Martes zibellina)* × Pine Marten (*Martes martes*) Hybridization, *Genome Biology and Evolution*, Volume 18, Issue 3, March 2026, evag018, https://doi.org/10.1093/gbe/evag018

In the originally published version of this manuscript, the affiliation provided for the third co-author was incorrect.

This affiliation has been corrected to read “Laboratory of System Dynamics, A. P. Ershov Institute of Informatics Systems, 630090 Novosibirsk, Pr. Lavrentieva, 6, Russia” instead of “Youth Laboratory of Molecular Genetics, Yugra State University, Khanty-Mansiysk 628011, Russia”.

