# Supplementary material for: Genomics of Sable (Martes zibellina) × Pine Marten (Martes martes) Hybridization
Source: Genome Biol Evol. 2026 Mar 5;18(3):evag018. doi: 10.1093/gbe/evag018 (PMC12960073; doi:10.1093/gbe/evag018)
Supplement: evag018_Supplementary_Data [file evag018_supplementary_data.zip › SupplementaryFiguresAndTables.pdf]

# Supplementary Figures and Tables

Andrey A. Tomarovsky[1,2*], Azamat A. Totikov[1,2x], Tatiana M. Bulyonkova[3x], Polina L. Perelman[1], Alexei V. Abramov[4], Natalia A. Serdyukova[1], Aliya R. Yakupova[5,6], Dmitry Prokopov[7,8], Violetta R. Beklemisheva[1], Mikkel-Holger S Sinding[9,10], Guzel Davletshina[1], Maria Pobedintseva[1], Ksenia Krasheninnikova[11], Daniel W. Foerster[12], Anna S. Mukhacheva[13], Alexandra Mironova[14], Michail Sidorov[15], Wenhui Nie[16], Jinhuan Wang[16], Svetlana A. Romanenko[1], Anastasiya A. Proskuryakova[1], Malcolm Ferguson-Smith[17], Fengtang Yang[18], Nikolay Cherkasov[19], Elena Balanovskaya[20], M. Thomas P. Gilbert[21,22], Innokentiy M. Okhlopkov[15], Anna Zhuk[23,24], Alexander S. Graphodatsky[1], Roger Powell[25], Klaus-Peter Koepfli[26], Sergei Kliver[21]

[1] Laboratory of Diversity and Evolution of Genomes, Institute of Molecular and Cellular Biology SB RAS, 8/2 Acad. Lavrentiev ave., Novosibirsk, 630090, Russia. polina.perelman@gmail.com (https://orcid.org/0000-0002-0982-5100), serd@mcb.nsc.ru (https://orcid.org/0000-0002-0409-1371), bekl@mcb.nsc.ru (https://orcid.org/0000-0002-9122-4143), Davlet15628@gmail.com (https://orcid.org/0009-0000-0650-2675), pobedintseva12@gmail.com (https://orcid.org/0000-0001-8447-9626), rosa@mcb.nsc.ru (https://orcid.org/0000-0002-0951-5209), andrena@mcb.nsc.ru (https://orcid.org/0000-0003-3812-4853), graf@mcb.nsc.ru (https://orcid.org/0000-0002-8282-1085).

[2] Department of Natural Sciences, Novosibirsk State University, 1 Pirogova str., Novosibirsk, 630090, Russia. andrey.tomarovsky@gmail.com (https://orcid.org/0000-0002-6414-704X), a.totickov1@gmail.com (https://orcid.org/0000-0003-1236-631X).

[3] Youth Laboratory of Molecular Genetics, Yugra State University, 16 Ulitsa Chekhova, Khanty-Mansiysk, 628011, Russia. ressaure@gmail.com (https://orcid.org/0000-0002-5215-2001).

[4] Laboratory for Theriology, Zoological Institute RAS, 1 Universitetskaya emb., St. Petersburg, 199034, Russia. a.abramov@mail.ru (https://orcid.org/0000-0001-9709-4469).

[5] Division of Evolutionary Biology, Ludwig-Maximilians-Universität, 2, Großhaderner str, Planegg, 82152, Germany. yakupova@bio.lmu.de (https://orcid.org/0000-0003-1486-0864).

[6] Microevolution and Biodiversity, Max Planck Institute for Biological Intelligence, Eberhard-Gwinner-Straße, Seewiesen, 82319, Germany. aliya.yakupova@bi.mpg.de

[7] Centre for Haemato-Oncology, Barts Cancer Institute, Queen Mary University of London, London, UK. d.prokopov@qmul.ac.uk (https://orcid.org/0000-0001-8420-5203).

[8] QMUL Centre for Epigenetics, Queen Mary University of London, London, UK.

[9] Center for Evolutionary Hologenomics, The Globe Institute, The University of Copenhagen, Copenhagen, Denmark; mhssinding@gmail.com (https://orcid.org/0000-0003-1371-219X).

[10] Department of Biology, The University of Copenhagen, Copenhagen, Denmark.

[11] Independent researcher, Wellcome Trust Genome Campus, Hinxton, Saffron Walden CB10 1RQ, United Kingdom. krasheninnikova@gmail.com (https://orcid.org/0000-0002-0604-2047).

[12] Leibniz Institute for Zoo and Wildlife Research (IZW), Alfred Kowalke Straße 17, 10315 Berlin, Germany. DWGFoerster@gmail.com (https://orcid.org/0000-0002-6934-0404).

[13] Sikhote-Alin Biosphere Zapovednik, 44 Partizanskaya str., Ternei, 692150, Russia. siam83@mail.ru (https://orcid.org/0009-0008-0177-8873).

[14] Laboratoire de Physiologie Cellulaire and Végétale, Univ. Grenoble Alpes/CNRS/CEA/INRA/IRIG, Grenoble, France. aleksandra.s.mironova@gmail.com (https://orcid.org/0009-0000-3831-8151)

[15] Institute of Biological Problems of Cryolithozone SB RAS, 41 Lenina ave., Yakutsk, 677000, Russia. sidorov_michail86@mail.ru (https://orcid.org/0000-0003-0333-261X), imokhlopkov@yandex.ru (https://orcid.org/0000-0002-6227-5216).

[16] State Key Laboratory of Genetic Resources and Evolution, Kunming Institute of Zoology, Chinese Academy of Sciences, Kunming 650223, China, whnie@mail.kiz.ac.cn, wangjing315@163.com.

[17] Cambridge Resource Centre for Comparative Genomics, Department of Veterinary Medicine, University of Cambridge, Cambridge CB3 OES, UK. maf12@cam.ac.uk (https://orcid.org/0000-0001-9372-1381)

[18] School of Life Sciences and Medicine, Shandong University of Technology, Zibo, China. yangfengtang@sdut.edu.cn (https://orcid.org/0000-0002-3573-2354).

[19] Vavilov Institute of General Genetics, Moscow, Russia. x@utrail.org (https://orcid.org/0000-0003-1416-0200).

[20] Laboratory of human population genetics, Research Centre for Medical Genetics, Moscow 115522, Russia. balanovska@mail.ru (https://orcid.org/0000-0002-3882-8300).

[21] Center for Evolutionary Hologenomics, The Globe Institute, The University of Copenhagen, 5A, Oester Farimagsgade, Copenhagen, 1353, Denmark. tgilbert@sund.ku.dk (https://orcid.org/0000-0002-5805-7195), sergei.kliver@sund.ku.dk (https://orcid.org/0000-0002-2965-3617).

[22] University Museum, NTNU, Trondheim, Norway.

[23] Institute of Applied Computer Science, ITMO University, 197101 St. Petersburg, Russia. ania.zhuk@gmail.com (https://orcid.org/0000-0001-8683-9533).

[24] Laboratory of Amyloid Biology, St. Petersburg State University, 199034 St. Petersburg, Russia.

[25] North Carolina State University. rpowell@ncsu.edu (https://orcid.org/0000-0001-9419-4034).

[26] Smithsonian-Mason School of Conservation, 1500 Remount Road, Front Royal, VA 22630, USA. klauspeter.koepfli527@gmail.com (https://orcid.org/0000-0001-7281-0676).

* corresponding author

[x] equal contribution

# Table of Contents

**Supplementary Figures and Tables........................................................................................ 1**

Table of Contents........................................................................................................................ 4

Supplementary Figures (SF)........................................................................................................6

Supplementary Figure SF1. 23-mer frequency distributions for filtered genome data of putative sables (A), putative pine martens (B), and putative hybrids (C).................................. 6

Supplementary Figure SF2. Principal component analysis (PCA).............................................7

Supplementary Figure SF3. Model fitting of heterozygosity distributions................................ 8

Supplementary Figure SF4. Localization of STR markers and ancestry analysis for three STR datasets.........................................................................................................................................9

Supplementary figure SF5. Mitochondrial phylogenetic tree...................................................10

Supplementary Figure SF6. Distributions of mean heterozygosity (SNP only)........................11

Supplementary Figure SF7. Runs of homozygosity (RoH)......................................................12

Supplementary Figure SF8. Correlation between introgression levels and the “population explosion” peak height in hybrids........................................................................................ 13

Supplementary Figure SF9. Scoped demographic history reconstruction................................14

Supplementary Figure SF10. Weighted Fst between pure sables and pure pine martens..........15

Supplementary Figure SF11. Geographic distribution of samples with local ancestry analysis... 15

Supplementary Tables (ST)........................................................................................................ 16

Supplementary Table ST1. Sample information.......................................................................16

Supplementary Table ST2. Samples quality. Number of reads, k-mers coverage, genome sizes and downsampling fraction.........................................................................................................18

Supplementary Table ST3. Fossil based calibrations used to date divergence times between species.........................................................................................................................................20

Supplementary Table ST4. Global and local ancestry............................................................. 21

Supplementary Table ST5. Heterozygous SNP counts (autosomes only)................................ 22

Supplementary Table ST6. RoH content................................................................................. 23

Supplementary Table ST7. Candidate regions, associated with differences between the sable and the pine marten.............................................................................................................24

Supplementary Table ST8. Comparison of Fst values for the p-arm of chromosome 11 with estimates on the p-arms of other chromosomes.........................................................................25

Supplementary Table ST9. Estimated divergence times between Martes species (M. zibellina, M. martes and M. foina) across phylogenetic studies................................................................25

References.......................................................................................................................... 27

# Supplementary Figures (SF)

**Supplementary Figure SF1.** 23-mer frequency distributions for filtered genome data of putative sables (A), putative pine martens (B), and putative hybrids (C).

The upper plots are shown in logarithmic scale on both axes, while the lower plots are presented in linear scale. The estimated genome size for each sample is indicated in the legend.

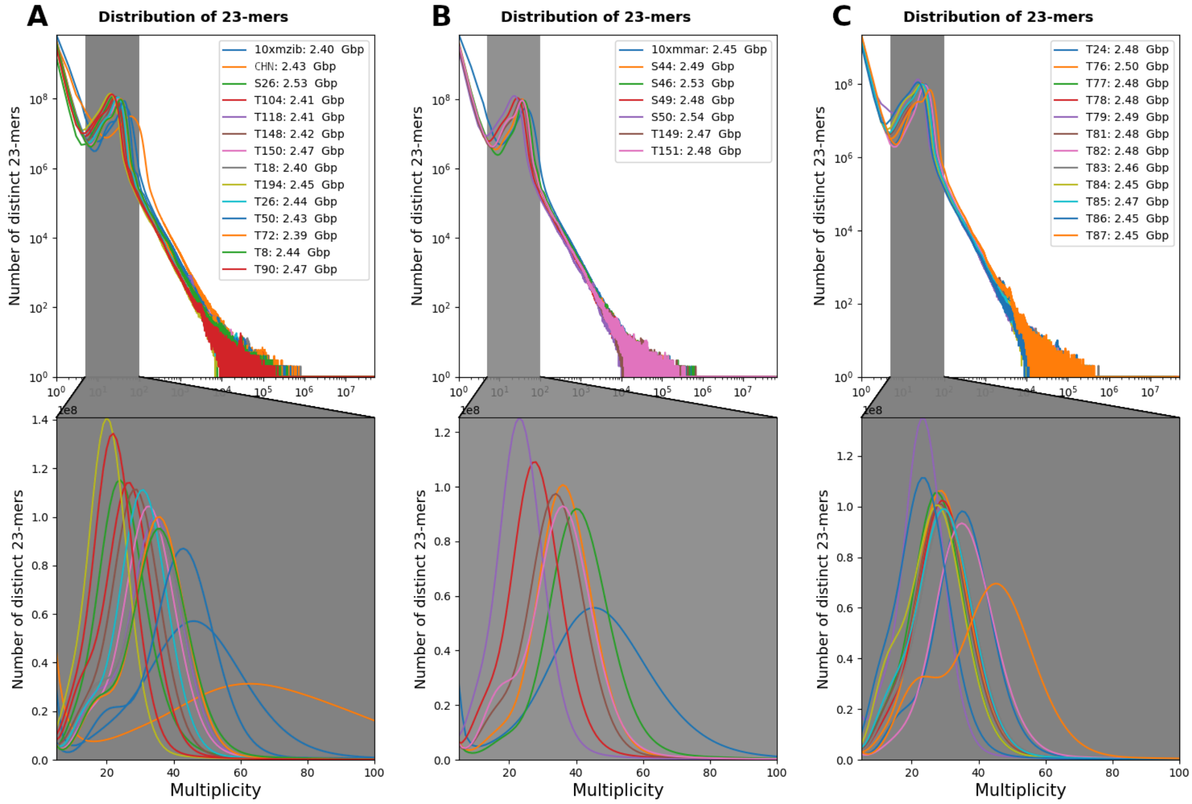

**Supplementary Figure SF2.** Principal component analysis (PCA).

PCA plot based on 1,719,592 SNPs from the autosomes of the sable assembly. The first and second principal components are shown on the X and Y axes, respectively, with the percentage of explained variance indicated in parentheses. Each point represents an individual sample, labeled accordingly.

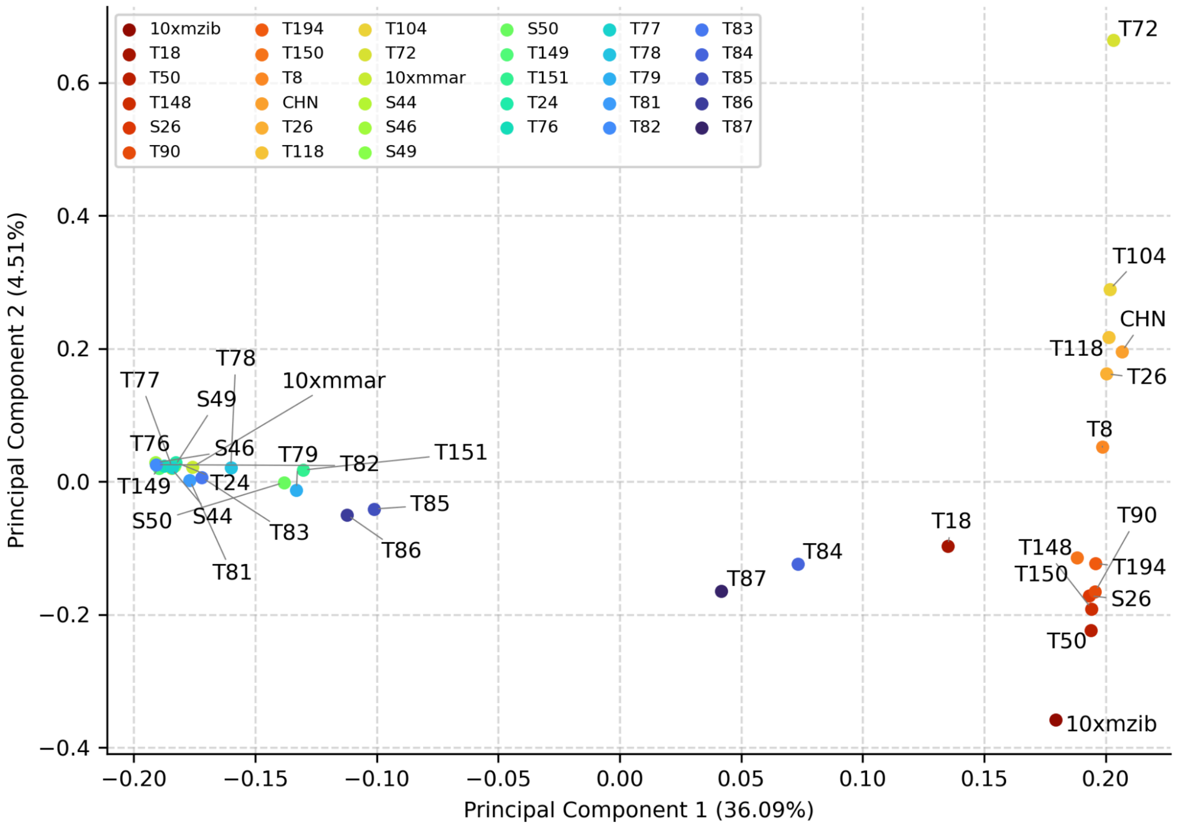

**Supplementary Figure SF3.** Model fitting of heterozygosity distributions.

Mean heterozygosity was calculated in 1 Mbp sliding windows with a 100 kbp step (autosomes only). The first bin, corresponding to runs of homozygosity (RoH), was removed for all samples. The blue step plots represent the original empirical distributions to be fitted. The green curves correspond to the combined fitted distributions, and the red curves denote individual fitted components. Red triangles indicate the mean values of the individual components. The Y-axis shows probability density, and the X-axis represents the heterozygous SNP/kbp. Corresponding mean values for each distribution in Supplementary File 5.

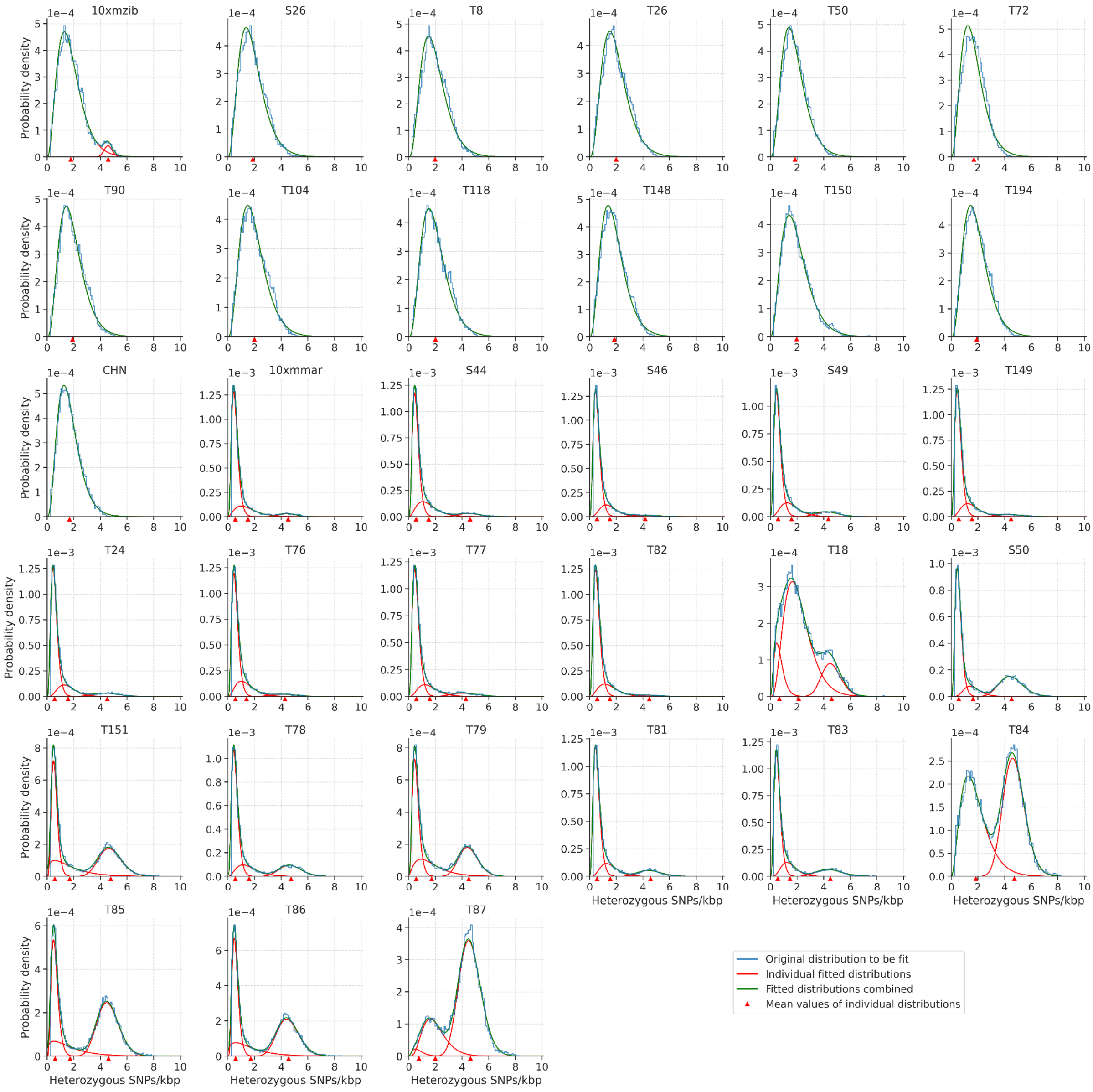

**Supplementary Figure SF4.** Localization of STR markers and ancestry analysis for three STR datasets.

Chromosomal localization of STR markers from different datasets and corresponding ancestry analysis. A – combined set of all mapped STRs loci; B – mapped markers from (Rozhnov et al. 2013); C – mapped markers from (Kashtanov et al. 2022); D – ancestry analysis for each set of STR loci. Samples are labeled along the X-axis while the Y-axis represents the mean probability of ancestry.

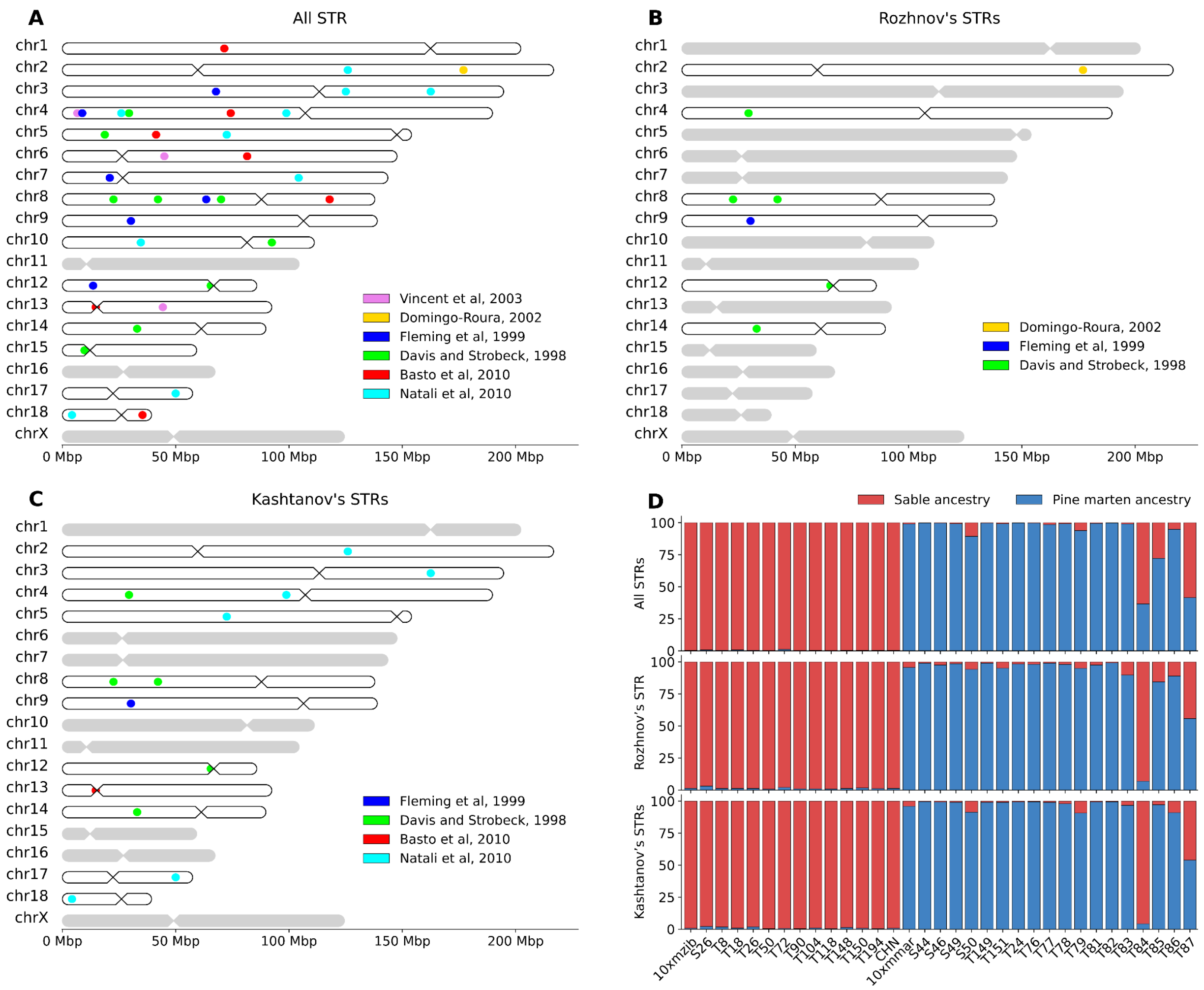

**Supplementary figure SF5.** Mitochondrial phylogenetic tree.

Nodes with more than 90 bootstrap support are marked in green, and nodes between 70 and 90 are marked in yellow. Nodes with support values less than 70 were deleted. Three main clade groups (A, B, and C) were identified, which included subclades (A1-5, B1-4 and C1-4). The studied samples are color-coded: sables with mtDNA from sable are shown in purple; pine martens with mtDNA from pine marten are shown in blue; pine martens with mtDNA from sable are shown in red; hybrids with mtDNA from sable are shown in green; hybrids with mtDNA from pine marten are shown in orange. *M. foina* (NC_020643.1) was used as an outgroup (not shown).

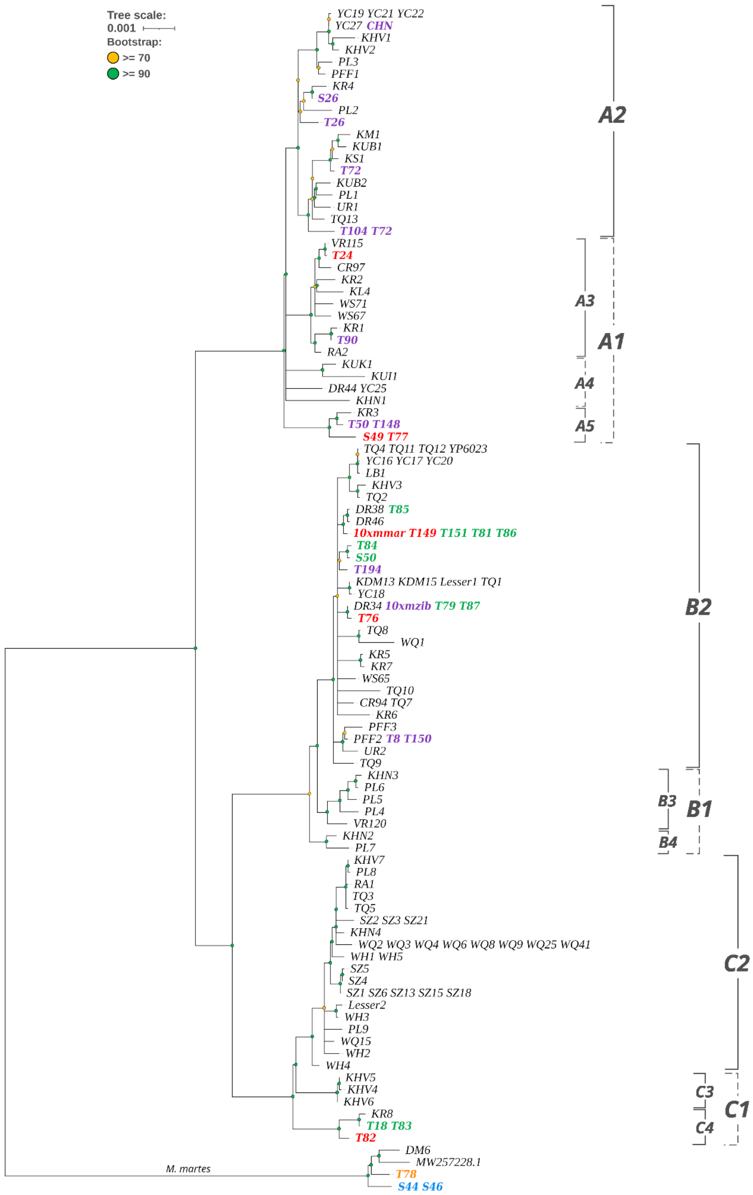

**Supplementary Figure SF6.** Distributions of mean heterozygosity (SNP only).

Mean heterozygosity was calculated in 1 Mbp sliding windows with a 100 kbp step (autosomes only). The distributions show the number of windows (in thousands, $10^3$) on the Y-axis and heterozygous SNP/kbp on the X-axis.

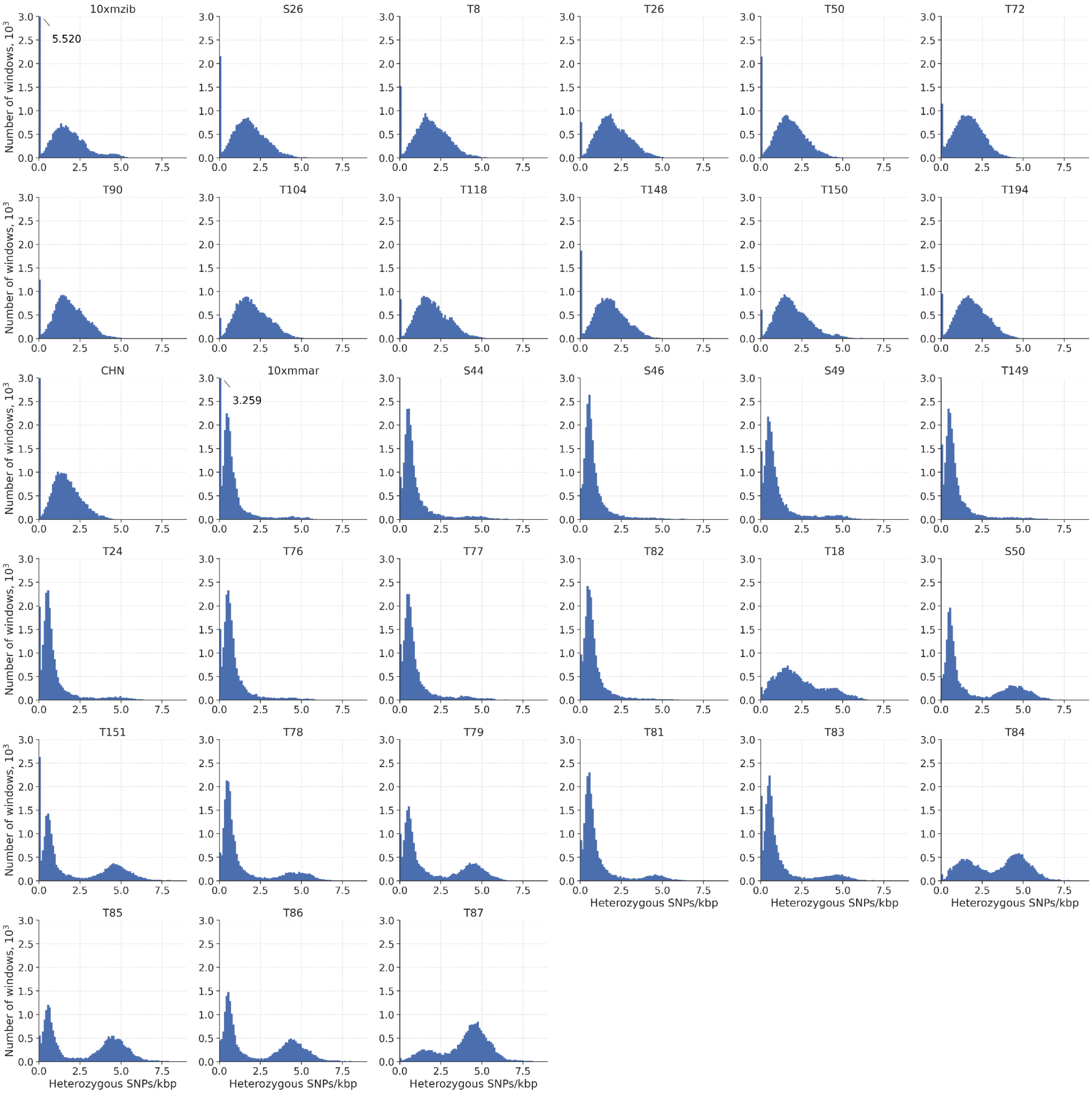

**Supplementary Figure SF7.** Runs of homozygosity (RoH).

A – cumulative distribution of RoH length in the pure martens, pure sables and hybrids (from left to right). B – Cumulative fraction of the genome covered by RoHs of different size categories: Short RoH (<1 Mbp), Long RoH (≥1 Mbp), and Ultra Long RoH (≥10 Mbp). Non-RoH indicates the fraction of the genome not covered by any detected RoH segments.

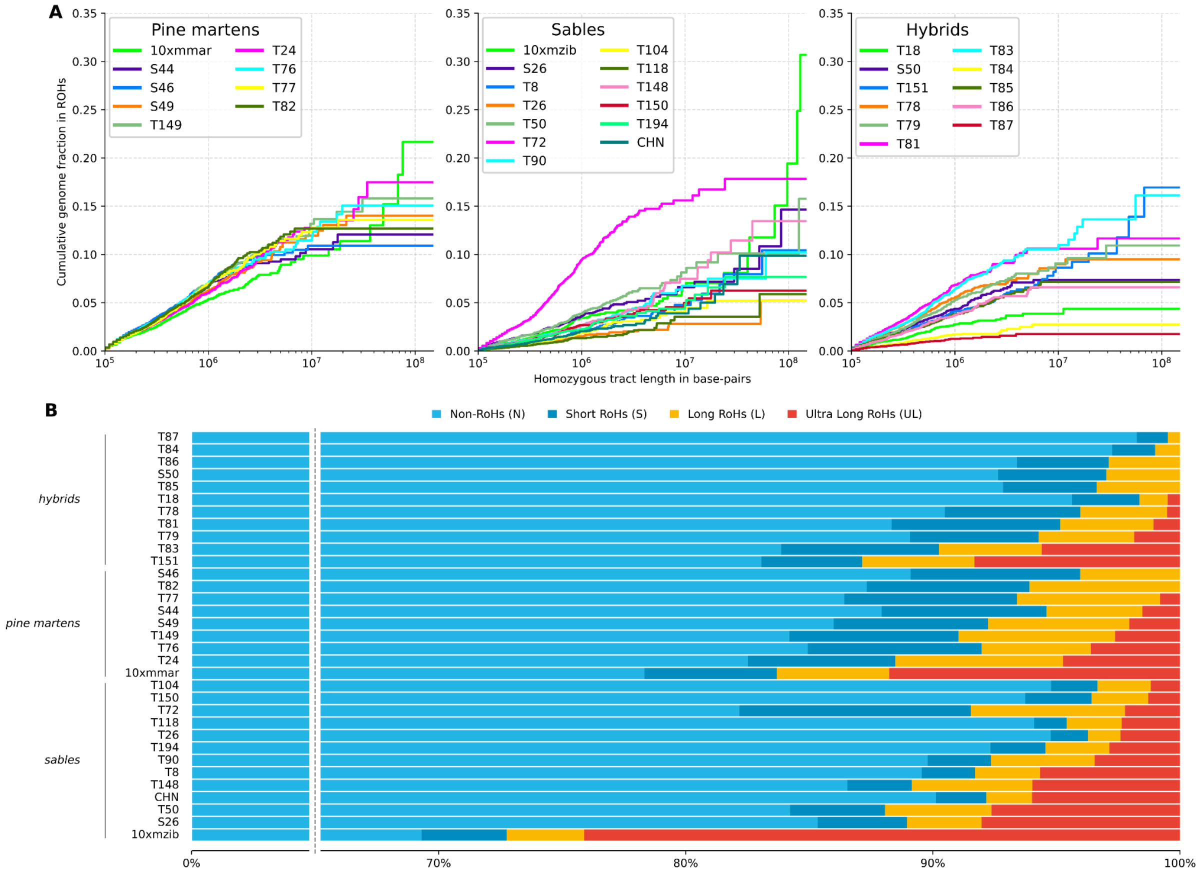

**Supplementary Figure SF8.** Correlation between introgression levels and the "population explosion" peak height in hybrids.

Scatter plot showing the relationship between introgression (Y-axis, %) and effective population size (X-axis, $10^4$). The top-right corner displays the coefficient of determination ($R^2$), while the bottom-right corner shows the correlation coefficients and p-values for Kendall's tau, Pearson's r, and Spearman's rho.

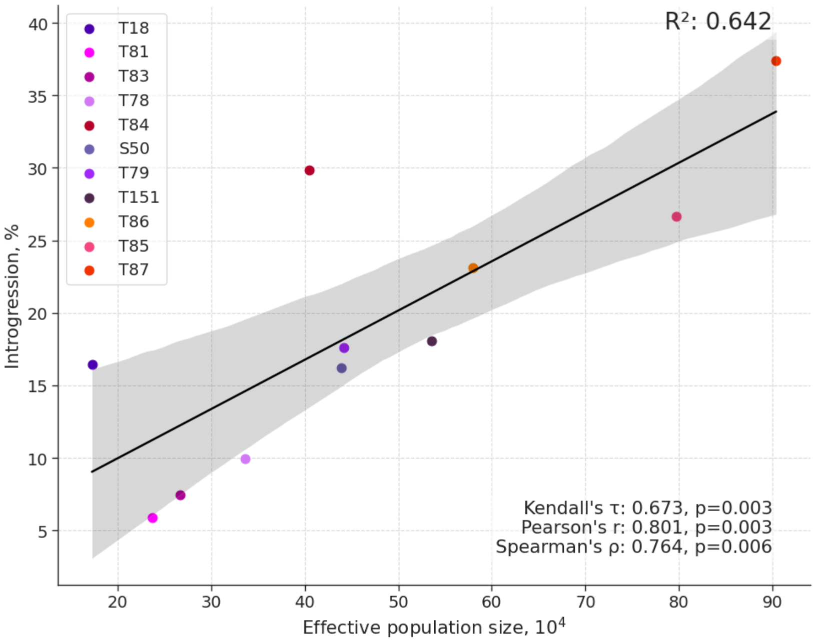

**Supplementary Figure SF9.** Scoped demographic history reconstruction.

Detailed view of the demographic history of pure sables (green curves), pure pine martens (blue curves), and hybrids (red curves). The Y-axis represents effective population size ($\times 10^4$), and the X-axis represents time in years ago. Mutation rate ($\mu$) = $4.64 \times 10^{-9}$ and generation time (g) = 5 years. Highlighted Marine Isotope Stages (MIS, cold stages only) are 6 (130–191 kya), 8 (243–300 kya), 10 (337–374 kya), and 12 (424–478 kya). MPT – Mid-Pleistocene Transition (0.7–1.25 Mya).

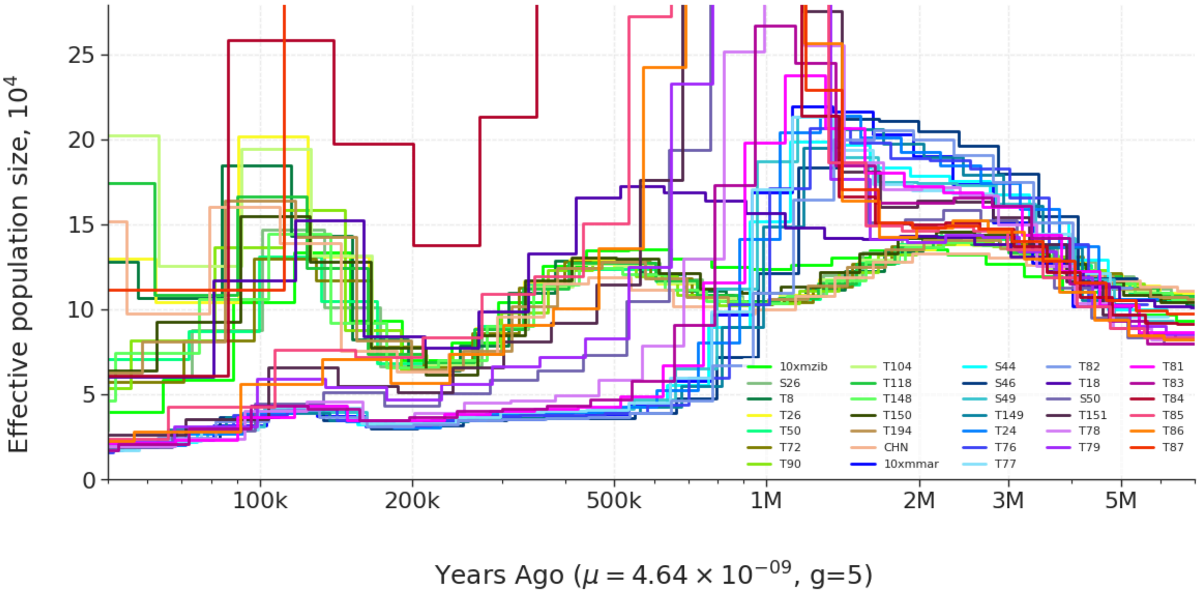

**Supplementary Figure SF10.** Weighted $F_{st}$ between pure sables and pure pine martens.

A – Heatmap of weighted $F_{st}$ values (chrX excluded, shown in light grey). The color scale on the right represents $F_{st}$ values from dark blue (extremely low, 0) to brown (very high, >1). Fst was calculated in 1 Mbp sliding windows with a 100 kbp step; B – Cumulative distribution of weighted Fst values, with the Y-axis showing the number of 1 Mbp windows and the X-axis representing $F_{st}$ thresholds in increments of 0.05; C – Boxplots showing weighted Fst values for the p-arms of chr11, 12, 13, 15, and 18, with the Y-axis representing weighted $F_{st}$ and the X-axis showing the corresponding chromosomes.

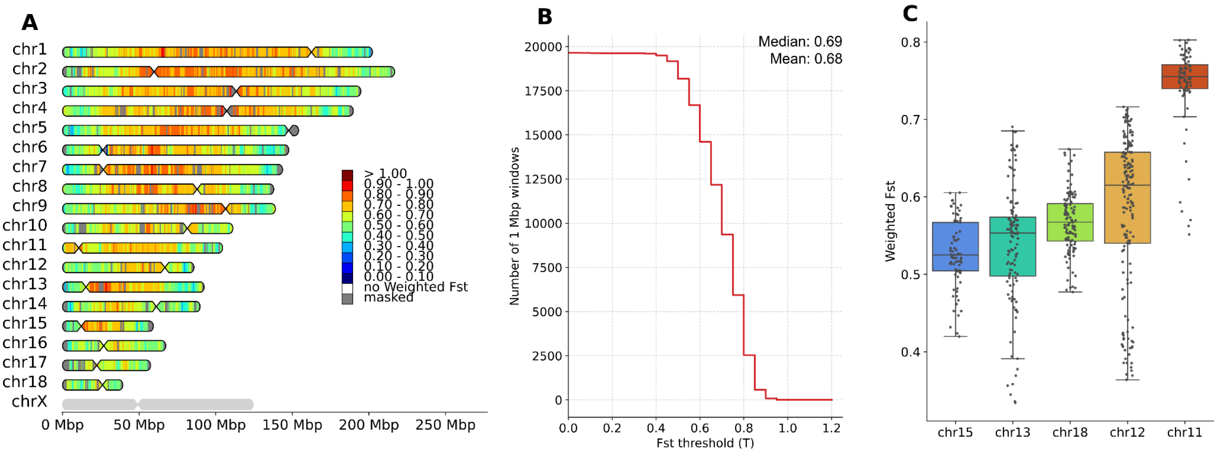


**Supplementary Figure SF11.** Geographic distribution of samples with local ancestry analysis.

Map showing the locations of all samples. The range of pine martens is shown in yellow, the range of sables in red, and the sympatric zone in maroon. For each sample, a bar plot indicates the proportion of ancestry derived from sables and pine martens, based on the results of the local ancestry analysis.

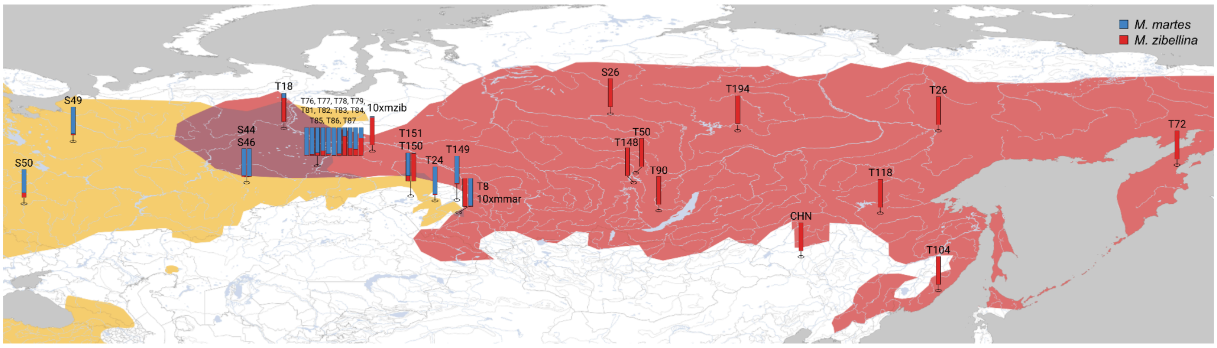

# Supplementary Tables (ST)

**Supplementary Table ST1.** Sample information.

| Species * | Data type ** | ID | SRA / BioProject ID | Origin | Point on map | Sex ***** |
|---|---|---|---|---|---|---|
| *M. zibellina* | 10X | 10xmzib*** | SRR22412799 | Khanty-Mansi Autonomous Okrug – Yugra, Ugut | 17 | F |
| *M. zibellina* | PE | S26 | SRR28749757 | Krasnoyarsk Krai, Evenkia Autonomous Okrug, Baikit | 5 | F |
| *M. zibellina* | PE | T8 | SRR28749756 | Altai Krai complex "Magistralny" | 15 | M |
| *M. zibellina* | PE | T18 | SRR28749745 | Khanty-Mansi Autonomous Okrug – Yugra, Peregrebnoe | 14 | M |
| *M. zibellina* | PE | T26 | SRR28749734 | Sakha – Yakutia, Okhotskiy Perevoz | 8 | F |
| *M. zibellina* | PE | T50 | SRR28749733 | Irkutsk Oblast, Nevon | 13 | M |
| *M. zibellina* | PE | T72 | SRR28749732 | Kamchatskiy Krai, Ivashka | 6 | M |
| *M. zibellina* | PE | T90 | SRR28749731 | Irkutsk Oblast, Naran | 12 | F |
| *M. zibellina* | PE | T104 | SRR28749730 | Primorskiy Krai, Terney | 16 | M |
| *M. zibellina* | PE | T118 | SRR28749729 | Amur Oblast, Dugda | 4 | M |
| *M. zibellina* | PE | T148 | SRR28749728 | Irkutsk region, Ust'-Ilimsk | 18 | F |
| *M. zibellina* | PE | T150 | SRR28749755 | Novosibirsk Oblast, Kuibyshev | 9 | F |
| *M. zibellina* | PE | T194 | SRR28749754 | Sakha – Yakutia, Mirnyi | 11 | F |
| *M. zibellina* | PE | CHN **** | PRJNA495455 | Greater Khingan Mountains, China | 21 | F |
| *M. martes* | 10X | 10xmmar *** | SRR22412800 | Altai Krai, Barnaul, "Lesnaya skazka" zoo | 1 | F |
| *M. martes* | PE | S44 | SRR28749753 | Sverdlovsk Oblast, Verkhnyaya Pyshma | 20 | F |
| *M. martes* | PE | S46 | SRR28749752 | Sverdlovsk Oblast, Verkhnyaya Pyshma | 20 | F |
| *M. martes* | PE | S49 | SRR28749751 | Arkhangelsk Oblast, Velsk | 19 | M |

| Species * | Data type ** | ID | SRA / BioProject ID | Origin | Point on map | Sex ***** |
|---|---|---|---|---|---|---|
| *M. martes* | PE | S50 | SRR28749750 | Kaluga Oblast, Kaluga | 7 | F |
| *M. martes* | PE | T149 | SRR28749749 | Novosibirsk Oblast, Baryshevo | 2 | M |
| *M. martes* | PE | T151 | SRR28749748 | Novosibirsk Oblast, Kuibyshev | 9 | M |
| *Putative hybrid* | PE | T24 | SRR28749747 | Novosibirsk Oblast, Chulym | 3 | M |
| *Putative hybrid* | PE | T76 | SRR28749746 | Tyumen Oblast, Malyi Narys | 10 | F |
| *Putative hybrid* | PE | T77 | SRR28749744 | Tyumen Oblast, Malyi Narys | 10 | M |
| *Putative hybrid* | PE | T78 | SRR28749743 | Tyumen Oblast, Malyi Narys | 10 | F |
| *Putative hybrid* | PE | T79 | SRR28749742 | Tyumen Oblast, Malyi Narys | 10 | F |
| *Putative hybrid* | PE | T81 | SRR28749741 | Tyumen Oblast, Malyi Narys | 10 | M |
| *Putative hybrid* | PE | T82 | SRR28749740 | Tyumen Oblast, Malyi Narys | 10 | F |
| *Putative hybrid* | PE | T83 | SRR28749739 | Tyumen Oblast, Malyi Narys | 10 | M |
| *Putative hybrid* | PE | T84 | SRR28749738 | Tyumen Oblast, Malyi Narys | 10 | M |
| *Putative hybrid* | PE | T85 | SRR28749737 | Tyumen Oblast, Malyi Narys | 10 | F |
| *Putative hybrid* | PE | T86 | SRR28749736 | Tyumen Oblast, Malyi Narys | 10 | M |
| *Putative hybrid* | PE | T87 | SRR28749735 | Tyumen Oblast, Malyi Narys | 10 | M |

* The initial classification of the samples, based on simple morphological traits: tail length, fur length and quality and throat patch size.

** Data type: “PE” – paired end Illumina reads, “10X” – 10x Genomics linked reads;

*** Reference individuals, previously published in (Tomarovsky et al. 2025);

**** Previously published sample (Liu et al. 2020);

***** Sex: M – male, F – female;

**Supplementary Table ST2.** Samples quality. Number of reads, k-mers coverage, genome sizes and downsampling fraction.

| Sample | Read length | Number of raw reads, mln | Number of filtered reads, mln | Saved reads, % | Kmer multiplicity at first maximum | Estimated haplome coverage (genomescope2) | Coverage (genomescope2) | Genome size (genomescope2), Gbp | Downsampling fraction * |
|---|---|---|---|---|---|---|---|---|---|
| 10xmzib | 128+150 | 619.13 | 566.65 | 91.52 | 46 | 24.85 | 49.7 | 2.4 | 0.44 |
| S26 | 150+150 | 269.04 | 261.32 | 97.13 | 24 | 12.66 | 25.32 | 2.53 | 0.87 |
| T8 | 150+150 | 366.9 | 357.24 | 97.37 | 36 | 18.44 | 36.88 | 2.44 | 0.6 |
| T26 | 150+150 | 319.08 | 309.51 | 97 | 31 | 15.91 | 31.82 | 2.44 | 0.69 |
| T50 | 150+150 | 437.93 | 423.14 | 96.62 | 43 | 22.01 | 44.02 | 2.43 | 0.5 |
| T72 | 150+150 | 361.13 | 349.85 | 96.88 | 36 | 18.34 | 36.68 | 2.39 | 0.6 |
| T90 | 150+150 | 232.63 | 225.76 | 97.05 | 22 | 11.49 | 22.98 | 2.47 | 0.96 |
| T104 | 150+150 | 275.03 | 266.11 | 96.76 | 27 | 13.87 | 27.74 | 2.41 | 0.79 |
| T118 | 150+150 | 352.81 | 342.09 | 96.96 | 35 | 17.82 | 35.64 | 2.41 | 0.62 |
| T148 | 150+150 | 294.84 | 286.42 | 97.15 | 29 | 14.84 | 29.68 | 2.42 | 0.74 |
| T150 | 150+150 | 341 | 331.36 | 97.17 | 33 | 16.88 | 33.76 | 2.47 | 0.65 |
| T194 | 150+150 | 214.47 | 207.9 | 96.93 | 20 | 10.62 | 21.24 | 2.45 | NA |
| CHN | 120+120 | 977.18 | 955.63 | 97.79 | 63 | 38.39 | 76.78 | 2.43 | 0.29 |
| 10xmmar | 128+150 | 633.01 | 578.26 | 91.35 | 45 | 24.81 | 49.62 | 2.45 | 0.44 |
| S44 | 150+150 | 380.48 | 369.5 | 97.11 | 36 | 18.66 | 37.32 | 2.49 | 0.59 |
| S46 | 150+150 | 427.28 | 417.27 | 97.66 | 40 | 20.8 | 41.6 | 2.53 | 0.53 |
| S49 | 150+150 | 291.26 | 284.51 | 97.68 | 28 | 14.46 | 28.92 | 2.48 | 0.76 |
| T149 | 150+150 | 351.28 | 343.66 | 97.83 | 34 | 17.59 | 35.18 | 2.47 | 0.63 |
| T24 | 150+150 | 376.9 | 365.39 | 96.95 | 35 | 18.24 | 36.48 | 2.48 | 0.6 |
| T76 | 150+150 | 307.97 | 302.5 | 98.22 | 29 | 15.07 | 30.14 | 2.5 | 0.73 |

| Sample | Read length | Number of raw reads, mln | Number of filtered reads, mln | Saved reads, % | Kmer multiplicity at first maximum | Estimated haplome coverage (genomescope2) | Coverage (genomescope2) | Genome size (genomescope2), Gbp | Downsampling fraction * |
|---|---|---|---|---|---|---|---|---|---|
| T77 | 150+150 | 295.04 | 289.54 | 98.14 | 28 | 14.53 | 29.06 | 2.48 | 0.76 |
| T82 | 150+150 | 370.46 | 363.71 | 98.18 | 35 | 18.29 | 36.58 | 2.48 | 0.6 |
| T18 | 150+150 | 356.65 | 345.64 | 96.91 | 35 | 18.07 | 36.14 | 2.4 | 0.61 |
| S50 | 150+150 | 250.61 | 244.71 | 97.65 | 23 | 12.18 | 24.36 | 2.54 | 0.9 |
| T151 | 150+150 | 377.11 | 367.49 | 97.45 | 36 | 18.68 | 37.36 | 2.48 | 0.59 |
| T78 | 150+150 | 312.95 | 306.79 | 98.03 | 29 | 15.4 | 30.8 | 2.48 | 0.71 |
| T79 | 150+150 | 253.9 | 245.87 | 96.84 | 23 | 12.14 | 24.28 | 2.49 | 0.91 |
| T81 | 150+150 | 305.17 | 299.3 | 98.08 | 28 | 15.06 | 30.12 | 2.48 | 0.73 |
| T83 | 150+150 | 317.82 | 311.74 | 98.08 | 30 | 15.81 | 31.62 | 2.46 | 0.7 |
| T84 | 150+150 | 292.12 | 287.13 | 98.29 | 28 | 14.63 | 29.26 | 2.45 | 0.75 |
| T85 | 150+150 | 315.36 | 309.73 | 98.21 | 30 | 15.63 | 31.26 | 2.47 | 0.7 |
| T86 | 150+150 | 251 | 246.61 | 98.25 | 23 | 12.58 | 25.16 | 2.45 | 0.87 |
| T87 | 150+150 | 471.25 | 462.92 | 98.23 | 45 | 23.59 | 47.18 | 2.45 | 0.47 |

* NA – not applicable, i.e., downsampling was unnecessary; all other values indicate the fraction of data retained after downsampling.

**Supplementary Table ST3.** Fossil based calibrations used to date divergence times between species.

| Node | Node minimum age | | | | | | | Node maximum age | | | | | | |
|---|---|---|---|---|---|---|---|---|---|---|---|---|---|---|
| | **Age (Ma)** | **Evidence** | **Reference** | **Oldest fossil taxon** | **min age** | **max age** | **description** | **Age (Ma)** | **Evidence** | **Reference** | **Oldest fossil taxon** | **min age** | **max age** | **description** |
| Mustelidae - Ailuridae split | **30.9** | fossil | (Wang et al. 2005; Law et al. 2018) | *Mustelictis olivieri* | 30.9 | 32.8 | oldest Mustelinae or sister species | **32.1** | fossil | (Wang et al. 2005; Law et al. 2018) | *Amphicticeps dorog* | 30.4 | 32.1 | stem Arctoidea |
| Meles - Neogale + Lutrinae split | **13.6** | fossil | (Salesa et al. 2013; Law et al. 2018) | *Taxodon sansaniensis* | 13.6 | 15 | oldest Melinae | **-** | NA | NA | NA | NA | NA | NA |
| *Eira - Martes + Gulo* | **7.05** | fossil | (Samuels and Cavin 2013; Li et al. 2014) | *Pekania occulta* | 7.05 | 7.3 | Oldest *Pekania* fossil | **20.44** | geostratigraphy | (Li et al. 2014) | NA | NA | NA | The upper bound is the age of the Aquitanian–Burdigalian boundary within the Early Miocene |
| *Martes - Gulo* | **3.3** | fossil | (Stach 1959; Li et al. 2014; Marciszak et al. 2024) | *Martes wenzensis* | 3.3 | 4 | Oldest proven *Martes* fossil | **20.44** | geostratigraphy | (Li et al. 2014) | NA | NA | NA | The upper bound is the age of the Aquitanian–Burdigalian boundary within the Early Miocene |
| *M. foina - M. zibelina + M. martes* | **1.75** | fossil | (Wolsan 1990; Li et al. 2014) | *Martes vetus* | 1.75 | 2 | Oldest sable/pine-marten like species | **-** | NA | NA | NA | NA | NA | NA |

**Supplementary Table ST4.** Global and local ancestry.

Cells are color-coded as follows: green and dark blue – pure sables and pure pine martens, respectively; yellow and blue – atypical sables and atypical martens, respectively; orange and light blue – backcross-like hybrids, respectively; and dark red – F1-like hybrids.

| Sample | Global ancestry (% of sable) | Local ancestry (% of sable) | Difference |
|---|---|---|---|
| 10xmzib | 100 | 96.85 | 3.15 |
| S26 | 100 | 99.79 | 0.21 |
| T8 | 100 | 99.9 | 0.1 |
| T26 | 100 | 99.87 | 0.13 |
| T50 | 100 | 99.92 | 0.08 |
| T72 | 100 | 99.86 | 0.14 |
| T90 | 100 | 99.87 | 0.13 |
| T104 | 100 | 99.77 | 0.23 |
| T118 | 100 | 99.85 | 0.15 |
| T148 | 100 | 99.91 | 0.09 |
| T150 | 100 | 98.38 | 1.62 |
| T194 | 100 | 99.85 | 0.15 |
| CHN | 100 | 99.9 | 0.1 |
| 10xmmar | 0 | 3.02 | 3.02 |
| S44 | 0 | 3.57 | 3.57 |
| S46 | 0 | 1.69 | 1.69 |
| S49 | 0 | 4.17 | 4.17 |
| T149 | 0 | 2.52 | 2.52 |
| T24 | 0 | 3.55 | 3.55 |
| T76 | 0 | 2.51 | 2.51 |
| T77 | 0 | 3.76 | 3.76 |
| T82 | 0 | 2.02 | 2.02 |
| T18 | 84.5 | 83.55 | 0.95 |
| S50 | 12.6 | 16.21 | 3.61 |
| T151 | 14 | 18.09 | 4.09 |
| T78 | 6 | 9.96 | 3.96 |
| T79 | 12.9 | 17.6 | 4.7 |
| T81 | 0 | 5.92 | 5.92 |
| T83 | 0 | 7.48 | 7.48 |

| Sample | Global ancestry (% of sable) | Local ancestry (% of sable) | Difference |
|---|---|---|---|
| T84 | 67.8 | 70.14 | 2.34 |
| T85 | 22.5 | 26.64 | 4.14 |
| T86 | 19.6 | 23.1 | 3.5 |
| T87 | 59.6 | 62.62 | 3.02 |

**Supplementary Table ST5.** Heterozygous SNP counts (autosomes only).

Mean and median heterozygosity densities (hetSNPs/kbp) were calculated in 1 Mbp windows with 100 kbp step.

| Sample | Number of hetSNPs*, mln | Density, hetSNPs/kbp | |
|---|---|---|---|
| | | Mean | Median |
| 10xmzib | 3.14 | 1.39 | 1.32 |
| S26 | 3.87 | 1.71 | 1.67 |
| T8 | 4.23 | 1.87 | 1.82 |
| T26 | 4.45 | 1.97 | 1.87 |
| T50 | 3.80 | 1.69 | 1.66 |
| T72 | 3.66 | 1.63 | 1.61 |
| T90 | 4.10 | 1.83 | 1.74 |
| T104 | 4.48 | 1.99 | 1.88 |
| T118 | 4.43 | 1.96 | 1.86 |
| T148 | 3.89 | 1.73 | 1.69 |
| T150 | 4.44 | 1.96 | 1.79 |
| T194 | 4.24 | 1.88 | 1.81 |
| CHN | 3.63 | 1.61 | 1.54 |
| 10xmmar | 1.86 | 0.8 | 0.55 |
| S44 | 2.28 | 1.01 | 0.65 |
| S46 | 2.00 | 0.87 | 0.62 |
| S49 | 2.37 | 1.05 | 0.65 |
| T149 | 2.04 | 0.9 | 0.62 |
| T24 | 2.20 | 0.97 | 0.61 |
| T76 | 2.10 | 0.91 | 0.63 |
| T77 | 2.31 | 0.99 | 0.64 |
| T82 | 1.95 | 0.86 | 0.63 |
| T18 | 5.29 | 2.36 | 2.05 |

| Sample | Number of hetSNPs*, mln | Density, hetSNPs/kbp | |
|---|---|---|---|
| | | Mean | Median |
| S50 | 4.46 | 1.96 | 0.86 |
| T151 | 4.75 | 2.08 | 0.87 |
| T78 | 3.43 | 1.52 | 0.71 |
| T79 | 4.50 | 2.01 | 0.98 |
| T81 | 2.67 | 1.16 | 0.66 |
| T83 | 2.79 | 1.22 | 0.65 |
| T84 | 7.52 | 3.36 | 3.7 |
| T85 | 6.09 | 2.72 | 2.97 |
| T86 | 5.51 | 2.45 | 1.45 |
| T87 | 8.88 | 3.97 | 4.3 |

* heterozygous SNPs

**Supplementary Table ST6.** RoH content.

| Sample | Number of RoH | Total length, Mbp | % of genome | % of genome * | | |
|---|---|---|---|---|---|---|
| | | | | Short | Long | Ultra Long |
| 10xmzib | 305 | 685.19 | 30.69 | 3.45 | 3.13 | 24.1 |
| S26 | 363 | 327.29 | 14.66 | 3.61 | 3.02 | 8.03 |
| T8 | 202 | 233.1 | 10.44 | 2.15 | 2.62 | 5.67 |
| T26 | 147 | 116.74 | 5.23 | 1.51 | 1.31 | 2.41 |
| T50 | 373 | 352.26 | 15.78 | 3.84 | 4.3 | 7.63 |
| T72 | 781 | 398.08 | 17.83 | 9.38 | 6.23 | 2.23 |
| T90 | 270 | 228.06 | 10.21 | 2.57 | 4.19 | 3.45 |
| T104 | 166 | 116.5 | 5.22 | 1.89 | 2.15 | 1.18 |
| T118 | 130 | 131.66 | 5.9 | 1.32 | 2.23 | 2.35 |
| T148 | 269 | 300.45 | 13.46 | 2.6 | 4.88 | 5.97 |
| T150 | 240 | 139.7 | 6.26 | 2.67 | 2.3 | 1.29 |
| T194 | 233 | 171.47 | 7.68 | 2.24 | 2.58 | 2.85 |
| CHN | 194 | 220.48 | 9.87 | 2.04 | 1.85 | 5.98 |
| 10xmmar | 535 | 483.75 | 21.66 | 5.35 | 4.54 | 11.77 |
| S44 | 643 | 269.36 | 12.06 | 6.66 | 3.89 | 1.52 |
| S46 | 679 | 243.34 | 10.9 | 6.86 | 4.04 | 0 |
| S49 | 619 | 312.86 | 14.01 | 6.25 | 5.71 | 2.05 |
| T149 | 660 | 353.12 | 15.81 | 6.85 | 6.33 | 2.63 |
| T24 | 612 | 390.56 | 17.49 | 5.98 | 6.78 | 4.73 |

| Sample | Number of RoH | Total length, Mbp | % of genome | % of genome * | | |
|---|---|---|---|---|---|---|
| | | | | Short | Long | Ultra Long |
| T76 | 666 | 336.25 | 15.06 | 7.03 | 4.42 | 3.61 |
| T77 | 640 | 303.38 | 13.59 | 6.99 | 5.79 | 0.81 |
| T82 | 670 | 283 | 12.67 | 6.59 | 6.08 | 0 |
| T18 | 268 | 97.63 | 4.37 | 2.73 | 1.14 | 0.5 |
| S50 | 445 | 164.18 | 7.35 | 4.37 | 2.98 | 0 |
| T151 | 406 | 378.36 | 16.94 | 4.09 | 4.53 | 8.32 |
| T78 | 535 | 212.31 | 9.51 | 5.48 | 3.5 | 0.52 |
| T79 | 477 | 244.06 | 10.93 | 5.21 | 3.87 | 1.85 |
| T81 | 643 | 260.52 | 11.67 | 6.82 | 3.77 | 1.07 |
| T83 | 597 | 360.3 | 16.14 | 6.38 | 4.16 | 5.6 |
| T84 | 166 | 61.14 | 2.74 | 1.73 | 1.01 | 0 |
| T85 | 385 | 159.89 | 7.16 | 3.79 | 3.37 | 0 |
| T86 | 371 | 147.26 | 6.6 | 3.72 | 2.88 | 0 |
| T87 | 130 | 39.01 | 1.75 | 1.25 | 0.5 | 0 |

* Short RoH (< 1 Mbp), Long RoH (>= 1 Mbp < 10 Mbp) and Ultra Long RoH (>= 10 Mbp).

**Supplementary Table ST7.** Candidate regions, associated with differences between the sable and the pine marten.

**Bold** markers nested loci with both high Tajima's D (TJD1) and high FST (FST1). *Italic* highlights a loci with GO term enrichment.

| Region ID | Description | Chr | Start | End | Length (Mbp) | Number of genes | Mean $F_{st}$ | Mean Tajima's D |
|---|---|---|---|---|---|---|---|---|
| INV1 | Inversion | chr11 | 2079555 | 13548101 | 11.5 | 31 | 0.74 | 0.44 |
| **TJD1** | **High Tajima's D** | **chr1** | **65300000** | **66800000** | **1.5** | **23** | **0.93** | **2.08** |
| **FST1** | **High $F_{st}$** | **chr1** | **64500000** | **67200000** | **2.7** | **40** | **0.92** | **1.94** |
| FST2 | High $F_{st}$ | chr2 | 58200000 | 59300000 | 1.1 | 9 | 0.90 | 1.61 |
| FST3 | High $F_{st}$ | chr2 | 60500000 | 61800000 | 1.3 | 6 | 0.90 | 1.66 |
| *FST4* | *High $F_{st}$* | *chr2* | *111100000* | *112200000* | *1.1* | *27* | *0.90* | *1.62* |
| FST5 | High $F_{st}$ | chr3 | 109200000 | 111100000 | 1.9 | 14 | 0.92 | 1.50 |
| *FST6* | *High $F_{st}$* | *chr3* | *114500000* | *115700000* | *1.2* | *14* | *0.90* | *1.19* |
| FST7 | High $F_{st}$ | chr4 | 103400000 | 105400000 | 2 | 64 | 0.91 | 1.71 |
| FST8 | High $F_{st}$ | chr6 | 58100000 | 59800000 | 1.7 | 43 | 0.91 | 1.78 |
| FST9 | High $F_{st}$ | chr7 | 58100000 | 59700000 | 1.6 | 42 | 0.91 | 1.58 |

| Region ID | Description | Chr | Start | End | Length (Mbp) | Number of genes | Mean $F_{st}$ | Mean Tajima's D |
|---|---|---|---|---|---|---|---|---|
| FST10 | High $F_{st}$ | chr7 | 59800000 | 60900000 | 1.1 | 23 | 0.90 | 1.35 |
| FST11 | High $F_{st}$ | chr9 | 82900000 | 84100000 | 1.2 | 34 | 0.91 | 1.43 |
| FST12 | High $F_{st}$ | chr9 | 84900000 | 86200000 | 1.3 | 26 | 0.90 | 1.50 |
| *FST13* | *High $F_{st}$* | *chr9* | *86600000* | *88100000* | *1.5* | *48* | *0.91* | *1.80* |
| FST14 | High $F_{st}$ | chr13 | 28100000 | 29900000 | 1.8 | 53 | 0.90 | 1.55 |

**Supplementary Table ST8.** Comparison of $F_{st}$ values for the p-arm of chromosome 11 with estimates on the p-arms of other chromosomes.

| Chr | Coordinates of p-arm | Size of p-arm, bp | Number of windows | Mean $F_{st}$ | One-sided Mann–Whitney U test, p-value | |
|---|---|---|---|---|---|---|
| | | | | | raw | corrected* |
| chr11 | 0 - 10553932 | 10’553’932 | 84 | 0.74 | NA | NA |
| chr12 | 67017660 - 85782648 | 18’764’988 | 170 | 0.58 | 1.83E-31 | 7.34E-31 |
| chr13 | 0 - 15232218 | 15’232’218 | 120 | 0.54 | 2.52E-31 | 1.01E-30 |
| chr15 | 0 - 12185599 | 12’185’599 | 77 | 0.53 | 4.78E-27 | 1.91E-26 |
| chr18 | 26068618 - 39344853 | 13’276’235 | 113 | 0.57 | 6.28E-30 | 2.51E-29 |

* corrected – Bonferroni-adjusted p-values.

**Supplementary Table ST9.** Estimated divergence times between *Martes* species (*M. zibellina*, *M. martes* and *M. foina*) across phylogenetic studies.

| Node | Study | Divergence Time (Mya) | CI | Markers |
|---|---|---|---|---|
| *M. zibellina* - *M. martes* | (Law et al. 2018) | 1.06 | 0.66 - 1.55 | 46 genes (4 mitochondrial and 42 nuclear genes) |
| | (Hassanin et al. 2021) | 1.1 | unavailable | whole mtDNA |
| | (Koepfli et al. 2008) | 1.1 | 0.6 - 1.6 | 22 mitochondrial genes |
| | (Li et al. 2014) | 0.68 | 0.54 - 0.84 | whole mtDNA |
| | This study (correlated) | 2.05 | 1.52 - 2.70 | 4-fold degenerated sites from 5989 genes |
| | This study (independent) | 1.49 | 1.05 - 1.98 | 4-fold degenerated sites from 5989 genes |
| *M. foina* - *M. zibellina* + | (Law et al. 2018) | 2.56 | 1.93 - 3.29 | 46 genes (4 mitochondrial and 42 nuclear genes) |
| | (Hassanin et al. 2021) | 5.1 | unavailable | whole mtDNA |

| Node | Study | Divergence Time (Mya) | CI | Markers |
|---|---|---|---|---|
| *M. martes* | (Koepfli et al. 2008) | 2.8 | 1.9 - 3.8 | 22 mitochondrial genes |
| | (Li et al. 2014) | 2.93 | 2.35 - 3.54 | whole mtDNA |
| | This study (correlated) | 3.72 | 2.78 - 4.84 | 4-fold degenerated sites from 5989 genes |
| | This study (independent) | 2.77 | 2.14 - 3.55 | 4-fold degenerated sites from 5989 genes |

# References

Hassanin A, Veron G, Ropiquet A, Vuuren BJ van, Lécu A, Goodman SM, Haider J, Nguyen TT. 2021. Evolutionary history of Carnivora (Mammalia, Laurasiatheria) inferred from mitochondrial genomes. *PLOS ONE* 16:e0240770.

Kashtanov SN, Zakharov ES, Begletsov OA, Svishcheva GR, Rychkov SYu, Filimonov PA, Onokhov AA, Levenkova ES, Meschersky IG, Rozhnov VV. 2022. Expansion of the Sable (Martes zibellina L.) from the North of the Central Siberian Plateau into Tundra Ecosystems. *Russ. J. Genet.* 58:955–966.

Koepfli K-P, Deere KA, Slater GJ, Begg C, Begg K, Grassman L, Lucherini M, Veron G, Wayne RK. 2008. Multigene phylogeny of the Mustelidae: Resolving relationships, tempo and biogeographic history of a mammalian adaptive radiation. *BMC Biol.* 6:10.

Law CJ, Slater GJ, Mehta RS. 2018. Lineage Diversity and Size Disparity in Musteloidea: Testing Patterns of Adaptive Radiation Using Molecular and Fossil-Based Methods. *Syst. Biol.* 67:127–144.

Li B, Wolsan M, Wu D, Zhang W, Xu Y, Zeng Z. 2014. Mitochondrial genomes reveal the pattern and timing of marten (Martes), wolverine (Gulo), and fisher (Pekania) diversification. *Mol. Phylogenet. Evol.* 80:156–164.

Liu G, Zhao C, Xu D, Zhang Huanxin, Monakhov V, Shang S, Gao X, Sha W, Ma J, Zhang W, et al. 2020. First Draft Genome of the Sable, Martes zibellina. *Genome Biol. Evol.* 12:59–65.

Marciszak A, Lipecki G, Spassov N. 2024. Martes wenzensis Stach, 1959 within the early history of the genus Martes Pinel, 1792. *Acta Geol. Pol.* 74:e4.

Rozhnov VV, Pishchulina SL, Meschersky IG, Simakin LV. 2013. On the ratio of phenotype and genotype of sable and pine marten in sympatry zone in the Northern Urals. *Mosc. Univ. Biol. Sci. Bull.* 68:178–181.

Salesa MJ, Antón M, Siliceo G, Pesquero MD, Morales J, Alcalá L. 2013. A non-aquatic otter (Mammalia, Carnivora, Mustelidae) from the Late Miocene (Vallesian, MN 10) of La Roma 2 (Alfambra, Teruel, Spain): systematics and functional anatomy. *Zool. J. Linn. Soc.* 169:448–482.

Samuels JX, Cavin J. 2013. The earliest known fisher (Mustelidae), a new species from the Rattlesnake Formation of Oregon. *J. Vertebr. Paleontol.* 33:448–454.

Stach J. 1959. On some Mustelinae from the Pliocene bone breccia of Węże. *Acta Palaeontol. Pol.* 4.

Tomarovsky AA, Khan R, Dudchenko O, Beklemisheva VR, Perelman PL, Totikov AA, Serdyukova NA, Bulyonkova TM, Pobedintseva M, Abramov AV, et al. 2025. Novel chromosome-length genome assemblies of three distinct subspecies of pine marten, sable, and yellow-throated marten (genus Martes, family Mustelidae). :2025.09.22.677678. Available from: https://www.biorxiv.org/content/10.1101/2025.09.22.677678v1

Wang X, McKenna MC, Dashzeveg D. 2005. Amphicticeps and Amphicynodon (Arctoidea, Carnivora) from Hsanda Gol Formation, central Mongolia and phylogeny of basal arctoids with comments on zoogeography. *Am. Mus. Novit.* 2005:1–60.

Wolsan M. 1990. Lower Pleistocene carnivores of Poland. *Quarterpäläntologie* 8:277–280.
