# Supplementary material for: Genomics of Sable (Martes zibellina) × Pine Marten (Martes martes) Hybridization
Source: Genome Biol Evol. 2026 Mar 5;18(3):evag018. doi: 10.1093/gbe/evag018 (PMC12960073; doi:10.1093/gbe/evag018)
Supplement: evag018_Supplementary_Data [file evag018_supplementary_data.zip › SupplementaryFiles/SupplementaryFile_6.Distribution_of_the_heterozygous_and_homozygous along_chromosomes.pdf]

10xmzib

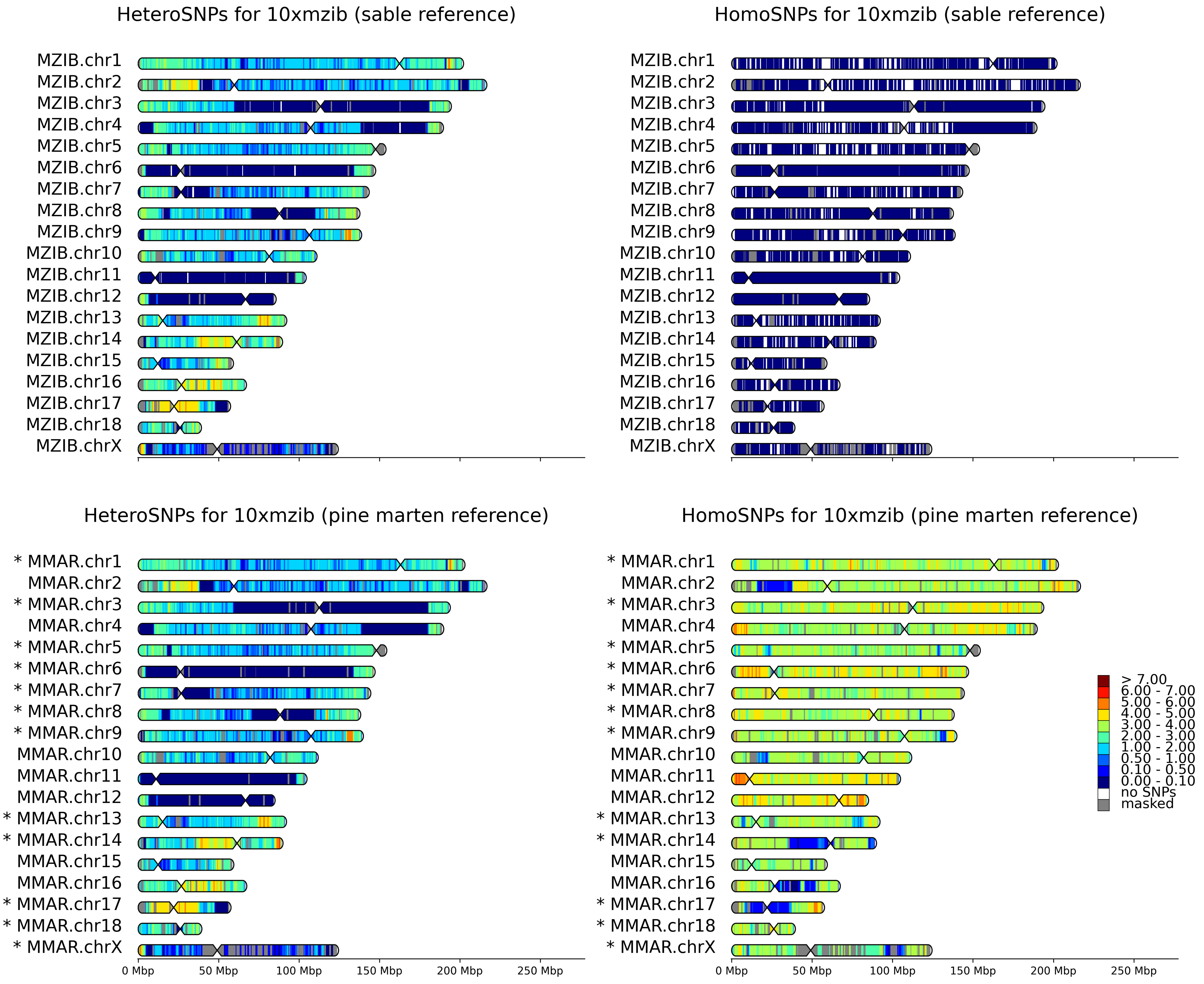
HeteroSNPs for 10xmzib (sable reference)
HomoSNPs for 10xmzib (sable reference)
MZIB.chr1
MZIB.chr2
MZIB.chr3
MZIB.chr4
MZIB.chr5
MZIB.chr6
MZIB.chr7
MZIB.chr8
MZIB.chr9
MZIB.chr10
MZIB.chr11
MZIB.chr12
MZIB.chr13
MZIB.chr14
MZIB.chr15
MZIB.chr16
MZIB.chr17
MZIB.chr18
MZIB.chrX
HeteroSNPs for 10xmzib (pine marten reference)
HomoSNPs for 10xmzib (pine marten reference)
* MMAR.chr1
MMAR.chr2
* MMAR.chr3
MMAR.chr4
* MMAR.chr5
* MMAR.chr6
* MMAR.chr7
* MMAR.chr8
* MMAR.chr9
MMAR.chr10
MMAR.chr11
MMAR.chr12
* MMAR.chr13
* MMAR.chr14
MMAR.chr15
MMAR.chr16
* MMAR.chr17
* MMAR.chr18
* MMAR.chrX
0 Mbp
50 Mbp
100 Mbp
150 Mbp
200 Mbp
250 Mbp
> 7.00
6.00 - 7.00
5.00 - 6.00
4.00 - 5.00
3.00 - 4.00
2.00 - 3.00
1.00 - 2.00
0.50 - 1.00
0.10 - 0.50
0.00 - 0.10
no SNPs
masked

S26

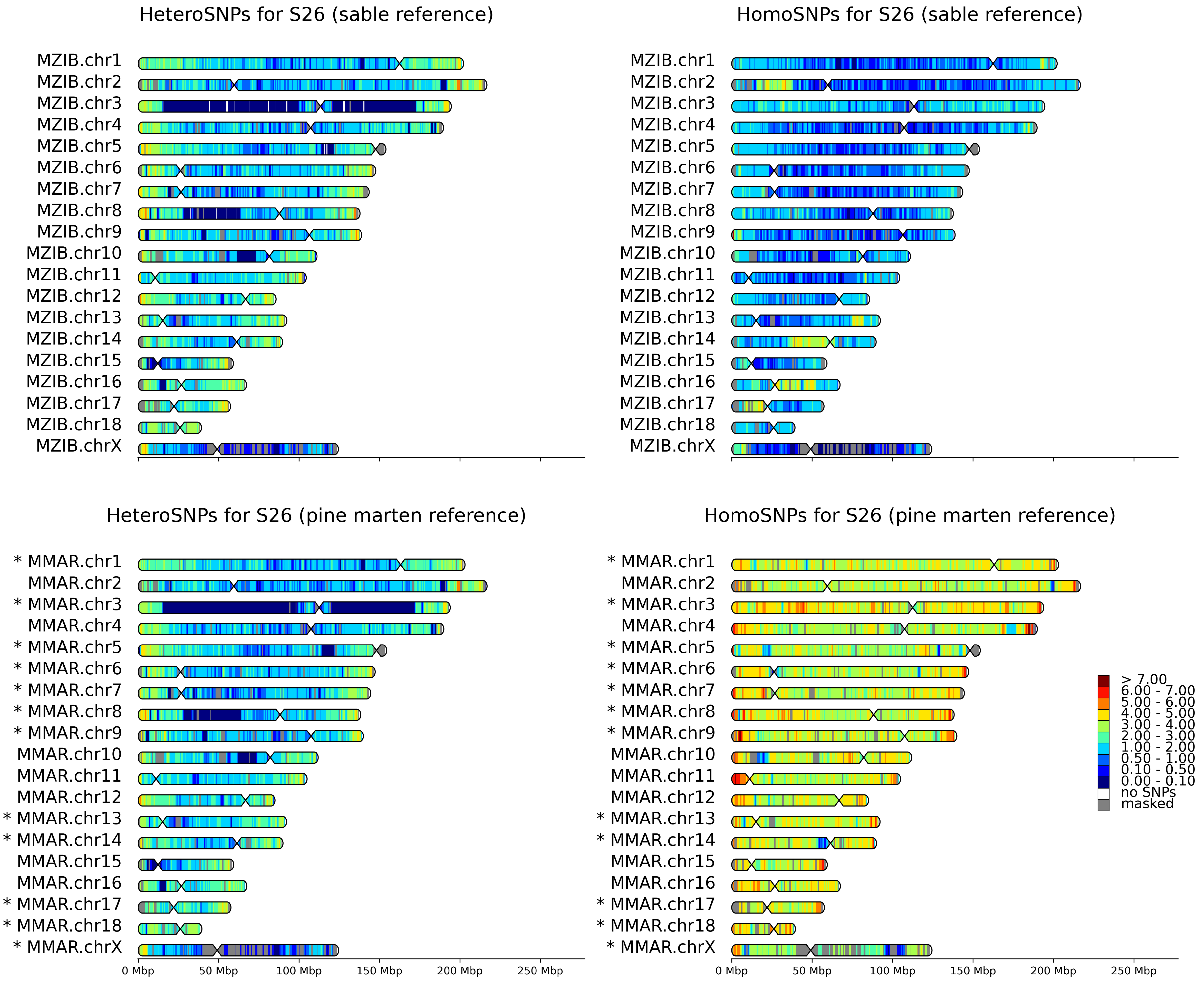

HeteroSNPs for S26 (sable reference)
HomoSNPs for S26 (sable reference)
MZIB.chr1
MZIB.chr2
MZIB.chr3
MZIB.chr4
MZIB.chr5
MZIB.chr6
MZIB.chr7
MZIB.chr8
MZIB.chr9
MZIB.chr10
MZIB.chr11
MZIB.chr12
MZIB.chr13
MZIB.chr14
MZIB.chr15
MZIB.chr16
MZIB.chr17
MZIB.chr18
MZIB.chrX
HeteroSNPs for S26 (pine marten reference)
HomoSNPs for S26 (pine marten reference)
* MMAR.chr1
MMAR.chr2
* MMAR.chr3
MMAR.chr4
* MMAR.chr5
* MMAR.chr6
* MMAR.chr7
* MMAR.chr8
* MMAR.chr9
MMAR.chr10
MMAR.chr11
MMAR.chr12
* MMAR.chr13
* MMAR.chr14
MMAR.chr15
MMAR.chr16
* MMAR.chr17
* MMAR.chr18
* MMAR.chrX
0 Mbp
50 Mbp
100 Mbp
150 Mbp
200 Mbp
250 Mbp
> 7.00
6.00 - 7.00
5.00 - 6.00
4.00 - 5.00
3.00 - 4.00
2.00 - 3.00
1.00 - 2.00
0.50 - 1.00
0.10 - 0.50
0.00 - 0.10
no SNPs
masked

T8

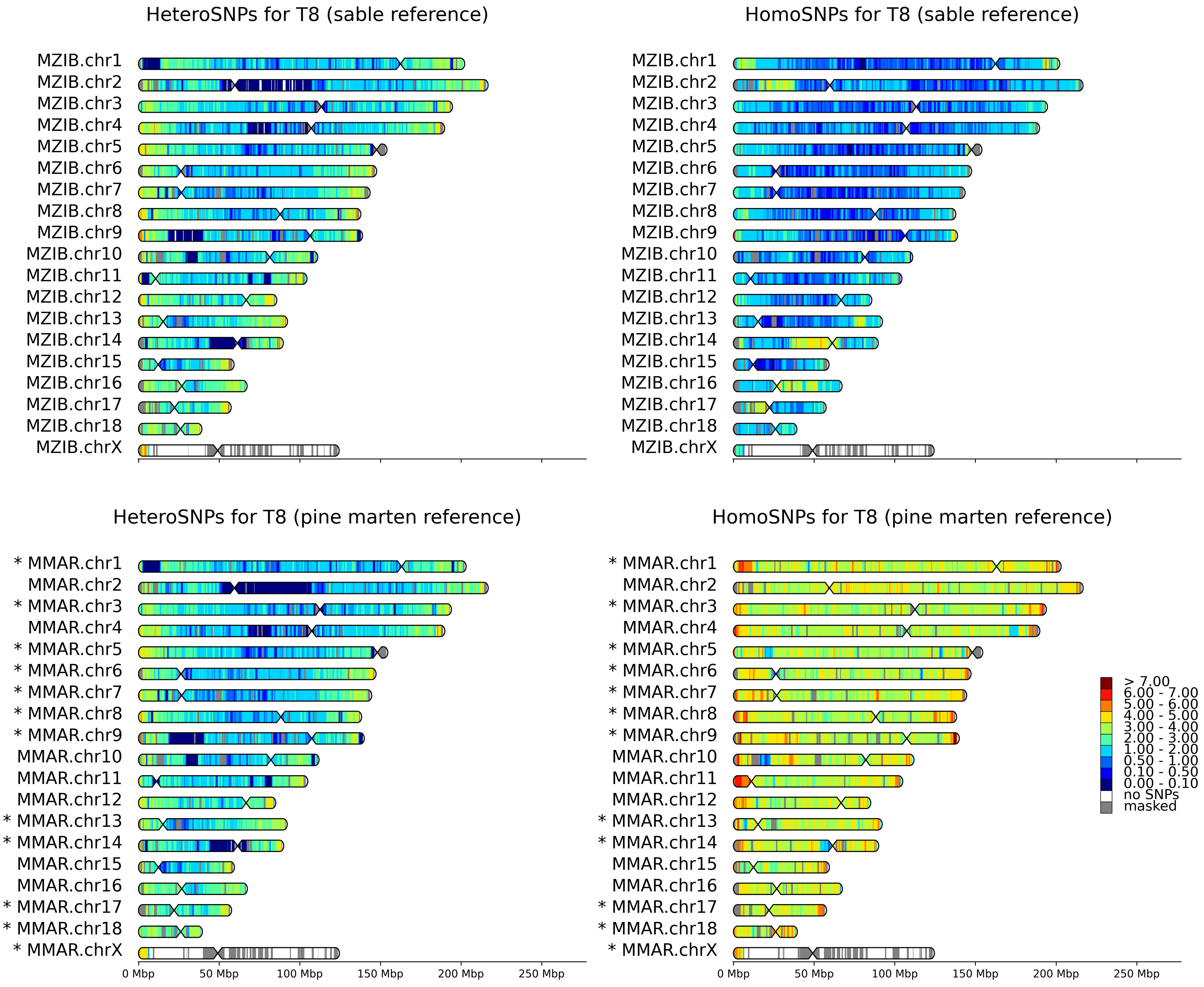
HeteroSNPs for T8 (sable reference)
HomoSNPs for T8 (sable reference)
MZIB.chr1
MZIB.chr2
MZIB.chr3
MZIB.chr4
MZIB.chr5
MZIB.chr6
MZIB.chr7
MZIB.chr8
MZIB.chr9
MZIB.chr10
MZIB.chr11
MZIB.chr12
MZIB.chr13
MZIB.chr14
MZIB.chr15
MZIB.chr16
MZIB.chr17
MZIB.chr18
MZIB.chrX
HeteroSNPs for T8 (pine marten reference)
HomoSNPs for T8 (pine marten reference)
* MMAR.chr1
MMAR.chr2
* MMAR.chr3
MMAR.chr4
* MMAR.chr5
* MMAR.chr6
* MMAR.chr7
* MMAR.chr8
* MMAR.chr9
MMAR.chr10
MMAR.chr11
MMAR.chr12
* MMAR.chr13
* MMAR.chr14
MMAR.chr15
MMAR.chr16
* MMAR.chr17
* MMAR.chr18
* MMAR.chrX
0 Mbp
50 Mbp
100 Mbp
150 Mbp
200 Mbp
250 Mbp
> 7.00
6.00 - 7.00
5.00 - 6.00
4.00 - 5.00
3.00 - 4.00
2.00 - 3.00
1.00 - 2.00
0.50 - 1.00
0.10 - 0.50
0.00 - 0.10
no SNPs
masked

T18

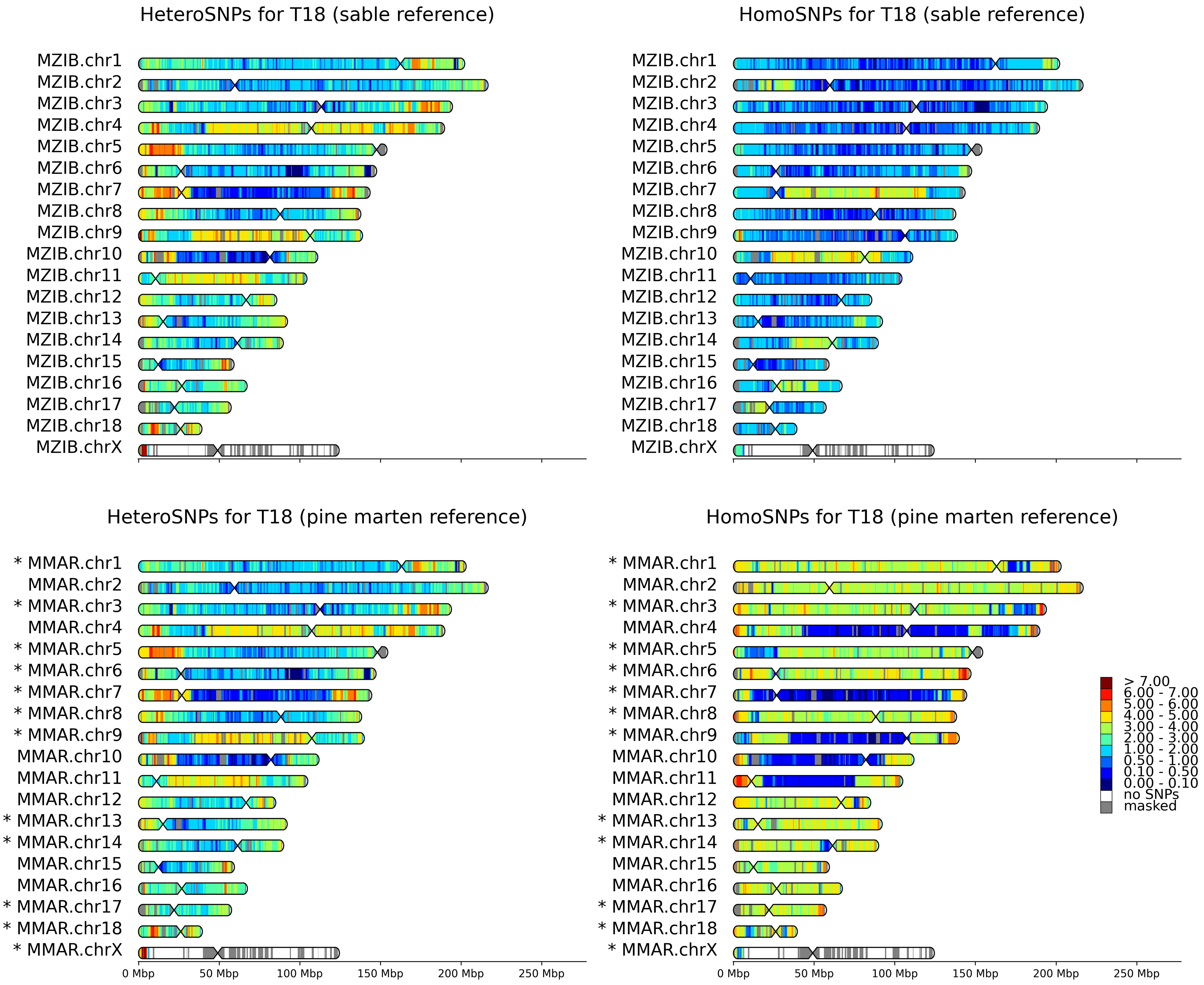
HeteroSNPs for T18 (sable reference)
HomoSNPs for T18 (sable reference)
HeteroSNPs for T18 (pine marten reference)
HomoSNPs for T18 (pine marten reference)
MZIB.chr1
MZIB.chr2
MZIB.chr3
MZIB.chr4
MZIB.chr5
MZIB.chr6
MZIB.chr7
MZIB.chr8
MZIB.chr9
MZIB.chr10
MZIB.chr11
MZIB.chr12
MZIB.chr13
MZIB.chr14
MZIB.chr15
MZIB.chr16
MZIB.chr17
MZIB.chr18
MZIB.chrX
* MMAR.chr1
MMAR.chr2
* MMAR.chr3
MMAR.chr4
* MMAR.chr5
* MMAR.chr6
* MMAR.chr7
* MMAR.chr8
* MMAR.chr9
MMAR.chr10
MMAR.chr11
MMAR.chr12
* MMAR.chr13
* MMAR.chr14
MMAR.chr15
MMAR.chr16
* MMAR.chr17
* MMAR.chr18
* MMAR.chrX
0 Mbp
50 Mbp
100 Mbp
150 Mbp
200 Mbp
250 Mbp
> 7.00
6.00 - 7.00
5.00 - 6.00
4.00 - 5.00
3.00 - 4.00
2.00 - 3.00
1.00 - 2.00
0.50 - 1.00
0.10 - 0.50
0.00 - 0.10
no SNPs
masked

T26

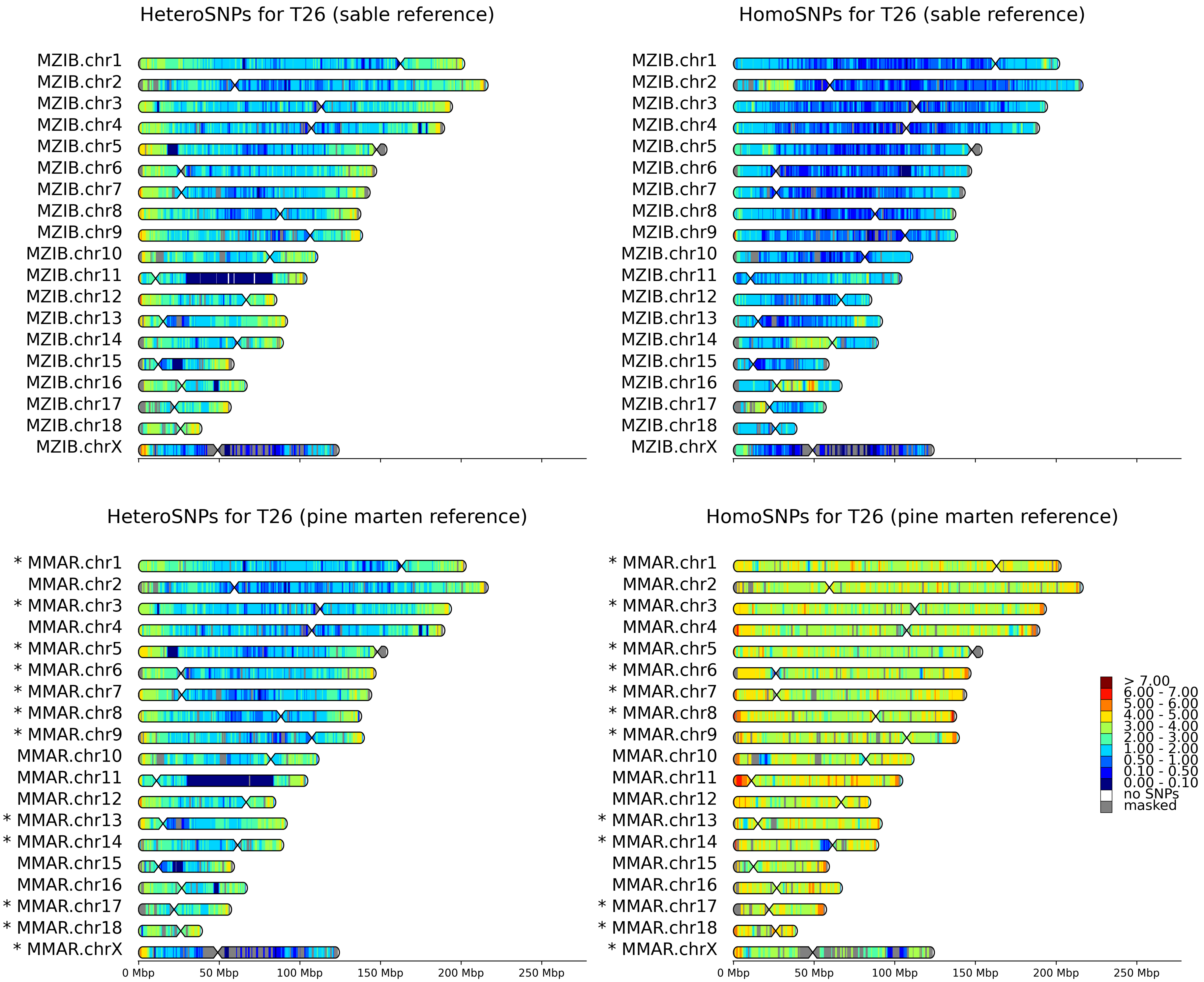
HeteroSNPs for T26 (sable reference)
HomoSNPs for T26 (sable reference)
MZIB.chr1
MZIB.chr2
MZIB.chr3
MZIB.chr4
MZIB.chr5
MZIB.chr6
MZIB.chr7
MZIB.chr8
MZIB.chr9
MZIB.chr10
MZIB.chr11
MZIB.chr12
MZIB.chr13
MZIB.chr14
MZIB.chr15
MZIB.chr16
MZIB.chr17
MZIB.chr18
MZIB.chrX
HeteroSNPs for T26 (pine marten reference)
HomoSNPs for T26 (pine marten reference)
* MMAR.chr1
MMAR.chr2
* MMAR.chr3
MMAR.chr4
* MMAR.chr5
* MMAR.chr6
* MMAR.chr7
* MMAR.chr8
* MMAR.chr9
MMAR.chr10
MMAR.chr11
MMAR.chr12
* MMAR.chr13
* MMAR.chr14
MMAR.chr15
MMAR.chr16
* MMAR.chr17
* MMAR.chr18
* MMAR.chrX
0 Mbp
50 Mbp
100 Mbp
150 Mbp
200 Mbp
250 Mbp
> 7.00
6.00 - 7.00
5.00 - 6.00
4.00 - 5.00
3.00 - 4.00
2.00 - 3.00
1.00 - 2.00
0.50 - 1.00
0.10 - 0.50
0.00 - 0.10
no SNPs
masked

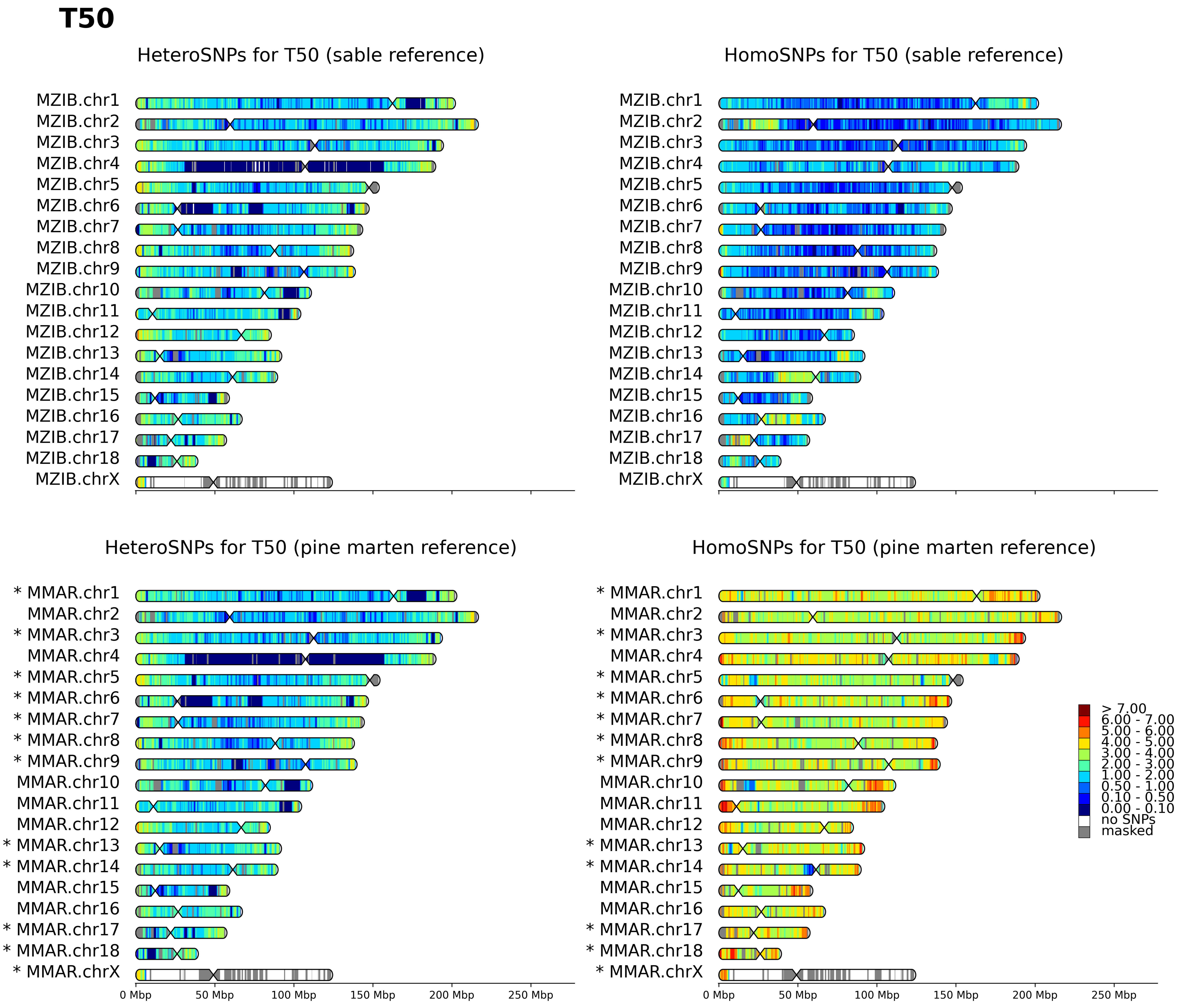
T50
HeteroSNPs for T50 (sable reference)
HomoSNPs for T50 (sable reference)
MZIB.chr1
MZIB.chr2
MZIB.chr3
MZIB.chr4
MZIB.chr5
MZIB.chr6
MZIB.chr7
MZIB.chr8
MZIB.chr9
MZIB.chr10
MZIB.chr11
MZIB.chr12
MZIB.chr13
MZIB.chr14
MZIB.chr15
MZIB.chr16
MZIB.chr17
MZIB.chr18
MZIB.chrX
HeteroSNPs for T50 (pine marten reference)
HomoSNPs for T50 (pine marten reference)
* MMAR.chr1
MMAR.chr2
* MMAR.chr3
MMAR.chr4
* MMAR.chr5
* MMAR.chr6
* MMAR.chr7
* MMAR.chr8
* MMAR.chr9
MMAR.chr10
MMAR.chr11
MMAR.chr12
* MMAR.chr13
* MMAR.chr14
MMAR.chr15
MMAR.chr16
* MMAR.chr17
* MMAR.chr18
* MMAR.chrX
0 Mbp
50 Mbp
100 Mbp
150 Mbp
200 Mbp
250 Mbp
> 7.00
6.00 - 7.00
5.00 - 6.00
4.00 - 5.00
3.00 - 4.00
2.00 - 3.00
1.00 - 2.00
0.50 - 1.00
0.10 - 0.50
0.00 - 0.10
no SNPs
masked

T72

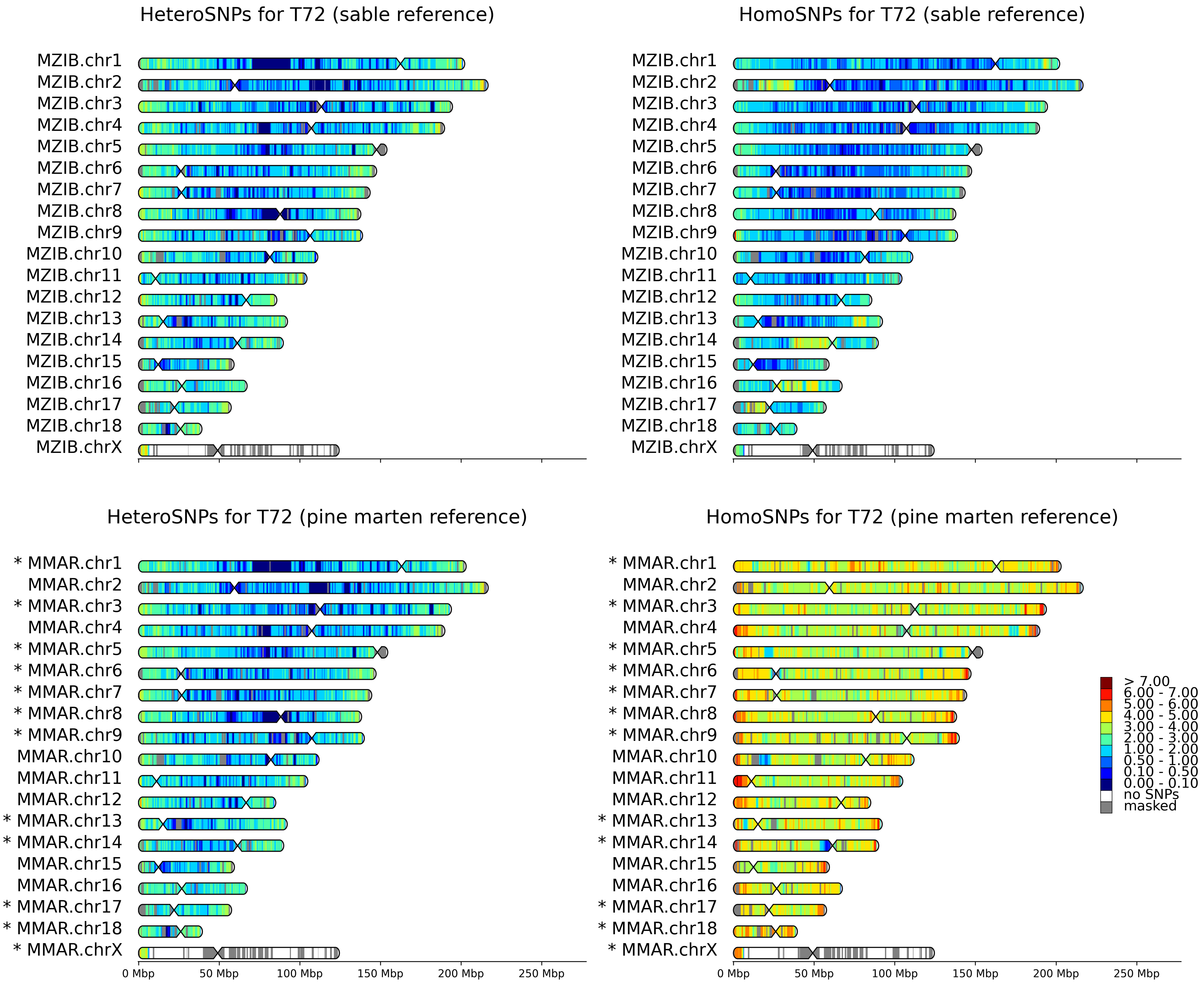
HeteroSNPs for T72 (sable reference)
HomoSNPs for T72 (sable reference)
MZIB.chr1
MZIB.chr2
MZIB.chr3
MZIB.chr4
MZIB.chr5
MZIB.chr6
MZIB.chr7
MZIB.chr8
MZIB.chr9
MZIB.chr10
MZIB.chr11
MZIB.chr12
MZIB.chr13
MZIB.chr14
MZIB.chr15
MZIB.chr16
MZIB.chr17
MZIB.chr18
MZIB.chrX
HeteroSNPs for T72 (pine marten reference)
HomoSNPs for T72 (pine marten reference)
* MMAR.chr1
MMAR.chr2
* MMAR.chr3
MMAR.chr4
* MMAR.chr5
* MMAR.chr6
* MMAR.chr7
* MMAR.chr8
* MMAR.chr9
MMAR.chr10
MMAR.chr11
MMAR.chr12
* MMAR.chr13
* MMAR.chr14
MMAR.chr15
MMAR.chr16
* MMAR.chr17
* MMAR.chr18
* MMAR.chrX
0 Mbp
50 Mbp
100 Mbp
150 Mbp
200 Mbp
250 Mbp
> 7.00
6.00 - 7.00
5.00 - 6.00
4.00 - 5.00
3.00 - 4.00
2.00 - 3.00
1.00 - 2.00
0.50 - 1.00
0.10 - 0.50
0.00 - 0.10
no SNPs
masked

T90

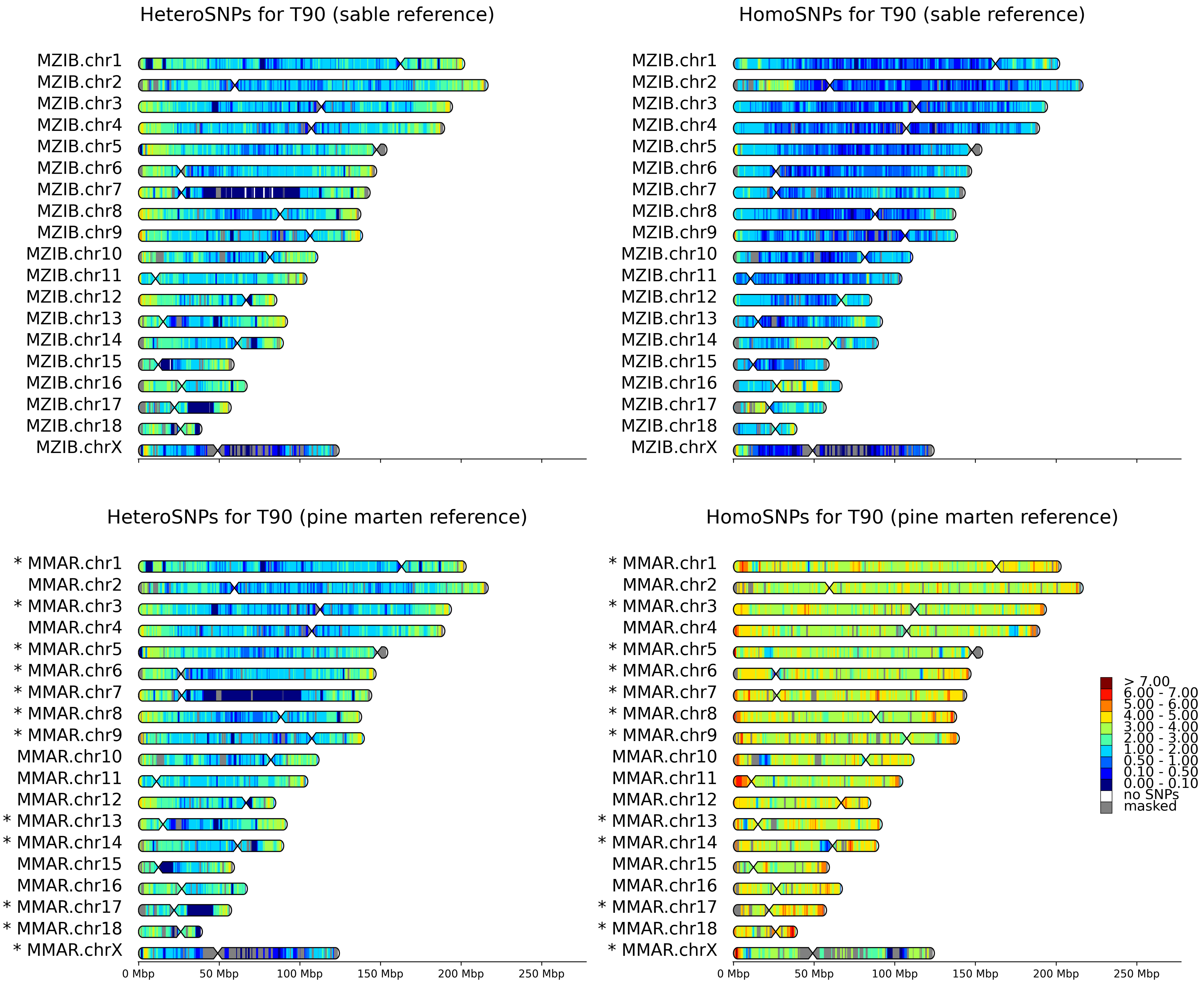
HeteroSNPs for T90 (sable reference)
HomoSNPs for T90 (sable reference)
MZIB.chr1
MZIB.chr2
MZIB.chr3
MZIB.chr4
MZIB.chr5
MZIB.chr6
MZIB.chr7
MZIB.chr8
MZIB.chr9
MZIB.chr10
MZIB.chr11
MZIB.chr12
MZIB.chr13
MZIB.chr14
MZIB.chr15
MZIB.chr16
MZIB.chr17
MZIB.chr18
MZIB.chrX
HeteroSNPs for T90 (pine marten reference)
HomoSNPs for T90 (pine marten reference)
* MMAR.chr1
MMAR.chr2
* MMAR.chr3
MMAR.chr4
* MMAR.chr5
* MMAR.chr6
* MMAR.chr7
* MMAR.chr8
* MMAR.chr9
MMAR.chr10
MMAR.chr11
MMAR.chr12
* MMAR.chr13
* MMAR.chr14
MMAR.chr15
MMAR.chr16
* MMAR.chr17
* MMAR.chr18
* MMAR.chrX
0 Mbp
50 Mbp
100 Mbp
150 Mbp
200 Mbp
250 Mbp
> 7.00
6.00 - 7.00
5.00 - 6.00
4.00 - 5.00
3.00 - 4.00
2.00 - 3.00
1.00 - 2.00
0.50 - 1.00
0.10 - 0.50
0.00 - 0.10
no SNPs
masked

T104

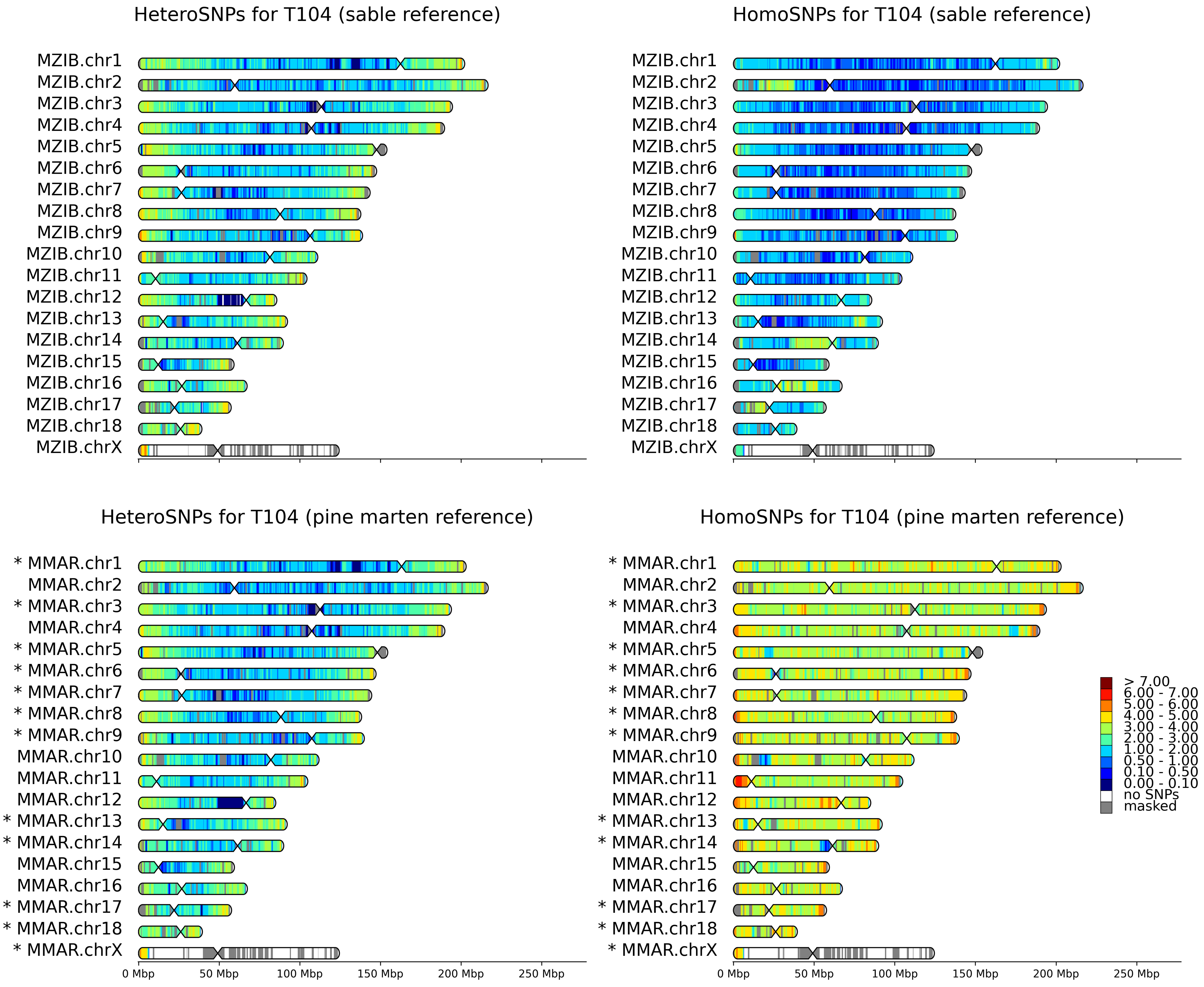

HeteroSNPs for T104 (sable reference)
HomoSNPs for T104 (sable reference)
MZIB.chr1
MZIB.chr2
MZIB.chr3
MZIB.chr4
MZIB.chr5
MZIB.chr6
MZIB.chr7
MZIB.chr8
MZIB.chr9
MZIB.chr10
MZIB.chr11
MZIB.chr12
MZIB.chr13
MZIB.chr14
MZIB.chr15
MZIB.chr16
MZIB.chr17
MZIB.chr18
MZIB.chrX
HeteroSNPs for T104 (pine marten reference)
HomoSNPs for T104 (pine marten reference)
* MMAR.chr1
MMAR.chr2
* MMAR.chr3
MMAR.chr4
* MMAR.chr5
* MMAR.chr6
* MMAR.chr7
* MMAR.chr8
* MMAR.chr9
MMAR.chr10
MMAR.chr11
MMAR.chr12
* MMAR.chr13
* MMAR.chr14
MMAR.chr15
MMAR.chr16
* MMAR.chr17
* MMAR.chr18
* MMAR.chrX
0 Mbp
50 Mbp
100 Mbp
150 Mbp
200 Mbp
250 Mbp
> 7.00
6.00 - 7.00
5.00 - 6.00
4.00 - 5.00
3.00 - 4.00
2.00 - 3.00
1.00 - 2.00
0.50 - 1.00
0.10 - 0.50
0.00 - 0.10
no SNPs
masked

T118

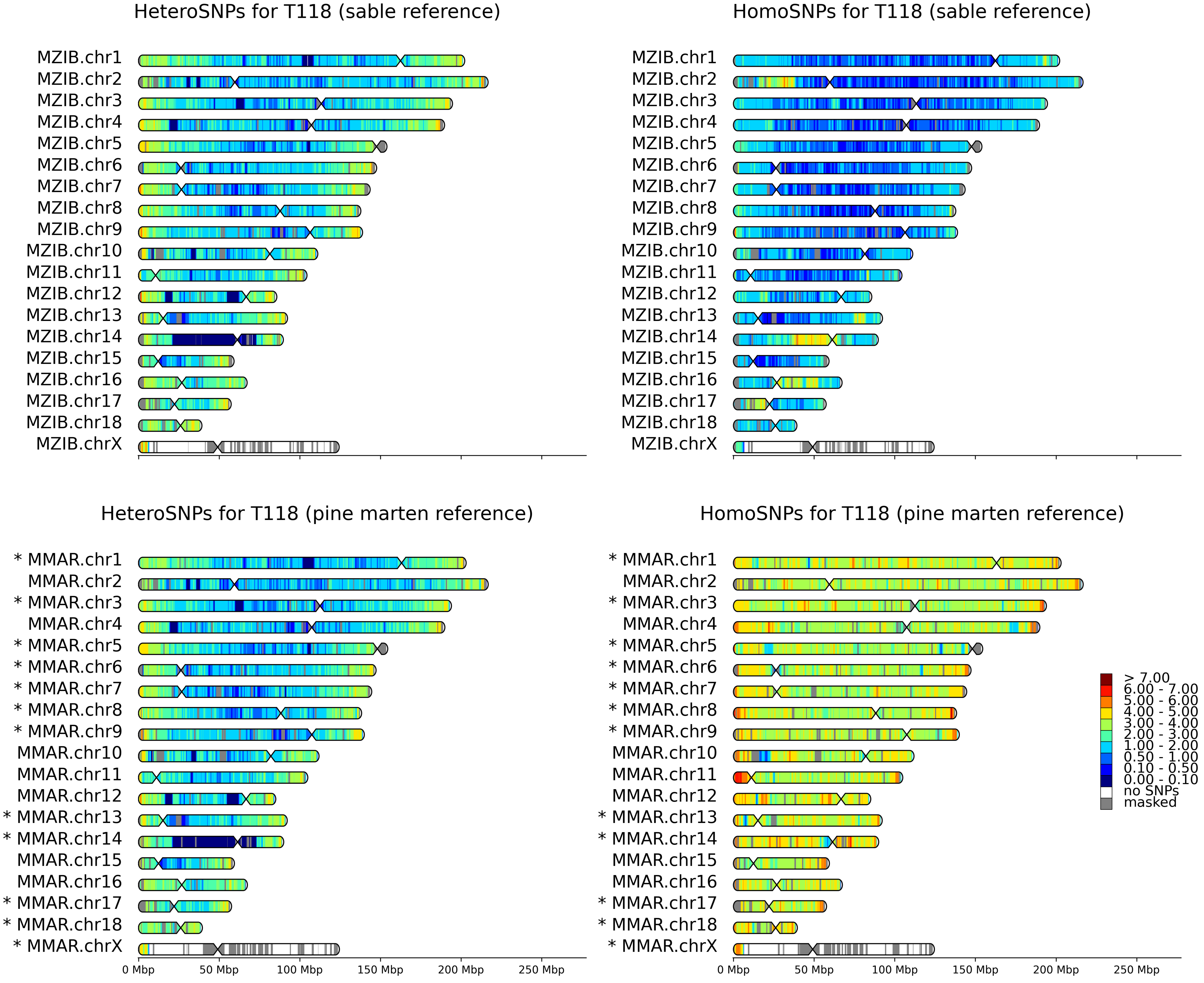

HeteroSNPs for T118 (sable reference)
HomoSNPs for T118 (sable reference)
MZIB.chr1
MZIB.chr2
MZIB.chr3
MZIB.chr4
MZIB.chr5
MZIB.chr6
MZIB.chr7
MZIB.chr8
MZIB.chr9
MZIB.chr10
MZIB.chr11
MZIB.chr12
MZIB.chr13
MZIB.chr14
MZIB.chr15
MZIB.chr16
MZIB.chr17
MZIB.chr18
MZIB.chrX
HeteroSNPs for T118 (pine marten reference)
HomoSNPs for T118 (pine marten reference)
* MMAR.chr1
MMAR.chr2
* MMAR.chr3
MMAR.chr4
* MMAR.chr5
* MMAR.chr6
* MMAR.chr7
* MMAR.chr8
* MMAR.chr9
MMAR.chr10
MMAR.chr11
MMAR.chr12
* MMAR.chr13
* MMAR.chr14
MMAR.chr15
MMAR.chr16
* MMAR.chr17
* MMAR.chr18
* MMAR.chrX
0 Mbp
50 Mbp
100 Mbp
150 Mbp
200 Mbp
250 Mbp
> 7.00
6.00 - 7.00
5.00 - 6.00
4.00 - 5.00
3.00 - 4.00
2.00 - 3.00
1.00 - 2.00
0.50 - 1.00
0.10 - 0.50
0.00 - 0.10
no SNPs
masked

T148

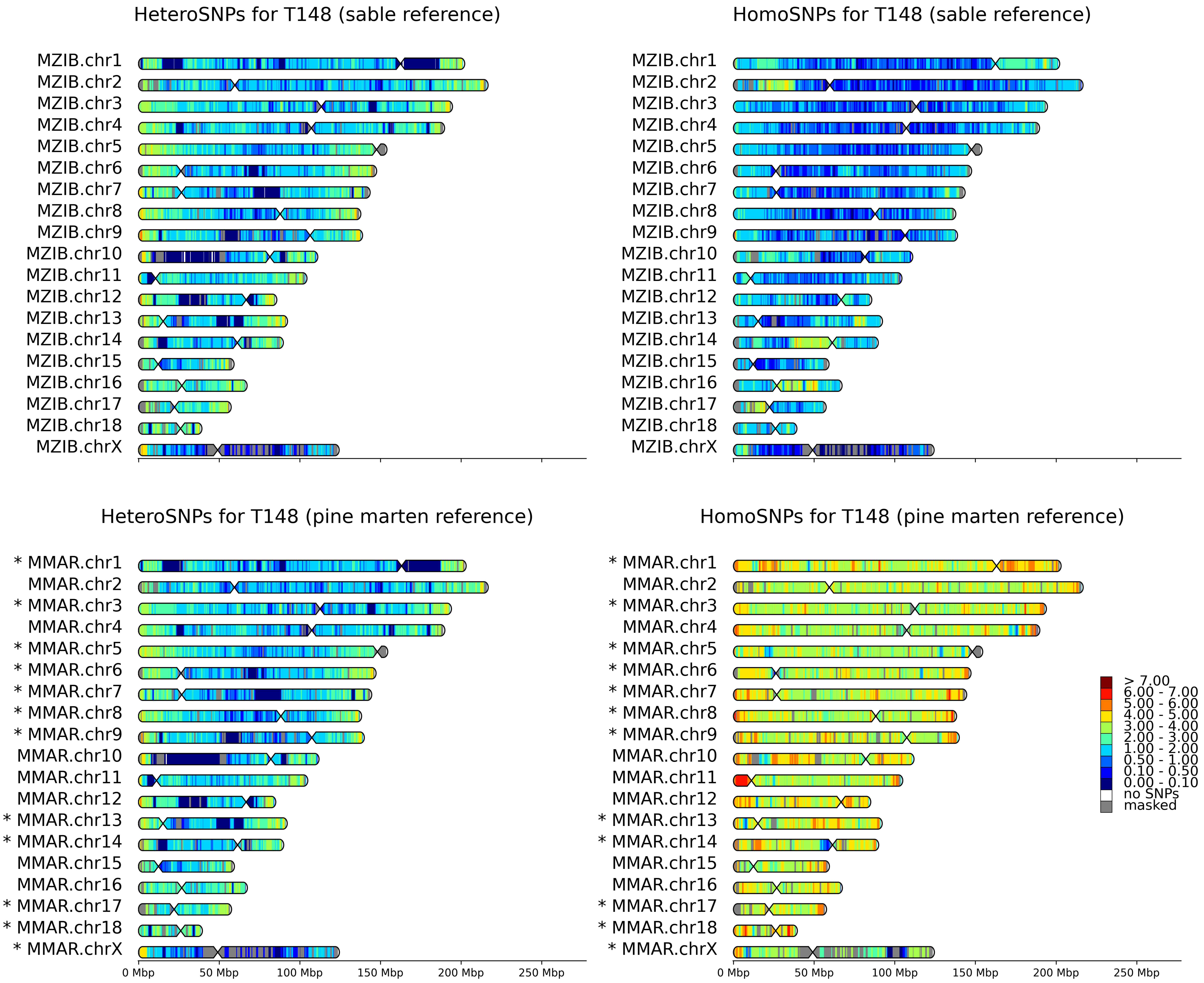
HeteroSNPs for T148 (sable reference)
HomoSNPs for T148 (sable reference)
MZIB.chr1
MZIB.chr2
MZIB.chr3
MZIB.chr4
MZIB.chr5
MZIB.chr6
MZIB.chr7
MZIB.chr8
MZIB.chr9
MZIB.chr10
MZIB.chr11
MZIB.chr12
MZIB.chr13
MZIB.chr14
MZIB.chr15
MZIB.chr16
MZIB.chr17
MZIB.chr18
MZIB.chrX
HeteroSNPs for T148 (pine marten reference)
HomoSNPs for T148 (pine marten reference)
* MMAR.chr1
MMAR.chr2
* MMAR.chr3
MMAR.chr4
* MMAR.chr5
* MMAR.chr6
* MMAR.chr7
* MMAR.chr8
* MMAR.chr9
MMAR.chr10
MMAR.chr11
MMAR.chr12
* MMAR.chr13
* MMAR.chr14
MMAR.chr15
MMAR.chr16
* MMAR.chr17
* MMAR.chr18
* MMAR.chrX
0 Mbp
50 Mbp
100 Mbp
150 Mbp
200 Mbp
250 Mbp
> 7.00
6.00 - 7.00
5.00 - 6.00
4.00 - 5.00
3.00 - 4.00
2.00 - 3.00
1.00 - 2.00
0.50 - 1.00
0.10 - 0.50
0.00 - 0.10
no SNPs
masked

T150

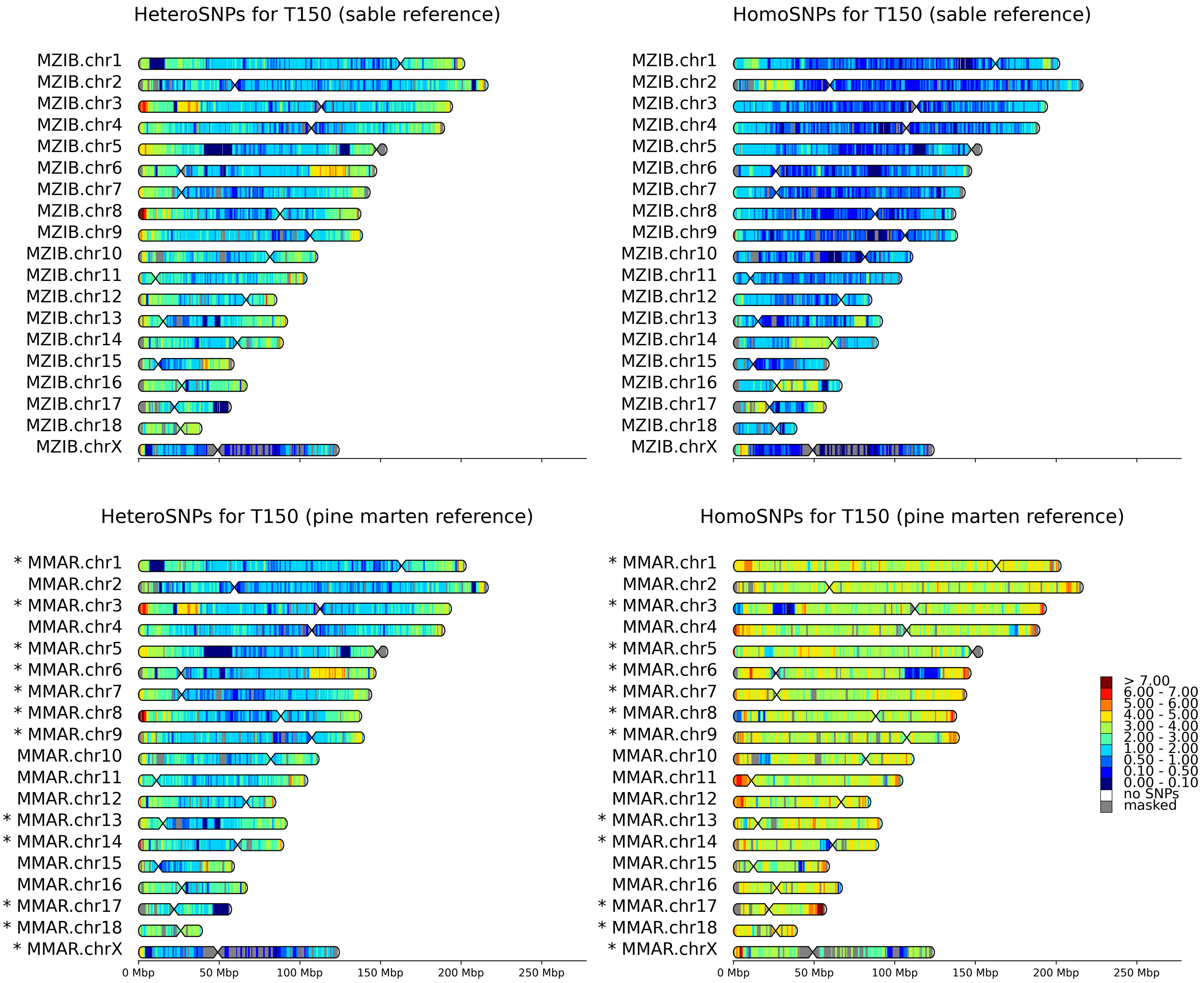

HeteroSNPs for T150 (sable reference)
HomoSNPs for T150 (sable reference)
MZIB.chr1
MZIB.chr2
MZIB.chr3
MZIB.chr4
MZIB.chr5
MZIB.chr6
MZIB.chr7
MZIB.chr8
MZIB.chr9
MZIB.chr10
MZIB.chr11
MZIB.chr12
MZIB.chr13
MZIB.chr14
MZIB.chr15
MZIB.chr16
MZIB.chr17
MZIB.chr18
MZIB.chrX
HeteroSNPs for T150 (pine marten reference)
HomoSNPs for T150 (pine marten reference)
* MMAR.chr1
MMAR.chr2
* MMAR.chr3
MMAR.chr4
* MMAR.chr5
* MMAR.chr6
* MMAR.chr7
* MMAR.chr8
* MMAR.chr9
MMAR.chr10
MMAR.chr11
MMAR.chr12
* MMAR.chr13
* MMAR.chr14
MMAR.chr15
MMAR.chr16
* MMAR.chr17
* MMAR.chr18
* MMAR.chrX
0 Mbp
50 Mbp
100 Mbp
150 Mbp
200 Mbp
250 Mbp
> 7.00
6.00 - 7.00
5.00 - 6.00
4.00 - 5.00
3.00 - 4.00
2.00 - 3.00
1.00 - 2.00
0.50 - 1.00
0.10 - 0.50
0.00 - 0.10
no SNPs
masked

T194

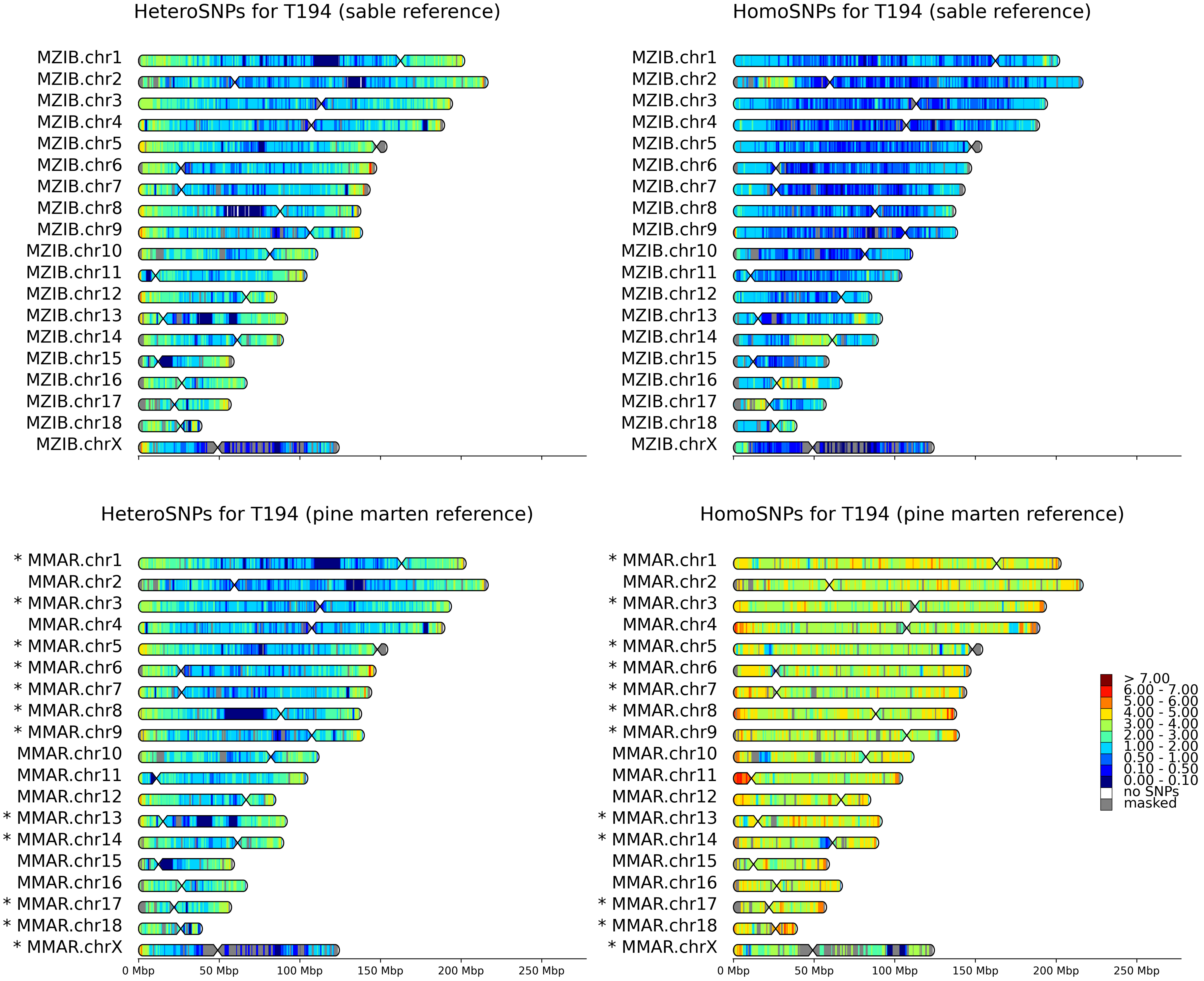
HeteroSNPs for T194 (sable reference)
HomoSNPs for T194 (sable reference)
MZIB.chr1
MZIB.chr2
MZIB.chr3
MZIB.chr4
MZIB.chr5
MZIB.chr6
MZIB.chr7
MZIB.chr8
MZIB.chr9
MZIB.chr10
MZIB.chr11
MZIB.chr12
MZIB.chr13
MZIB.chr14
MZIB.chr15
MZIB.chr16
MZIB.chr17
MZIB.chr18
MZIB.chrX
HeteroSNPs for T194 (pine marten reference)
HomoSNPs for T194 (pine marten reference)
* MMAR.chr1
MMAR.chr2
* MMAR.chr3
MMAR.chr4
* MMAR.chr5
* MMAR.chr6
* MMAR.chr7
* MMAR.chr8
* MMAR.chr9
MMAR.chr10
MMAR.chr11
MMAR.chr12
* MMAR.chr13
* MMAR.chr14
MMAR.chr15
MMAR.chr16
* MMAR.chr17
* MMAR.chr18
* MMAR.chrX
0 Mbp
50 Mbp
100 Mbp
150 Mbp
200 Mbp
250 Mbp
> 7.00
6.00 - 7.00
5.00 - 6.00
4.00 - 5.00
3.00 - 4.00
2.00 - 3.00
1.00 - 2.00
0.50 - 1.00
0.10 - 0.50
0.00 - 0.10
no SNPs
masked

CHN

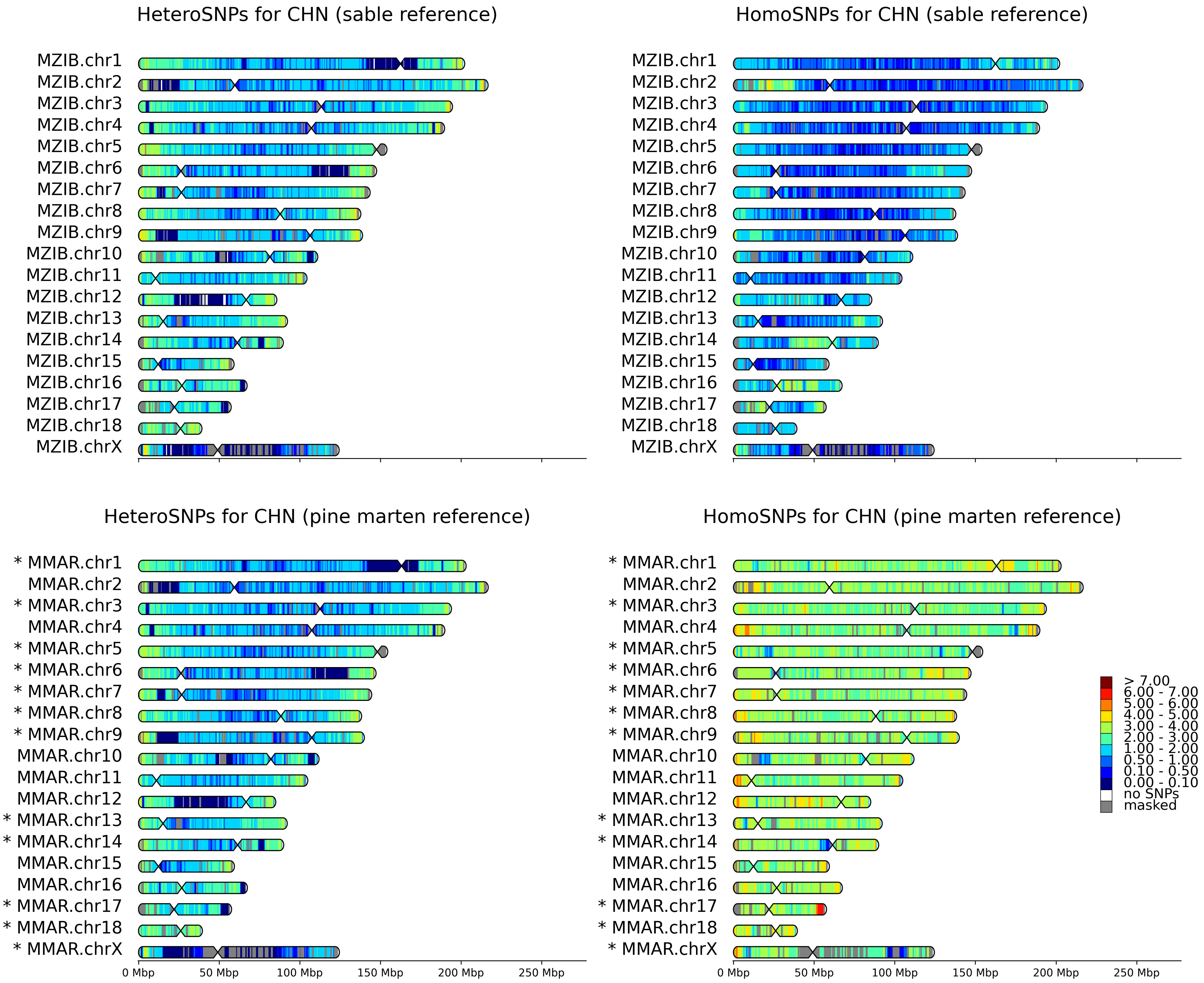
HeteroSNPs for CHN (sable reference)
HomoSNPs for CHN (sable reference)
MZIB.chr1
MZIB.chr2
MZIB.chr3
MZIB.chr4
MZIB.chr5
MZIB.chr6
MZIB.chr7
MZIB.chr8
MZIB.chr9
MZIB.chr10
MZIB.chr11
MZIB.chr12
MZIB.chr13
MZIB.chr14
MZIB.chr15
MZIB.chr16
MZIB.chr17
MZIB.chr18
MZIB.chrX
HeteroSNPs for CHN (pine marten reference)
HomoSNPs for CHN (pine marten reference)
* MMAR.chr1
MMAR.chr2
* MMAR.chr3
MMAR.chr4
* MMAR.chr5
* MMAR.chr6
* MMAR.chr7
* MMAR.chr8
* MMAR.chr9
MMAR.chr10
MMAR.chr11
MMAR.chr12
* MMAR.chr13
* MMAR.chr14
MMAR.chr15
MMAR.chr16
* MMAR.chr17
* MMAR.chr18
* MMAR.chrX
0 Mbp
50 Mbp
100 Mbp
150 Mbp
200 Mbp
250 Mbp
> 7.00
6.00 - 7.00
5.00 - 6.00
4.00 - 5.00
3.00 - 4.00
2.00 - 3.00
1.00 - 2.00
0.50 - 1.00
0.10 - 0.50
0.00 - 0.10
no SNPs
masked

10xmmar

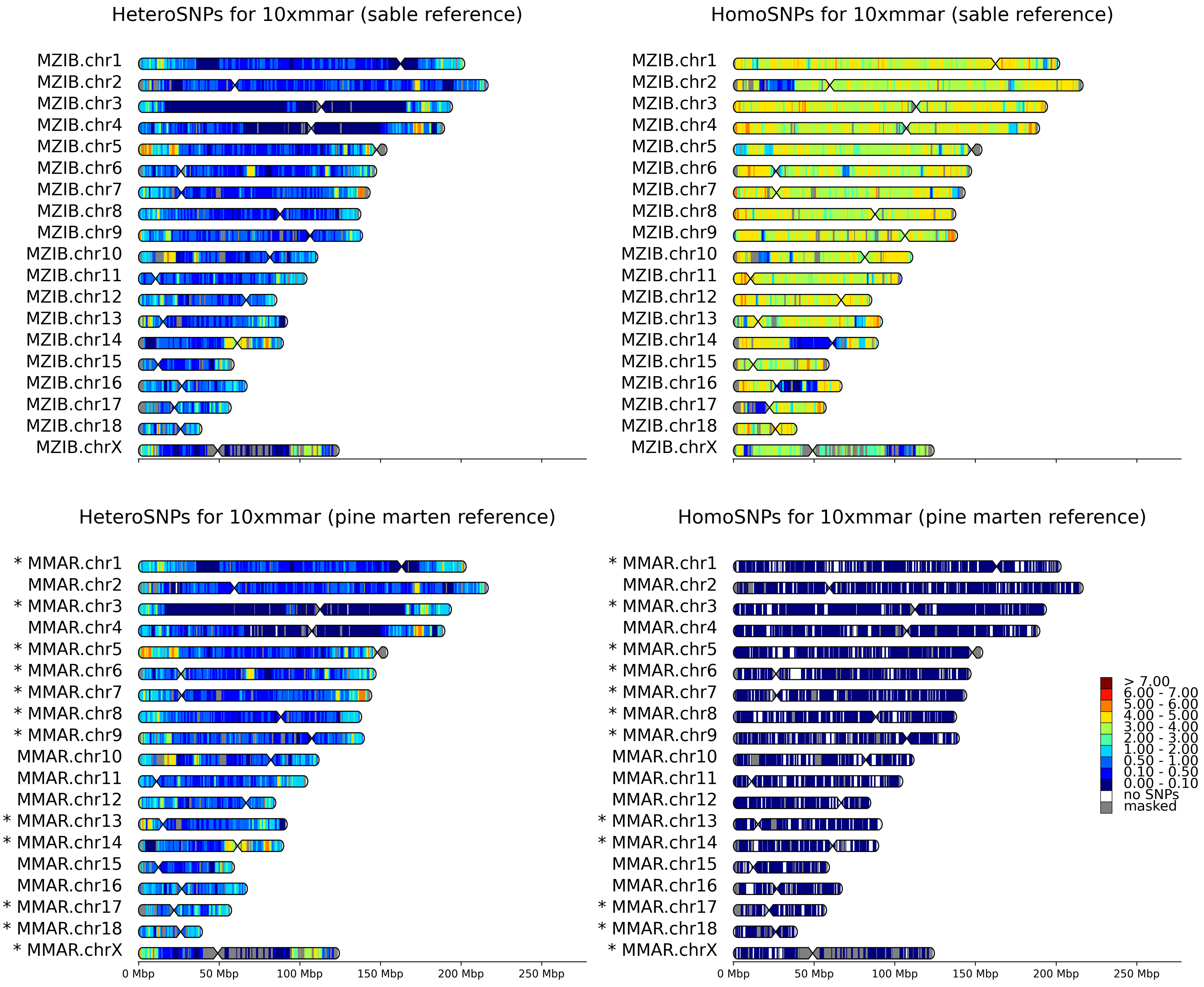
HeteroSNPs for 10xmmar (sable reference)
HomoSNPs for 10xmmar (sable reference)
MZIB.chr1
MZIB.chr2
MZIB.chr3
MZIB.chr4
MZIB.chr5
MZIB.chr6
MZIB.chr7
MZIB.chr8
MZIB.chr9
MZIB.chr10
MZIB.chr11
MZIB.chr12
MZIB.chr13
MZIB.chr14
MZIB.chr15
MZIB.chr16
MZIB.chr17
MZIB.chr18
MZIB.chrX
HeteroSNPs for 10xmmar (pine marten reference)
HomoSNPs for 10xmmar (pine marten reference)
* MMAR.chr1
MMAR.chr2
* MMAR.chr3
MMAR.chr4
* MMAR.chr5
* MMAR.chr6
* MMAR.chr7
* MMAR.chr8
* MMAR.chr9
MMAR.chr10
MMAR.chr11
MMAR.chr12
* MMAR.chr13
* MMAR.chr14
MMAR.chr15
MMAR.chr16
* MMAR.chr17
* MMAR.chr18
* MMAR.chrX
0 Mbp
50 Mbp
100 Mbp
150 Mbp
200 Mbp
250 Mbp
> 7.00
6.00 - 7.00
5.00 - 6.00
4.00 - 5.00
3.00 - 4.00
2.00 - 3.00
1.00 - 2.00
0.50 - 1.00
0.10 - 0.50
0.00 - 0.10
no SNPs
masked

S44

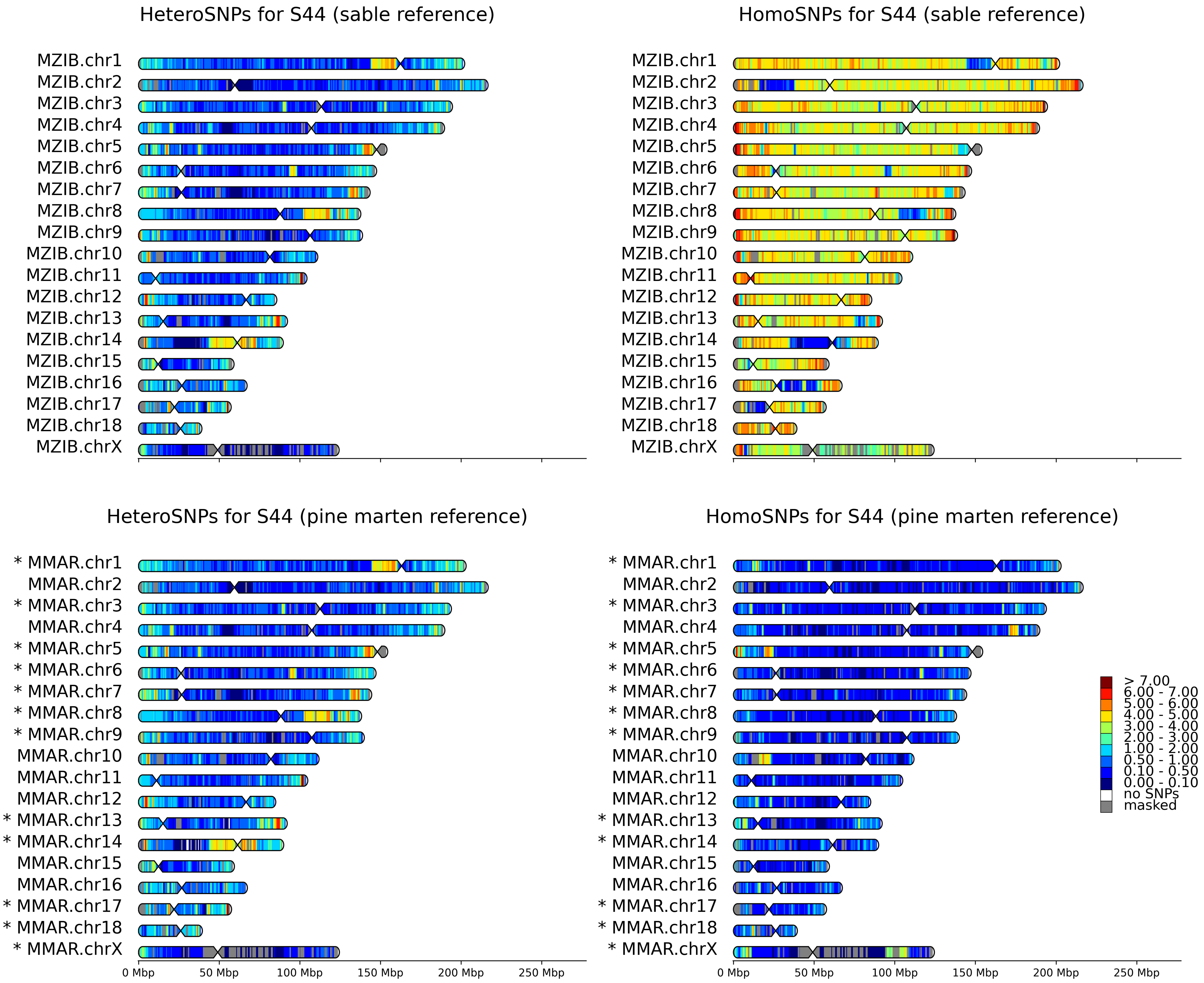
HeteroSNPs for S44 (sable reference)
HomoSNPs for S44 (sable reference)
MZIB.chr1
MZIB.chr2
MZIB.chr3
MZIB.chr4
MZIB.chr5
MZIB.chr6
MZIB.chr7
MZIB.chr8
MZIB.chr9
MZIB.chr10
MZIB.chr11
MZIB.chr12
MZIB.chr13
MZIB.chr14
MZIB.chr15
MZIB.chr16
MZIB.chr17
MZIB.chr18
MZIB.chrX
HeteroSNPs for S44 (pine marten reference)
HomoSNPs for S44 (pine marten reference)
* MMAR.chr1
MMAR.chr2
* MMAR.chr3
MMAR.chr4
* MMAR.chr5
* MMAR.chr6
* MMAR.chr7
* MMAR.chr8
* MMAR.chr9
MMAR.chr10
MMAR.chr11
MMAR.chr12
* MMAR.chr13
* MMAR.chr14
MMAR.chr15
MMAR.chr16
* MMAR.chr17
* MMAR.chr18
* MMAR.chrX
0 Mbp
50 Mbp
100 Mbp
150 Mbp
200 Mbp
250 Mbp
> 7.00
6.00 - 7.00
5.00 - 6.00
4.00 - 5.00
3.00 - 4.00
2.00 - 3.00
1.00 - 2.00
0.50 - 1.00
0.10 - 0.50
0.00 - 0.10
no SNPs
masked

S46

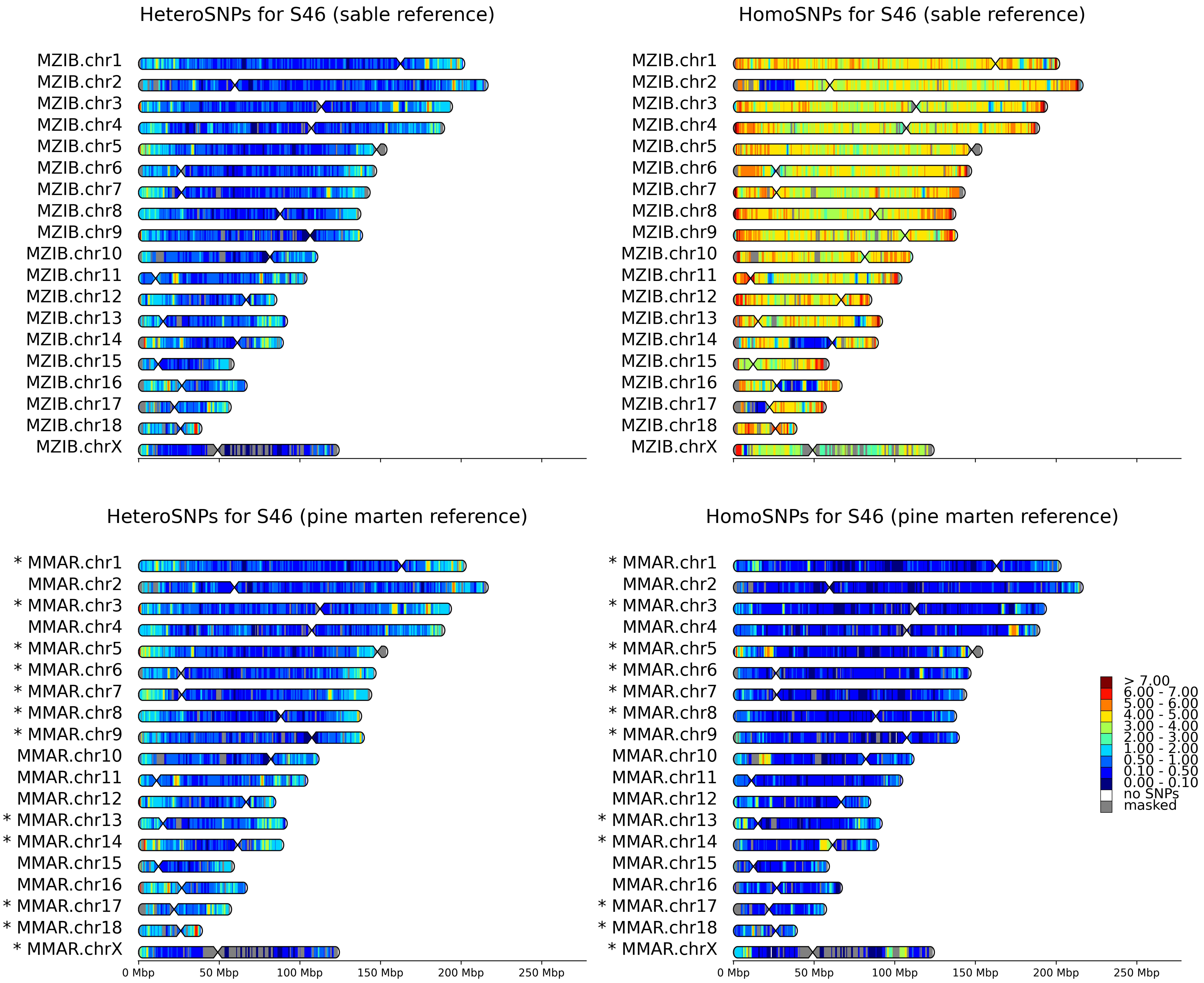

HeteroSNPs for S46 (sable reference)
HomoSNPs for S46 (sable reference)
MZIB.chr1
MZIB.chr2
MZIB.chr3
MZIB.chr4
MZIB.chr5
MZIB.chr6
MZIB.chr7
MZIB.chr8
MZIB.chr9
MZIB.chr10
MZIB.chr11
MZIB.chr12
MZIB.chr13
MZIB.chr14
MZIB.chr15
MZIB.chr16
MZIB.chr17
MZIB.chr18
MZIB.chrX
HeteroSNPs for S46 (pine marten reference)
HomoSNPs for S46 (pine marten reference)
* MMAR.chr1
MMAR.chr2
* MMAR.chr3
MMAR.chr4
* MMAR.chr5
* MMAR.chr6
* MMAR.chr7
* MMAR.chr8
* MMAR.chr9
MMAR.chr10
MMAR.chr11
MMAR.chr12
* MMAR.chr13
* MMAR.chr14
MMAR.chr15
MMAR.chr16
* MMAR.chr17
* MMAR.chr18
* MMAR.chrX
0 Mbp
50 Mbp
100 Mbp
150 Mbp
200 Mbp
250 Mbp
> 7.00
6.00 - 7.00
5.00 - 6.00
4.00 - 5.00
3.00 - 4.00
2.00 - 3.00
1.00 - 2.00
0.50 - 1.00
0.10 - 0.50
0.00 - 0.10
no SNPs
masked

S49

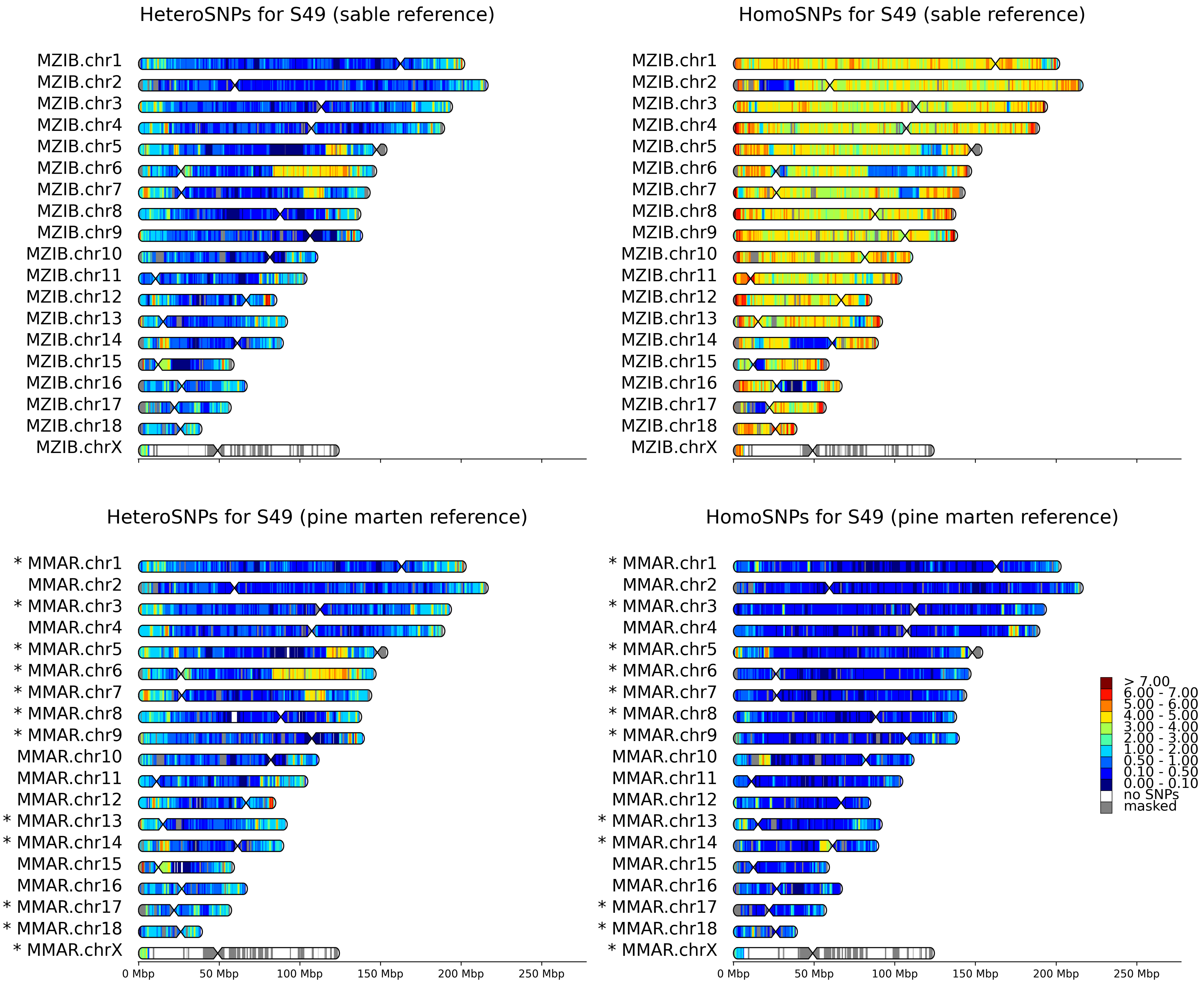
HeteroSNPs for S49 (sable reference)
HomoSNPs for S49 (sable reference)
MZIB.chr1
MZIB.chr2
MZIB.chr3
MZIB.chr4
MZIB.chr5
MZIB.chr6
MZIB.chr7
MZIB.chr8
MZIB.chr9
MZIB.chr10
MZIB.chr11
MZIB.chr12
MZIB.chr13
MZIB.chr14
MZIB.chr15
MZIB.chr16
MZIB.chr17
MZIB.chr18
MZIB.chrX
HeteroSNPs for S49 (pine marten reference)
HomoSNPs for S49 (pine marten reference)
* MMAR.chr1
MMAR.chr2
* MMAR.chr3
MMAR.chr4
* MMAR.chr5
* MMAR.chr6
* MMAR.chr7
* MMAR.chr8
* MMAR.chr9
MMAR.chr10
MMAR.chr11
MMAR.chr12
* MMAR.chr13
* MMAR.chr14
MMAR.chr15
MMAR.chr16
* MMAR.chr17
* MMAR.chr18
* MMAR.chrX
0 Mbp
50 Mbp
100 Mbp
150 Mbp
200 Mbp
250 Mbp
> 7.00
6.00 - 7.00
5.00 - 6.00
4.00 - 5.00
3.00 - 4.00
2.00 - 3.00
1.00 - 2.00
0.50 - 1.00
0.10 - 0.50
0.00 - 0.10
no SNPs
masked

S50

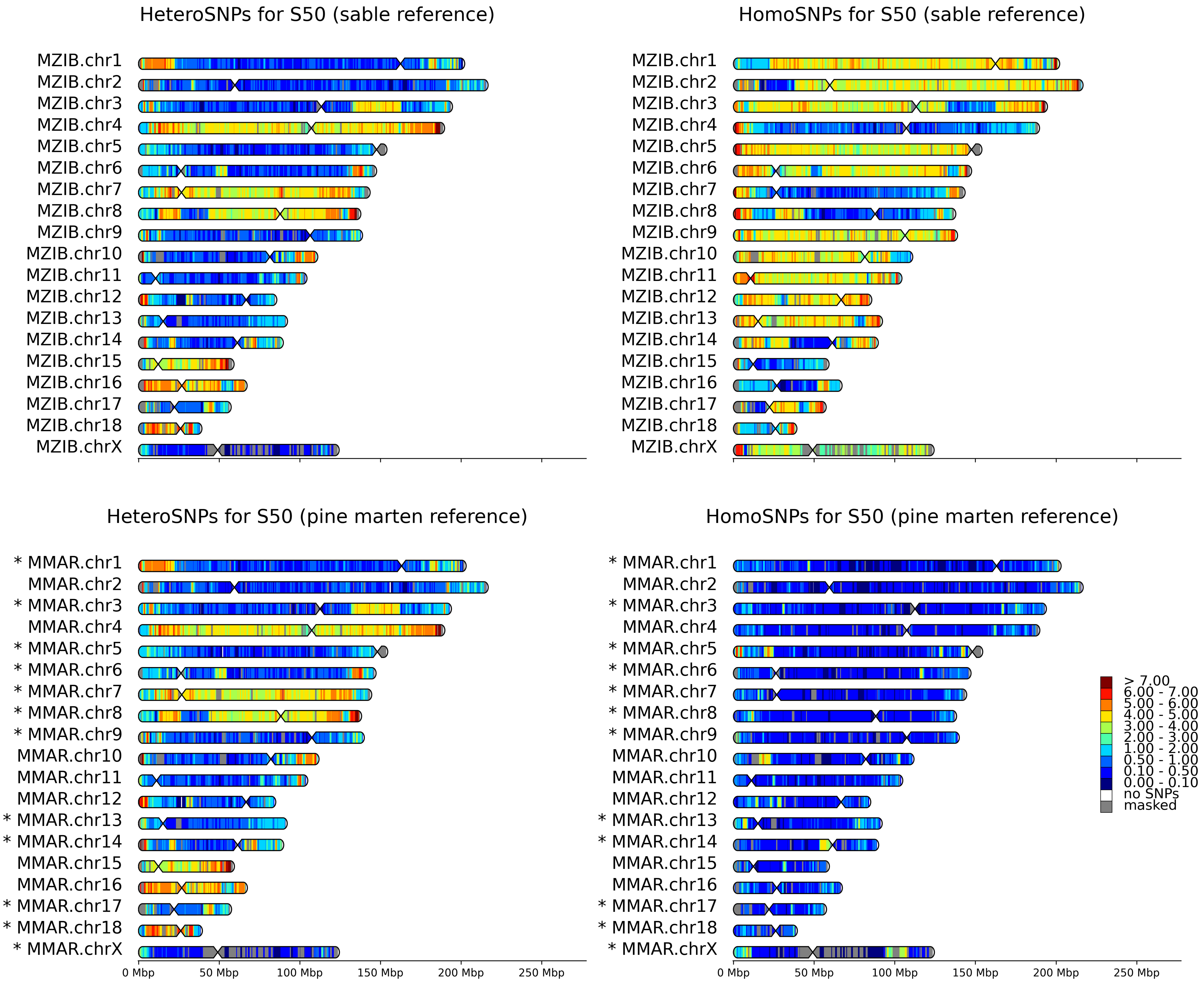

HeteroSNPs for S50 (sable reference)
HomoSNPs for S50 (sable reference)
MZIB.chr1
MZIB.chr2
MZIB.chr3
MZIB.chr4
MZIB.chr5
MZIB.chr6
MZIB.chr7
MZIB.chr8
MZIB.chr9
MZIB.chr10
MZIB.chr11
MZIB.chr12
MZIB.chr13
MZIB.chr14
MZIB.chr15
MZIB.chr16
MZIB.chr17
MZIB.chr18
MZIB.chrX
HeteroSNPs for S50 (pine marten reference)
HomoSNPs for S50 (pine marten reference)
* MMAR.chr1
MMAR.chr2
* MMAR.chr3
MMAR.chr4
* MMAR.chr5
* MMAR.chr6
* MMAR.chr7
* MMAR.chr8
* MMAR.chr9
MMAR.chr10
MMAR.chr11
MMAR.chr12
* MMAR.chr13
* MMAR.chr14
MMAR.chr15
MMAR.chr16
* MMAR.chr17
* MMAR.chr18
* MMAR.chrX
0 Mbp
50 Mbp
100 Mbp
150 Mbp
200 Mbp
250 Mbp
> 7.00
6.00 - 7.00
5.00 - 6.00
4.00 - 5.00
3.00 - 4.00
2.00 - 3.00
1.00 - 2.00
0.50 - 1.00
0.10 - 0.50
0.00 - 0.10
no SNPs
masked

T149

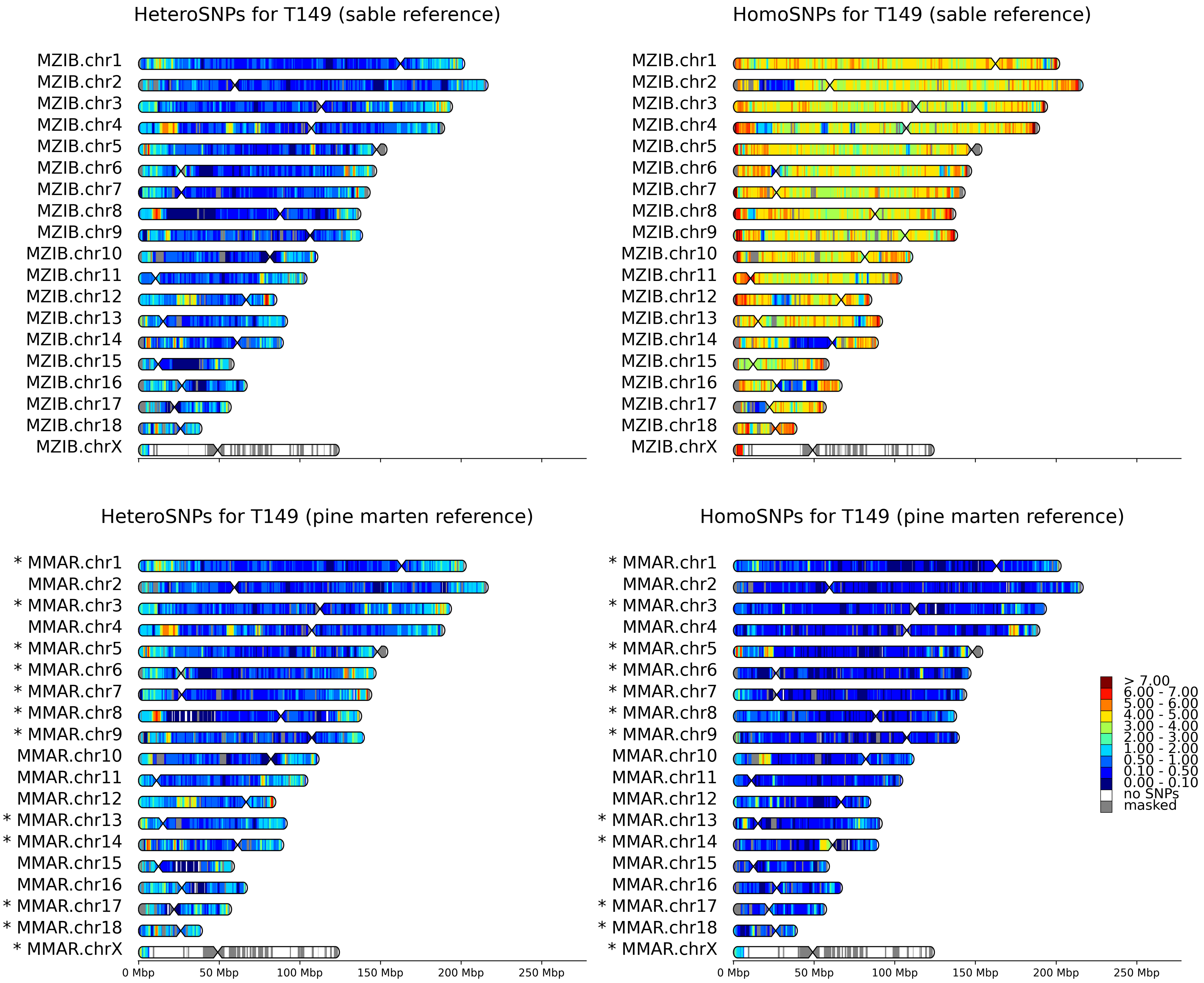
HeteroSNPs for T149 (sable reference)
HomoSNPs for T149 (sable reference)
HeteroSNPs for T149 (pine marten reference)
HomoSNPs for T149 (pine marten reference)
MZIB.chr1
MZIB.chr2
MZIB.chr3
MZIB.chr4
MZIB.chr5
MZIB.chr6
MZIB.chr7
MZIB.chr8
MZIB.chr9
MZIB.chr10
MZIB.chr11
MZIB.chr12
MZIB.chr13
MZIB.chr14
MZIB.chr15
MZIB.chr16
MZIB.chr17
MZIB.chr18
MZIB.chrX
* MMAR.chr1
MMAR.chr2
* MMAR.chr3
MMAR.chr4
* MMAR.chr5
* MMAR.chr6
* MMAR.chr7
* MMAR.chr8
* MMAR.chr9
MMAR.chr10
MMAR.chr11
MMAR.chr12
* MMAR.chr13
* MMAR.chr14
MMAR.chr15
MMAR.chr16
* MMAR.chr17
* MMAR.chr18
* MMAR.chrX
0 Mbp
50 Mbp
100 Mbp
150 Mbp
200 Mbp
250 Mbp
> 7.00
6.00 - 7.00
5.00 - 6.00
4.00 - 5.00
3.00 - 4.00
2.00 - 3.00
1.00 - 2.00
0.50 - 1.00
0.10 - 0.50
0.00 - 0.10
no SNPs
masked

T151

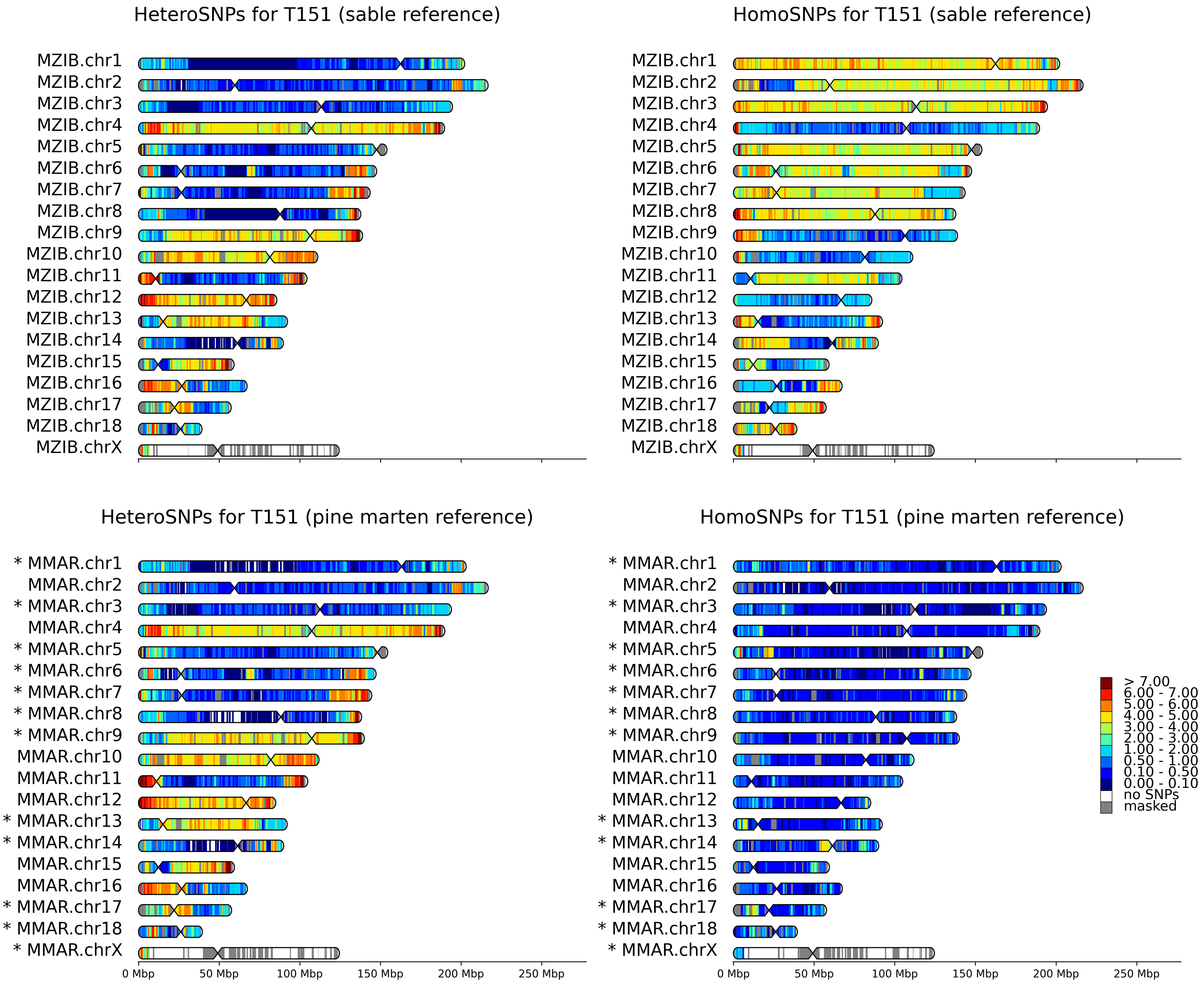

HeteroSNPs for T151 (sable reference)
HomoSNPs for T151 (sable reference)
MZIB.chr1
MZIB.chr2
MZIB.chr3
MZIB.chr4
MZIB.chr5
MZIB.chr6
MZIB.chr7
MZIB.chr8
MZIB.chr9
MZIB.chr10
MZIB.chr11
MZIB.chr12
MZIB.chr13
MZIB.chr14
MZIB.chr15
MZIB.chr16
MZIB.chr17
MZIB.chr18
MZIB.chrX
HeteroSNPs for T151 (pine marten reference)
HomoSNPs for T151 (pine marten reference)
* MMAR.chr1
MMAR.chr2
* MMAR.chr3
MMAR.chr4
* MMAR.chr5
* MMAR.chr6
* MMAR.chr7
* MMAR.chr8
* MMAR.chr9
MMAR.chr10
MMAR.chr11
MMAR.chr12
* MMAR.chr13
* MMAR.chr14
MMAR.chr15
MMAR.chr16
* MMAR.chr17
* MMAR.chr18
* MMAR.chrX
0 Mbp
50 Mbp
100 Mbp
150 Mbp
200 Mbp
250 Mbp
> 7.00
6.00 - 7.00
5.00 - 6.00
4.00 - 5.00
3.00 - 4.00
2.00 - 3.00
1.00 - 2.00
0.50 - 1.00
0.10 - 0.50
0.00 - 0.10
no SNPs
masked

T24

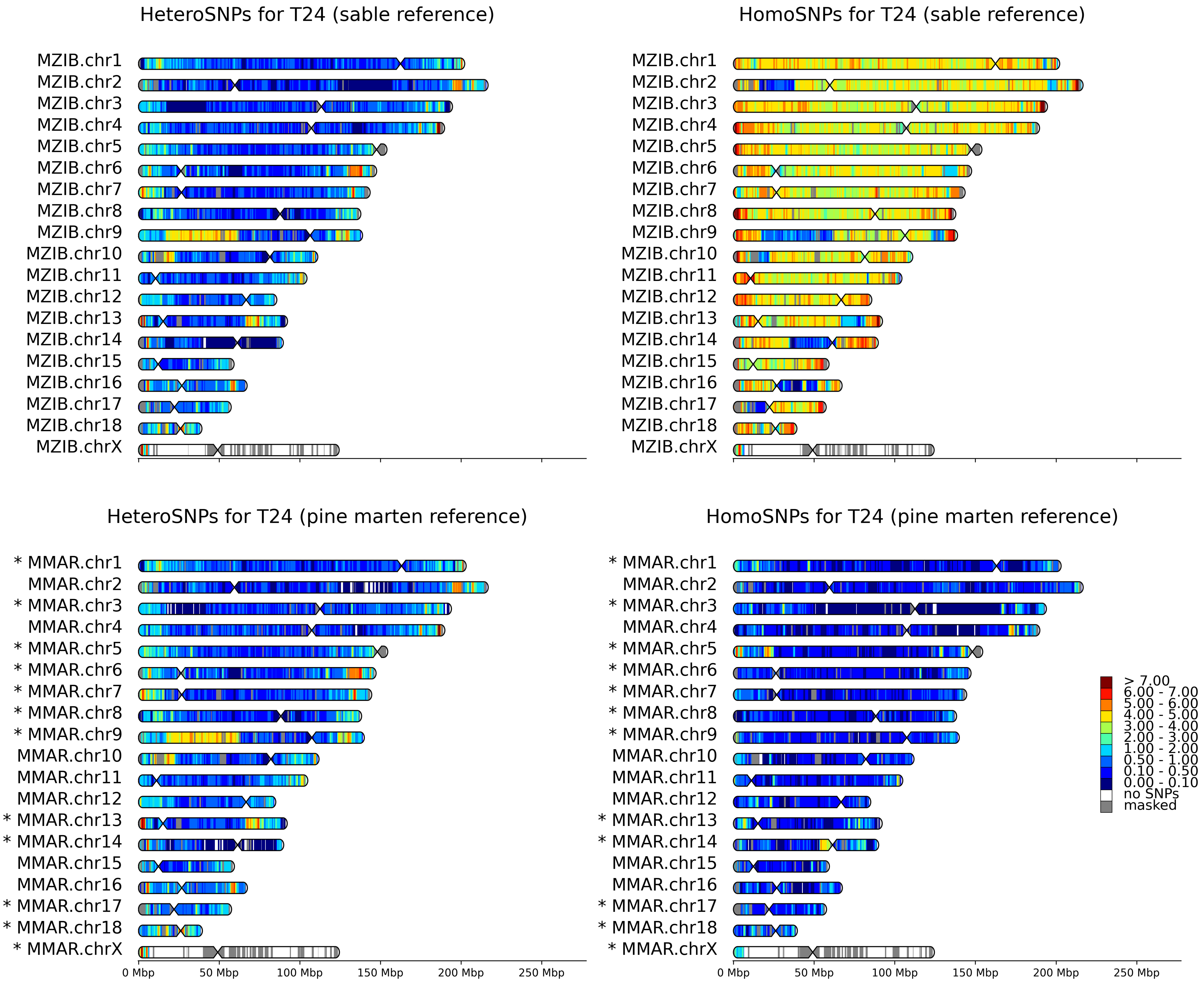
HeteroSNPs for T24 (sable reference)
HomoSNPs for T24 (sable reference)
MZIB.chr1
MZIB.chr2
MZIB.chr3
MZIB.chr4
MZIB.chr5
MZIB.chr6
MZIB.chr7
MZIB.chr8
MZIB.chr9
MZIB.chr10
MZIB.chr11
MZIB.chr12
MZIB.chr13
MZIB.chr14
MZIB.chr15
MZIB.chr16
MZIB.chr17
MZIB.chr18
MZIB.chrX
HeteroSNPs for T24 (pine marten reference)
HomoSNPs for T24 (pine marten reference)
* MMAR.chr1
MMAR.chr2
* MMAR.chr3
MMAR.chr4
* MMAR.chr5
* MMAR.chr6
* MMAR.chr7
* MMAR.chr8
* MMAR.chr9
MMAR.chr10
MMAR.chr11
MMAR.chr12
* MMAR.chr13
* MMAR.chr14
MMAR.chr15
MMAR.chr16
* MMAR.chr17
* MMAR.chr18
* MMAR.chrX
0 Mbp
50 Mbp
100 Mbp
150 Mbp
200 Mbp
250 Mbp
> 7.00
6.00 - 7.00
5.00 - 6.00
4.00 - 5.00
3.00 - 4.00
2.00 - 3.00
1.00 - 2.00
0.50 - 1.00
0.10 - 0.50
0.00 - 0.10
no SNPs
masked

T76

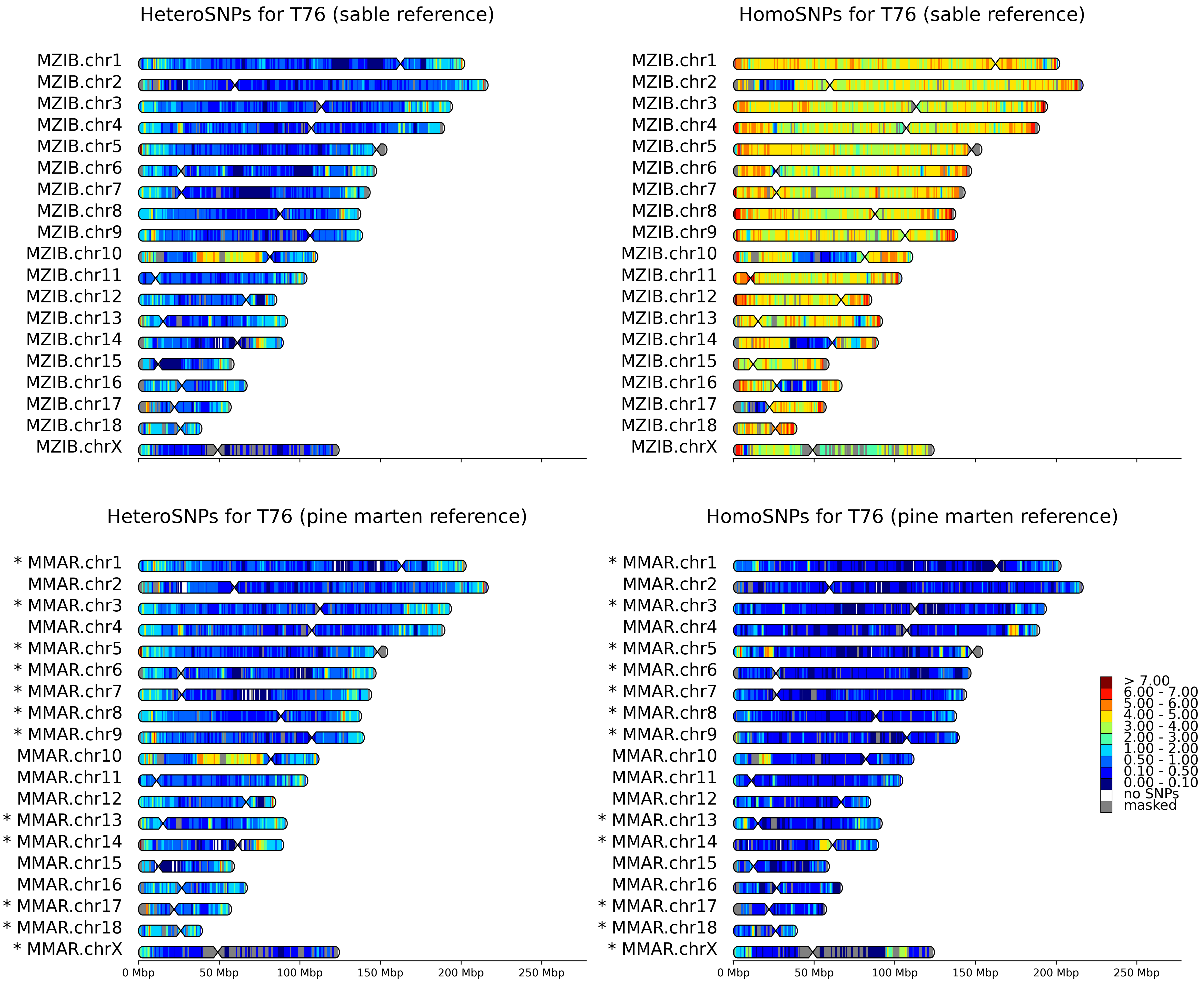
HeteroSNPs for T76 (sable reference)
HomoSNPs for T76 (sable reference)
MZIB.chr1
MZIB.chr2
MZIB.chr3
MZIB.chr4
MZIB.chr5
MZIB.chr6
MZIB.chr7
MZIB.chr8
MZIB.chr9
MZIB.chr10
MZIB.chr11
MZIB.chr12
MZIB.chr13
MZIB.chr14
MZIB.chr15
MZIB.chr16
MZIB.chr17
MZIB.chr18
MZIB.chrX
HeteroSNPs for T76 (pine marten reference)
HomoSNPs for T76 (pine marten reference)
* MMAR.chr1
MMAR.chr2
* MMAR.chr3
MMAR.chr4
* MMAR.chr5
* MMAR.chr6
* MMAR.chr7
* MMAR.chr8
* MMAR.chr9
MMAR.chr10
MMAR.chr11
MMAR.chr12
* MMAR.chr13
* MMAR.chr14
MMAR.chr15
MMAR.chr16
* MMAR.chr17
* MMAR.chr18
* MMAR.chrX
0 Mbp
50 Mbp
100 Mbp
150 Mbp
200 Mbp
250 Mbp
> 7.00
6.00 - 7.00
5.00 - 6.00
4.00 - 5.00
3.00 - 4.00
2.00 - 3.00
1.00 - 2.00
0.50 - 1.00
0.10 - 0.50
0.00 - 0.10
no SNPs
masked

T77

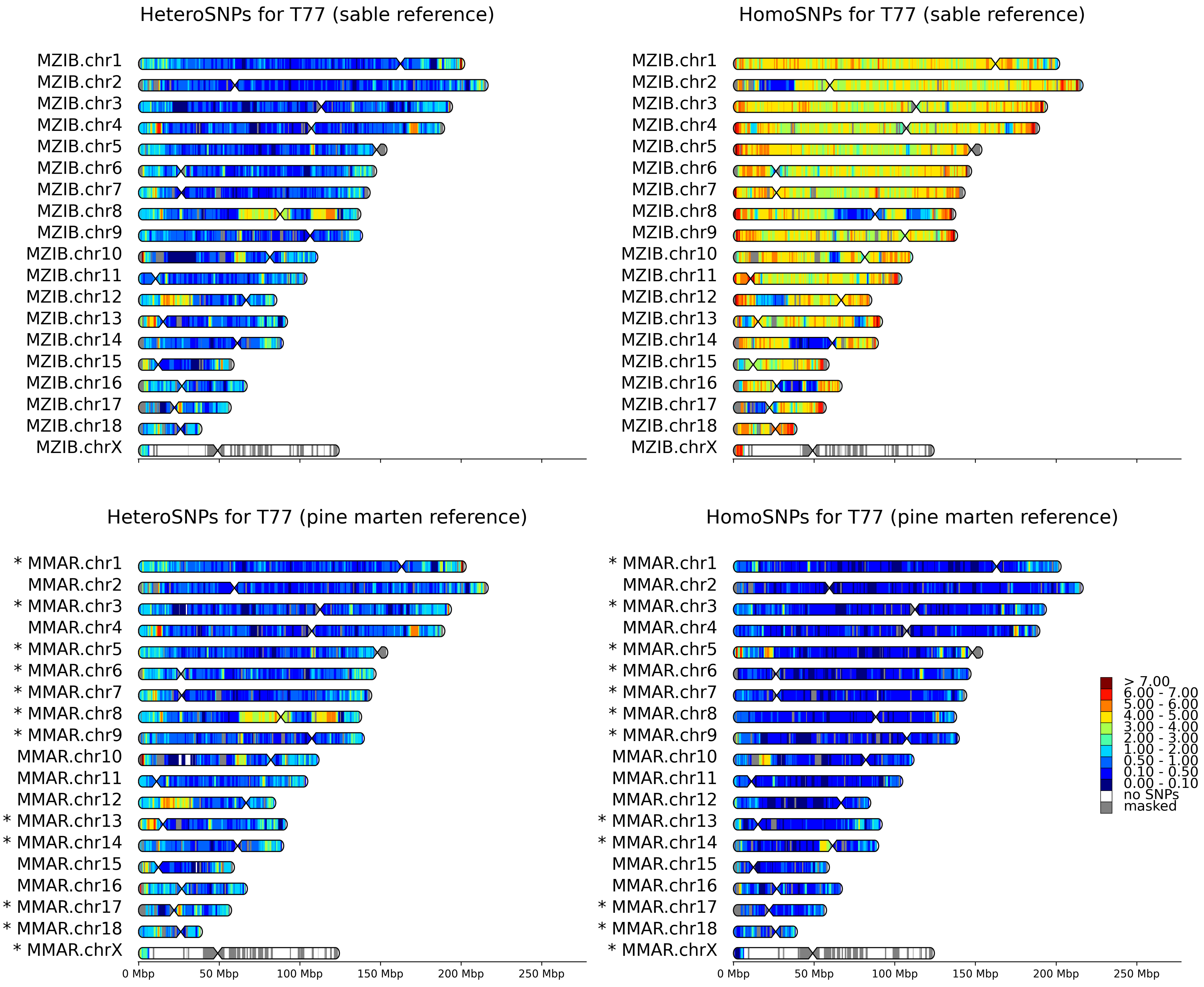
HeteroSNPs for T77 (sable reference)
HomoSNPs for T77 (sable reference)
MZIB.chr1
MZIB.chr2
MZIB.chr3
MZIB.chr4
MZIB.chr5
MZIB.chr6
MZIB.chr7
MZIB.chr8
MZIB.chr9
MZIB.chr10
MZIB.chr11
MZIB.chr12
MZIB.chr13
MZIB.chr14
MZIB.chr15
MZIB.chr16
MZIB.chr17
MZIB.chr18
MZIB.chrX
HeteroSNPs for T77 (pine marten reference)
HomoSNPs for T77 (pine marten reference)
* MMAR.chr1
MMAR.chr2
* MMAR.chr3
MMAR.chr4
* MMAR.chr5
* MMAR.chr6
* MMAR.chr7
* MMAR.chr8
* MMAR.chr9
MMAR.chr10
MMAR.chr11
MMAR.chr12
* MMAR.chr13
* MMAR.chr14
MMAR.chr15
MMAR.chr16
* MMAR.chr17
* MMAR.chr18
* MMAR.chrX
0 Mbp
50 Mbp
100 Mbp
150 Mbp
200 Mbp
250 Mbp
> 7.00
6.00 - 7.00
5.00 - 6.00
4.00 - 5.00
3.00 - 4.00
2.00 - 3.00
1.00 - 2.00
0.50 - 1.00
0.10 - 0.50
0.00 - 0.10
no SNPs
masked

T78

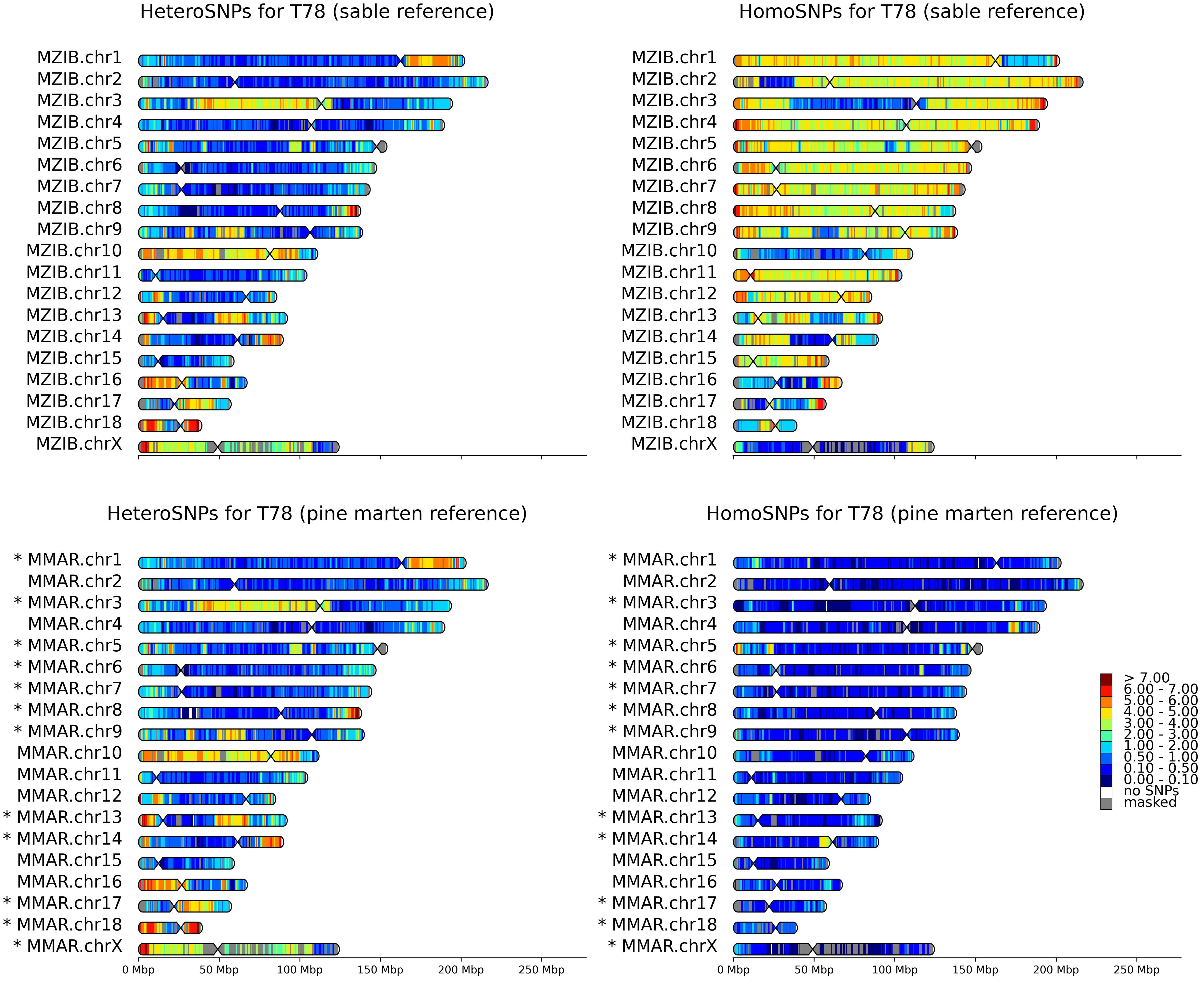
HeteroSNPs for T78 (sable reference)
HomoSNPs for T78 (sable reference)
HeteroSNPs for T78 (pine marten reference)
HomoSNPs for T78 (pine marten reference)
MZIB.chr1
MZIB.chr2
MZIB.chr3
MZIB.chr4
MZIB.chr5
MZIB.chr6
MZIB.chr7
MZIB.chr8
MZIB.chr9
MZIB.chr10
MZIB.chr11
MZIB.chr12
MZIB.chr13
MZIB.chr14
MZIB.chr15
MZIB.chr16
MZIB.chr17
MZIB.chr18
MZIB.chrX
* MMAR.chr1
MMAR.chr2
* MMAR.chr3
MMAR.chr4
* MMAR.chr5
* MMAR.chr6
* MMAR.chr7
* MMAR.chr8
* MMAR.chr9
MMAR.chr10
MMAR.chr11
MMAR.chr12
* MMAR.chr13
* MMAR.chr14
MMAR.chr15
MMAR.chr16
* MMAR.chr17
* MMAR.chr18
* MMAR.chrX
0 Mbp
50 Mbp
100 Mbp
150 Mbp
200 Mbp
250 Mbp
> 7.00
6.00 - 7.00
5.00 - 6.00
4.00 - 5.00
3.00 - 4.00
2.00 - 3.00
1.00 - 2.00
0.50 - 1.00
0.10 - 0.50
0.00 - 0.10
no SNPs
masked

T79

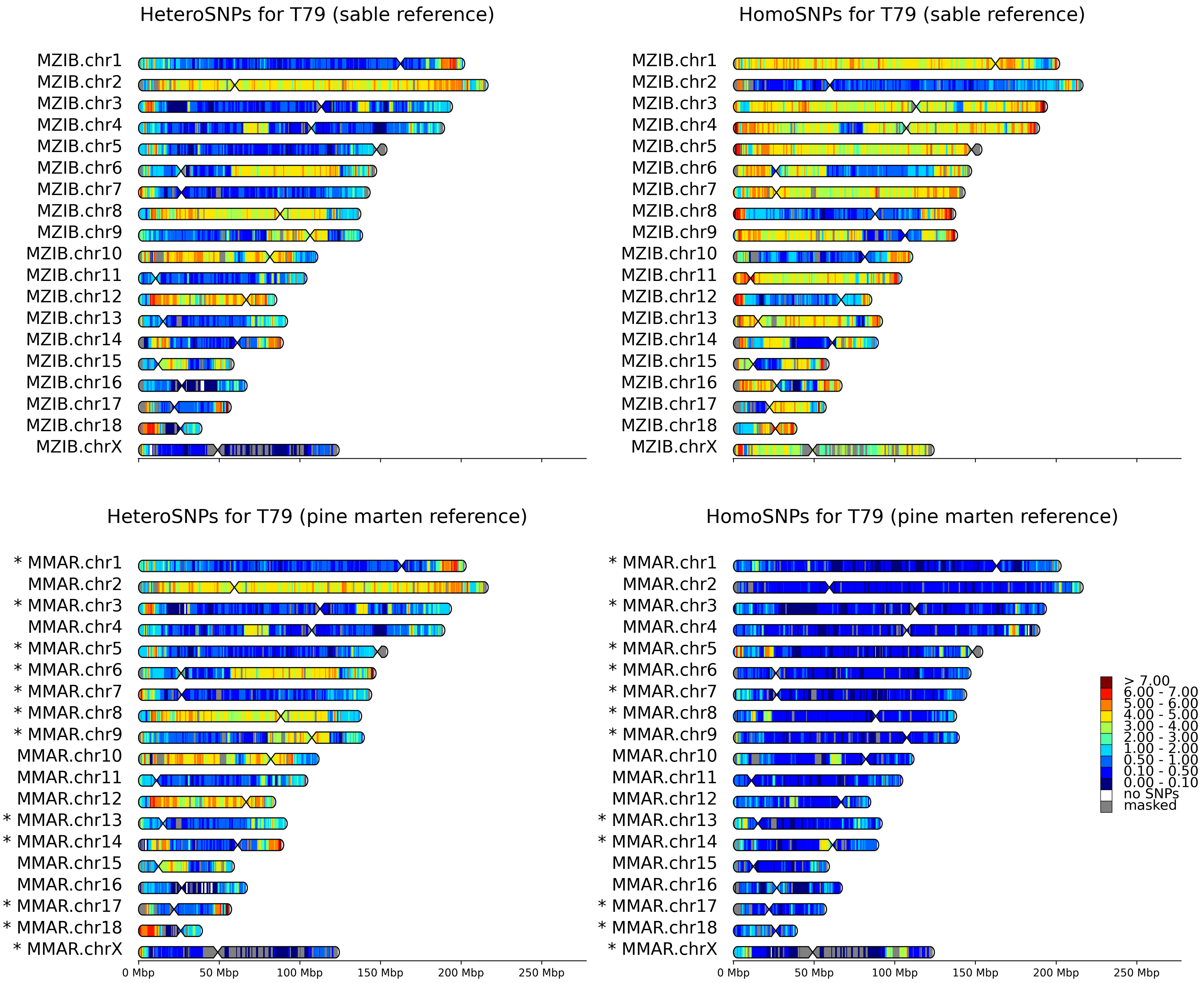
HeteroSNPs for T79 (sable reference)
HomoSNPs for T79 (sable reference)
MZIB.chr1
MZIB.chr2
MZIB.chr3
MZIB.chr4
MZIB.chr5
MZIB.chr6
MZIB.chr7
MZIB.chr8
MZIB.chr9
MZIB.chr10
MZIB.chr11
MZIB.chr12
MZIB.chr13
MZIB.chr14
MZIB.chr15
MZIB.chr16
MZIB.chr17
MZIB.chr18
MZIB.chrX
HeteroSNPs for T79 (pine marten reference)
HomoSNPs for T79 (pine marten reference)
* MMAR.chr1
MMAR.chr2
* MMAR.chr3
MMAR.chr4
* MMAR.chr5
* MMAR.chr6
* MMAR.chr7
* MMAR.chr8
* MMAR.chr9
MMAR.chr10
MMAR.chr11
MMAR.chr12
* MMAR.chr13
* MMAR.chr14
MMAR.chr15
MMAR.chr16
* MMAR.chr17
* MMAR.chr18
* MMAR.chrX
0 Mbp
50 Mbp
100 Mbp
150 Mbp
200 Mbp
250 Mbp
> 7.00
6.00 - 7.00
5.00 - 6.00
4.00 - 5.00
3.00 - 4.00
2.00 - 3.00
1.00 - 2.00
0.50 - 1.00
0.10 - 0.50
0.00 - 0.10
no SNPs
masked

T81

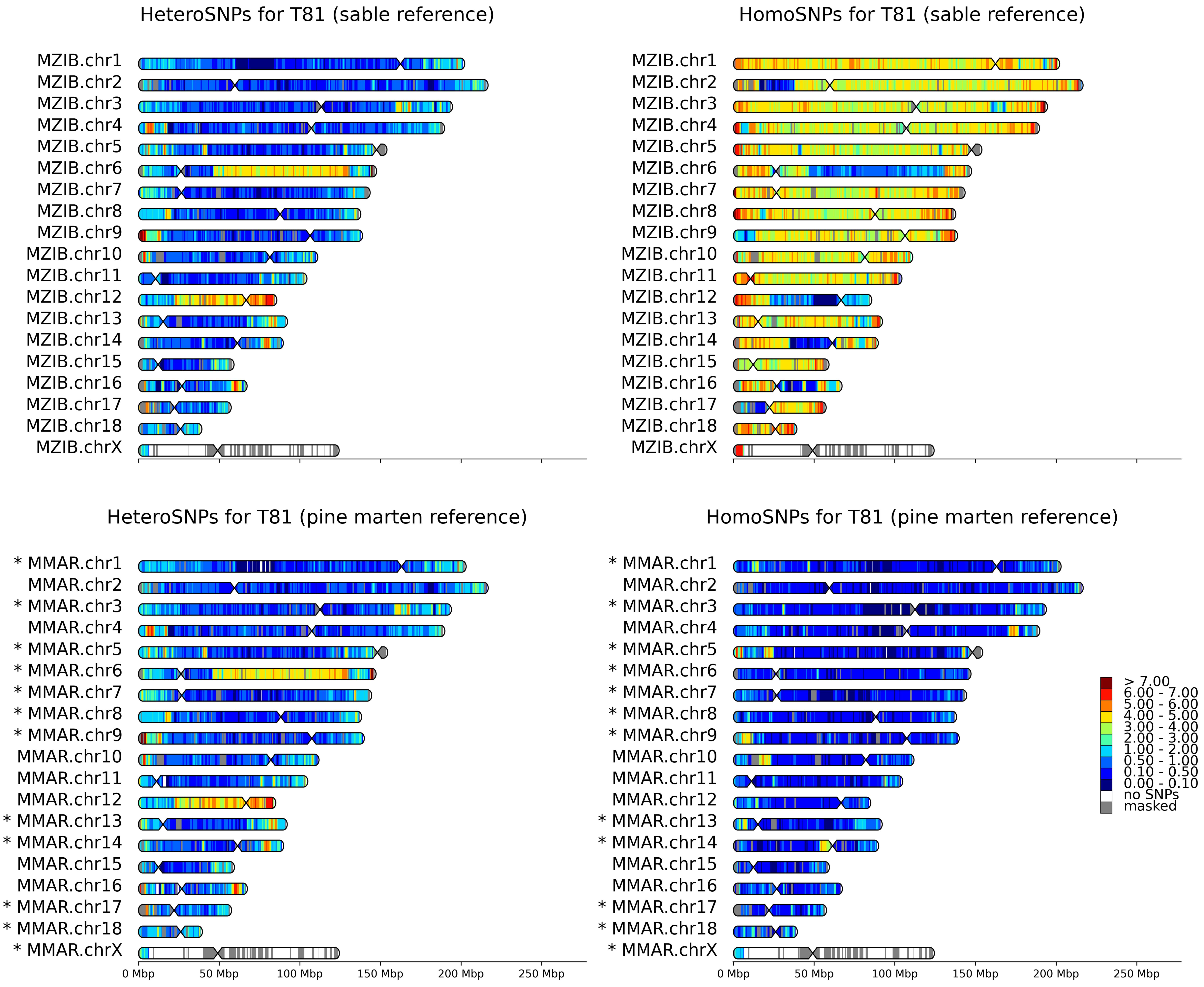
HeteroSNPs for T81 (sable reference)
HomoSNPs for T81 (sable reference)
MZIB.chr1
MZIB.chr2
MZIB.chr3
MZIB.chr4
MZIB.chr5
MZIB.chr6
MZIB.chr7
MZIB.chr8
MZIB.chr9
MZIB.chr10
MZIB.chr11
MZIB.chr12
MZIB.chr13
MZIB.chr14
MZIB.chr15
MZIB.chr16
MZIB.chr17
MZIB.chr18
MZIB.chrX
HeteroSNPs for T81 (pine marten reference)
HomoSNPs for T81 (pine marten reference)
* MMAR.chr1
MMAR.chr2
* MMAR.chr3
MMAR.chr4
* MMAR.chr5
* MMAR.chr6
* MMAR.chr7
* MMAR.chr8
* MMAR.chr9
MMAR.chr10
MMAR.chr11
MMAR.chr12
* MMAR.chr13
* MMAR.chr14
MMAR.chr15
MMAR.chr16
* MMAR.chr17
* MMAR.chr18
* MMAR.chrX
0 Mbp
50 Mbp
100 Mbp
150 Mbp
200 Mbp
250 Mbp
> 7.00
6.00 - 7.00
5.00 - 6.00
4.00 - 5.00
3.00 - 4.00
2.00 - 3.00
1.00 - 2.00
0.50 - 1.00
0.10 - 0.50
0.00 - 0.10
no SNPs
masked

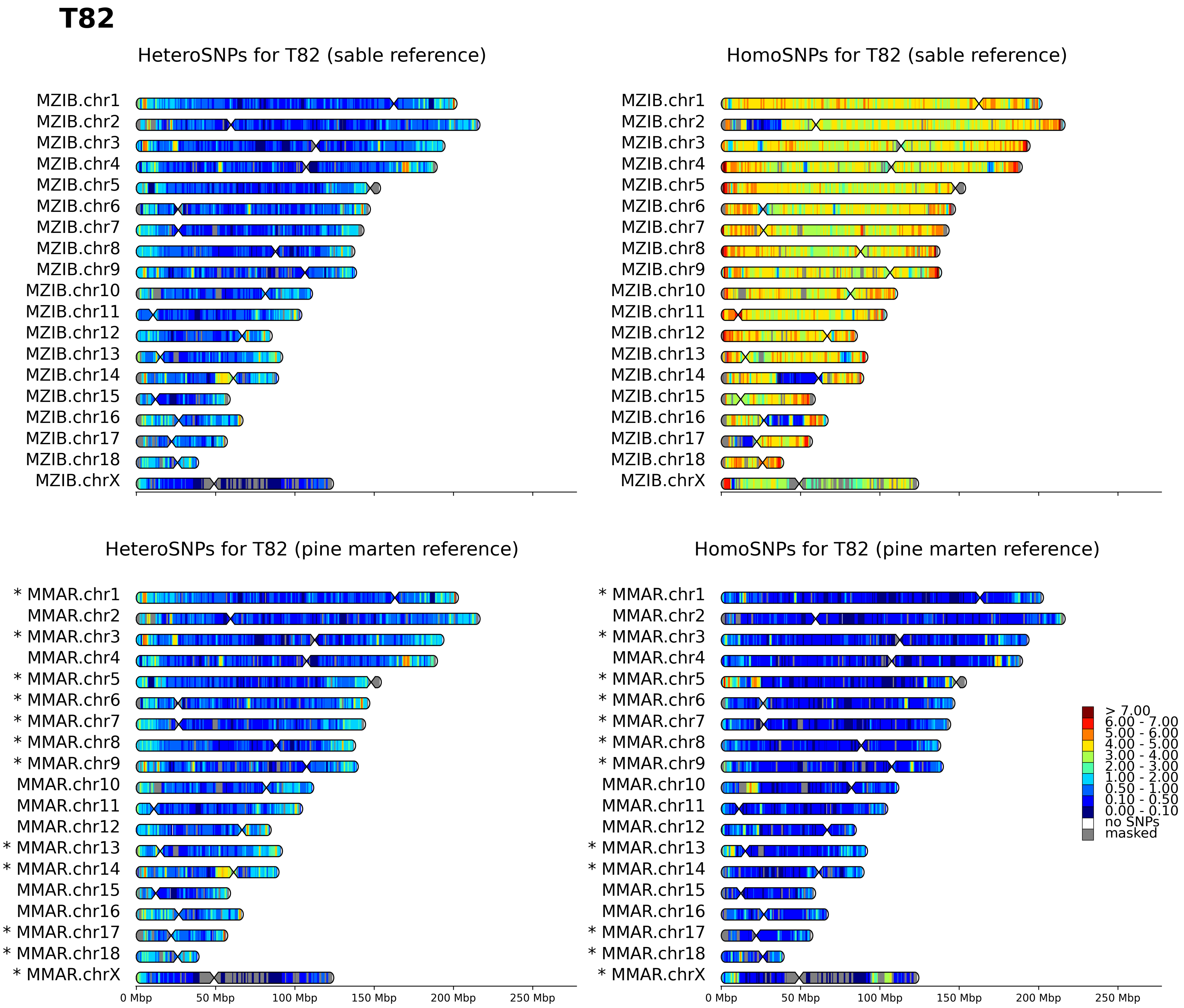
T82
HeteroSNPs for T82 (sable reference)
HomoSNPs for T82 (sable reference)
HeteroSNPs for T82 (pine marten reference)
HomoSNPs for T82 (pine marten reference)
MZIB.chr1
MZIB.chr2
MZIB.chr3
MZIB.chr4
MZIB.chr5
MZIB.chr6
MZIB.chr7
MZIB.chr8
MZIB.chr9
MZIB.chr10
MZIB.chr11
MZIB.chr12
MZIB.chr13
MZIB.chr14
MZIB.chr15
MZIB.chr16
MZIB.chr17
MZIB.chr18
MZIB.chrX
* MMAR.chr1
MMAR.chr2
* MMAR.chr3
MMAR.chr4
* MMAR.chr5
* MMAR.chr6
* MMAR.chr7
* MMAR.chr8
* MMAR.chr9
MMAR.chr10
MMAR.chr11
MMAR.chr12
* MMAR.chr13
* MMAR.chr14
MMAR.chr15
MMAR.chr16
* MMAR.chr17
* MMAR.chr18
* MMAR.chrX
0 Mbp
50 Mbp
100 Mbp
150 Mbp
200 Mbp
250 Mbp
> 7.00
6.00 - 7.00
5.00 - 6.00
4.00 - 5.00
3.00 - 4.00
2.00 - 3.00
1.00 - 2.00
0.50 - 1.00
0.10 - 0.50
0.00 - 0.10
no SNPs
masked

T83

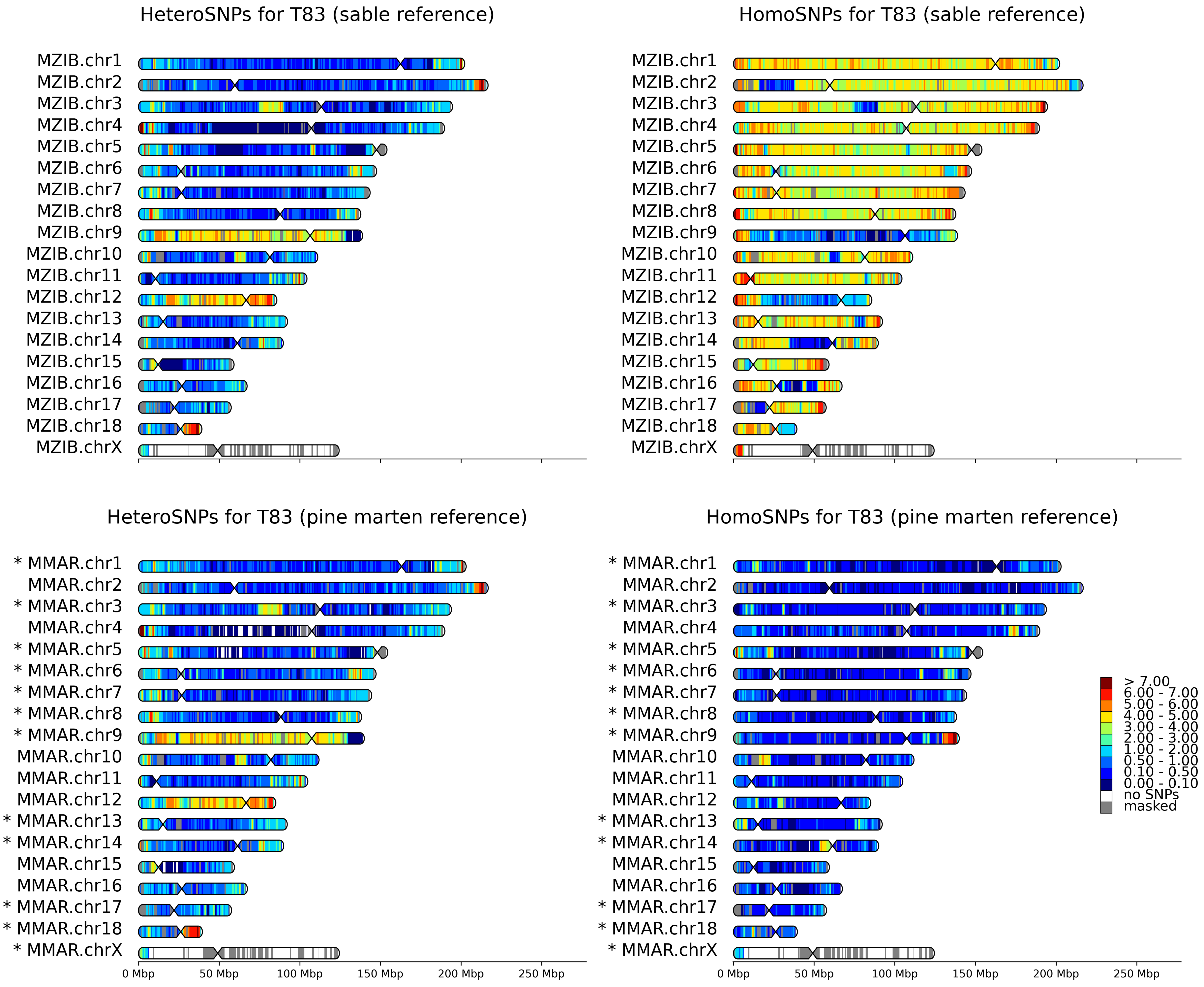
HeteroSNPs for T83 (sable reference)
MZIB.chr1
MZIB.chr2
MZIB.chr3
MZIB.chr4
MZIB.chr5
MZIB.chr6
MZIB.chr7
MZIB.chr8
MZIB.chr9
MZIB.chr10
MZIB.chr11
MZIB.chr12
MZIB.chr13
MZIB.chr14
MZIB.chr15
MZIB.chr16
MZIB.chr17
MZIB.chr18
MZIB.chrX
HomoSNPs for T83 (sable reference)
HeteroSNPs for T83 (pine marten reference)
* MMAR.chr1
MMAR.chr2
* MMAR.chr3
MMAR.chr4
* MMAR.chr5
* MMAR.chr6
* MMAR.chr7
* MMAR.chr8
* MMAR.chr9
MMAR.chr10
MMAR.chr11
MMAR.chr12
* MMAR.chr13
* MMAR.chr14
MMAR.chr15
MMAR.chr16
* MMAR.chr17
* MMAR.chr18
* MMAR.chrX
HomoSNPs for T83 (pine marten reference)
0 Mbp
50 Mbp
100 Mbp
150 Mbp
200 Mbp
250 Mbp
> 7.00
6.00 - 7.00
5.00 - 6.00
4.00 - 5.00
3.00 - 4.00
2.00 - 3.00
1.00 - 2.00
0.50 - 1.00
0.10 - 0.50
0.00 - 0.10
no SNPs
masked

T84

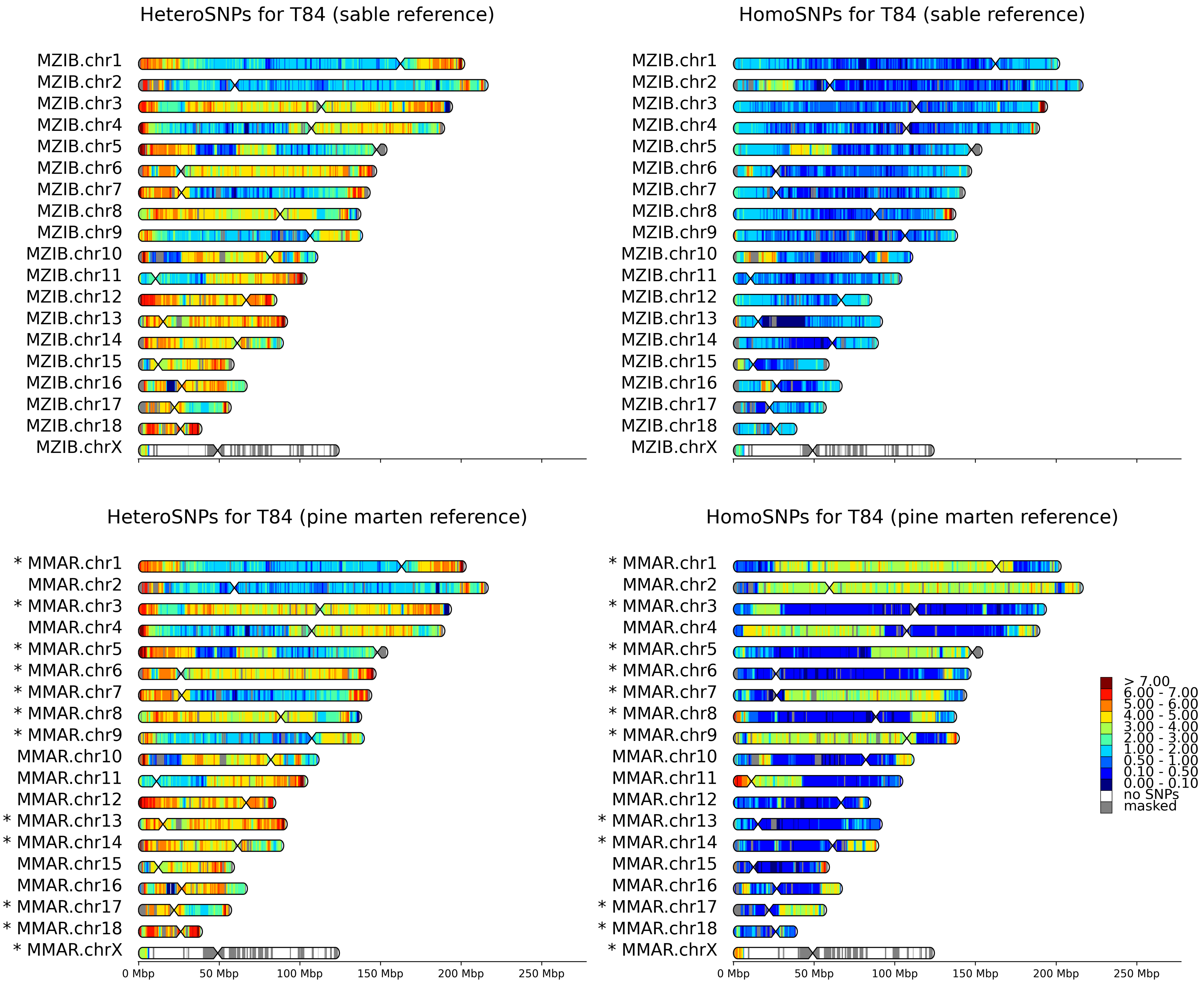
HeteroSNPs for T84 (sable reference)
HomoSNPs for T84 (sable reference)
MZIB.chr1
MZIB.chr2
MZIB.chr3
MZIB.chr4
MZIB.chr5
MZIB.chr6
MZIB.chr7
MZIB.chr8
MZIB.chr9
MZIB.chr10
MZIB.chr11
MZIB.chr12
MZIB.chr13
MZIB.chr14
MZIB.chr15
MZIB.chr16
MZIB.chr17
MZIB.chr18
MZIB.chrX
HeteroSNPs for T84 (pine marten reference)
HomoSNPs for T84 (pine marten reference)
* MMAR.chr1
MMAR.chr2
* MMAR.chr3
MMAR.chr4
* MMAR.chr5
* MMAR.chr6
* MMAR.chr7
* MMAR.chr8
* MMAR.chr9
MMAR.chr10
MMAR.chr11
MMAR.chr12
* MMAR.chr13
* MMAR.chr14
MMAR.chr15
MMAR.chr16
* MMAR.chr17
* MMAR.chr18
* MMAR.chrX
0 Mbp
50 Mbp
100 Mbp
150 Mbp
200 Mbp
250 Mbp
> 7.00
6.00 - 7.00
5.00 - 6.00
4.00 - 5.00
3.00 - 4.00
2.00 - 3.00
1.00 - 2.00
0.50 - 1.00
0.10 - 0.50
0.00 - 0.10
no SNPs
masked

T85

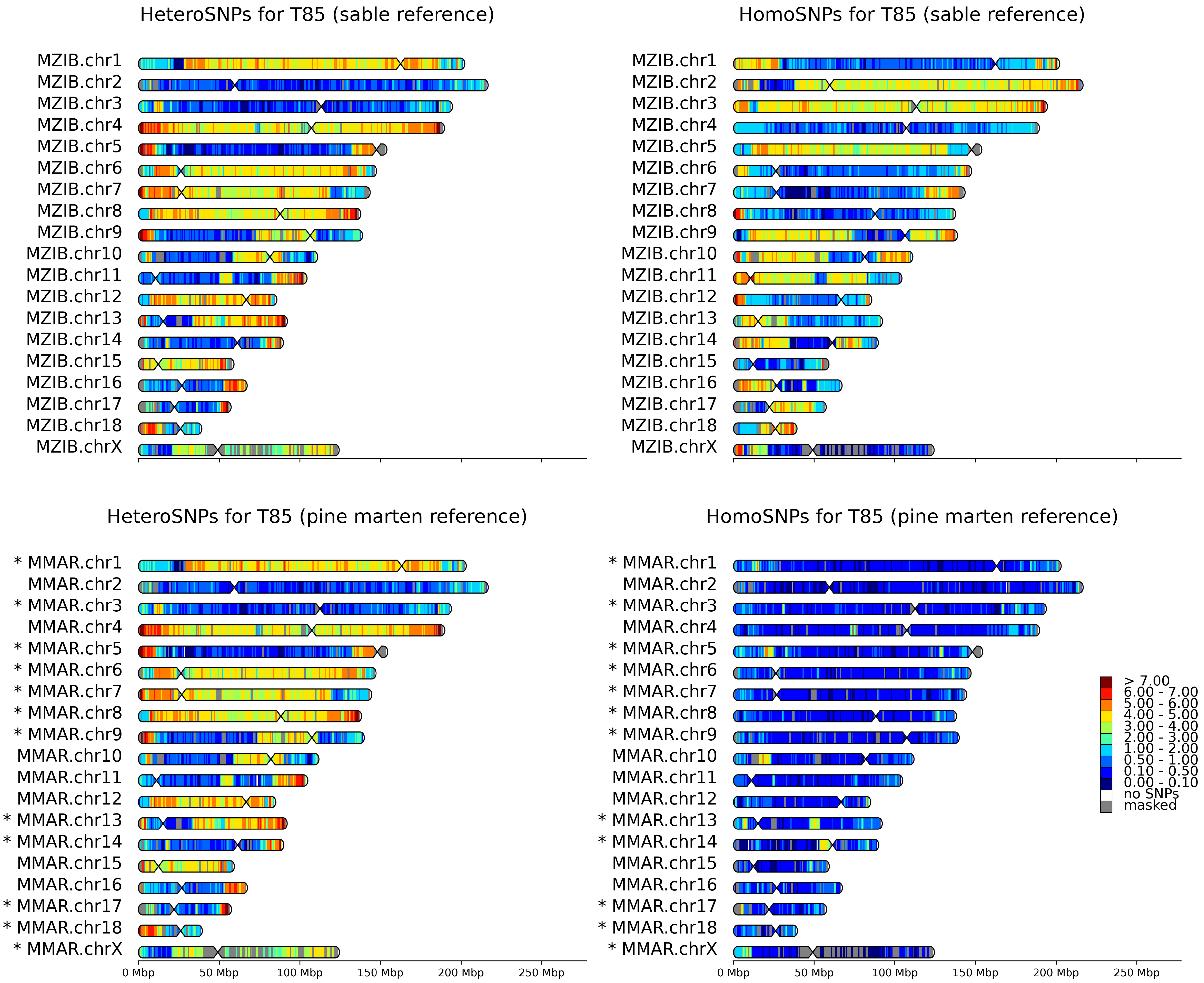
HeteroSNPs for T85 (sable reference)
HomoSNPs for T85 (sable reference)
MZIB.chr1
MZIB.chr2
MZIB.chr3
MZIB.chr4
MZIB.chr5
MZIB.chr6
MZIB.chr7
MZIB.chr8
MZIB.chr9
MZIB.chr10
MZIB.chr11
MZIB.chr12
MZIB.chr13
MZIB.chr14
MZIB.chr15
MZIB.chr16
MZIB.chr17
MZIB.chr18
MZIB.chrX
HeteroSNPs for T85 (pine marten reference)
HomoSNPs for T85 (pine marten reference)
* MMAR.chr1
MMAR.chr2
* MMAR.chr3
MMAR.chr4
* MMAR.chr5
* MMAR.chr6
* MMAR.chr7
* MMAR.chr8
* MMAR.chr9
MMAR.chr10
MMAR.chr11
MMAR.chr12
* MMAR.chr13
* MMAR.chr14
MMAR.chr15
MMAR.chr16
* MMAR.chr17
* MMAR.chr18
* MMAR.chrX
0 Mbp
50 Mbp
100 Mbp
150 Mbp
200 Mbp
250 Mbp
> 7.00
6.00 - 7.00
5.00 - 6.00
4.00 - 5.00
3.00 - 4.00
2.00 - 3.00
1.00 - 2.00
0.50 - 1.00
0.10 - 0.50
0.00 - 0.10
no SNPs
masked

T86

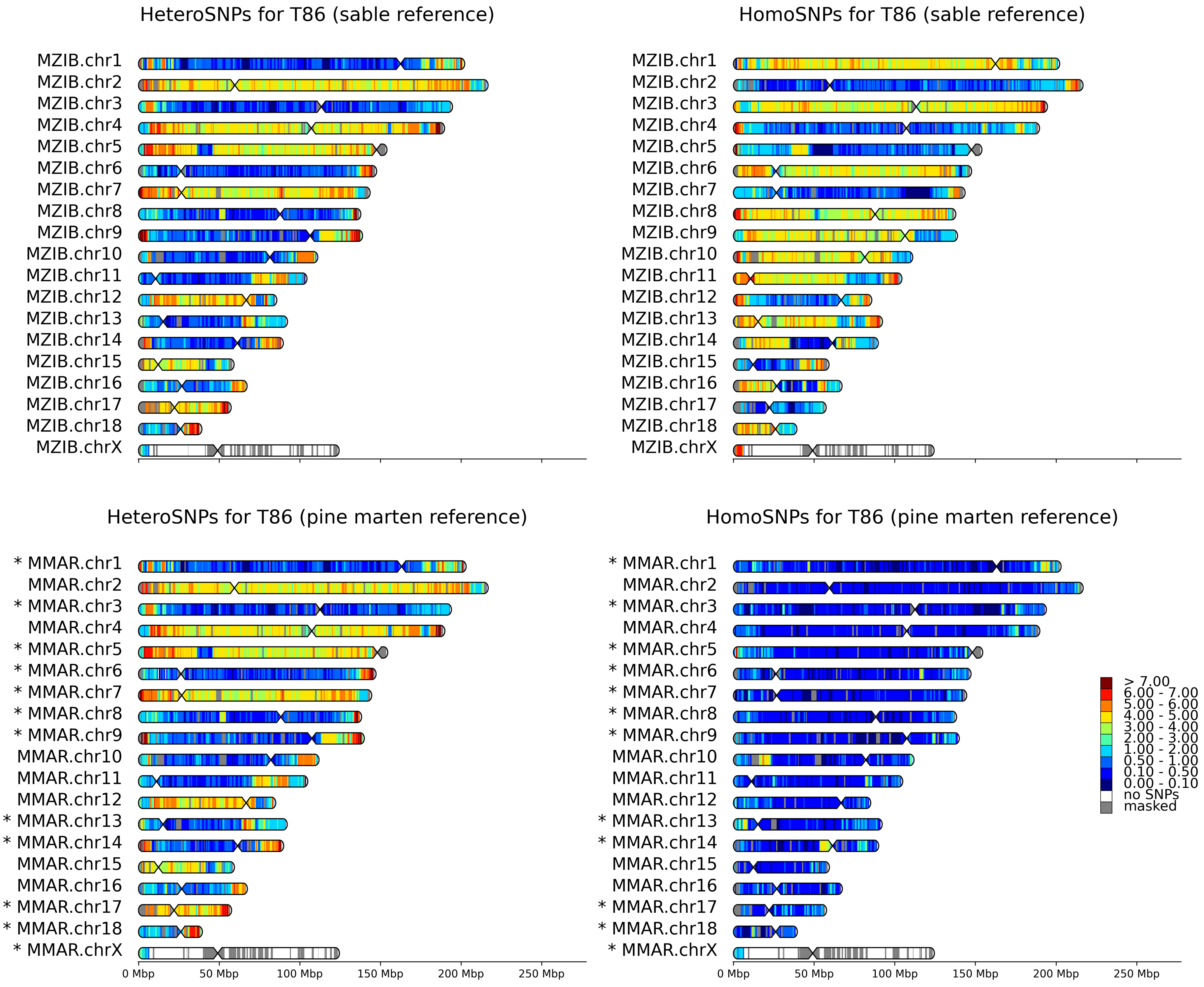
HeteroSNPs for T86 (sable reference)
HomoSNPs for T86 (sable reference)
MZIB.chr1
MZIB.chr2
MZIB.chr3
MZIB.chr4
MZIB.chr5
MZIB.chr6
MZIB.chr7
MZIB.chr8
MZIB.chr9
MZIB.chr10
MZIB.chr11
MZIB.chr12
MZIB.chr13
MZIB.chr14
MZIB.chr15
MZIB.chr16
MZIB.chr17
MZIB.chr18
MZIB.chrX
HeteroSNPs for T86 (pine marten reference)
HomoSNPs for T86 (pine marten reference)
* MMAR.chr1
MMAR.chr2
* MMAR.chr3
MMAR.chr4
* MMAR.chr5
* MMAR.chr6
* MMAR.chr7
* MMAR.chr8
* MMAR.chr9
MMAR.chr10
MMAR.chr11
MMAR.chr12
* MMAR.chr13
* MMAR.chr14
MMAR.chr15
MMAR.chr16
* MMAR.chr17
* MMAR.chr18
* MMAR.chrX
0 Mbp
50 Mbp
100 Mbp
150 Mbp
200 Mbp
250 Mbp
> 7.00
6.00 - 7.00
5.00 - 6.00
4.00 - 5.00
3.00 - 4.00
2.00 - 3.00
1.00 - 2.00
0.50 - 1.00
0.10 - 0.50
0.00 - 0.10
no SNPs
masked

T87

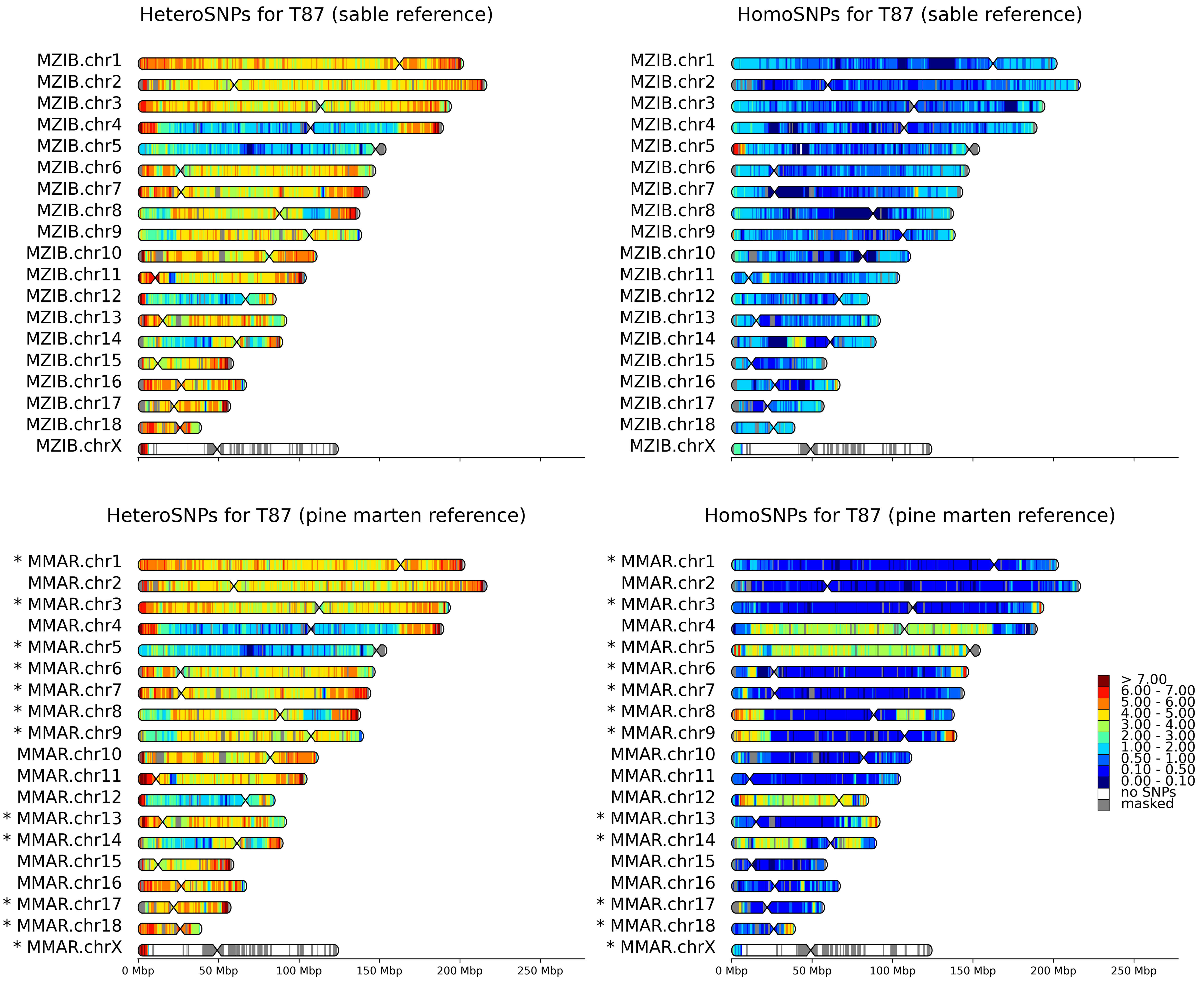
HeteroSNPs for T87 (sable reference)
HomoSNPs for T87 (sable reference)
MZIB.chr1
MZIB.chr2
MZIB.chr3
MZIB.chr4
MZIB.chr5
MZIB.chr6
MZIB.chr7
MZIB.chr8
MZIB.chr9
MZIB.chr10
MZIB.chr11
MZIB.chr12
MZIB.chr13
MZIB.chr14
MZIB.chr15
MZIB.chr16
MZIB.chr17
MZIB.chr18
MZIB.chrX
HeteroSNPs for T87 (pine marten reference)
HomoSNPs for T87 (pine marten reference)
* MMAR.chr1
MMAR.chr2
* MMAR.chr3
MMAR.chr4
* MMAR.chr5
* MMAR.chr6
* MMAR.chr7
* MMAR.chr8
* MMAR.chr9
MMAR.chr10
MMAR.chr11
MMAR.chr12
* MMAR.chr13
* MMAR.chr14
MMAR.chr15
MMAR.chr16
* MMAR.chr17
* MMAR.chr18
* MMAR.chrX
0 Mbp
50 Mbp
100 Mbp
150 Mbp
200 Mbp
250 Mbp
> 7.00
6.00 - 7.00
5.00 - 6.00
4.00 - 5.00
3.00 - 4.00
2.00 - 3.00
1.00 - 2.00
0.50 - 1.00
0.10 - 0.50
0.00 - 0.10
no SNPs
masked
