# Supplementary material for: Genomics of Sable (Martes zibellina) × Pine Marten (Martes martes) Hybridization
Source: Genome Biol Evol. 2026 Mar 5;18(3):evag018. doi: 10.1093/gbe/evag018 (PMC12960073; doi:10.1093/gbe/evag018)
Supplement: evag018_Supplementary_Data [file evag018_supplementary_data.zip › SupplementaryFiles/SupplementaryFile_8.Runs_of_homozygosity.pdf]

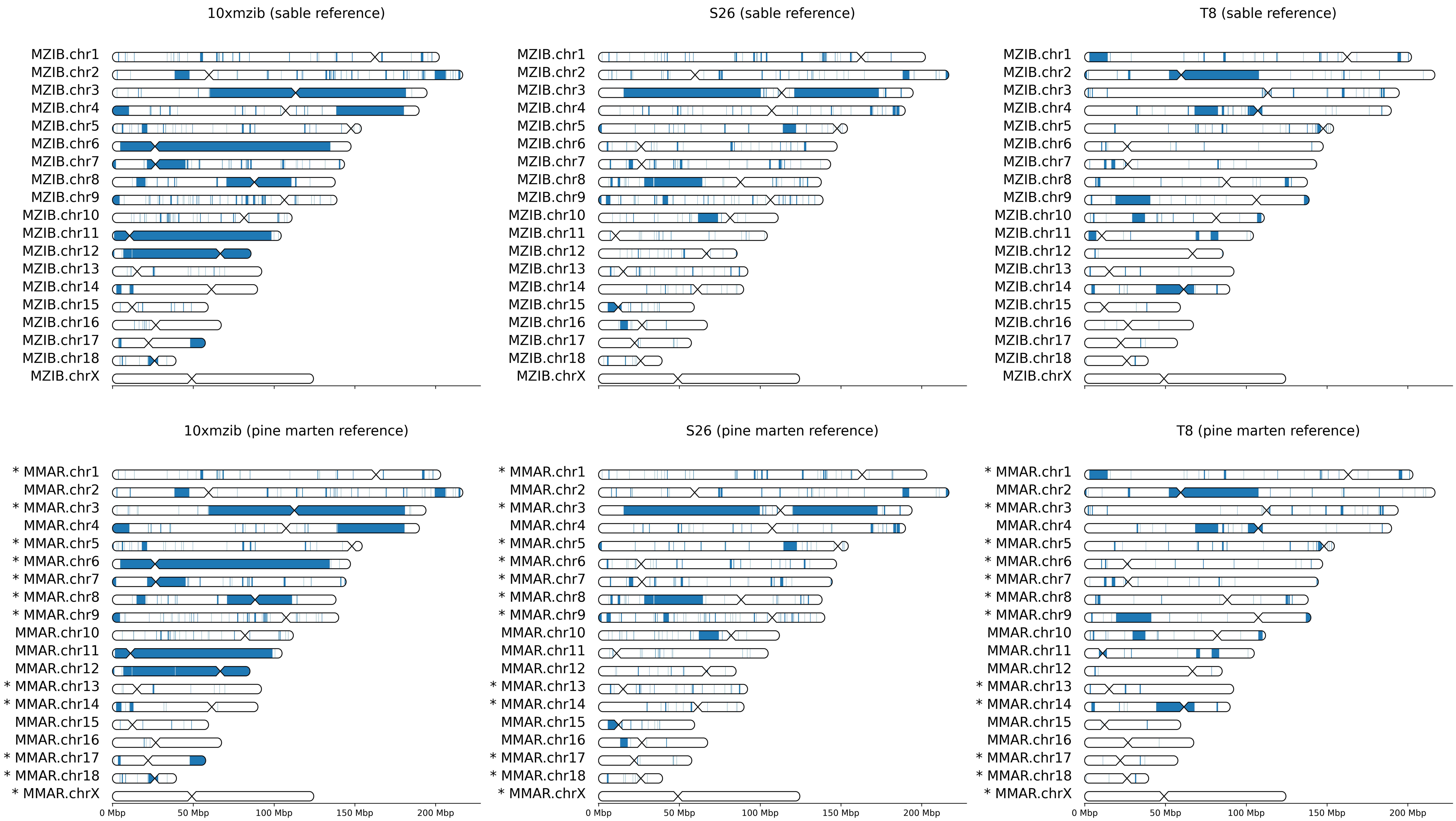
10xmzib (sable reference)
S26 (sable reference)
T8 (sable reference)
MZIB.chr1
MZIB.chr2
MZIB.chr3
MZIB.chr4
MZIB.chr5
MZIB.chr6
MZIB.chr7
MZIB.chr8
MZIB.chr9
MZIB.chr10
MZIB.chr11
MZIB.chr12
MZIB.chr13
MZIB.chr14
MZIB.chr15
MZIB.chr16
MZIB.chr17
MZIB.chr18
MZIB.chrX
10xmzib (pine marten reference)
S26 (pine marten reference)
T8 (pine marten reference)
* MMAR.chr1
MMAR.chr2
* MMAR.chr3
MMAR.chr4
* MMAR.chr5
* MMAR.chr6
* MMAR.chr7
* MMAR.chr8
* MMAR.chr9
MMAR.chr10
MMAR.chr11
MMAR.chr12
* MMAR.chr13
* MMAR.chr14
MMAR.chr15
MMAR.chr16
* MMAR.chr17
* MMAR.chr18
* MMAR.chrX
0 Mbp
50 Mbp
100 Mbp
150 Mbp
200 Mbp

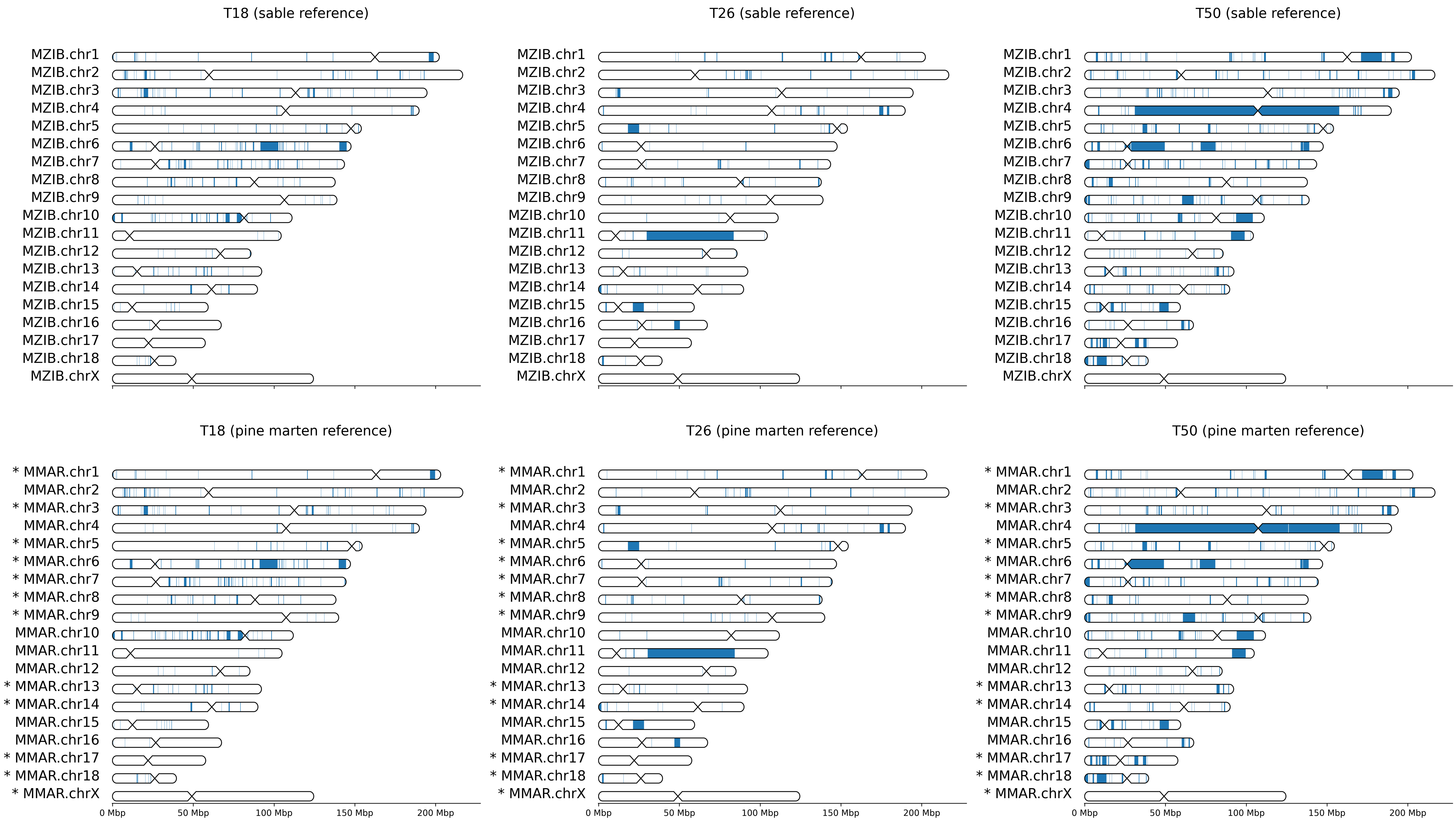
T18 (sable reference)
T26 (sable reference)
T50 (sable reference)
MZIB.chr1
MZIB.chr2
MZIB.chr3
MZIB.chr4
MZIB.chr5
MZIB.chr6
MZIB.chr7
MZIB.chr8
MZIB.chr9
MZIB.chr10
MZIB.chr11
MZIB.chr12
MZIB.chr13
MZIB.chr14
MZIB.chr15
MZIB.chr16
MZIB.chr17
MZIB.chr18
MZIB.chrX
T18 (pine marten reference)
T26 (pine marten reference)
T50 (pine marten reference)
* MMAR.chr1
MMAR.chr2
* MMAR.chr3
MMAR.chr4
* MMAR.chr5
* MMAR.chr6
* MMAR.chr7
* MMAR.chr8
* MMAR.chr9
MMAR.chr10
MMAR.chr11
MMAR.chr12
* MMAR.chr13
* MMAR.chr14
MMAR.chr15
MMAR.chr16
* MMAR.chr17
* MMAR.chr18
* MMAR.chrX
0 Mbp
50 Mbp
100 Mbp
150 Mbp
200 Mbp

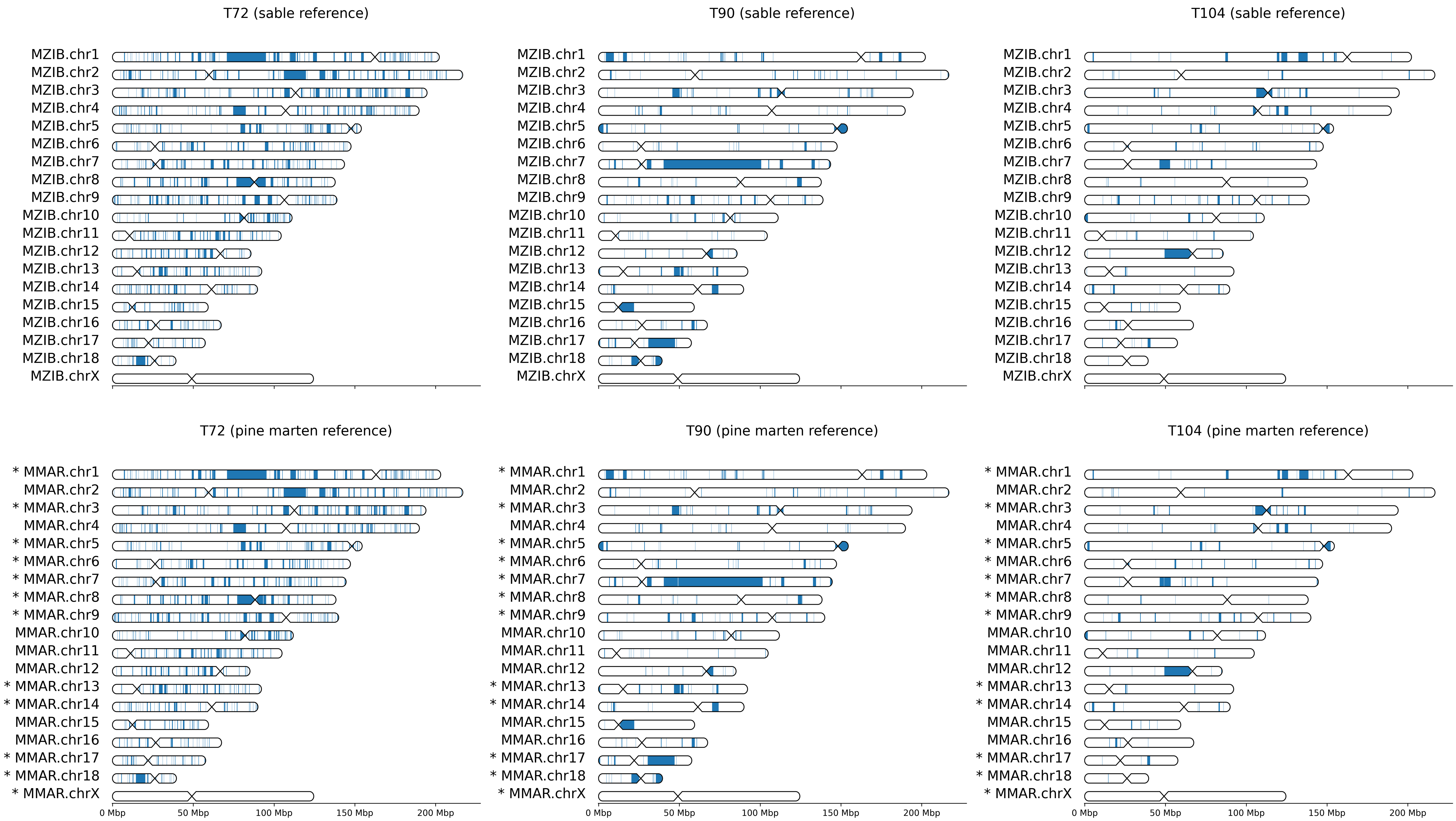

T72 (sable reference)
T90 (sable reference)
T104 (sable reference)
MZIB.chr1
MZIB.chr2
MZIB.chr3
MZIB.chr4
MZIB.chr5
MZIB.chr6
MZIB.chr7
MZIB.chr8
MZIB.chr9
MZIB.chr10
MZIB.chr11
MZIB.chr12
MZIB.chr13
MZIB.chr14
MZIB.chr15
MZIB.chr16
MZIB.chr17
MZIB.chr18
MZIB.chrX
T72 (pine marten reference)
T90 (pine marten reference)
T104 (pine marten reference)
* MMAR.chr1
MMAR.chr2
* MMAR.chr3
MMAR.chr4
* MMAR.chr5
* MMAR.chr6
* MMAR.chr7
* MMAR.chr8
* MMAR.chr9
MMAR.chr10
MMAR.chr11
MMAR.chr12
* MMAR.chr13
* MMAR.chr14
MMAR.chr15
MMAR.chr16
* MMAR.chr17
* MMAR.chr18
* MMAR.chrX
0 Mbp
50 Mbp
100 Mbp
150 Mbp
200 Mbp

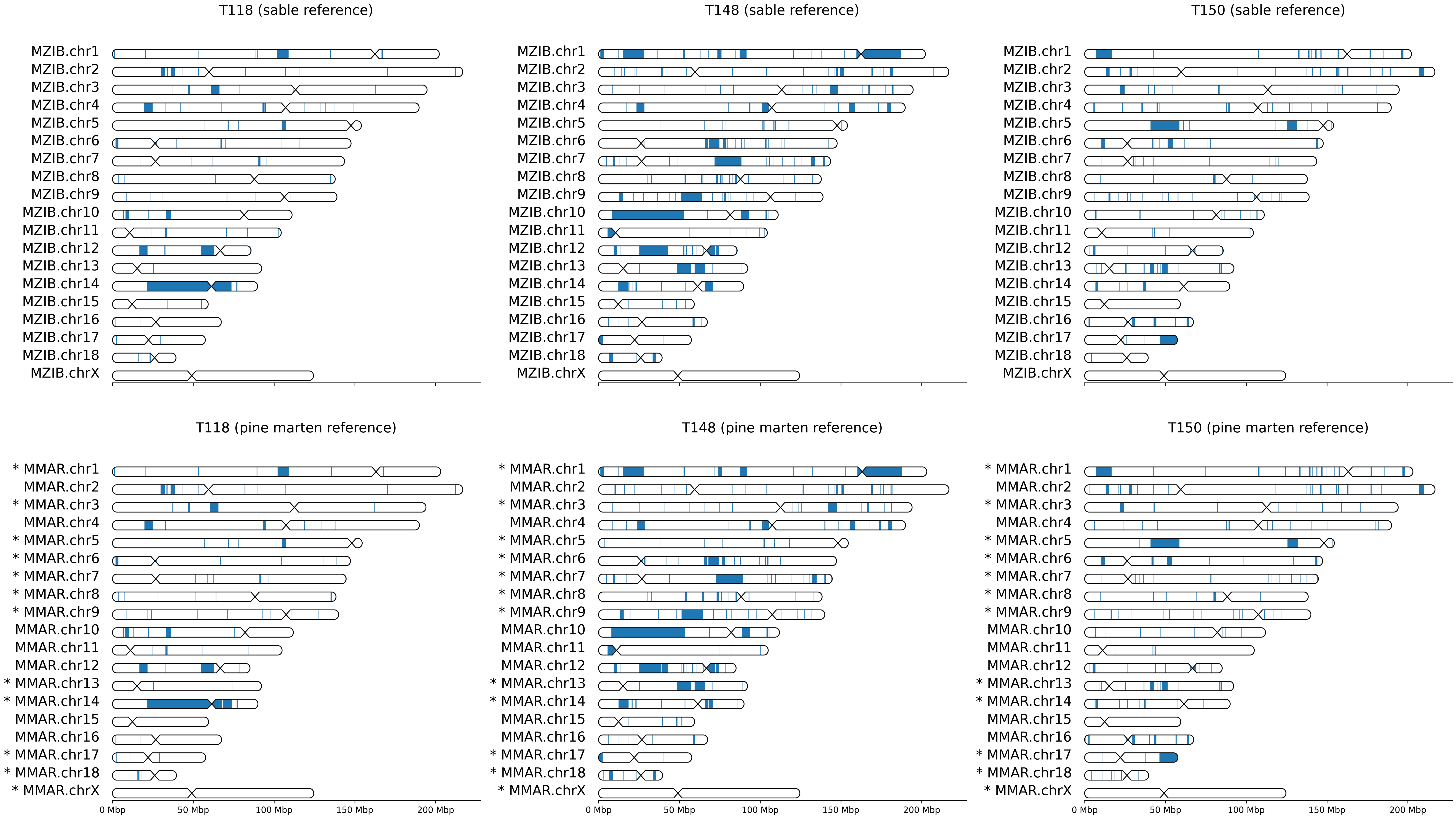

T118 (sable reference)
T148 (sable reference)
T150 (sable reference)
MZIB.chr1
MZIB.chr2
MZIB.chr3
MZIB.chr4
MZIB.chr5
MZIB.chr6
MZIB.chr7
MZIB.chr8
MZIB.chr9
MZIB.chr10
MZIB.chr11
MZIB.chr12
MZIB.chr13
MZIB.chr14
MZIB.chr15
MZIB.chr16
MZIB.chr17
MZIB.chr18
MZIB.chrX
T118 (pine marten reference)
T148 (pine marten reference)
T150 (pine marten reference)
* MMAR.chr1
MMAR.chr2
* MMAR.chr3
MMAR.chr4
* MMAR.chr5
* MMAR.chr6
* MMAR.chr7
* MMAR.chr8
* MMAR.chr9
MMAR.chr10
MMAR.chr11
MMAR.chr12
* MMAR.chr13
* MMAR.chr14
MMAR.chr15
MMAR.chr16
* MMAR.chr17
* MMAR.chr18
* MMAR.chrX
0 Mbp
50 Mbp
100 Mbp
150 Mbp
200 Mbp

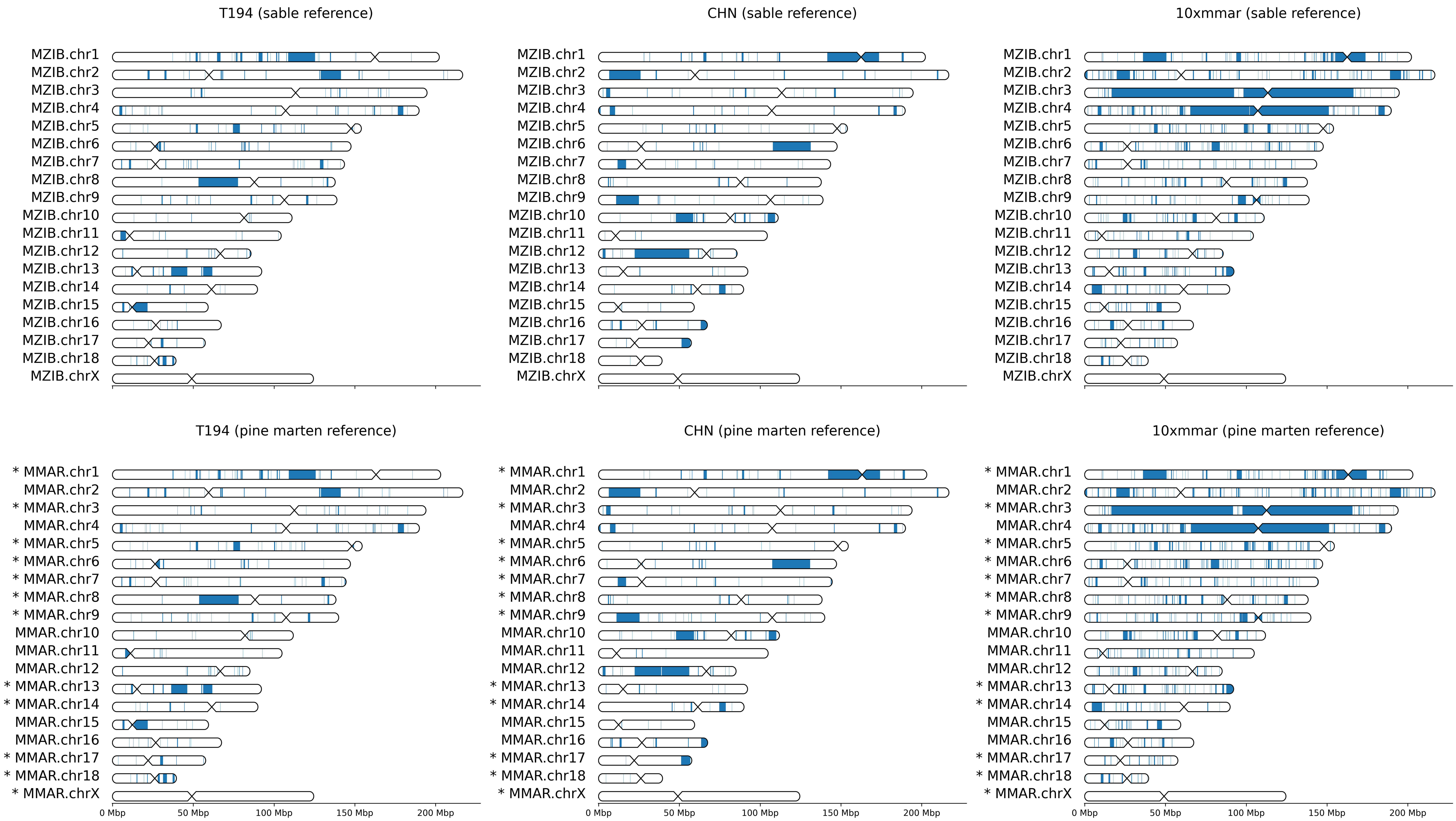

T194 (sable reference)
CHN (sable reference)
10xmmar (sable reference)
MZIB.chr1
MZIB.chr2
MZIB.chr3
MZIB.chr4
MZIB.chr5
MZIB.chr6
MZIB.chr7
MZIB.chr8
MZIB.chr9
MZIB.chr10
MZIB.chr11
MZIB.chr12
MZIB.chr13
MZIB.chr14
MZIB.chr15
MZIB.chr16
MZIB.chr17
MZIB.chr18
MZIB.chrX
T194 (pine marten reference)
CHN (pine marten reference)
10xmmar (pine marten reference)
* MMAR.chr1
MMAR.chr2
* MMAR.chr3
MMAR.chr4
* MMAR.chr5
* MMAR.chr6
* MMAR.chr7
* MMAR.chr8
* MMAR.chr9
MMAR.chr10
MMAR.chr11
MMAR.chr12
* MMAR.chr13
* MMAR.chr14
MMAR.chr15
MMAR.chr16
* MMAR.chr17
* MMAR.chr18
* MMAR.chrX
0 Mbp
50 Mbp
100 Mbp
150 Mbp
200 Mbp

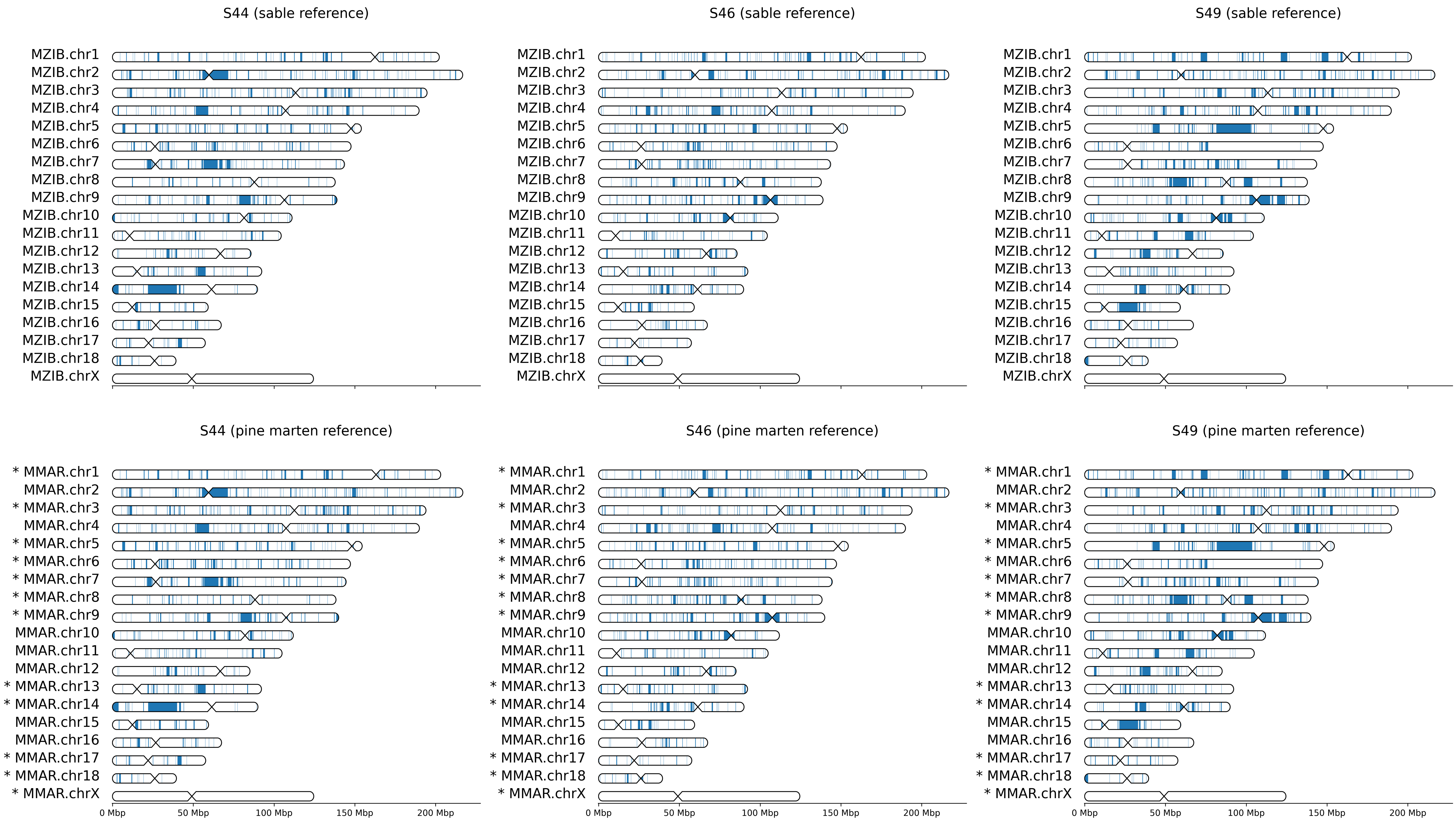

S44 (sable reference)
S46 (sable reference)
S49 (sable reference)
MZIB.chr1
MZIB.chr2
MZIB.chr3
MZIB.chr4
MZIB.chr5
MZIB.chr6
MZIB.chr7
MZIB.chr8
MZIB.chr9
MZIB.chr10
MZIB.chr11
MZIB.chr12
MZIB.chr13
MZIB.chr14
MZIB.chr15
MZIB.chr16
MZIB.chr17
MZIB.chr18
MZIB.chrX
S44 (pine marten reference)
S46 (pine marten reference)
S49 (pine marten reference)
* MMAR.chr1
MMAR.chr2
* MMAR.chr3
MMAR.chr4
* MMAR.chr5
* MMAR.chr6
* MMAR.chr7
* MMAR.chr8
* MMAR.chr9
MMAR.chr10
MMAR.chr11
MMAR.chr12
* MMAR.chr13
* MMAR.chr14
MMAR.chr15
MMAR.chr16
* MMAR.chr17
* MMAR.chr18
* MMAR.chrX
0 Mbp
50 Mbp
100 Mbp
150 Mbp
200 Mbp

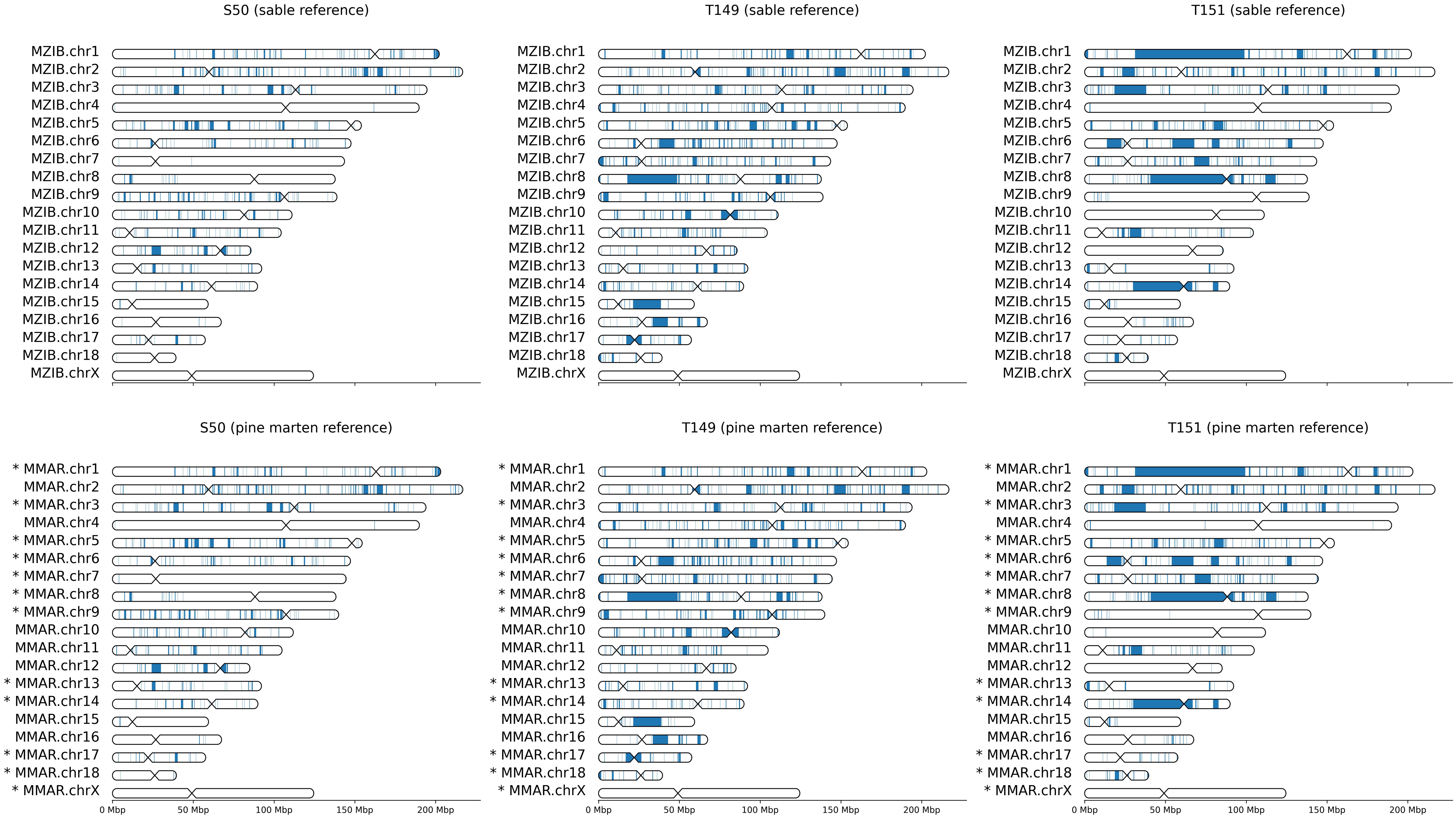
S50 (sable reference)
T149 (sable reference)
T151 (sable reference)
MZIB.chr1
MZIB.chr2
MZIB.chr3
MZIB.chr4
MZIB.chr5
MZIB.chr6
MZIB.chr7
MZIB.chr8
MZIB.chr9
MZIB.chr10
MZIB.chr11
MZIB.chr12
MZIB.chr13
MZIB.chr14
MZIB.chr15
MZIB.chr16
MZIB.chr17
MZIB.chr18
MZIB.chrX
S50 (pine marten reference)
T149 (pine marten reference)
T151 (pine marten reference)
* MMAR.chr1
MMAR.chr2
* MMAR.chr3
MMAR.chr4
* MMAR.chr5
* MMAR.chr6
* MMAR.chr7
* MMAR.chr8
* MMAR.chr9
MMAR.chr10
MMAR.chr11
MMAR.chr12
* MMAR.chr13
* MMAR.chr14
MMAR.chr15
MMAR.chr16
* MMAR.chr17
* MMAR.chr18
* MMAR.chrX
0 Mbp
50 Mbp
100 Mbp
150 Mbp
200 Mbp

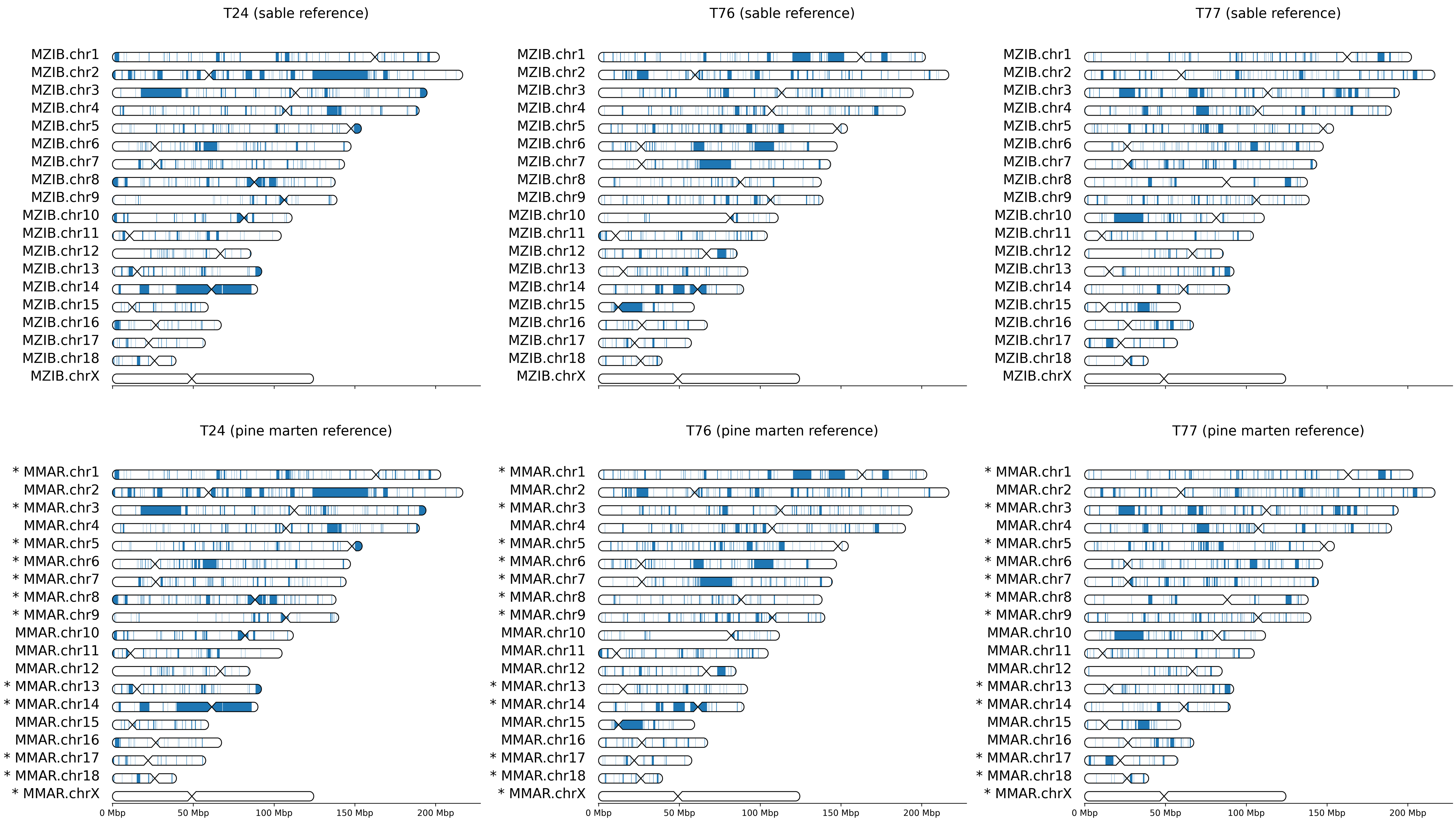

T24 (sable reference)
T76 (sable reference)
T77 (sable reference)
MZIB.chr1
MZIB.chr2
MZIB.chr3
MZIB.chr4
MZIB.chr5
MZIB.chr6
MZIB.chr7
MZIB.chr8
MZIB.chr9
MZIB.chr10
MZIB.chr11
MZIB.chr12
MZIB.chr13
MZIB.chr14
MZIB.chr15
MZIB.chr16
MZIB.chr17
MZIB.chr18
MZIB.chrX
T24 (pine marten reference)
T76 (pine marten reference)
T77 (pine marten reference)
* MMAR.chr1
MMAR.chr2
* MMAR.chr3
MMAR.chr4
* MMAR.chr5
* MMAR.chr6
* MMAR.chr7
* MMAR.chr8
* MMAR.chr9
MMAR.chr10
MMAR.chr11
MMAR.chr12
* MMAR.chr13
* MMAR.chr14
MMAR.chr15
MMAR.chr16
* MMAR.chr17
* MMAR.chr18
* MMAR.chrX
0 Mbp
50 Mbp
100 Mbp
150 Mbp
200 Mbp

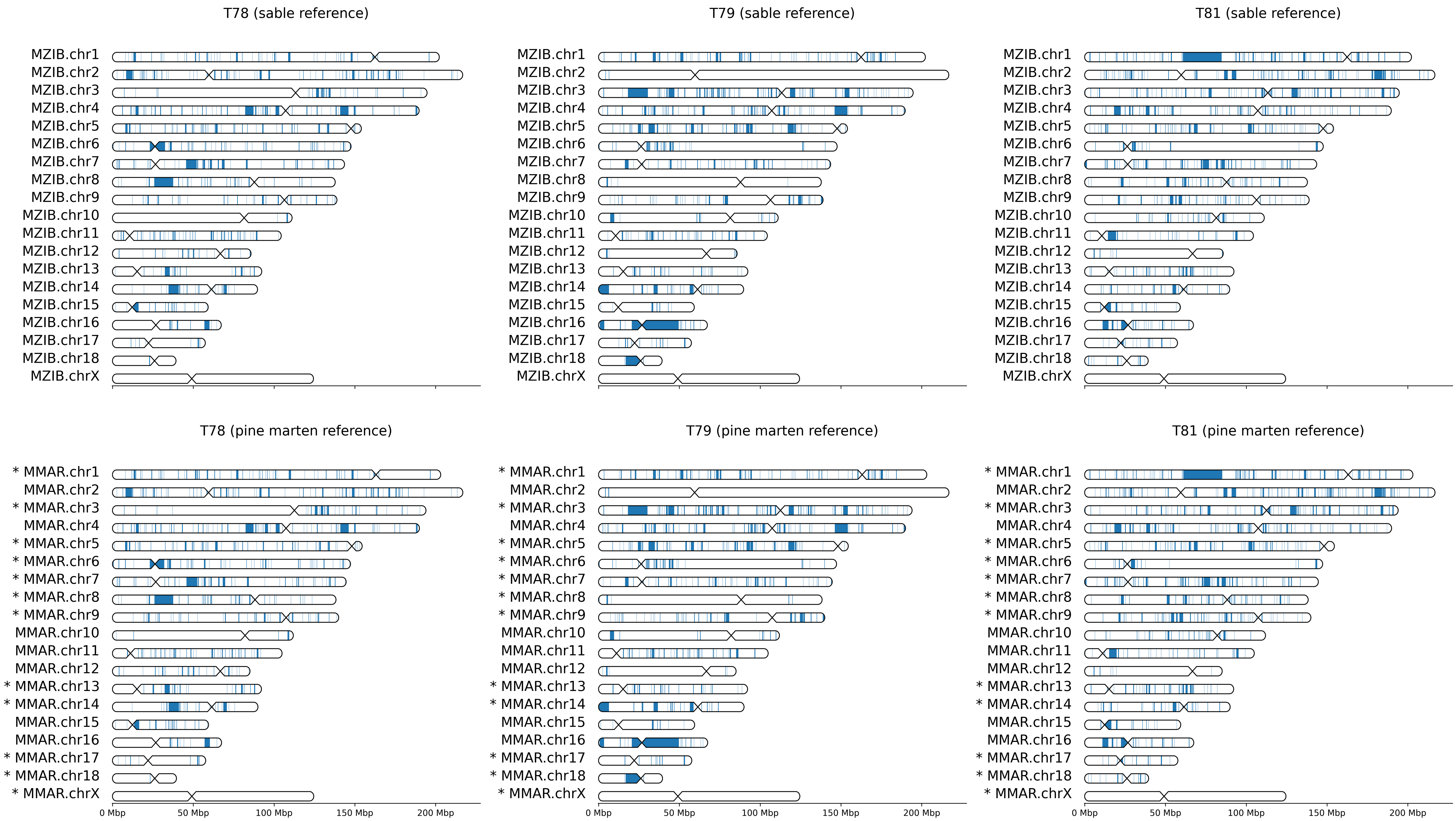
T78 (sable reference)
T79 (sable reference)
T81 (sable reference)
MZIB.chr1
MZIB.chr2
MZIB.chr3
MZIB.chr4
MZIB.chr5
MZIB.chr6
MZIB.chr7
MZIB.chr8
MZIB.chr9
MZIB.chr10
MZIB.chr11
MZIB.chr12
MZIB.chr13
MZIB.chr14
MZIB.chr15
MZIB.chr16
MZIB.chr17
MZIB.chr18
MZIB.chrX
T78 (pine marten reference)
T79 (pine marten reference)
T81 (pine marten reference)
* MMAR.chr1
MMAR.chr2
* MMAR.chr3
MMAR.chr4
* MMAR.chr5
* MMAR.chr6
* MMAR.chr7
* MMAR.chr8
* MMAR.chr9
MMAR.chr10
MMAR.chr11
MMAR.chr12
* MMAR.chr13
* MMAR.chr14
MMAR.chr15
MMAR.chr16
* MMAR.chr17
* MMAR.chr18
* MMAR.chrX
0 Mbp
50 Mbp
100 Mbp
150 Mbp
200 Mbp

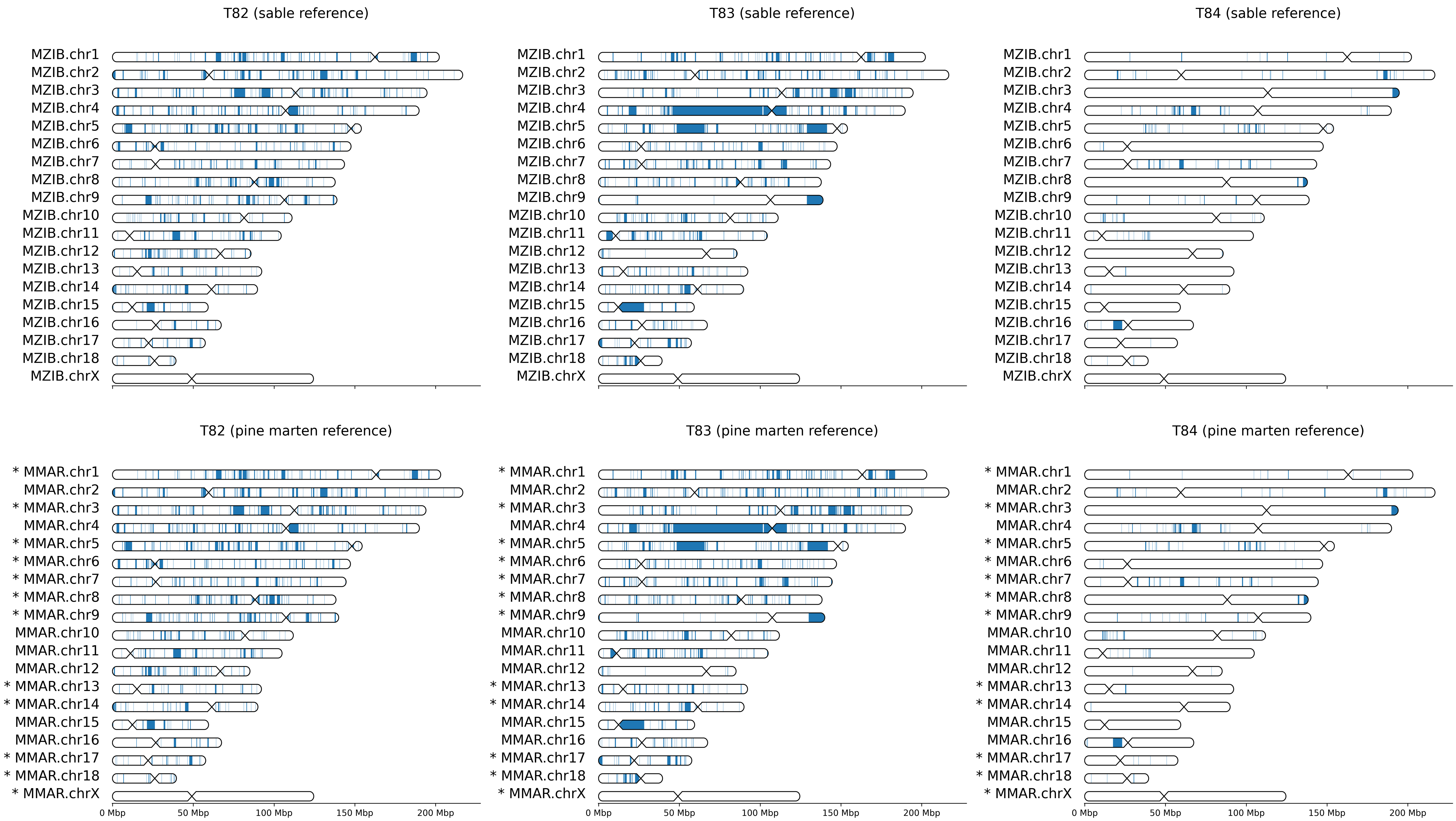
T82 (sable reference)
T83 (sable reference)
T84 (sable reference)
MZIB.chr1
MZIB.chr2
MZIB.chr3
MZIB.chr4
MZIB.chr5
MZIB.chr6
MZIB.chr7
MZIB.chr8
MZIB.chr9
MZIB.chr10
MZIB.chr11
MZIB.chr12
MZIB.chr13
MZIB.chr14
MZIB.chr15
MZIB.chr16
MZIB.chr17
MZIB.chr18
MZIB.chrX
T82 (pine marten reference)
T83 (pine marten reference)
T84 (pine marten reference)
* MMAR.chr1
MMAR.chr2
* MMAR.chr3
MMAR.chr4
* MMAR.chr5
* MMAR.chr6
* MMAR.chr7
* MMAR.chr8
* MMAR.chr9
MMAR.chr10
MMAR.chr11
MMAR.chr12
* MMAR.chr13
* MMAR.chr14
MMAR.chr15
MMAR.chr16
* MMAR.chr17
* MMAR.chr18
* MMAR.chrX
0 Mbp
50 Mbp
100 Mbp
150 Mbp
200 Mbp

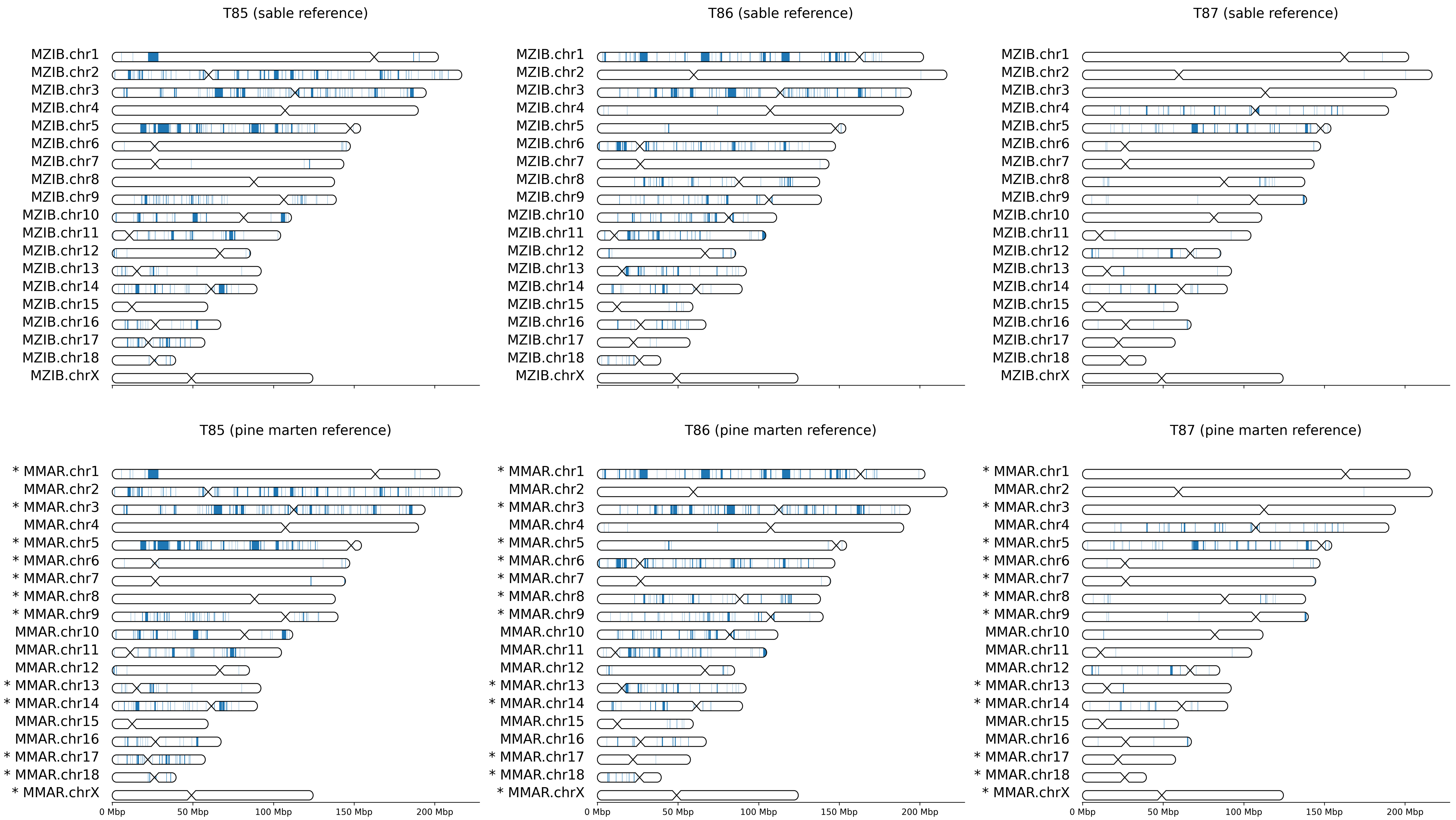

T85 (sable reference)
T86 (sable reference)
T87 (sable reference)
MZIB.chr1
MZIB.chr2
MZIB.chr3
MZIB.chr4
MZIB.chr5
MZIB.chr6
MZIB.chr7
MZIB.chr8
MZIB.chr9
MZIB.chr10
MZIB.chr11
MZIB.chr12
MZIB.chr13
MZIB.chr14
MZIB.chr15
MZIB.chr16
MZIB.chr17
MZIB.chr18
MZIB.chrX
T85 (pine marten reference)
T86 (pine marten reference)
T87 (pine marten reference)
* MMAR.chr1
MMAR.chr2
* MMAR.chr3
MMAR.chr4
* MMAR.chr5
* MMAR.chr6
* MMAR.chr7
* MMAR.chr8
* MMAR.chr9
MMAR.chr10
MMAR.chr11
MMAR.chr12
* MMAR.chr13
* MMAR.chr14
MMAR.chr15
MMAR.chr16
* MMAR.chr17
* MMAR.chr18
* MMAR.chrX
0 Mbp
50 Mbp
100 Mbp
150 Mbp
200 Mbp
