# Supplementary material for: Genomics of Sable (Martes zibellina) × Pine Marten (Martes martes) Hybridization
Source: Genome Biol Evol. 2026 Mar 5;18(3):evag018. doi: 10.1093/gbe/evag018 (PMC12960073; doi:10.1093/gbe/evag018)
Supplement: evag018_Supplementary_Data [file evag018_supplementary_data.zip › SupplementaryMaterials.docx]

Supplementary Materials (SM)

Andrey A. Tomarovsky^1,2*^, Azamat A. Totikov^1,2x^, Tatiana M. Bulyonkova^3x^, Polina L. Perelman^1^, Alexei V. Abramov^4^, Natalia A. Serdyukova^1^, Aliya R. Yakupova^5,6^, Dmitry Prokopov^7,8^, Violetta R. Beklemisheva^1^, Mikkel-Holger S Sinding^9,10^, Guzel Davletshina^1^, Maria Pobedintseva^1^, Ksenia Krasheninnikova^11^, Daniel W. Foerster^12^, Anna S. Mukhacheva^13^, Alexandra Mironova^14^, Michail Sidorov^15^, Wenhui Nie^16^, Jinhuan Wang^16^, Svetlana A. Romanenko^1^, Anastasiya A. Proskuryakova^1^, Malcolm Ferguson-Smith^17^, Fengtang Yang^18^, Nikolay Cherkasov^19^, Elena Balanovskaya^20^, M. Thomas P. Gilbert^21,22^, Innokentiy M. Okhlopkov^15^, Anna Zhuk^23,24^, Alexander S. Graphodatsky^1^, Roger Powell^25^, Klaus-Peter Koepfli^26^, Sergei Kliver^21^

^1^ Laboratory of Diversity and Evolution of Genomes, Institute of Molecular and Cellular Biology SB RAS, 8/2 Acad. Lavrentiev ave., Novosibirsk, 630090, Russia. [polina.perelman@gmail.com](mailto:polina.perelman@gmail.com) (<https://orcid.org/0000-0002-0982-5100>), [serd@mcb.nsc.ru](mailto:serd@mcb.nsc.ru) (<https://orcid.org/0000-0002-0409-1371>), [bekl@mcb.nsc.ru](mailto:bekl@mcb.nsc.ru) (<https://orcid.org/0000-0002-9122-4143>), [Davlet15628@gmail.com](mailto:Davlet15628@gmail.com) (<https://orcid.org/0009-0000-0650-2675>), [pobedintseva12@gmail.com](mailto:pobedintseva12@gmail.com) (<https://orcid.org/0000-0001-8447-9626>), [rosa@mcb.nsc.ru](mailto:rosa@mcb.nsc.ru) (<https://orcid.org/0000-0002-0951-5209>),

[andrena@mcb.nsc.ru](mailto:andrena@mcb.nsc.ru) (<https://orcid.org/0000-0003-3812-4853>), [graf@mcb.nsc.ru](mailto:graf@mcb.nsc.ru) (<https://orcid.org/0000-0002-8282-1085>).

^2^ Department of Natural Sciences, Novosibirsk State University, 1 Pirogova str., Novosibirsk, 630090, Russia. [andrey.tomarovsky@gmail.com](mailto:andrey.tomarovsky@gmail.com) (<https://orcid.org/0000-0002-6414-704X>), [a.totickov1@gmail.com](mailto:a.totickov1@gmail.com) (<https://orcid.org/0000-0003-1236-631X>).

^3^ Youth Laboratory of Molecular Genetics, Yugra State University, 16 Ulitsa Chekhova, Khanty-Mansiysk, 628011, Russia. [ressaure@gmail.com](mailto:ressaure@gmail.com) (<https://orcid.org/0000-0002-5215-2001>).

^4^ Laboratory for Theriology, Zoological Institute RAS, 1 Universitetskaya emb., St. Petersburg, 199034, Russia. [a.abramov@mail.ru](mailto:a.abramov@mail.ru) (<https://orcid.org/0000-0001-9709-4469>).

^5^ Division of Evolutionary Biology, Ludwig-Maximilians-Universität, 2, Großhaderner str, Planegg, 82152, Germany. [yakupova@bio.lmu.de](mailto:yakupova@bio.lmu.de) (<https://orcid.org/0000-0003-1486-0864>).

^6^ Microevolution and Biodiversity, Max Planck Institute for Biological Intelligence, Eberhard-Gwinner-Straße, Seewiesen, 82319, Germany. [aliya.yakupova@bi.mpg.de](mailto:aliya.yakupova@bi.mpg.de)

^7^ Centre for Haemato-Oncology, Barts Cancer Institute, Queen Mary University of London, London, UK. [d.prokopov@qmul.ac.uk](mailto:d.prokopov@qmul.ac.uk) (<https://orcid.org/0000-0001-8420-5203>).

^8^ QMUL Centre for Epigenetics, Queen Mary University of London, London, UK.

^9^ Center for Evolutionary Hologenomics, The Globe Institute, The University of Copenhagen, Copenhagen, Denmark; [mhssinding@gmail.com](mailto:mhssinding@gmail.com) (<https://orcid.org/0000-0003-1371-219X>).

^10^ Department of Biology, The University of Copenhagen, Copenhagen, Denmark.

^11^ Independent researcher, Wellcome Trust Genome Campus, Hinxton, Saffron Walden CB10 1RQ, United Kingdom. [krasheninnikova@gmail.com](mailto:krasheninnikova@gmail.com) (<https://orcid.org/0000-0002-0604-2047>).

^12^ Leibniz Institute for Zoo and Wildlife Research (IZW), Alfred Kowalke Straße 17, 10315 Berlin, Germany. [DWGFoerster@gmail.com](mailto:DWGFoerster@gmail.com) (<https://orcid.org/0000-0002-6934-0404>).

^13^ Sikhote-Alin Biosphere Zapovednik, 44 Partizanskaya str., Ternei, 692150, Russia. [siam83@mail.ru](mailto:siam83@mail.ru) (<https://orcid.org/0009-0008-0177-8873>).

^14^ Laboratoire de Physiologie Cellulaire and Végétale, Univ. Grenoble Alpes/CNRS/CEA/INRA/IRIG, Grenoble, France. [aleksandra.s.mironova@gmail.com](mailto:aleksandra.s.mironova@gmail.com) (<https://orcid.org/0009-0000-3831-8151>)

^15^ Institute of Biological Problems of Cryolithozone SB RAS, 41 Lenina ave., Yakutsk, 677000, Russia. [sidorov_michail86@mail.ru](mailto:sidorov_michail86@mail.ru) (<https://orcid.org/0000-0003-0333-261X>), [imokhlopkov@yandex.ru](mailto:imokhlopkov@yandex.ru) (<https://orcid.org/0000-0002-6227-5216>).

^16^ State Key Laboratory of Genetic Resources and Evolution, Kunming Institute of Zoology, Chinese Academy of Sciences, Kunming 650223, China, [whnie@mail.kiz.ac.cn](mailto:whnie@mail.kiz.ac.cn), [wangjing315@163.com](mailto:wangjing315@163.com).

^17^ Cambridge Resource Centre for Comparative Genomics, Department of Veterinary Medicine, University of Cambridge, Cambridge CB3 OES, UK. [maf12@cam.ac.uk](mailto:maf12@cam.ac.uk) (<https://orcid.org/0000-0001-9372-1381>)

^18^ School of Life Sciences and Medicine, Shandong University of Technology, Zibo, China. [yangfengtang@sdut.edu.cn](mailto:yangfengtang@sdut.edu.cn) (<https://orcid.org/0000-0002-3573-2354>).

^19^ Vavilov Institute of General Genetics, Moscow, Russia. [x@utrail.org](mailto:x@utrail.org) (<https://orcid.org/0000-0003-1416-0200>).

^20^ Laboratory of human population genetics, Research Centre for Medical Genetics, Moscow 115522, Russia. [balanovska@mail.ru](mailto:balanovska@mail.ru) (<https://orcid.org/0000-0002-3882-8300>).

^21^ Center for Evolutionary Hologenomics, The Globe Institute, The University of Copenhagen, 5A, Oester Farimagsgade, Copenhagen, 1353, Denmark. [tgilbert@sund.ku.dk](mailto:tgilbert@sund.ku.dk) (<https://orcid.org/0000-0002-5805-7195>), [sergei.kliver@sund.ku.dk](mailto:sergei.kliver@sund.ku.dk) (<https://orcid.org/0000-0002-2965-3617>).

^22^ University Museum, NTNU, Trondheim, Norway.

^23^ Institute of Applied Computer Science, ITMO University, 197101 St. Petersburg, Russia. [ania.zhuk@gmail.com](mailto:ania.zhuk@gmail.com) (<https://orcid.org/0000-0001-8683-9533>).

^24^ Laboratory of Amyloid Biology, St. Petersburg State University, 199034 St. Petersburg, Russia.

^25^ North Carolina State University. [rpowell@ncsu.edu](mailto:rpowell@ncsu.edu) (<https://orcid.org/0000-0001-9419-4034>).

^26^ Smithsonian-Mason School of Conservation, 1500 Remount Road, Front Royal, VA 22630, USA. [klauspeter.koepfli527@gmail.com](mailto:klauspeter.koepfli527@gmail.com) (<https://orcid.org/0000-0001-7281-0676>).

* corresponding author

^x^ equal contribution

# Table of Contents

[**Table of Contents 4**](#_nltvb7vc19px)

[**List of abbreviations 6**](#_hs64susagb38)

[**1. Supplementary facts for Introduction 7**](#_q0hdizkzfduu)

[1.1. Morphological features of kidases (hybrids) compared to the parental species 7](#_g7mktq49pjp8)

[**2. Supplementary Results and Discussion 8**](#_94tppzze22wi)

[2.1. Samples and ranges 8](#_4ed03w4r0anm)

[2.2. Phylogeny and dating 9](#_o3inyrnf5r4w)

[2.3. Fossil calibrations 9](#_ih8h97vbw1jf)

[2.4. Divergence times and reference choice 12](#_3xcmujk2j6z8)

[2.5. Reference-related bias 13](#_458jr3eetouz)

[2.6. Concept of the heterozygosity component analysis (HCA) 21](#_m6lgbvrttdsj)

[2.7. Classification of individuals by morphology 23](#_587nb4uecqcm)

[2.8. Issues of dating demographic trajectories 25](#_eb5u0fj54d4z)

[2.9. Divergence dating from demographic trajectories 26](#_31n2t6goh7w)

[2.10. Speculations on migration routes and speciation 26](#_tbso32qo4e9e)

[2.11. Candidate genes related to phenotypic and diet differences 27](#_wutyjixaof94)

[**3. Supplementary Methods 30**](#_pe9pcbbgnl02)

[3.1 Phylogeny reconstruction 30](#_pjh19qczpns8)

[3.2. Localization and genotyping of previously known STR loci. 31](#_cnmatz3sbuvd)

[3.3. Components of heterozygosity distributions 33](#_rr7yktq2vjp6)

[3.4. Algorithm for identification of a pseudoautosomal region. 35](#_p0le6plirqrv)

[3.5. snakeSTR pipeline. 36](#_6beqp1xhtf25)

[3.6. Fst and Tajima’s D 38](#_fpoflzjnan8p)

[3.7. Morphological analysis 40](#_yo3waonfsedc)

[**4. Additional Materials 41**](#_ndva7hk6ajis)

[4.1. Additional Figures 41](#_lzr02y2n6oy)

[4.2. Additional Tables 50](#_yx2h7qikjled)

[**5. Supplementary Files 61**](#_6bmdbkkz6864)

[Supplementary File 1. HyDe, F3- and D-stastistics 61](#_ezjmvtnc0a22)

[Supplementary File 2. Description of the genotyped STR loci. 62](#_28l0q2r9sut)

[Supplementary File 3. Description of the used mtDNA data. 62](#_710m616hta8e)

[Supplementary File 4. Mitochondrial haplotype network (large scale figure). 62](#_t0vqbwna1y17)

[Supplementary File 5. Model fitting statistics for Heterozygosity Component Analysis (HCA). 62](#_fj0foziljlcs)

[Supplementary File 6. Distribution of the heterozygous and homozygous along chromosomes. 62](#_fsw7ylf85xe6)

[Supplementary File 7. Local ancestry along chromosomes. 63](#_7qihfl4itb58)

[Supplementary File 8. Runs of homozygosity (RoH). 63](#_57m3qanhcm1y)

[Supplementary File 9. GO-analysis and description of related genes for regions of interest. 63](#_63pdg2eboqcx)

[Supplementary File 10. Phylogenetic trees in NEWICK format. 63](#_bio91rvmznte)

[Supplementary File 11. In silico PCR of STR loci. 64](#_4ysnziafpmdq)

[Supplementary File 12. Input data for haplotype network reconstruction (PopArt). 64](#_74cw3sdohuyh)

[**6. References 65**](#_tav2cxbfjc28)

# List of abbreviations

MT – Main Text

SM – Supplementary Method

AM – Additional Materials

AF – Additional Figures

AT – Additional Tables

PCA – Principal Component Analysis

CI – Confidence Interval

RoH – Run of Homozygosity

HCA – Heterozygosity Component Analysis

# 1. Supplementary facts for Introduction

## 1.1. Morphological features of kidases (hybrids) compared to the parental species

Generally, kidases possess mixed morphological traits inherited from both parent species (MT Figure 1A). Traditionally, identification of kidases relies on tail length and coat coloration [(Yurgenson 1947)](https://www.zotero.org/google-docs/?cEIfSi). Compared to pine martens, hybrid individuals often exhibit sable-like features, such as a shorter tail with fewer caudal vertebrae (typically 15–16, compared to 15–22 in pine martens), which barely extends beyond the hind feet [(Monakhov 2011; Monakhov 2022)](https://www.zotero.org/google-docs/?lhN45a). Their coat is more variably colored and often darker, with high-contrast banding of down hair, and the upper neck, ears and face tend to be paler. The throat patch is often reduced or completely absent, unlike pine martens, where it is normally prominent [(Yurgenson 1947; Pavlinin 1963)](https://www.zotero.org/google-docs/?7H5Rzg). Additional features inherited from sables may include shorter and darker guard hairs at the tail tip, paler face, and shorter guard hair on the rump and hind limbs. In winter, like the sable, kidases may develop elongated bristle hairs on their feet that facilitate movement in deep snow, as well as dense fur covering the footpads and toe pads, which provides protection against cold.

While sable and pine marten show roughly similar head-body lengths in both males and females [(Monakhov 2011; Monakhov 2022)](https://www.zotero.org/google-docs/?tLhoB6), a morphometric study showed that kidases occupy an intermediate position between the parental species, consistent with first-generation hybrids (F1), although the classification accuracy was limited to 62.5% [(Monakhov and Uspenskaya 2013)](https://www.zotero.org/google-docs/?OGraud). Notably, the sable was considered vulnerable to genetic swamping due to this introgression. To improve identification, Monakhov (2021) introduced a craniometric index Δ (delta), defined as the distance from the postorbital constriction to a line connecting the postorbital processes in the sagittal plane [(Monakhov 2021a)](https://www.zotero.org/google-docs/?kEwlmH). This index allowed for highly accurate discrimination between sables and pine martens, with over 97% classification accuracy. However, it remains unclear whether this index can reliably identify hybrids or assess the extent of introgression, prompting the need for molecular genetic analyses.

# 2. Supplementary Results and Discussion

## 2.1. Samples and ranges

Our dataset included 33 marten samples (Supplementary Table ST1), 30 of which were sequenced as a part of this study. After classification 9 have been assigned as pure pine martens, 13 as pure sables and 11 as hybrid/introgressed individuals, i.e. the final groups of pure species were of similar size. For the sable, we sequenced at least one individual per sampled population, and covered most of its geographic range from Khanty-Mansi Autonomous Okrug – Yugra in the west to the Kamchatka peninsula in the east. For the pine marten, fewer samples were available, which limited our study to the Eastern part of its range. The hybrid samples mostly (8 of 11, except samples T18, T151, S50) were gathered from a single location (Tyumen Oblast, Malyi Narys) in the sympatric zone. T18 originated from another point within the zone of sympatry (Khanty-Mansi Autonomous Okrug–Yugra, Peregrebnoe). T151 was collected in the very Eastern part of the pine marten range, but outside of the overlap with sable area according to the IUCN (2024-02) [(Herrero et al. 2015; Monakhov 2015)](https://www.zotero.org/google-docs/?KAdpN9). The ranges for both species were assessed by IUCN in 2015 [(Herrero et al. 2015; Monakhov 2015)](https://www.zotero.org/google-docs/?2aD4pI) and, probably, are already outdated as it has been reported that sable has increased in abundance and is migrating at least in the northern [(S.N. Kashtanov et al. 2022)](https://www.zotero.org/google-docs/?RjP5TJ) direction out of its range. The status of the western border of the range is unclear.

The origin of the last hybrid sample (S50) is mysterious. It is the most Western among all the samples, and was gathered deep inside the area of the pine marten (Kaluga Oblast, Kaluga). Given the high fraction of the pine marten (only 16.47% of the sable), we consider this individual as a descendant (at least second or higher generation or backcross) of an escapee from a fur farm. Such fugitives were previously reported to even reach Finland, and are considered there as invasive aliens [(Partanen et al. 2020)](https://www.zotero.org/google-docs/?hLXZ3T).

## 2.2. Phylogeny and dating

We reconstructed and dated the phylogenetic tree of 21 Caniformia species using Maximum Likelihood (ML) (Additional Figure AF1A), Bayesian (Additional Figure AF1B) and coalescent-based (Additional Figure AF2, AF3; Additional Table AT1) methods and 5,989 single-copy genes (codon alignment 9,707,517 bp). We found no discrepancy in topology between ML and Bayesian trees, but on the coalescent tree the *Meles meles* was placed as an outgroup to all other included Mustelidae species instead of sister position to Lutrinae + Mustelinae. However, such a topology has a low quartet support (only 40%) close to the first alternative (36%), and the effective number (EN) of genes used to resolve the branches is low as well (2563.92 or 42.81%). On the ML and Bayesian tree, the related node has a slightly reduced bootstrap support (83) and posterior probability (95), respectively.

The fossil-based calibrated time trees are highly dependent on reliable fossil calibrations and a proper choice of molecular clock model. Our phylogenetic reconstruction revealed a high heterogeneity in substitution rates within Arctoidea and even within Mustelidae. Such a pattern forces usage of the independent rate model and requires multiple fossil calibrations ideally covering most of the nodes, which is unrealistic. This forced us to remove all lineages with either too low (all except *Ailurus fulgens* and Mustelidae) or too high (Mustelinae) substitution rates. Two fossil records (see *Fossil calibrations*) dating the split between Ailuridae and Mustelidae from both sides were an additional argument to retain *Ailurus fulgens*. It resulted in a reduced tree of 9 species (MT Figure 3), which was used to date divergence times between *Martes* species.

## 2.3. Fossil calibrations

For our set of nine species we constrained five nodes. Nearly all of them were limited either only from the lower boundary, or the upper boundary got a very relaxed geostratigraphic restriction (Supplementary Table ST3, Figure 1). The only exception was the root node, which we have constrained from both sides.


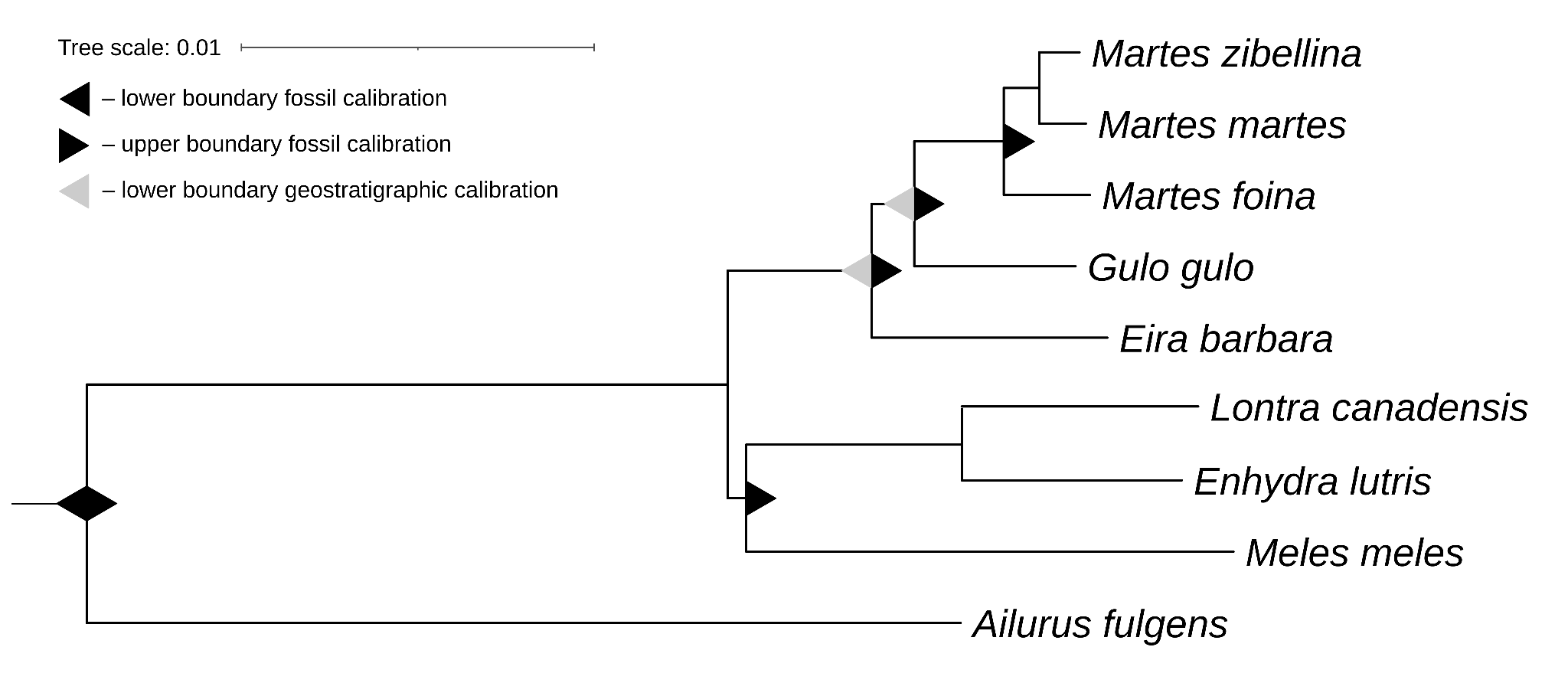


**Figure 1**. Fossil calibrated nodes.

*Mustelidae – Ailuridae split.* The root node of our tree was constrained from both sides. As the elder restriction we have used *Amphicticeps dorog* [(Wang et al. 2005)](https://www.zotero.org/google-docs/?UPBpZz), as the younger one (30.9 Mya) – *Mustelictis olivieri* (30.9-32.8 Mya) [(Wang et al. 2005)](https://www.zotero.org/google-docs/?JNZpnf), respectively. The first calibration was considered as the stem Arcoidea in previous publications, the second one - as a sister lineage to the extant and extinct Mustelidae [(Wang et al. 2005; Law et al. 2018)](https://www.zotero.org/google-docs/?zNPNyO).

*Meles – Neogale + Lutrinae split*. *Taxodon sansaniensis* [(Salesa et al. 2013)](https://www.zotero.org/google-docs/?5pXEe8) was considered by Law et, 2018 as the oldest extinct species within Melinae. We used this fossil to constrain the split between *Meles meles* and Lutrinae-Neogale lineages.

*Eira – Martes + Gulo split*. Li et al, 2014 used *Pekania occulta* [(Samuels and Cavin 2013)](https://www.zotero.org/google-docs/?hMBNRL) to set the lower boundary for split between *Pekania* and *Martes-Gulo* lineages. *Eira barbar*a (tayra) is considered to be a basal to all Mustelidae [(Koepfli et al. 2008)](https://www.zotero.org/google-docs/?hU9Nu2) species or a sister to Pekania pennanti [(Law et al. 2018)](https://www.zotero.org/google-docs/?ckaNXb). Therefore, *Pekania occulta* can be used to restrict split between *Eira barbara* and *Martes-Gulo* lineages from the lower side. As upper boundary for this node we used a geostratigrafic constraint of 20.44 Mya proposed by Li et al, 2014 [(Law et al. 2018)](https://www.zotero.org/google-docs/?Cyabtw).

*Calibrations within Martes genus (2 nodes)*. A paleontological evidence of undoubted extinct marten species is limited. Two main paleontological databases NOW (New and Old Worlds database of fossil mammals) [(Žliobaitė et al. 2023)](https://www.zotero.org/google-docs/?Bc61HY) and Paleobiology Database (PBDB; https://paleobiodb.org) contain multiple records of unrealistically old *“Martes”* species, for example, *“Martes” sansaniensis* (14-16 Mya), *“Martes” sainjon*i (16-17 Mya), *“Martes” munki* (11-17 Mya), *“Martes” laevidens* (17-19 Mya) and others. Such datings make the assignment of these fossils to the *Martes* genus unreliable as their ages are comparable to the epoch of early radiation of Mustelidae lineages [(Law et al. 2018; Hassanin et al. 2021)](https://www.zotero.org/google-docs/?VtC1fB). Tracing the related publication showed that even in the paleontological publications [(Nagel et al. 2009; Kargopoulos et al. 2022)](https://www.zotero.org/google-docs/?bq4mPY) the genus names of these species are mentioned in quotes or/and with ‘cf.’ abbreviation (short for confer, i.e. “compare”), for example, *“Martes” cf. munki* [(Kargopoulos et al. 2022)](https://www.zotero.org/google-docs/?56zMeg) or *Martes cf. munki* [(Nagel et al. 2009)](https://www.zotero.org/google-docs/?aWI1Zf). Therefore, only verified fossils can be used as calibrations within the *Martes* genus. There are only three trusted records so far: *Martes wenzensis* (3.3 - 4 Mya, the European locality of Węże 1, Central Poland) [(Stach 1959; Marciszak et al. 2024)](https://www.zotero.org/google-docs/?QIge2I), *Martes crassidens* (Jinyuan Cave, Liaoning Province, Northern China)[(Jiangzuo et al. 2021)](https://www.zotero.org/google-docs/?AATm5K), *Martes vetus* (1.75 - 2 Mya, European locality of Kielniki 3A, Southern Poland) [(Wolsan 1990)](https://www.zotero.org/google-docs/?MW7XHr). Nonetheless the broader dating interval (mid-earlier pleistocene), *M. crassidens* is considered to be older than *M. vetus*. However, the conservative assignments place both of them closer to the Holarctic marten group (*M. martes*, *M. zibellina*, *M. melampus*, *M. caurina* and *M. americana*) than to *M. foina* [(Jiangzuo et al. 2021)](https://www.zotero.org/google-docs/?v4j1HT). *M*. *wenzensis* is treated as the most ancient verified *Martes* species, but the precise dating is unknown yet - the current estimation is based on layers and covers early-mid pleistocene interval, i.e. (1.25 - 2.5 Mya) [(Jiangzuo et al. 2021)](https://www.zotero.org/google-docs/?pGyM7Q).

*Martes wenzensis* was previously used to calibrate the split between *Gulo-Martes* lineages (3.3 Mya, lower side) [(Li et al. 2014)](https://www.zotero.org/google-docs/?IQDvrf)*.* Given that recently found *M. crassidens* is considered to be younger, we did the same. Due to the relaxed lower boundary (1.25 Mya), *Martes crassidens* is the worse calibration for the split between *M. foina* and Holarctic martens than *M. vestus* (1.75 Mya), so we decided to use *M. vestus* to restrict this node from the lower side. The same constraint was used by Li et al, 2014 [(Li et al. 2014)](https://www.zotero.org/google-docs/?yaLvtY).

## 2.4. Divergence times and reference choice

To estimate the divergence time between the sable and the pine marten, we used clockwise sites extracted from a codon alignment (1,384,523 bp) generated with 5,989 single-copy orthologs and fossil-based calibration priors for five nodes to reconstruct a chronogram that included six other mustelid species (Supplementary Table ST3). We tested three types of molecular clocks: global, independent and correlated (Additional Table AT2). The global clock provided the youngest dating for all nodes except the root. For example, the Most Recent Common Ancestor (MRCA) of the sable and pine marten was dated at 1.35 (Confidence Interval (CI): 1.31 - 1.39) Mya versus 2.02 (CI: 1.53 - 2.58) Mya and 1.52 (CI: 1.05 - 2.06) Mya for the correlated and independent clocks, respectively. We also observed very narrow and unrealistic confidence intervals provided by the global model (Figure 2A) and decided to discard it. For the correlated (Figure 2B) and independent (MT Figure 1B) clocks we obtained very similar (~5% younger for the independent clock) datings for nodes 5, 6 and 7, whereas the discrepancy was significantly higher for the Guloninae lineage (up to 33%). Based on the distribution of the fossil calibrations and heterogeneity of substitution rates, we decided to rely on the estimates from the independent rate model (MT Figure 1B). Due to a relatively high distance with the sable and piner marten (2.8 Mya, CI: 2.11 – 3.66 Mya), usage of the stone marten as a reference might result in a reduction of mapping rate, reduce its precision, and bias downstream analysis, which will be difficult to detect. Instead we performed all the analyses twice, using both sable and pine marten assemblies as reference, and compared the results.


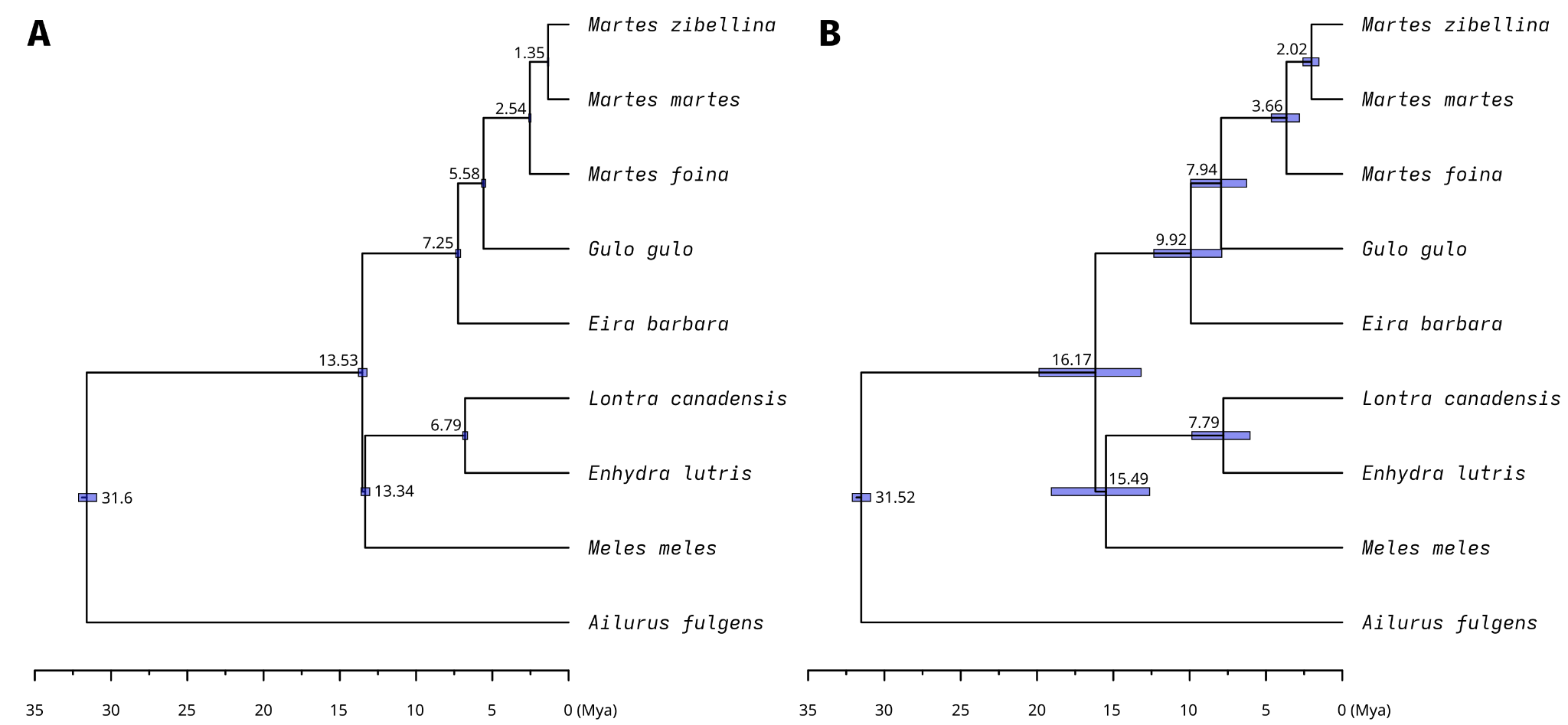


**Figure 2**. Time-calibrated phylogenetic trees for global and correlated clock models.

Types of molecular clocks: A – global; B – correlated. Branch lengths are proportional to time, with divergence dates shown in millions of years. For each node, the node ID is displayed above the branch, and the estimated divergence time (100 Mya) is shown below. Node bars indicate the 95% highest posterior density (HPD) intervals for node age uncertainty.

## 2.5. Reference-related bias

The read mapping to the sable and the pine marten references resulted in very similar mean (19.79 - 24.25x for the sable reference and 19.78 - 24.24x for the pine marten reference) and median coverage (19 - 24x for both) for all samples (Additional Table AT3). The maximal difference in mean coverage (only 0.35x) was observed for sample 10xmzib, whereas median coverage was different (1x) only for two samples: T76 and T82. We consider these differences as negligibly small. However, while comparing per-sample coverage based masking tracks (Additional Table AT4) we detected a notable reference related bias (Additional Figure AF4). We found a difference in the number of the low-coverage bases (less than 33% of the median coverage) for both pure sables and pure pine martens, which remained statistically significant (Wilcoxon signed rank test, p-value < 0.05) after adjustment for multiple comparisons and for both one-sided and two-sided tests (Table 1). For the high coverage (more than 250% of the median coverage) bases we detected a similar difference for the pure sables and hybrids (Table 1).

The difference between references remained in the unified coverage based mask (union of the pairwise intersection of per-sample masks, i.e. base is in unified mask if it was masked in at least two samples), which encompassed 118.7 Mbp for the sable reference and 130.0 Mbp for the pine marten reference. The distributions of masked bases in 1 Mbp windows with 100 kbp step showed a similar pattern for both references, but the mean and median values were different (Figure 3).

**Table 1.** Wilcoxon signed rank tests comparing the number of masked bases between the sable and pine marten references for the pure sables, pure pine martens and hybrids.

| **Individuals** | **W-test** | **p-value** | **Masked bases** | | |
| --- | --- | --- | --- | --- | --- |
|  |  |  | **all**** | **high coverage***** | **low coverage****** |
| *Pure sables* | two-sided | raw  adjusted* | **0.00024 0.00220** | **0.00171 0.01538** | **0.00024 0.00220** |
| *Pure pine martens* | two-sided | raw  adjusted | **0.00391 0.03516** | 0.03906 0.35156 | **0.00391 0.03516** |
| *Hybrids* | two-sided | raw  adjusted | 0.96582 >1 | **0.00098 0.00879** | 0.89844 >1 |
| *Pure sables* | one-sided | raw  adjusted | **0.00012 0.00110** | **0.00085 0.00769** | **0.00012 0.00110** |
| *Pure pine martens* | one-sided | raw  adjusted | **0.00195 0.01758** | 0.01953 0.17578 | **0.00195 0.01758** |
| *Hybrids* | one-sided | raw  adjusted | 0.48291 >1 | **0.00049 0.00439** | 0.44922 >1 |

** adjusted*  *– Bonferroni correction for 9 tests,*

*** all - all masked bases,*

**** high coverage – bases with coverage 2.5 times greater than the median of the whole genome,*

***** low coverage – bases with coverage 0.33 times less than the median of the whole genome.*

**Bold** highlights tests with p-value about significance level of 0.05.


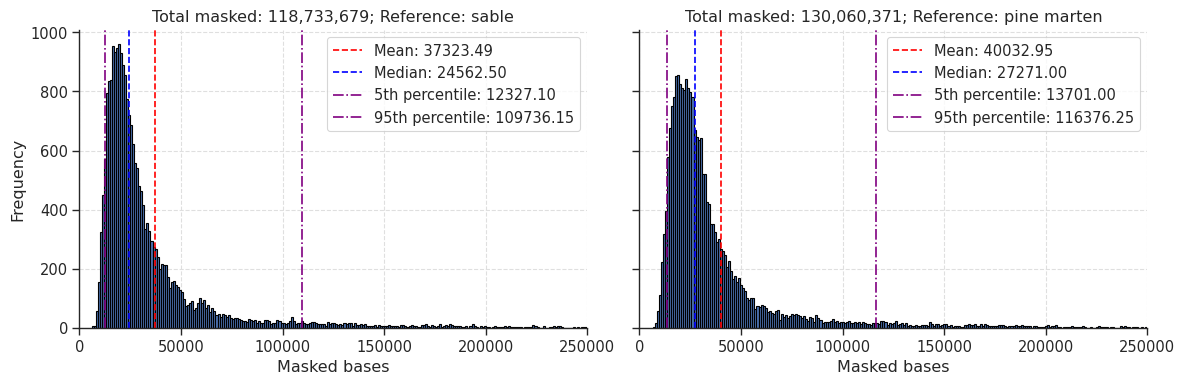


**Figure 3**. Distribution of base counts for unified coverage mask in 1 Mbp sliding windows with 100 kbp step for the sable and the pine marten references.

Before the variant calling, we identified and compared the size and coordinates of the pseudoautosomal region (PAR) in male samples using a coverage-based approach (details in the Supplementary methods). We detected only a negligible difference of 30 kbp in PAR length between the references: its length was 6.48 for the sable reference and 6.45 Mbp for the pine marten reference. In both cases PAR was located at the end of chrX as expected (Additional Figure AF5, Additional Table AT5). The variant calling followed by the quality filtering resulted in 34.8 million SNPs for the sable reference versus 34.5 million for the pine marten (0.87% difference). After applying the unified coverage masks, the counts slightly reduced to 32.5 and 32.3 million (0.62% difference), respectively. After filtering for missing genotypes, minimal allele frequency and consequent aggressive LD pruning the SNP counts remained close between references: 1.72 and 1.71 million SNPs, respectively. The shapes of the corresponding window-based distributions were highly similar, with close values of the mean (766 and 758 SNPs/kbp for the sable and the pine marten references) and median (756 and 749 SNPs/kbp) values (Figure 4).


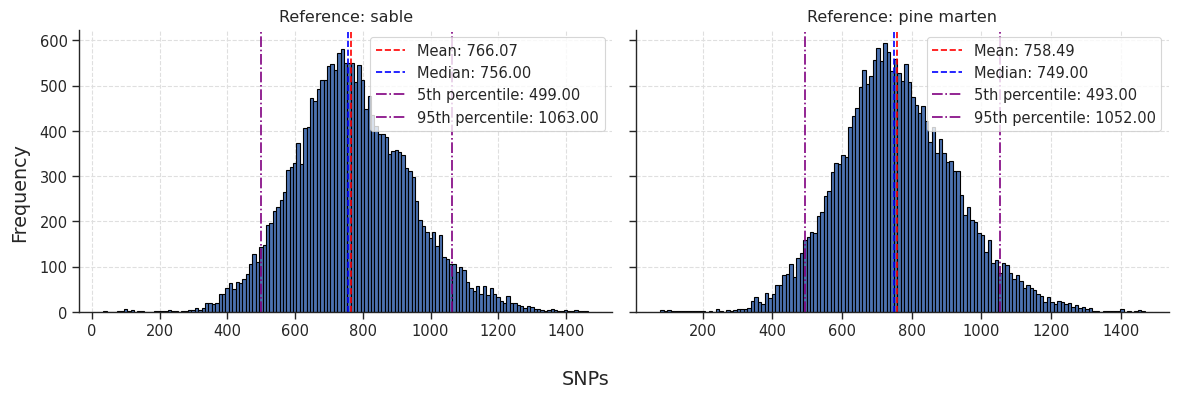


**Figure 4**. Distribution of LD-pruned SNP counts in 1 Mbp sliding windows with 100 kbp step for the sable and the pine marten references.

The PCA showed highly similar clustering patterns for both references (Figure 5; see Supplementary Figure SF2 for sample labels). Moreover, the fractions of variance explained by two first principal components are close: 36.09% (the sable reference) vs 37.10% (the pine marten reference) for PC1 and 4.51% vs 4.4% for PC2, respectively. Both global and local ancestry inferences (Figure 6) produced highly consistent results across references (Additional Table AT6) as well. The largest discrepancy (only ~1.1%) in both global and local ancestry proportions was observed for sample T87. The distributions of heterozygous SNPs and subsequent heterozygosity component analysis (see *Concept of the heterozygosity component analysis (HCA)* below) also demonstrated highly similar patterns across all samples (Figure 7, Additional Figure AF6-AF8, Additional Table AT7, Supplementary File 5).

RoH analysis potentially is vulnerable to the reference-related bias introduced by an elevated rate of false-positive heterozygous variant calls when a cross-species reference is used. In our dataset, such biases are expected to affect pine martens genotyped against the sable reference and, conversely, sables genotyped against the pine marten reference, while the magnitude and direction of the effect in hybrids should be variable. Consistent with these expectations, we observed a pronounced reference-related bias for ultra low heterozygosity (<0.1 hetSNPs/kbp) windows (Figure 8A). Specifically, in sables genotyped against the pine marten reference the peak corresponding to the RoH component was shifted from values near 0 hetSNPs/kbp to the slightly higher values compared with the variant calling performed against the sable reference. In contrast, the pine martens displayed the opposite pattern, as expected. Nevertheless, the threshold of 0.05 hetSNPs/kbp (Figure 8A, vertical dashed red line), which has been applied in several of our previous studies [(Kliver et al. 2023; Yakupova et al. 2023; A. Tomarovsky et al. 2025; Totikov et al. 2025)](https://www.zotero.org/google-docs/?YzmFBM) appears to be robust and insensitive to the observed reference-related bias. To verify whether this bias propagates to the downstream stages of the ROH detection pipeline (*get_ROH_regions.py* script from the Biocrutch v1.0 package (<https://github.com/tomarovsky/Biocrutch>)), we compared RoH identified using the pine marten and sable references, but obtained highly similar cumulative ROH lengths distributions. (Figure 8B, Additional Figure AF9, Additional Table AT8). The largest difference in total RoH content between the sable and pine marten references was observed for the sample 10xmmar (1.7%).

Finally, the comparison of PSMC demographic trajectories (Figure 9) revealed only negligible differences between references, which are related to small temporal shifts (along the X-axis) rather than to significant changes in the inferred demographic patterns.

We mined known informative STR loci for the Mustelidae family from literature [(Davis and Strobeck 1998a; M.A. Fleming et al. 1999; Domingo-Roura 2002; Vincent et al. 2003a; Basto et al. 2010a; Natali et al. 2010)](https://www.zotero.org/google-docs/?tBOSjC) and got a candidate set of 79 markers (Supplementary File 11). Out of them we found that only 36 loci for the sable reference and 44 for the pine marten reference are suitable for reliable genotyping from our 150 bp reads (Supplementary File 2, see Main Text Methods for detailed description). For Rozhnov’s (9 STR loci, [(Rozhnov et al. 2013)](https://www.zotero.org/google-docs/?wNaBD7)) subset we localize 7 markers for the sable reference and 8 for the pine marten reference, whereas for Kashtanov’s (16 STR loci, [(S.N. Kashtanov et al. 2022)](https://www.zotero.org/google-docs/?LkhRXu)) – 13 and 14, respectively. Notably, all the markers found in the sable reference were detected in the pine marten reference too. We calculated and compared the STR-based ancestry between references: **(A)** for all sable (36) markers vs all pine marten (44) markers (Additional Table AT9, All STRs), **(B)** for sable markers (7) vs pine marten markers (8) for Rozhnov’s subset, (Additional Table AT9, Rozhnov’s) **(C)** for sable markers (13) vs pine marten markers (14) for Kashtanov’s subset, (Additional Table AT9, Kashtanov’s), **(D)** only for common (36) markers (Additional Table AT9, All STRs), **(E)** only for common (7) markers from Rozhnov’s subset (Additional Table AT9, Rozhnov’s), **(F)** only for common (13) markers from Kashtanov’s subset (Additional Table AT9, Kashtanov’s). The highest difference between references was detected for samples T84 (comparison B, 11.7 % difference), T85 (comparison B, 7.4 %) and sample T87 (comparison C, 10.4 % difference) (Additional Figure AF10). In case of comparisons D, E and F, the maximal difference didn’t exceed 4.9%. We explain these notable discrepancies by two reasons. First, the low number of STR markers, which makes this analysis sensitive even for a single marker difference between compared sets (7 vs 8 markers in comparison B). Second, reliable STR genotyping from NGS reads requires a whole STR marker to be located within a single read, notably reducing the effective coverage of STR loci compared to the whole genome coverage. Thus, even in a case of exactly the same set of markers (comparisons D, E and F) the analysis is sensitive to the alignment rate, and as consequence, to the reference-related bias. However, despite being notable, this difference didn’t affect the conclusions of the manuscript. First, it is small, < 5% for the same sets of markers compared between references (Additional Table AT10), and reaches 5% only for three samples: T84, T87 and T85. Second, for “All STRs” subset it didn’t change the classification of samples as pure species or hybrid at all, and for Roznov’s and Kahtanov’s sets it affected only three and one samples, respectively (see coloration of the Additional Table AT10).


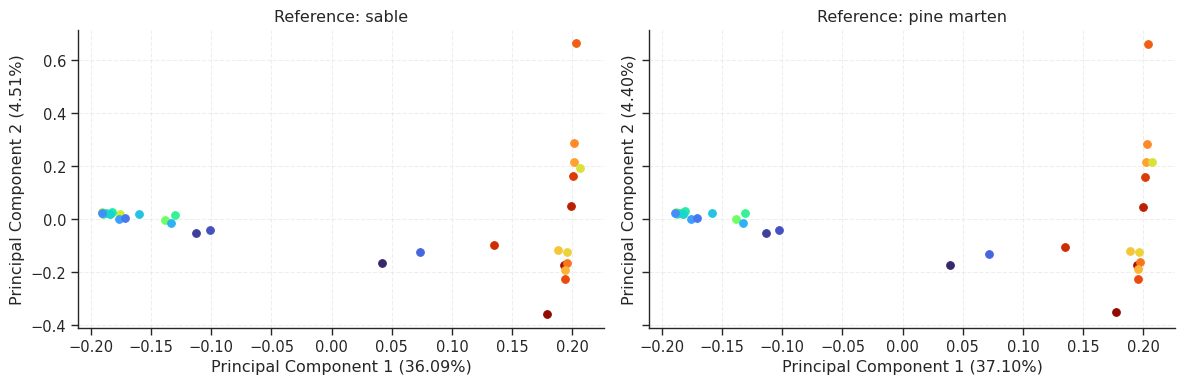


**Figure 5.** Comparison of the PCA results for the sable and pine marten references


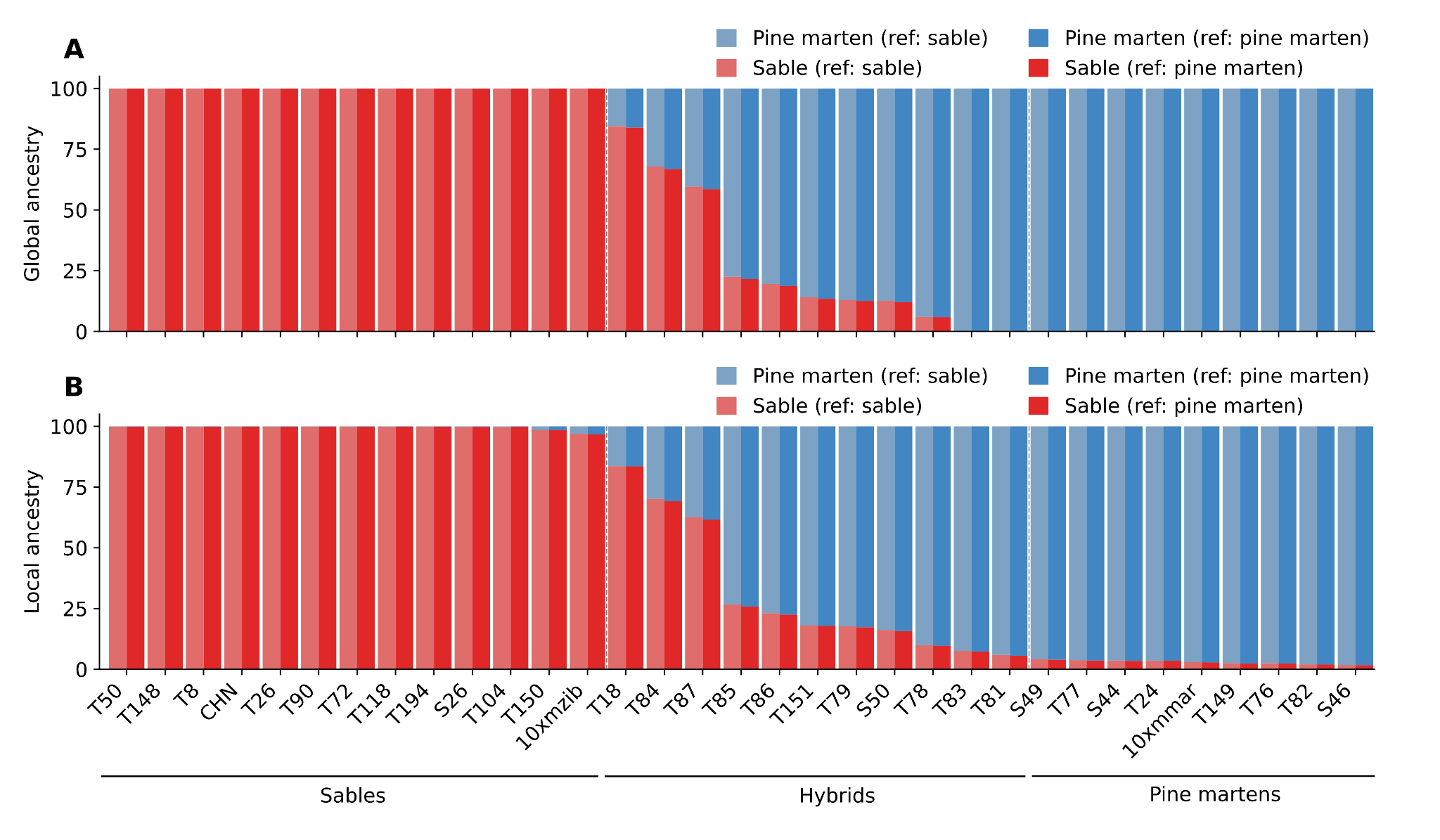


**Figure 6.** Comparison of the global (A) and local (B) ancestry for the sable and pine marten references


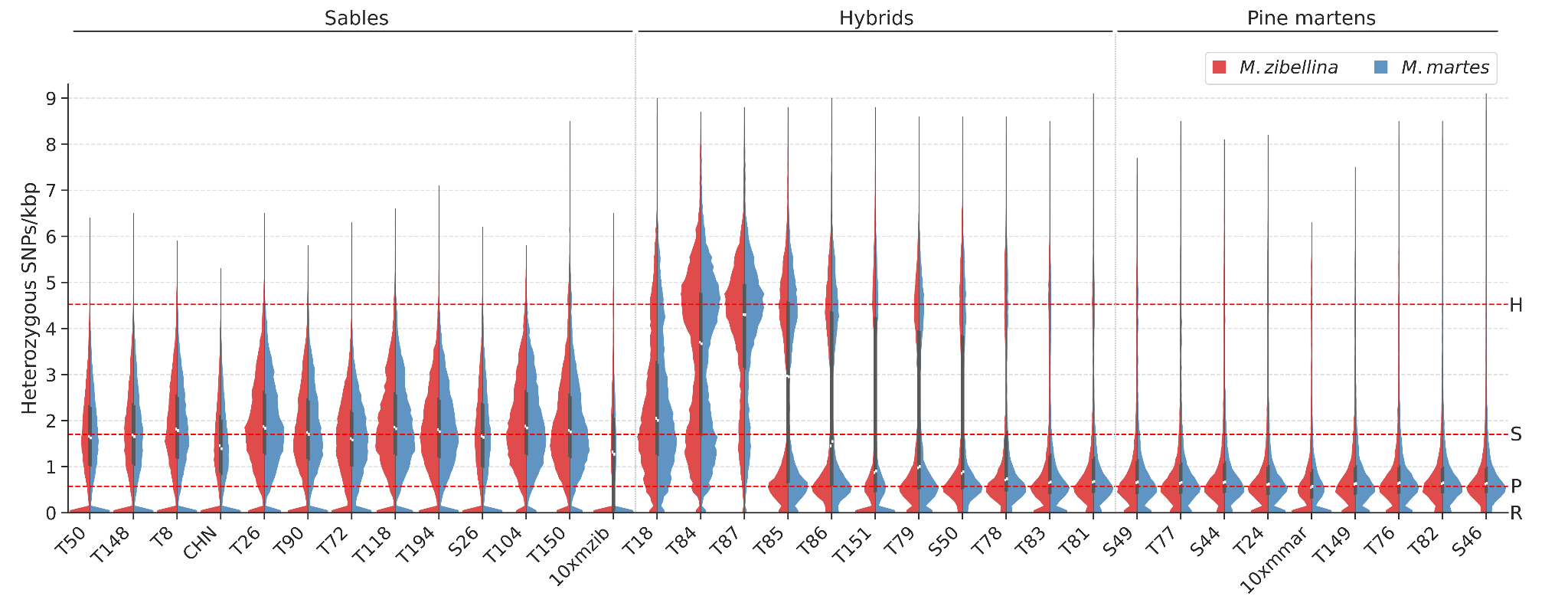


**Figure 7.** Comparison of per-sample heterozygosity distribution for the sable (left half) and pine marten (right half) references


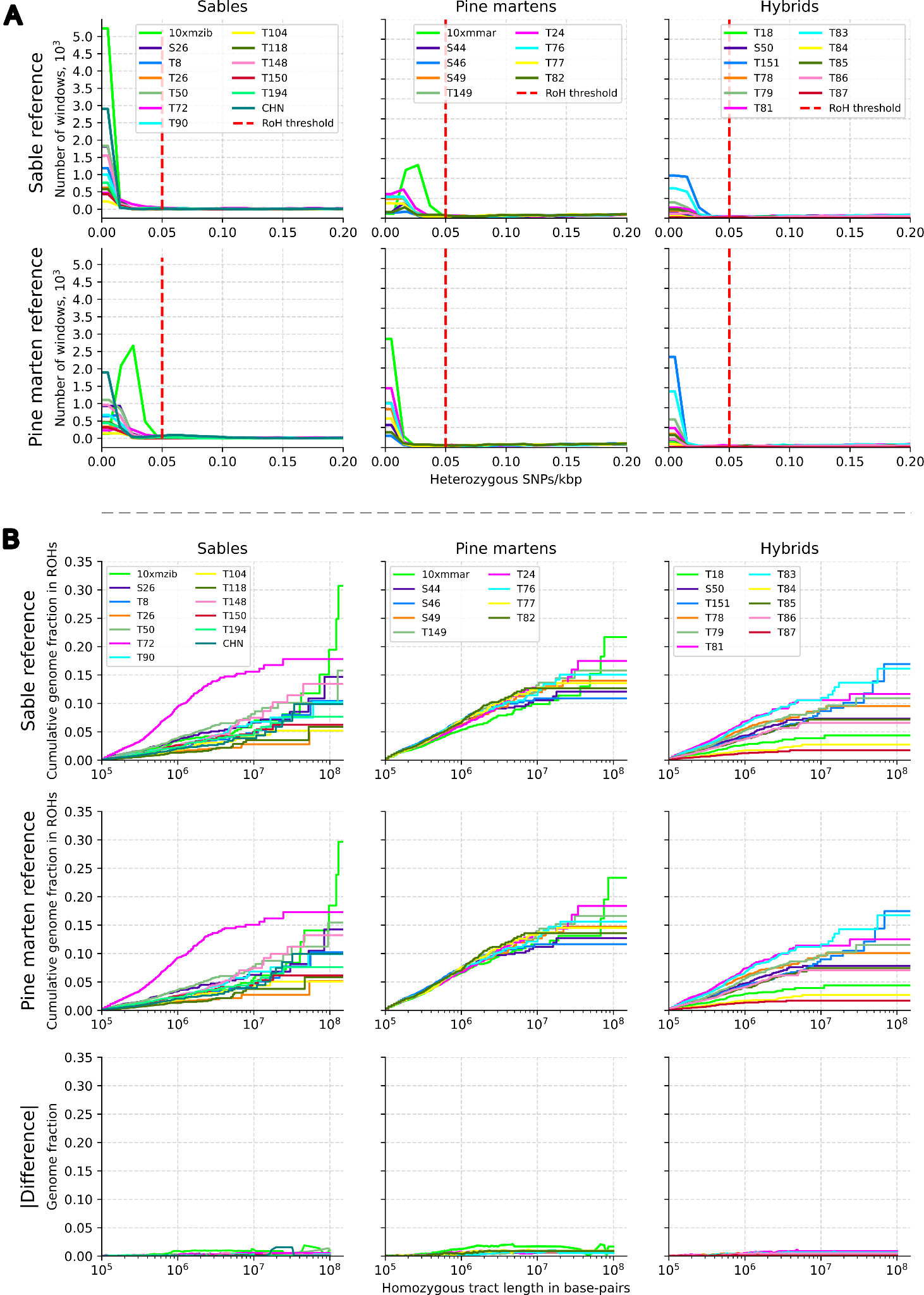


**Figure 8.** Reference-related bias and Runs of the Homozygosity (RoH)

A - reference-related bias in context of the threshold selection for the RoH window. Red dashed line depicts selected threshold of 0.05 hetSNPs/kbp; B - comparison of cumulative RoH length distributions for the sable and pine marten references


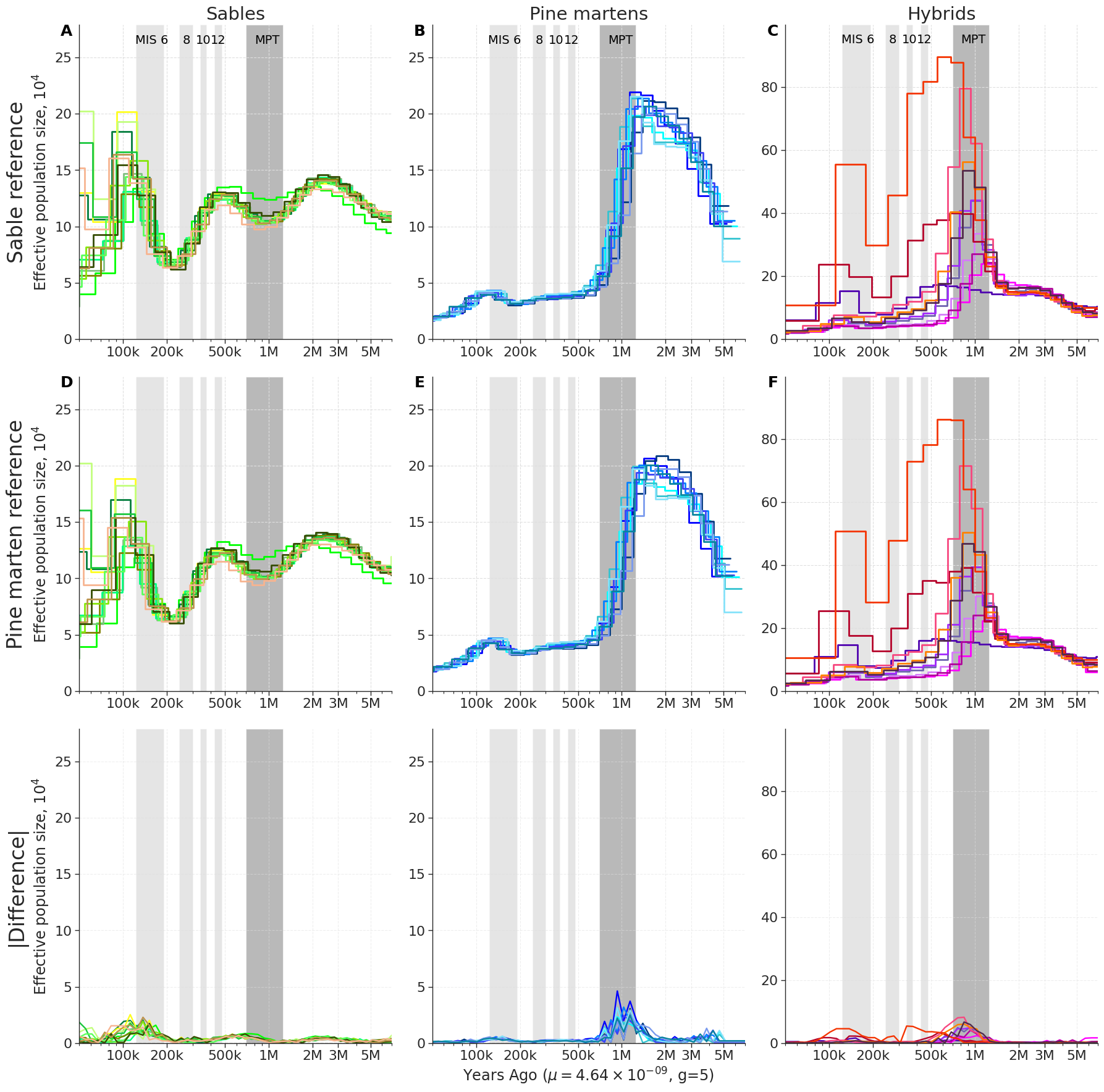


**Figure 9.** Comparison of PSMC demographic trajectories for the sable and pine marten references

## 2.6. Concept of the heterozygosity component analysis (HCA)

Mean and median heterozygosity are significantly affected by RoH and introgression and, therefore, provide only a “summary” estimate. A common way to step over this issue is to use a window-based approach, which provides a distribution of estimates in each of the windows instead of a single value (MT Figure 6 and 7A). It allows to study distribution of heterozygosity along the chromosomes (MT Figure 6B, Supplementary File 6), identify regions outlying by derivative metrics like Fst and Tajima’s D, etc. We decided to step further and introduce a concept of a new method – heterozygosity component analysis or HCA. An inspiration for it were heterozygosity distributions of our hybrid samples and Genomescope2 tool [(Ranallo-Benavidez et al. 2020)](https://www.zotero.org/google-docs/?FrN6Oj), which uses a similar approach, but for a different purpose (genome size estimation). Briefly (see Supplementary Methods for details), the basis of our method is a fitting of a linear combination of negative binomial distributions to the empirical distribution of the heterozygosity. We successfully decomposed distributions into the combination of pure pine marten (P), pure sable (S) and hybrid (H) components for all our samples (Figure 10, MT Figure 6A). Mean of the P-component was very similar among all samples containing it (0.536 - 0.763, σ = 0.047) and for pure pine martens (0.559 - 0.576, σ = 0.008) was close to the global median values (0.56 - 0.67, orange bars on Figure 10). However, the notable difference with the global mean values (0.84 - 1.07, light orange bars on ST Figure 10) is easy to explain by a small introgression from the sable to our pure pine marten samples. Because of high values of H-component (4.17 - 4.51, σ = 0.11) and low values of P-component (green dashes on Figure 10), global mean values were notably biased upwards by a small number of outlying hybrid windows. Presence of the small H-component (red vertical dashes on ST Figure 9) in pure pine martens was confirmed by the local (but not the global) admixture analysis (MT Figure 4D). Therefore, we have confirmed the robustness of our method and one more time highlighted that a single global mean value is a bad metric even in a case of low introgression.

After some additions (automatic detection of the starting parameters) and optimizations (better fitting procedure), our method can be used as a test for hybrid origin of the sample if no samples of pure species are available, or their number is not enough for a reliable Admixture/HyDe/D-statistic analysis. Principal limitation for such an application is a presence of a notable number of windows with significantly different heterozygosity, so it, for example, will not work for F1-hybrids, but should work for F1-like, as our sample T87 (MT Figure 6A). As a weak point of our method we have to mention that it is yet unclear at what degree imprecision of distribution fitting affects the values and if its contribution is comparable (or not) to biological variation among the samples.


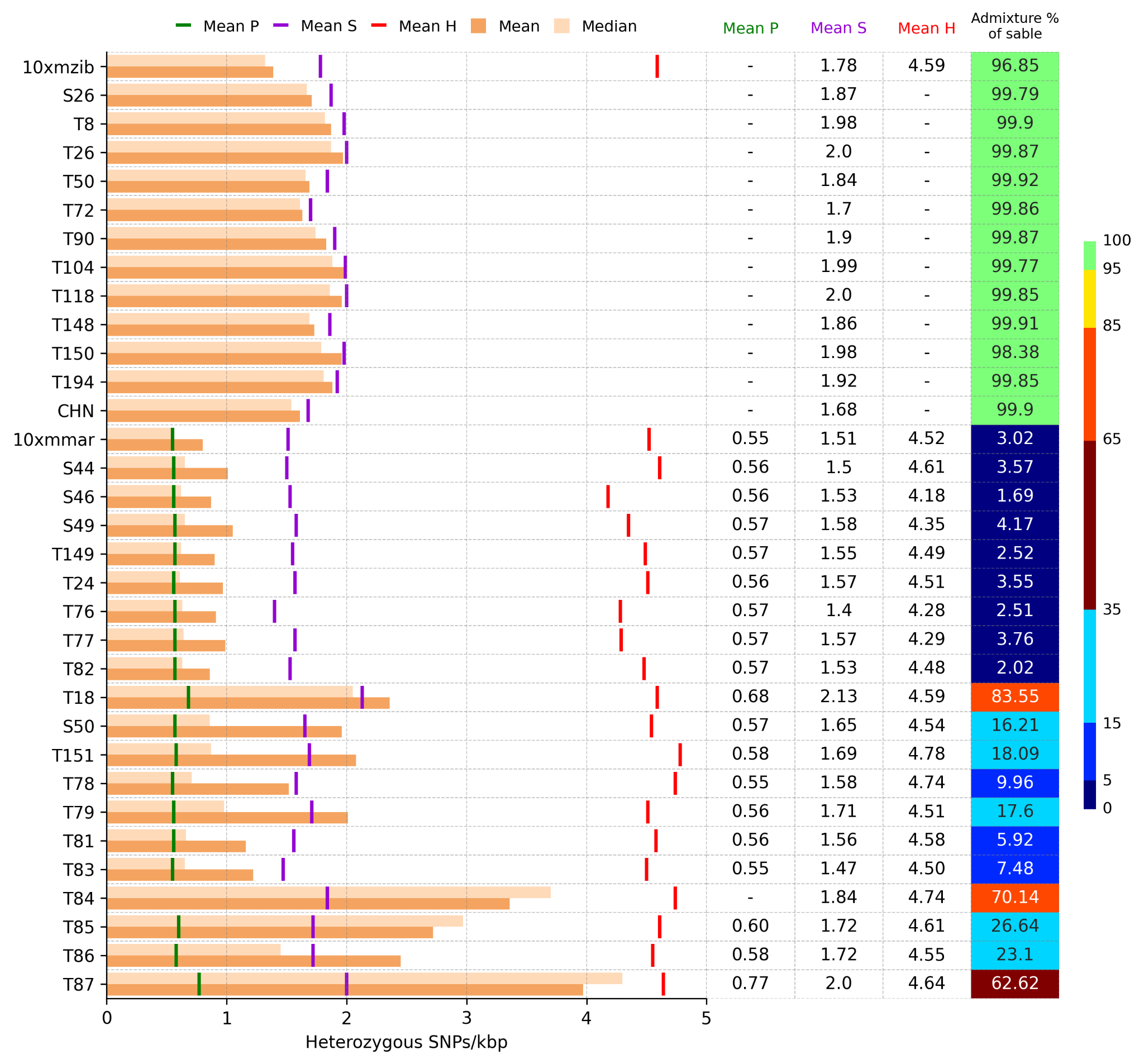


**Figure 10**. Heterozygosity Component Analysis (HCA)

The histogram displays mean (orange) and median (light orange) global heterozygosity across samples. Vertical dashes indicate the mean component values: green (pine marten, P), purple (sable, S), and red (hybrid, H). Absolute values of the P, S, and H components, along with admixture (%) from sable, are shown on the right

## 2.7. Classification of individuals by morphology

It is believed that the kidases (hybrids *M. martes* × *M. zibellina*) can be identified by the fur coloration and the relative length of the tail [(Pavlinin 1963)](https://www.zotero.org/google-docs/?WOChLG). In most cases, the coloration of kidas is similar to the color of the pine marten: the head is darker than the back (in sable, it is lighter), the belly is evenly colored, the throat spot is usually absent or represented by separate small spots. The pine marten has a long tail, more than half the length of the body. Usually it protrudes more than 1/4 of its length beyond the ends of the hind limbs extended backwards. The tail of the sable is relatively shorter; it protrudes slightly beyond the ends of hind legs. The tail of the kidas is of medium length. Based on the exterior features, all the studied specimens can be attributed to Martes martes, except for specimen T87, which can be identified as a hybrid (according to the color of the throat).

Many characters were used to distinguish the skulls of *M. zibellina* and *M. martes* – position and size of auditory bullae, various dental characters, mandibular characters, location of the carotid fossae, etc., however all of them are not certain enough [(Pavlinin 1963; Heptner et al. 1967)](https://www.zotero.org/google-docs/?oTcyKC). Recently, Monakhov (2020) proposed an additional craniological character for the identification of sable and pine marten [(Monakhov 2020)](https://www.zotero.org/google-docs/?odaTgL). According to his data, sable and pine marten differ in the distance from the postorbital constriction to the line between the postorbital processes in the sagittal plane (character Δ) with the average value of Δ for sable being much smaller than that for pine marten. This character showed a high level (more than 97%) of correct species identifications [(Monakhov 2020)](https://www.zotero.org/google-docs/?ELarnl). Based on this character our putative hybrids were closer to *M. martes* than to *M. zibellina*. However, skull morphology of hybrids in Mustelidae is poorly studied. Usually, F1 hybrids look craniologically similar to one of the parental species. Comparative analysis of the skulls of European mink *Mustela lutreola*, polecat *Mustela putorius* and their hybrids from the north-western part of Russia showed that most of the hybrid specimens were craniologically very similar to *M. putorius* [(Abramov and Tumanov 2003)](https://www.zotero.org/google-docs/?p7aedw). Skulls of hybrids between European and Asian badgers, *Meles meles* and *M. leucurus*, look more similar to *M. leucurus* [(Abramov and Puzachenko 2007)](https://www.zotero.org/google-docs/?Lcco2O).

Among our samples, skulls and pelts were available only for putative hybrids except T24 and T86, i.e. for T77, T78, T79, T81, T82,T83, T84, T85 and T87. All of them came from animals that had long tails protruding far beyond the hind legs. The throat patches were large, yellowish-red or white. These features are characteristic of the pine marten. Only the specimen T87 had the smaller throat patch, which also was divided into several blotches. The heads of the examined putative hybrids were concolorous with the body, a feature typical for the pine marten, while the sable usually has a paler upper neck, ears and face. Multivariate analyses of craniometric characteristics failed to identify the species status of putative hybrids. They were morphologically closer to *M. martes* than to *M. zibellina*. According to Monakhov (2020), the average morphometric character Δ for sable is 4.67 ± 0.07, whereas that for pine marten is 7.81 ± 0.09. Our putative hybrids have a Δ = 6.82 (5.76 - 9.37), while specimen T87 has a Δ = 7.1.

## 2.8. Issues of dating demographic trajectories

Dating of the demographic trajectory is always a difficult task due to difficulties with reliable estimation of generation time to the mutation rate ratio (g/*μ*). As a reference value for the *μ* used 4.64 × 10⁻⁹ substitution per site per generation, measured from the trio of American minks [(Bergeron et al. 2023)](https://www.zotero.org/google-docs/?BJaZAF). Borders of its confidence interval (CI: 2.94 × 10⁻⁹ - 7.37 × 10⁻⁹) we used to calculate CI for our datings. We have to note that Bergeron et al have sequenced and analyzed only a single mink trio for the estimation, and CI was inferred from a negative binomial distribution. American mink belongs to a different subfamily (Mustelinae) and our initial phylogeny reconstruction showed significantly longer branches for Mustelinae than for Guloninae (Additional Figure AF1A and B). Therefore, mink mutation rate might not be a good estimate for marten species, but no better estimates are available. For g we found no way to estimate a CI and used a fixed g=5. So the imprecision of the generation time is not included in the CIs of our datings.

In hybrid samples (MT Figure 8B) we found a specific pattern – a steeply and high peak, which can be used as an indicator of notable introgression. Moreover, we detected a strong and significant correlation (Kendall’s τ = 0.67, p-value = 0.003; Spearman’s ρ = 0.76, p-value = 0.006; Pearson’s r = 0.778, p-value = 0.005) between height of the peak (Supplementary Figure SF8) and admixture level. It suggests that the ratio of its heights in two or more samples can be used for comparative quantification of the admixture in terms more/less. The differences in trajectories observed in hybrids are likely related not only to the number of hybrid segments but also to their merging [(Cahill et al. 2016)](https://www.zotero.org/google-docs/?me9O0Q).

## 2.9. Divergence dating from demographic trajectories

The demographic trajectories also provided some clues for dating the divergence between sable and pine marten. It should occur between the start of the N_e_ decline in the pine marten and the end of the shared with the sable trajectory. Using the mutation rate *μ* = 4.64 × 10⁻⁹ [(Bergeron et al. 2023)](https://www.zotero.org/google-docs/?rN2lja), the divergence event is estimated to have occurred between 1.3 and 3.5 Mya. Taking a more conservative approach and incorporating the confidence interval boundaries of the mutation rate (2.94 × 10⁻⁹ – 7.37 × 10⁻⁹) expands this estimate considerably to between 0.82 and 5.52 Mya. However, the divergence of demographic trajectories is expected to precede the actual speciation event by a substantial margin. We previously observed similar demographic trajectory differences within the least weasel (comparable to the 1.3-3.5 Mya divergence between sable and pine marten), yet no speciation occurred in that case [(Totikov et al. 2025)](https://www.zotero.org/google-docs/?6PuU9x). This suggests that the upper boundary estimates of 3.5 Mya, and particularly 5.52 Mya, represent substantial overestimates. Alternatively, if we assume that isolation during the Mid-Pleistocene Transition was the primary driver of speciation and apply the mutation rate confidence interval boundaries to the 1.3 Mya estimate, we obtain a more reasonable confidence interval of 0.82-1.97 Mya.

## 2.10. Speculations on migration routes and speciation

Currently, martens *sensu stricto* (subgenus *Martes*) are distributed across the Northern Hemisphere. The sable is an Asian species whereas the pine marten is mostly European (but it penetrates into Asia). The American marten (*M. americana*) and the Pacific marten (*M. caurina*) range in Northern America, whereas the Japanese sable (*M. melampus*) inhabits the Japanese archipelago. Given the distribution and relatively stable (but oscillating) trajectory of the sable, we assume that the MRCA of pine marten and sable inhabited an area in Northern Asia close or partly overlapping with the current range of the sable. Therefore, the expansion of pine marten ancestors should have been towards Europe. However, the evolutionary history of sable-pine marten interactions is likely more complex than simple ancient divergence followed by recent hybridization. The Mid-Pleistocene Transition changed not only the period of glacial-interglacial cycles, but also significantly increased the amplitude of temperature changes [(Pisias and Moore 1981; Clark et al. 2006; Bajo et al. 2020)](https://www.zotero.org/google-docs/?tI5Iss). The high vagility of martens [(Zalewski et al. 2004)](https://www.zotero.org/google-docs/?ThbsIU) suggests that under such conditions, species ranges should be highly dynamic, fluctuating from small refugia to vast areas, with cold-adapted species gaining substantial advantages. The evolutionary processes after divergence from the MRCA have shaped the pine marten and the sable differently. The modern sable is considered to have greater adaptability to cold habitats, but the cost was a reduced tolerance to hot summers [(Monakhov 2021b)](https://www.zotero.org/google-docs/?0fvmnH). Some researchers even suggest that during the cold (but not glacial) periods of the Pleistocene, sable temporarily expanded westwards, even reaching Fennoscandia, and had retracted back to the Siberian forests during warmer times. In contrast, the pine marten was hypothesized to replace the sable during warm interglacial periods [(Davison et al. 2001; Stojak and Jędrzejewska 2022)](https://www.zotero.org/google-docs/?VkedUG). However, both species share a broad temperature range optimal for both, suggesting that multiple hybridization events likely occurred during periods of favorable climatic conditions. Thus, the current ranges of the sable and pine marten most likely are the result of multiple recolonization processes shaped by Pleistocene climatic fluctuations, which led to the formation of ancient and modern zones of sympatry and hybridization after the two lineages had diverged.

## 2.11. Candidate genes related to phenotypic and diet differences

To detect the most differentiated loci between the pure sables and pure martens, we calculated Fst (pure sables vs pure pine martens) and Tajima’s D (all samples as a single population) statistics in the sliding windows of 1 Mbp with a step of 100 kbp based sable genome assembly. Among 14 high Fst (>=0.9) regions we found three loci (FST4, FST6, FST13) enriched by proteins with specific GO terms (Supplementary File 9). The most interesting region, FST13 (chr9: 86,600,000–88,100,000), containing 48 genes, revealed a statistically significant enrichment in four GO categories: keratinization (GO:0031424), intermediate filament organization (GO:0045109), embryonic skeletal system development (GO:0048706) and anterior/posterior pattern specification (GO:0009952). Further examination of the gene list showed that it includes a cluster of HOX-C genes (homeobox C cluster) and ten cytokeratin type I and II genes: KRT18 (type I) and KRT80, KRT7, KRT81, KRT76, KRT3, KRT4, KRT79, KRT78, KRT8 (type II).

Tajima’s D (all samples as a single population, Figure 11A and D) metric revealed only a single region (TJD1) with significantly high value (D>2). It is located on the chr1, encompasses 1.5 Mbp and is nested in the high Fst loci FST1 (Supplementary Table ST7). Although no GO enrichment was found for this region, among its 23 genes is FGFR3, which plays a key role in bone development and maintenance (Supplementary File 9). We also calculated Tajima's D in 1 Mbp windows with a 100 kbp step separately for pure sables and pure pine martens (Figure 11B, E, C, F). The mean values were -0.42 for sables and -0.32 for pine martens. This analysis revealed 15 regions (TJD2–TJD16) with significantly negative Tajima’s D values (D < -2). Of these, five (TJD3, 7, 9, 11 and 14) showed enrichment in GO terms (Supplementary File 9). One of them, TJD7, is enriched by GO:0045796 (negative regulation of intestinal cholesterol absorption) and GO:0010949 (negative regulation of intestinal phytosterol absorption), because it contains the *ABCG5* and *ABCG8* genes, which encode sterolin-1 and sterolin-2 proteins (Supplementary File 9).


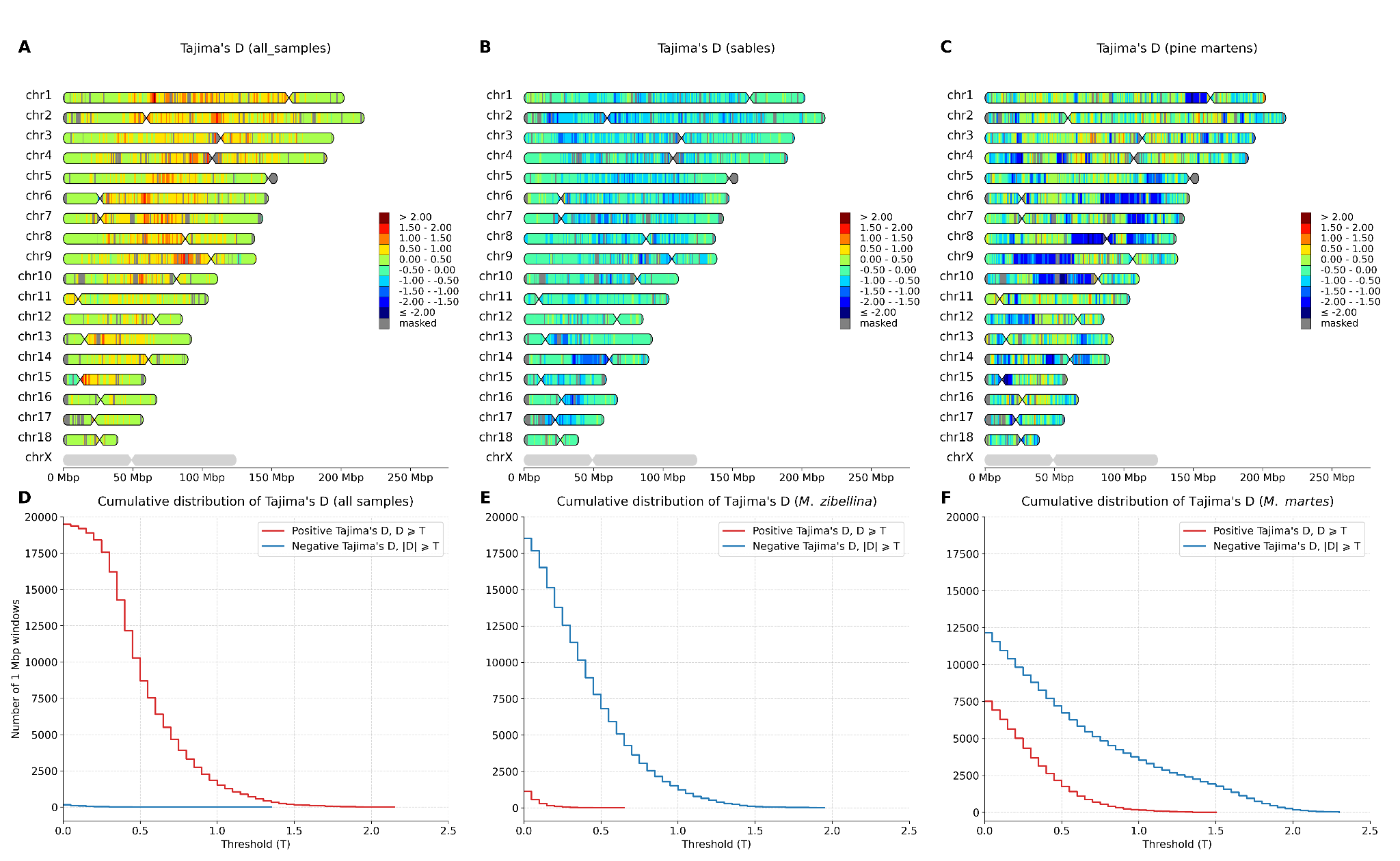


**Figure 11**. Distribution of Tajima’s D values on chromosomes.
A, B, C – Tajima’s D for all samples, pure sables and pure pine martens, respectively; D, E, F – cumulative distributions of Tajima’s D for the same groups. Tajima’s D values were calculated in the sliding windows of 1 Mbp with a step of 100 kbp

The previously described and cytogenetically confirmed inversion (11.5 Mbp, nearly the whole p-arm of chr11) between the sable and pine marten [(A.A. Tomarovsky et al. 2025)](https://www.zotero.org/google-docs/?crpkAE) in addition to two reproduction-related genes, *SPMIP7* and *ZPBP (*see section *“Fertility related genes within the inverted p-arm of chr11”* in the main text*),* also contains *GLI3*, a gene involved in developmental processes (Supplementary File 9). This gene is known to participate in the regulation of limb and skeletal development [(Kalff-Suske et al. 1999; Wang et al. 2024)](https://www.zotero.org/google-docs/?hA2DKs). In humans, the associated disorders include acrocallosal syndrome, characterized by distinct craniofacial features such as hypertelorism (widely spaced eyes) and a prominent forehead [(Elson et al. 2002)](https://www.zotero.org/google-docs/?cgOULq), which are similar to the skull differences between the sable and the pine marten [(Monakhov 2021a)](https://www.zotero.org/google-docs/?NnPL5j). This suggests that *GLI3* may be a promising candidate to study the genetics of morphological evolution of marten species. Other candidate genes we identified may also be associated with other phenotypic features. For example, the *FGFR3* gene, associated with craniosynostosis and multiple types of skeletal dysplasia [(Schibler et al. 2009)](https://www.zotero.org/google-docs/?CZ0pmj), might be linked to the increased vertebral number in the pine marten [(Monakhov 2011; Monakhov 2022)](https://www.zotero.org/google-docs/?9kpQRd). Similarly, the *HoxC* cluster, known for its roles in limb development [(Okamoto et al. 2011)](https://www.zotero.org/google-docs/?PoXKMK), could relate to the differences in paw size.

The two species are also known to exhibit significant dietary differences. The sable shows a flexible feeding pattern, shifting from a diet primarily composed of plant matter (nuts and berries) during years of abundant harvest to one predominantly consisting of animal prey (small vertebrates) [(Monakhov 2016; Cheprasov and Mordosov 2019)](https://www.zotero.org/google-docs/?UEFAs7). In areas with high yields of nut-producing five-needle conifers, sables rely heavily on nuts produced through windfall or cached by birds and squirrels throughout the fall and early winter [(Zakharov et al. 2016)](https://www.zotero.org/google-docs/?RpPEe6). In contrast, the pine marten does not feed on nuts in appreciable quantities, if at all [(Helldin 2000; Posłuszny et al. 2007; Twining et al. 2019)](https://www.zotero.org/google-docs/?zXbZ1c). Plant matter (mostly berries) contributes up to 50% or more of its diet in southern areas, but in most regions it is reported to be a hypercarnivore (>70% animal prey) during the year [(Zalewski 2005)](https://www.zotero.org/google-docs/?Gk4jHN). We found two genes, *ABCG5* and *ABCG8*, directly involved in the transport of dietary sterols (both cholesterol and plant sitosterols) [(Hazard and Patel 2007)](https://www.zotero.org/google-docs/?6LQGIP) overlap with regions having high negative Tajima’s D in the pure pine martens, but not in the pure sables. Previously, it was shown that simultaneous knockout of both genes in mice decreased the concentration of the cholesterol in the bile, resulting in its accumulation in the liver, as well as increased the plasma level of sitosterols [(Yu, Hammer, et al. 2002)](https://www.zotero.org/google-docs/?cKX5YG). In contrast, the overexpression of these genes increased the bile secretion of the cholesterol and halved its fractional absorption from the digestive tract [(Yu, Li-Hawkins, et al. 2002)](https://www.zotero.org/google-docs/?MhcFaM). Low Tajima’s D are typically interpreted as indicators of either negative selection or population expansion [(Carlson et al. 2005)](https://www.zotero.org/google-docs/?csMenH). Therefore, *ABCG5* and *ABCG8* are considered prime candidates for future genetic studies focusing on the dietary differences between sable and pine marten, specifically regarding the latter's adaptation to hypercarnivory.

# 3. Supplementary Methods

## 3.1 Phylogeny reconstruction

The phylogenetic tree was reconstructed using the BuscoClade pipeline v1.7 (https://github.com/tomarovsky/BuscoClade), based on a multiple codon alignment of conserved single-copy orthologous gene coding sequences (BUSCOs). We included genome assemblies from a total of 21 species (Additional Table AT11) [(Peng et al. 2014; Foote et al. 2015; Hu et al. 2017; Jones et al. 2017; Dudchenko et al. 2017; Dudchenko et al. 2018; Taylor et al. 2018; Miranda et al. 2021; Peng et al. 2021; Newman et al. 2022; Karimi et al. 2022; Lok et al. 2022; Mohr et al. 2022; Derežanin et al. 2022; Kliver et al. 2023; A. Tomarovsky et al. 2025; A.A. Tomarovsky et al. 2025)](https://www.zotero.org/google-docs/?om8XsU). BUSCO sequences were identified with BUSCO v5.4.2 [(Manni et al. 2021)](https://www.zotero.org/google-docs/?saAw0M) using the database Mammalia_odb v10, 2021-02-19. Only single-copy sequences common for all species were used in the alignment. The codon alignments were performed separately for each sequence using PRANK v170427 [(Löytynoja 2014)](https://www.zotero.org/google-docs/?EzaU1e), followed by a filtration of hypervariable and poorly aligned regions using GBlocks v0.91b [(Castresana 2000)](https://www.zotero.org/google-docs/?dkpxvf). The resulting filtered alignments were concatenated, yielding a final alignment of 9,707,517 bp. Phylogenetic inference was performed using the maximum likelihood (ML) method implemented in IQ-TREE v2.2.0 [(Minh et al. 2020)](https://www.zotero.org/google-docs/?d5YDzT) with automatic selection of the best-fitting substitution model using ModelFinder [(Kalyaanamoorthy et al. 2017)](https://www.zotero.org/google-docs/?hbFzFg) and 1,000 bootstrap replicates. Bayesian phylogeny inference was conducted using MrBayes v3.2.6 with the GTR substitution model and 2,000,000 generations of Markov chain Monte Carlo (MCMC). The first 15% of generations were discarded as burn-in before generation of the posterior probability majority consensus tree. An alternative phylogenetic reconstruction was performed using ASTRAL-III v5.7.1 [(Zhang et al. 2018)](https://www.zotero.org/google-docs/?dVg0kU), based on a set of separately reconstructed gene trees using IQ-TREE v2.2.0. To reduce noise, we applied a filtering step by collapsing nodes with bootstrap support below 70% prior to species tree inference. Visualization of phylogenetic trees was performed using the ETE Toolkit v3.1.2 [(Huerta-Cepas et al. 2016)](https://www.zotero.org/google-docs/?sYQStl). For the final analysis and time calibration we used a reduced tree of nine species (see SM section 2.2).

## 3.2. Localization and genotyping of previously known STR loci.

Using an *in silico* PCR approach, we identified the locations of STR loci previously described for various Mustelidae species [(Davis and Strobeck 1998b; Fleming et al. 1999; Domingo-Roura 2002; Vincent et al. 2003b; Basto et al. 2010b; Natali et al. 2010)](https://www.zotero.org/google-docs/?5IBeQE). Based on the results, the markers were divided into three categories: Localized (L), Declined (D) and Not Amplified (NA). Using the sable reference assembly, we localized 44 STRs (Supplementary File 11). An additional 16 markers had low-confidence mapping and were declined. The identification rate using the pine marten reference assembly was nearly the same: 48 high confidence mappings and 15 ambiguous. None of the remaining loci were amplified. All three categories have significant overlaps between the two reference assemblies: 87.7 % (43/49) for localized STRs, 72.2 % (13/18) for declined STRs, and 75 % (15/20) for not amplified STRs, respectively. The localized markers cover all chromosomes of both species, except chr16 (Figure 12). The mean density of markers is 2.3 and 2.5 loci per chromosome for sable and pine marten, respectively.


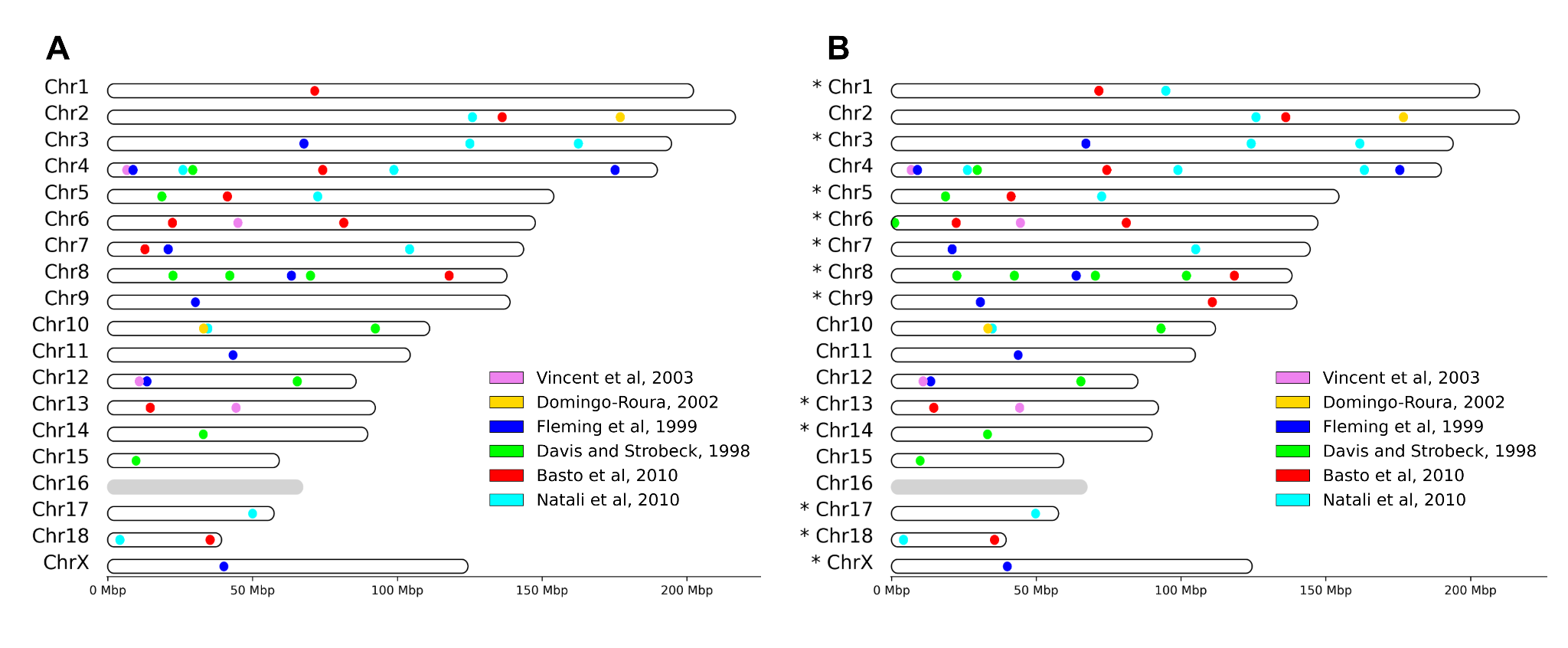


**Figure 12.** Localization of previously identified STR loci from various species of Mustelidae.
A – Distribution of STR loci in the sable reference assembly, B – Distribution of STR loci in the pine marten reference assembly. Pine marten chromosomes labeled with an asterisk were inverted to match the orientation of the sable homologous chromosomes

Further investigation of repeat motifs in the two reference assemblies (described in methods Section ”Whole genome alignments and connection between assembly and karyotype”) reduced the number of the suitable STR loci for genotyping to 36 and 44 in the sable and pine marten assemblies, respectively (Supplementary File 2). The reasons for discarding these loci were different. For example, Mf8.8, Mf4.10, Mf6.5 were too long for genotyping from 150 bp reads. The STR itself in each of these markers was longer than 100 bp in the reference sable genome. Moreover, the repeat in Mf8.8 was not completely assembled and contained a gap. Mer095 contained not one but two complementary STRs separated by a 26 bp insertion and also exceeded the 100 bp threshold. Mvis099 did not contain STR at all, and the sequences of repeats within Mel08 and Mvi_1273 were very different from the way they were originally described. Finally, Mvis020 was removed because it was X-linked.

Rozhnov et al. in their study of hybridization between the sable and the pine marten used only 9 STR markers: Mel10, Ma-1, Ma-3, Ma-8, Ma-15, Ma-18, Ma-19, Mvis072 and Mer041 [(Rozhnov et al. 2013)](https://www.zotero.org/google-docs/?bIzcAG). Seven of them passed our filters and were genotyped in our resequencing data (except Ma-3 and Mer041). Kashtanov et al. investigated population structure of the sable in Central Siberia using 8 STR markers from the Rozhnov et al. set (Mel10 was excluded) and 8 additional STR loci (Mf3.7, Mar08, Mar21, Mar36, Mar43, Mar53, Mar58 and Mar64) [(S. Kashtanov et al. 2022)](https://www.zotero.org/google-docs/?i9jLnn). Among the additional STR markers, only one (Mar53) was not genotyped using the sable genome assembly in our analysis. To compare our WGS-based ADMIXTURE results with these studies, we performed STRUCTURE analysis using three sets of STR markers: (1) full set, including all loci, (2) Rozhnov’s set and (3) Kashtanov’s set, excluding non genotyped loci.

## 3.3. Components of heterozygosity distributions

To analyze the components of heterozygosity distributions, we fitted our data with a linear combination of negative binomial distributions. For each sample we tried models with different numbers of components or different starting values of the parameters. For each sample we selected one of six models (Table 2). Comparing the distributions of our samples, we initially empirically identified 4 components that corresponded to modes of the groups of studied individuals: ROH component (0 SNPs/kbp), pine marten component (P) (0.5 SNPs/kbp), sable component (S) (1.5 SNPs/kbp) and hybrid component (H) (4.5 SNPs/kbp). Thus, starting values for mean (μ) and standard deviation (σ) of each component (Table 3) were empirically determined based on heterozygosity distributions of the studied samples.

As input data we used counts of the heterozygous SNPs in sliding windows of 1 Mb with a 100 kbp step size. Counts from X chromosomes were removed prior to the analysis for all samples. Distributions of counts were constructed with a bin width of 100 counts (0.1 SNPs/kbp). The first two bins (corresponding to ROH) were removed to improve model fitting. Model fitting for heterozygosity distributions was performed using the mix() function with ‘dist = "nbinom"’ from the R package mixdist v.0.5-5 [(Macdonald 2018)](https://www.zotero.org/google-docs/?2M0Imf).

**Table 2.** Fitted models and their components.

| **Model** | **Formula *** | **Components** | | |
| --- | --- | --- | --- | --- |
|  |  | **Pine marten** | **Sable** | **Hybrid** |
| General model | a_p_ * Nb_p_ + a_s_ * Nb_s_ + a_h_ * Nb_h_ | + | + | + |
| Model without hybrid heterozygosity | a_p_ * Nb_p_ + a_s_ * Nb_s_ | + | + | – |
| Pure pine model | a_p_ * Nb_p_ | + | – | – |
| Pure sable model | a_s_ * Nb_s_ | – | + | – |
| Pine + hybrid model | a_r_ * Nb_r_ + a_p_ * Nb_p_ + a_h_ * Nb_h_ | + | – | + |
| Sable + hybrid model | a_r_ * Nb_r_ + a_s_ * Nb_s_ + a_h_ * Nb_h_ | – | + | + |

* Formula coefficients:

- a – weight of an individual component (coefficient in linear combination).
- Nb – negative binomial distribution with mean value (μ) and standard deviation (σ).
- _p_ _s_ _h_ – subscripts of individual components (pine marten, sable and hybrid).

**Table 3.** Starting values for mean (μ) and standard deviation (σ) of each component.

| **Component** | **μ** | **σ** |
| --- | --- | --- |
| Pine marten (P) | 700 | 200 |
| Sable (S) | 1700 | 500 |
| Hybrid (H) | 4500 | 700 |

We selected the best model for each sample in two steps. First, we filtered models based on the final mean (μ) of the fitted model; if any component's μ fell outside the range of the starting μ ± standard deviation (σ), we discarded the model. Next, we selected the best model based on the smallest quadratic deviation, calculated as the sum of squared differences between the original and fitted distributions, excluding the first bin. After selecting the best model, we identified means and modes for each fitted distribution (Supplementary File 5). We clustered all mean values of individual fitted distributions from the best-fit models using hierarchical agglomerative clustering. We used the *linkage()* function from Scipy v1.13.1 with the "average" method. We visualized the results of the clustering as a dendrogram. We formed flat clusters using the *fcluster* function from Scipy, setting the optimal number of clusters to 3 (sable, pine marten, and hybrid clusters). We clustered the mean values of individual component distributions for each of the three groups using the maxclust criterion and calculated medians for each cluster. We performed all stages of the analysis based on both genome assemblies of sable and pine marten. Specifically, we calculated three median values for each of the three clusters relative to the sable reference, and similarly for the pine marten reference. Finally, we obtained and visualized the mean values of these medians for each cluster on the stripped histograms (MT Figure 7A).

## 3.4. Algorithm for identification of a pseudoautosomal region.

To set the correct ploidy of X chromosome segments during variant calling we identified coordinates of a pseudoautosomal region (PAR). First, median coverage was estimated in non-overlapping sliding windows of 10 kbp. Chains of at least 10 consecutive windows with median coverage (of each window) ≥70% of the whole genome value were merged. Next, a median coverage was calculated for windows between adjacent chains. If it exceeded 70% of the whole genome level adjacent chains were merged too to generate a set of candidate regions. Finally, we compared the candidates with per-base coverage and selected the correct one. The scheme of the procedure is provided on Figure 13.


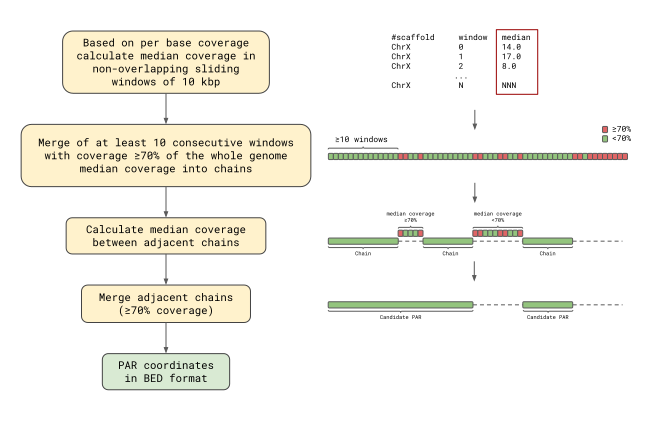


**Figure 13**. Scheme of algorithm for PAR identification.

## 3.5. snakeSTR pipeline.

The snakeSTR pipeline (https://github.com/mahajrod/snakeSTR), built on Snakemake, enables STR-typing and STR-based admixture analysis (Figure 14). Pipeline description:

**I - V. Preprocessing**. On the first stage for each sample and STR loci we extracted reads aligned to it, including 1000 bp flanks using Samtools v1.19.2 [(Li et al. 2009)](https://www.zotero.org/google-docs/?R6oDjm) (I, II), verified pairing using Bazam v1.0.1 [(Sadedin and Oshlack 2019)](https://www.zotero.org/google-docs/?mRS7eB) (III), mapped them back to the reference genome using BWA v0.7.17 [(Li and Durbin 2009)](https://www.zotero.org/google-docs/?iD0MwZ) (IV) and performed an indel-aware realignment using IndelRealigner from GATK v3.7 [(McKenna et al. 2010)](https://www.zotero.org/google-docs/?7yLPup) (V).

**VI - VII. STR-typing**. Next, we performed STR-typing using hipSTR v0.6.2 [(Willems et al. 2017)](https://www.zotero.org/google-docs/?WBgUnZ) (VI-VII). The following workflow includes both analysis of raw and filtered STR-loci sets. Analysis of raw STR-loci sets was performed only with a minimum read depth threshold of 5 (--min-reads). Analysis of filtered STR-loci sets was performed with additional filtering (VI a) according to the following criteria: also with a minimum read depth threshold of 5, a minimum call quality threshold of 0.9 (--min_call_qual), a maximum allowable fraction of reads with flank indels set to 0.15 (--max_call_flank_indel), a maximum allowable fraction of reads with stutter artifacts set to 0.15 (--max_call_stutter), a minimum call allele bias threshold of -2 (--min_call_allele_bias), and a minimum call strand bias threshold of -2 (--min_call_strand_bias). From this point, all downstream stages were performed for both raw and filtered STR-loci sets to assess the effect of the filtration on the final results.

**VIII - XIV. Admixture analysis**. STR-based admixture analysis performed using STRUCTURE v2.3.4 and Clumpp v1.1.2 [(Pritchard et al. 2000; Jakobsson and Rosenberg 2007)](https://www.zotero.org/google-docs/?gthENE) (VIII-XIV). Admixture analysis was performed for values of parameter K (number of populations) from 2 to 6 in triplicate for each. The presented DAG includes only K = 2 for clarity. Regarding Clumpp, the following parameters were employed: M = 1 (indicating the FullSearch method), W = 0 (denoting no weighting by the number of individuals in each population), and S = 2 (representing the use of the pairwise matrix similarity statistic G'). Additionally, the Greedy algorithm was employed with the option set to 2, indicating random input orders, and 100 repeats were performed to ensure robustness of the results.

**XV - XVI. Visualization**. Creation input files needed to visualize the clustering results using Pong v1.5 [(Behr et al. 2016)](https://www.zotero.org/google-docs/?e4umfy) (XV). Generation a Bash script to automatically run an interactive visualization using Pong with default parameters (XVI).


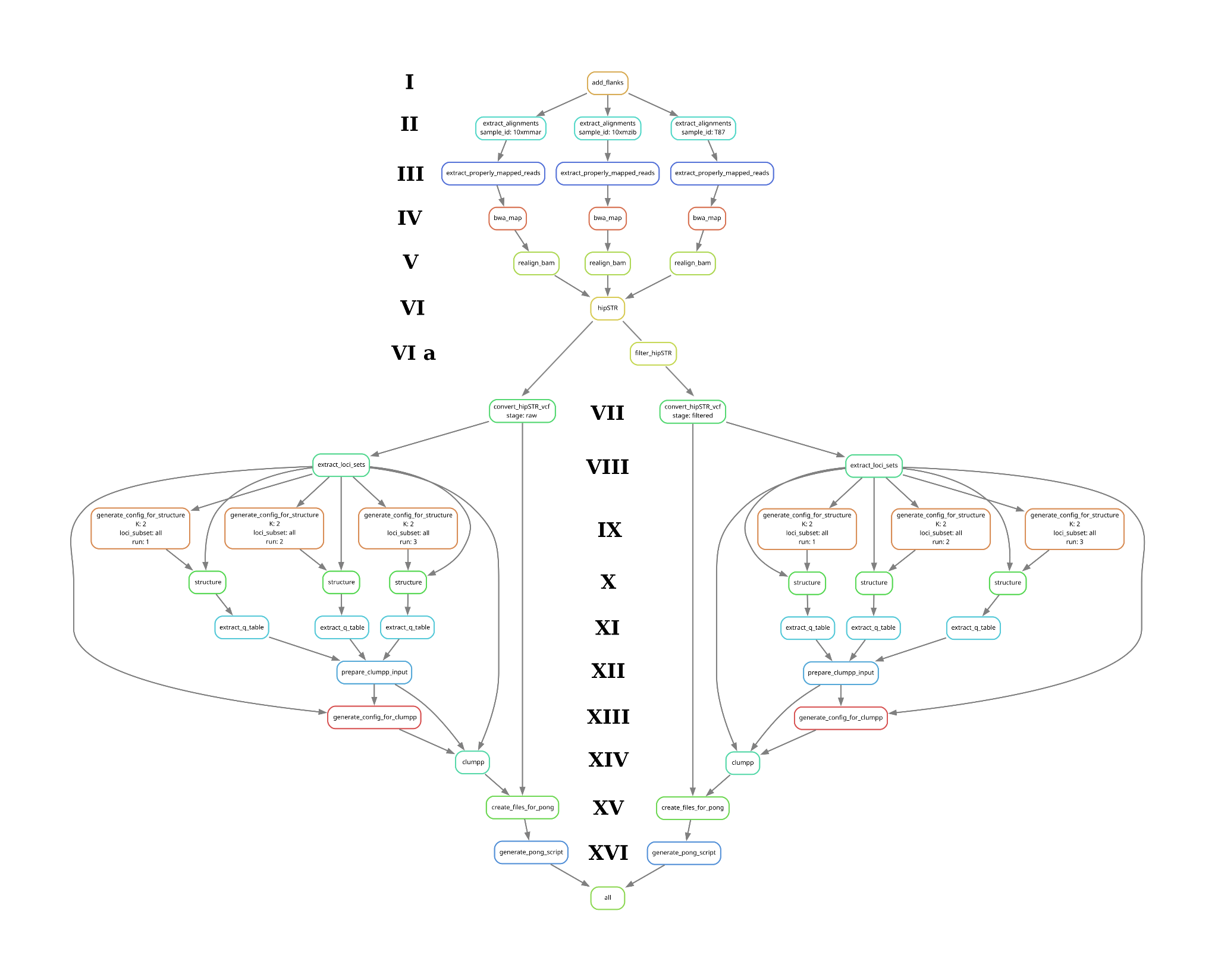


**Figure 14.** Rule graph of the snakeSTR pipeline.
Roman numerals indicate the steps of workflow.

## 3.6. Fst and Tajima’s D

Fst and Tajima’s D statistics were calculated using Vcftools v.0.1.16 and VCF-kit v.0.2.9 [(Danecek et al. 2011; Cook and Andersen 2017)](https://www.zotero.org/google-docs/?eSxtkE), respectively, in 1 Mbp sliding windows with a 100 kbp step, using the sable genome assembly as a reference. The comparison was performed between groups of pure sables and pure pine martens (based on local Admixture classification). Genomic windows with Fst and Tajima’s D estimates were filtered in two stages. First, windows containing more than 50,000 Ns were discarded. Next, filtering was performed based on repeat content, which was identified using Tandem Repeats Finder v4.09.1 [(Benson 1999)](https://www.zotero.org/google-docs/?DuAvpc), WindowMasker 1.0.0 [(Morgulis et al. 2006)](https://www.zotero.org/google-docs/?q70D69), and RepeatMasker v.4.1.2.p1 [(Smit et al. 2013)](https://www.zotero.org/google-docs/?QkDVx5). Conversion of GFF with repeats to BED format was performed using gff2bed from BEDOPS v.2.4.40 [(Neph et al. 2012)](https://www.zotero.org/google-docs/?gJ32WY). Obtaining masked bases by windows was done by merging and then intersecting using Bcftools v1.15.1 [(Danecek et al. 2021)](https://www.zotero.org/google-docs/?rPBA2L). For each window, we calculated the number of masked bases. The distributions of repeats were visualized as histograms for the whole assembly (Figure 15), as well as in the form of a heatmap (Figure 16). Based on the visualization of these distributions, we applied a cutoff at the 95th percentile, meaning that windows containing more than 607,592 bp of repeats were removed. Finally, the coordinates of regions consisting of overlapping windows are obtained using Bedtools v.2.31.1 [(Quinlan and Hall 2010)](https://www.zotero.org/google-docs/?HKmbzS).


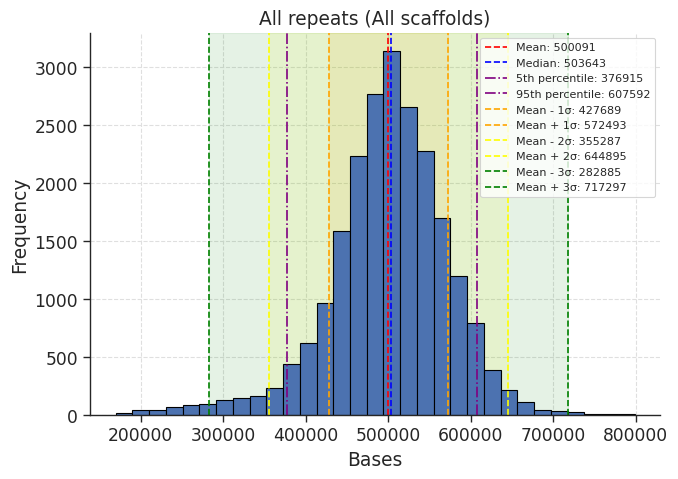


**Figure 15.** Distribution of repeats across 1 Mbp sliding windows in all scaffolds of the sable genome assembly.


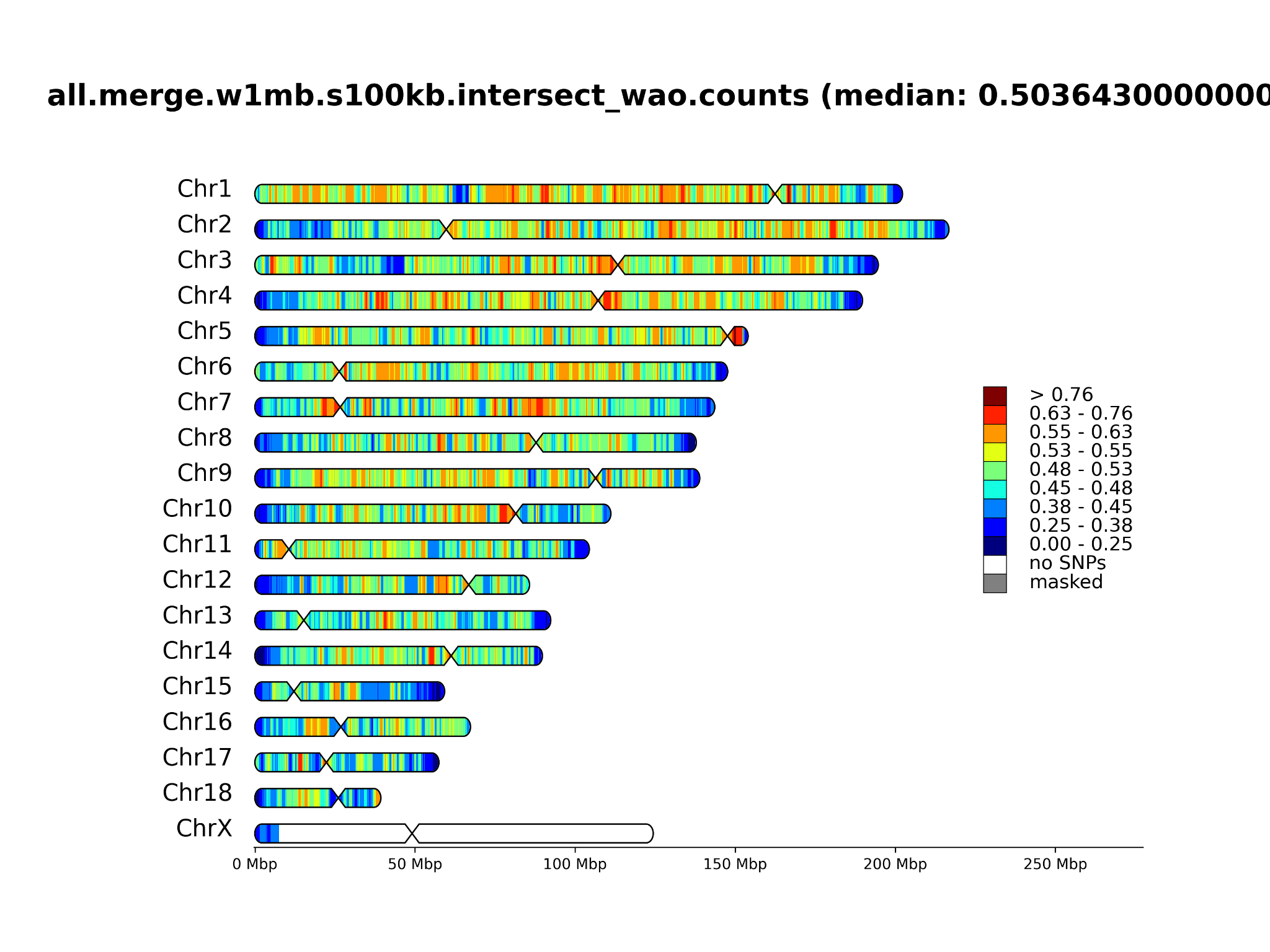


**Figure 16.** Density of the all repeats in the sliding windows of 1 Mbp with a step of 100 kbp.

## 3.7. Morphological analysis

Skulls and skins of ten martens from the sympatric zone (specimens T76-T79, T81-T85, T87) were investigated for differences in morphology. The skulls were compared with specimens of *M. martes* (n=32) and *M. zibellina* (n=44) from allopatric populations that represent putatively pure ancestries for each species, kept in the collections of the Zoological Institute, Russian Academy of Sciences (Saint Petersburg, Russia). Twenty four measurements were taken on each skull using a sliding caliper with an accuracy of 0.1 mm. See Abramov & Tumanov (2003) and Monakhov (2020) for the scheme of measurements [(Abramov and Tumanov 2003; Monakhov 2021a)](https://www.zotero.org/google-docs/?pvbZxT). Multivariate analyses (cluster analysis and discriminant analysis) were carried out to evaluate differences among the samples. Statistical analyses were performed in Statistica 6.0 [(StatSoft 2001)](https://www.zotero.org/google-docs/?MLuvkQ).

# 4. Additional Materials

## 4.1. Additional Figures

**Additional Figure AF1.** Phylogenetic trees based on data matrix of 5989 common single-copy BUSCOs.
A – IQtree (ML), B – MrBayes (BI).


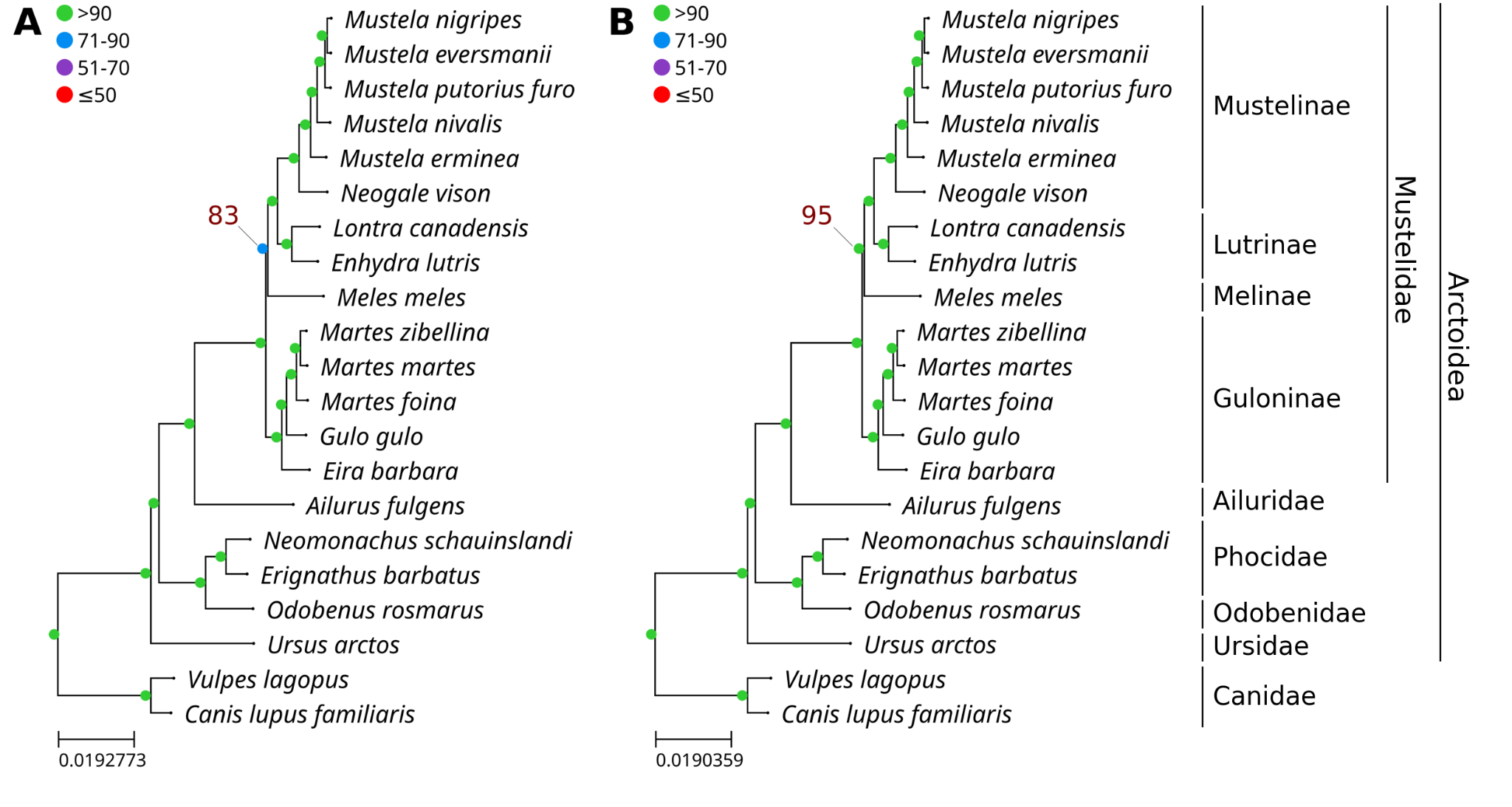


**Additional Figure AF2.** ASTRAL-III phylogenetic tree was reconstructed based on 5989 single-copy BUSCO gene alignments.

Individual gene trees were inferred using IQTree (1000 bootstraps). Nodes with bootstrap support values below 70 were excluded from the input trees. Numbers displayed at each node represent unique node identifiers, for which detailed statistical values are provided in Additional Table AT1. Colored dots indicate the main local posterior probabilities (pp1), expressed as percentages.


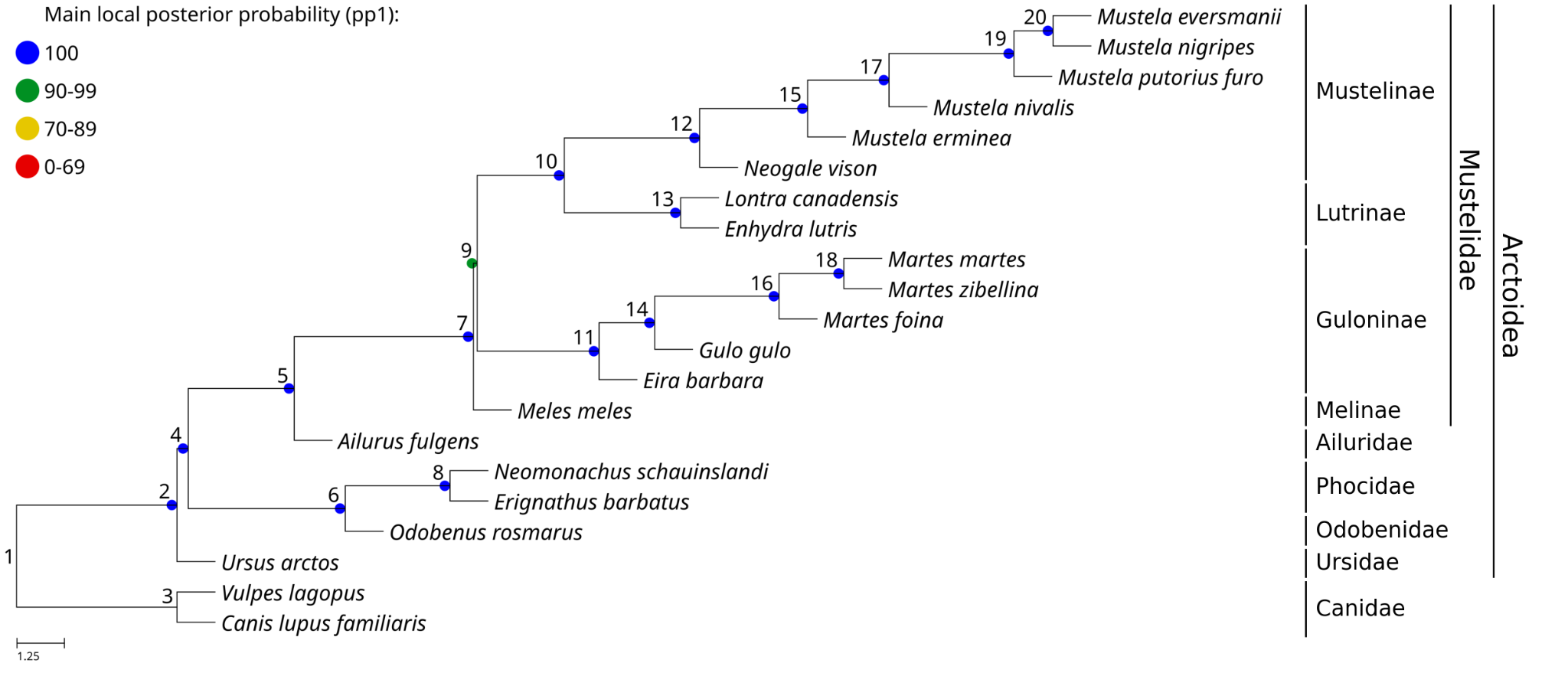


**Additional Figure AF3**. ASTRAL-III phylogenetic tree was reconstructed based on 5989 single-copy BUSCO gene alignments.
Individual gene trees were inferred using IQTree (1000 bootstraps). Nodes with bootstrap support values below 70 were excluded from the input trees. Detailed statistical values are provided in Additional Table AT1. Piecharts indicate the quartet supports.


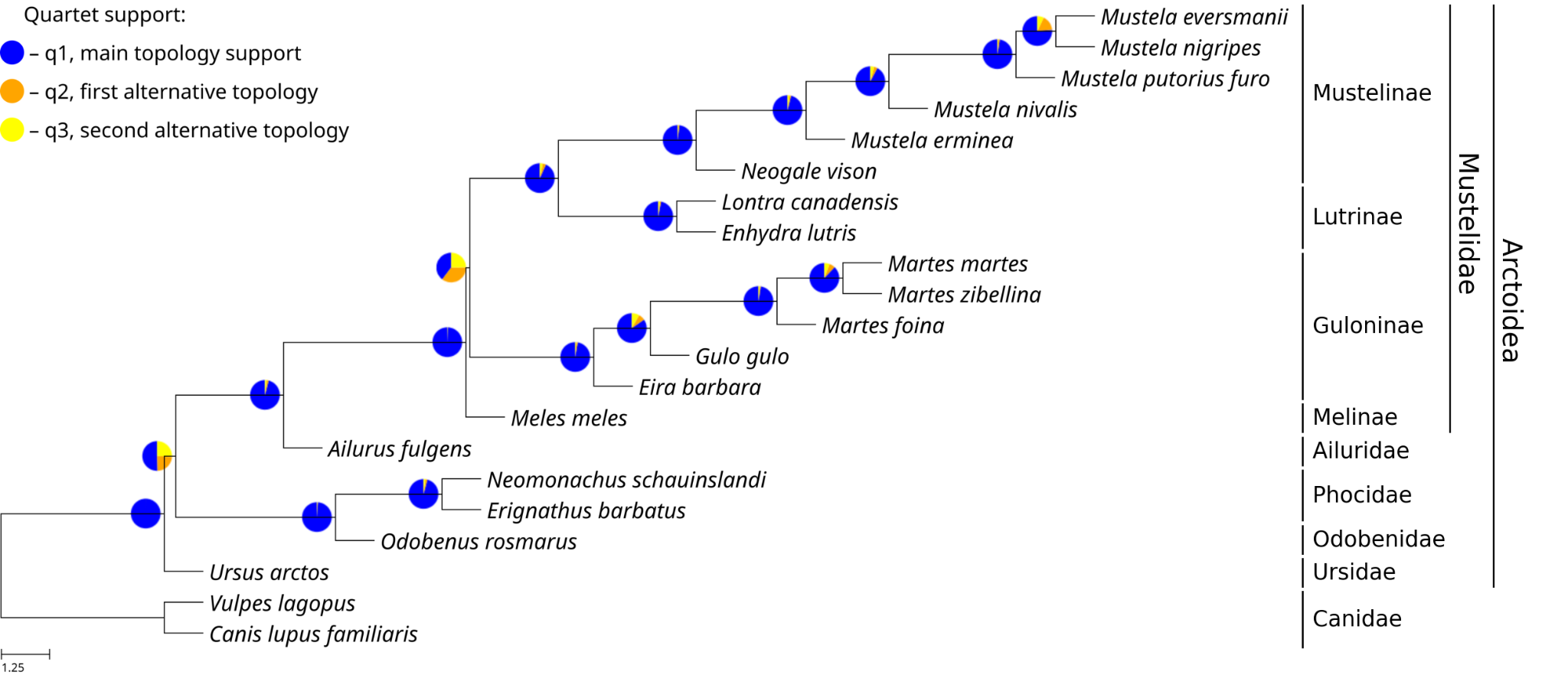


**Additional Figure AF4.** Difference in number of masked bases (all, high coverage and low coverage) between the sable and pine marten references for the pure sables, pure pine martens and hybrids.
High coverage – more than 250% of the median coverage, low coverage – less than 33% of the median coverage. Values are provided in Additional Table AT4.


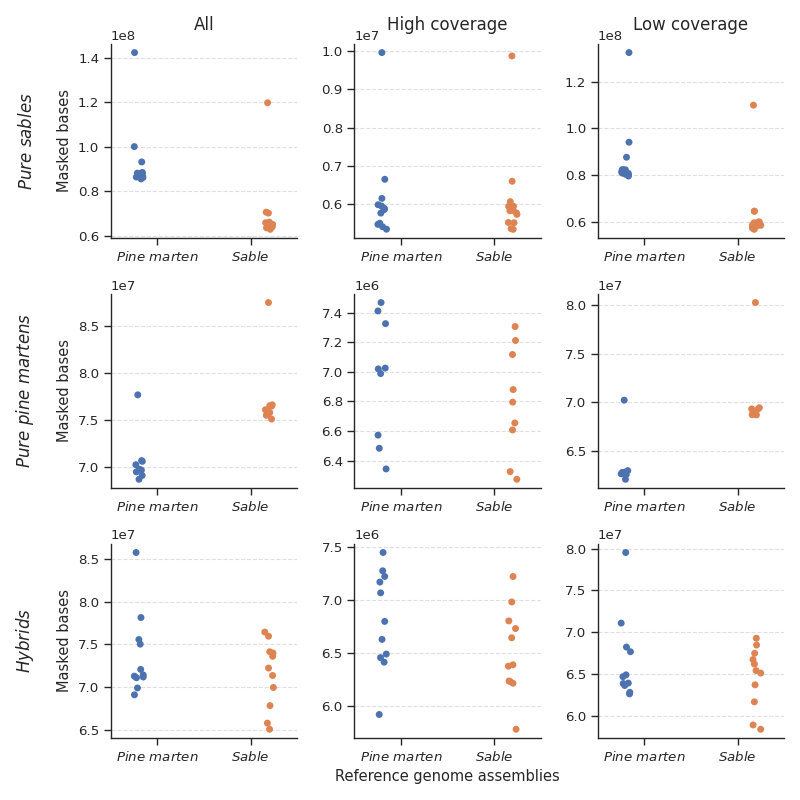


**Additional Figure AF5**. Median coverage in non-overlapping 10 kbp windows for males across the pseudoautosomal region in sable (A) and pine marten (B) genome assembly.


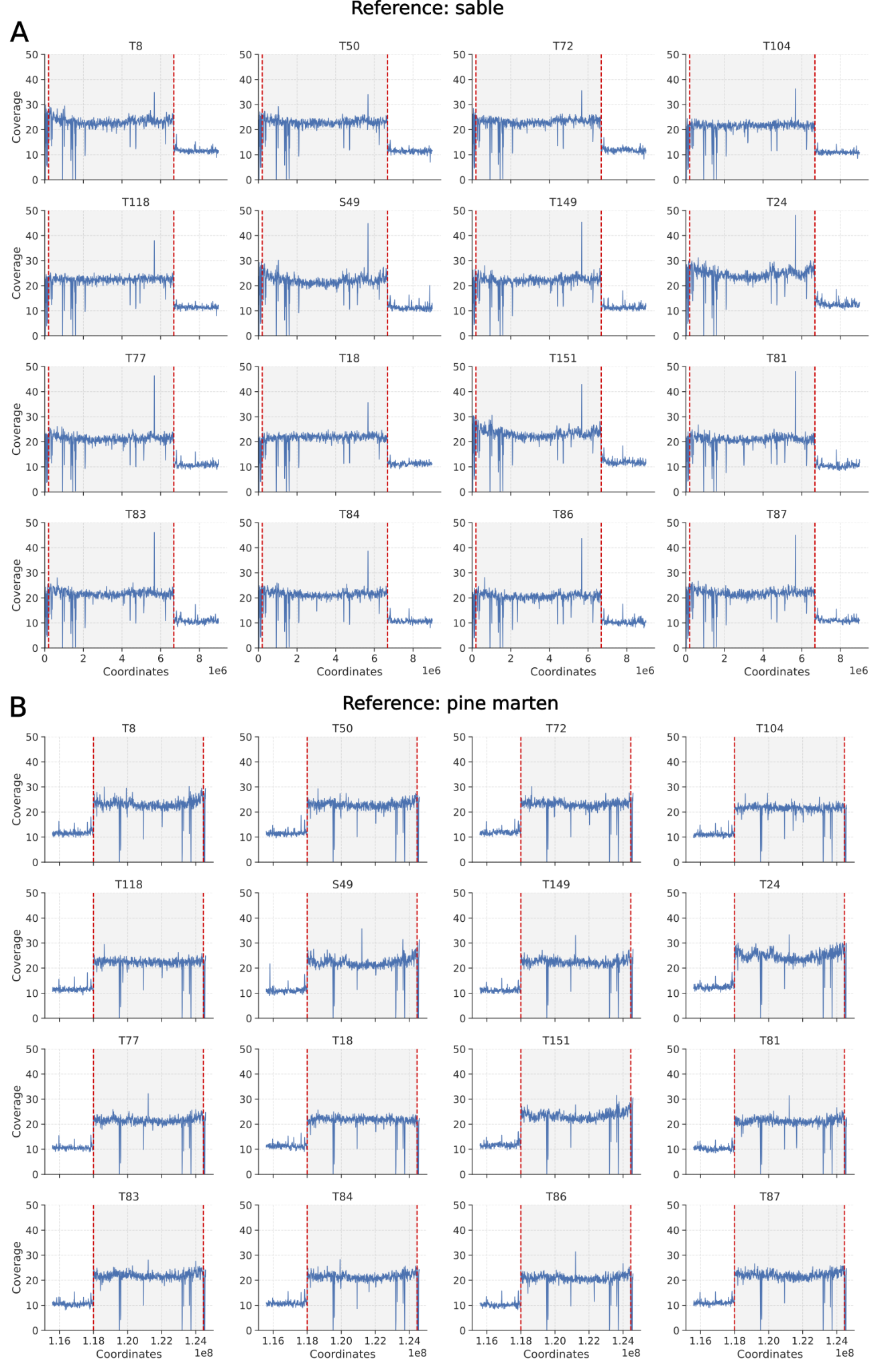


**Additional Figure AF6**. Distributions of mean heterozygosity (SNP only) counted in 1 Mbp sliding windows with 100 kbp step for both sable (red) and pine marten (blue) genomes.


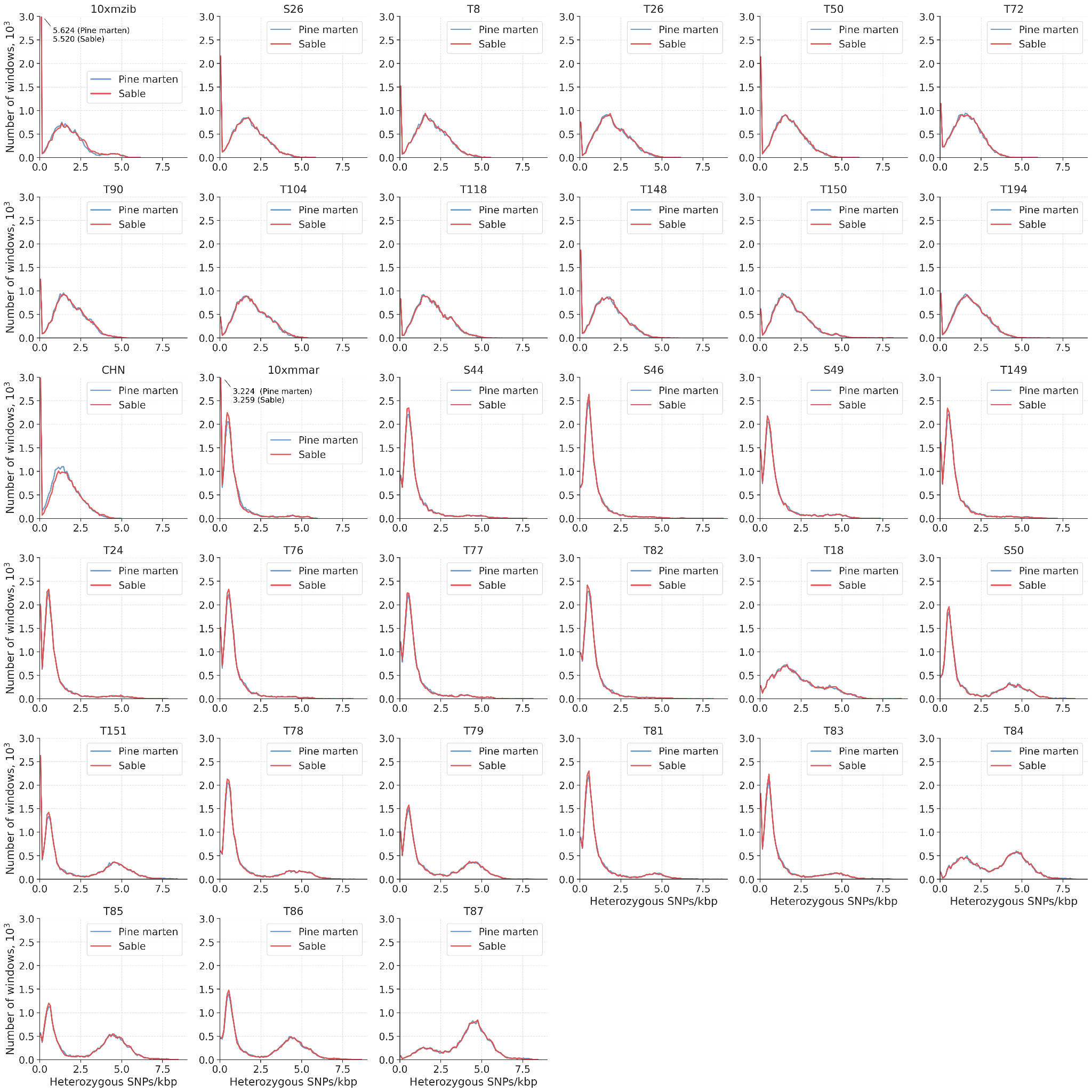


**Additional Figure AF7.** Fitting models for heterozygosity distributions (sable reference).

Red triangles indicate mean values for the distribution of individual model components.


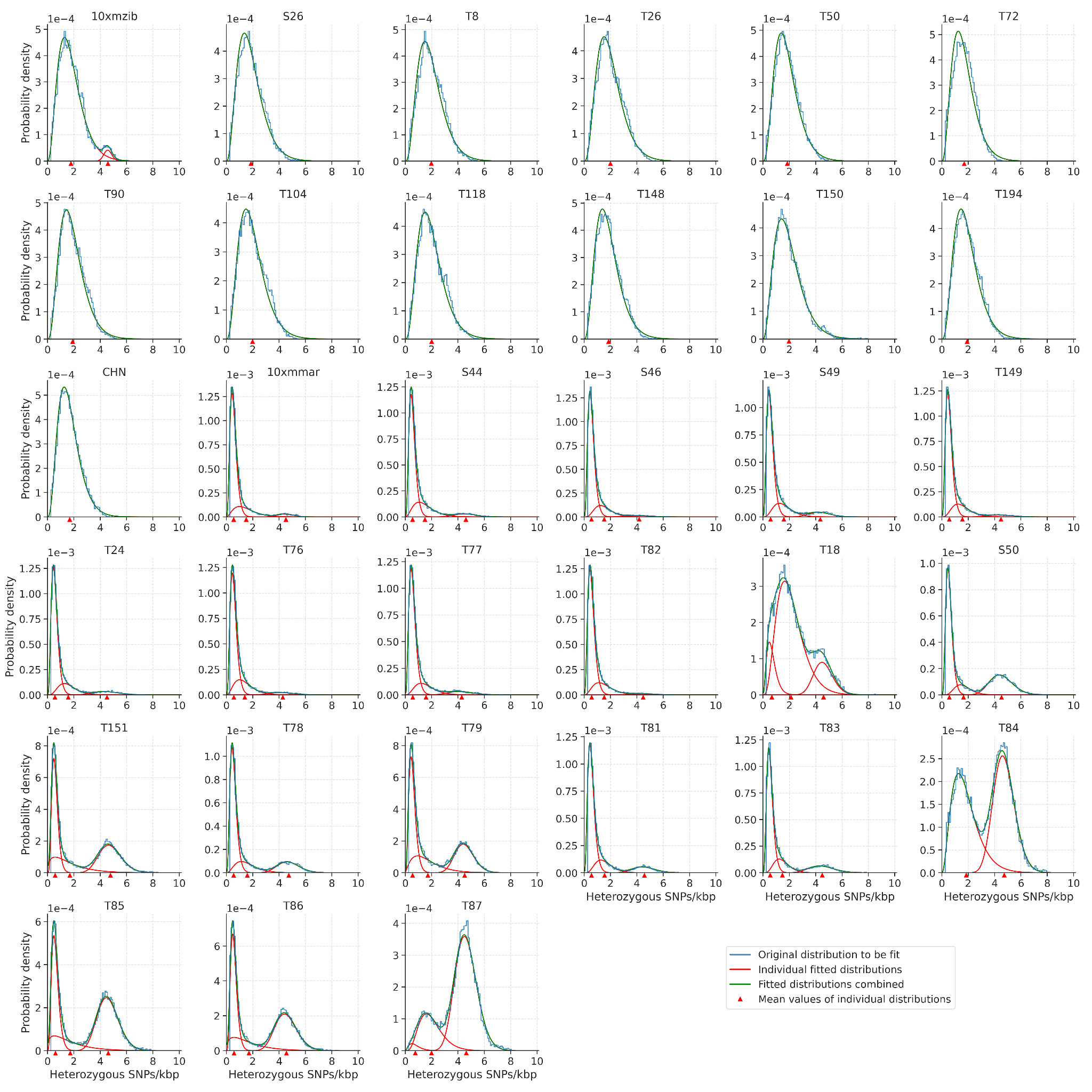


**Additional Figure AF8.** Fitting models for heterozygosity distributions (pine marten reference).

Red triangles indicate mean values for the distribution of individual model components.


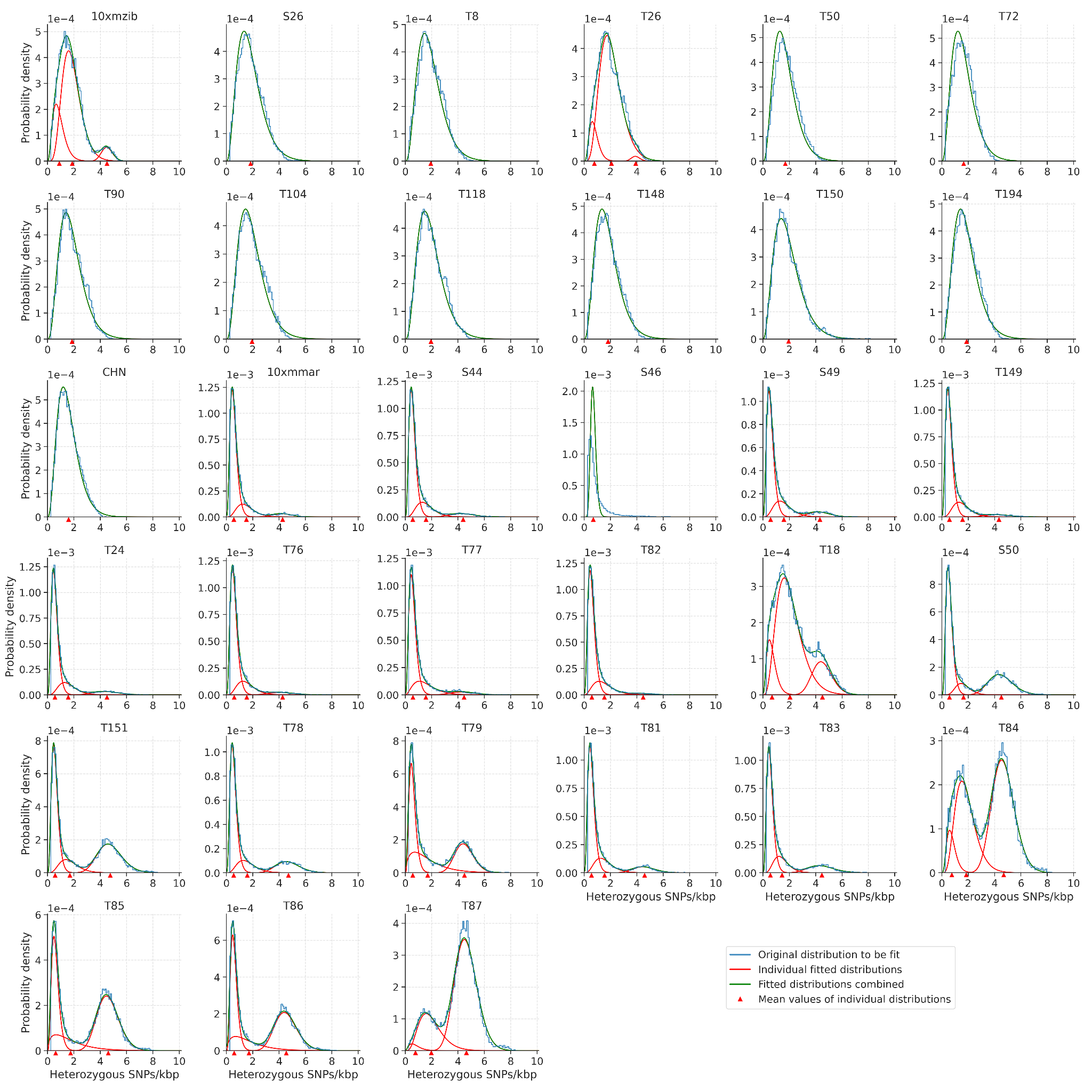


**Additional Figure AF9.** Cumulative lengths (fraction of the genome) of the RoH of different categories based on sable (A) and pine marten (B) reference.
RoH categories: Short RoH, S (< 1 Mbp), Long RoH, L (>= 1 Mbp and < 10 Mbp) and Ultra Long RoH, UL (>= 10 Mbp). Values in Additional Table AT8.


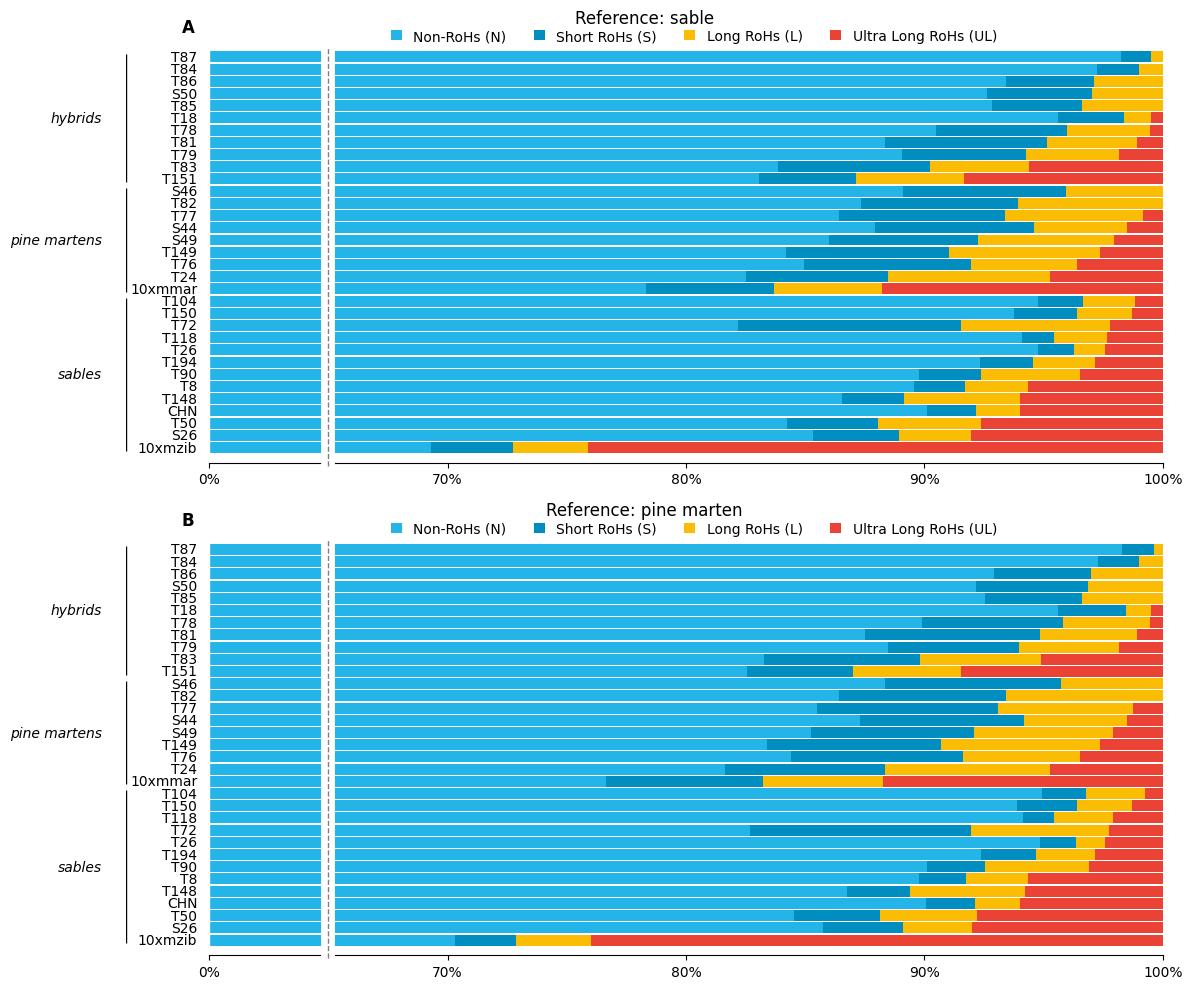


**Additional Figure AF10.** Localization of markers and ancestry analysis for three sets of STR loci (All, Rozhnov’s, Kashatonov’s) based on sable and pine marten genome.


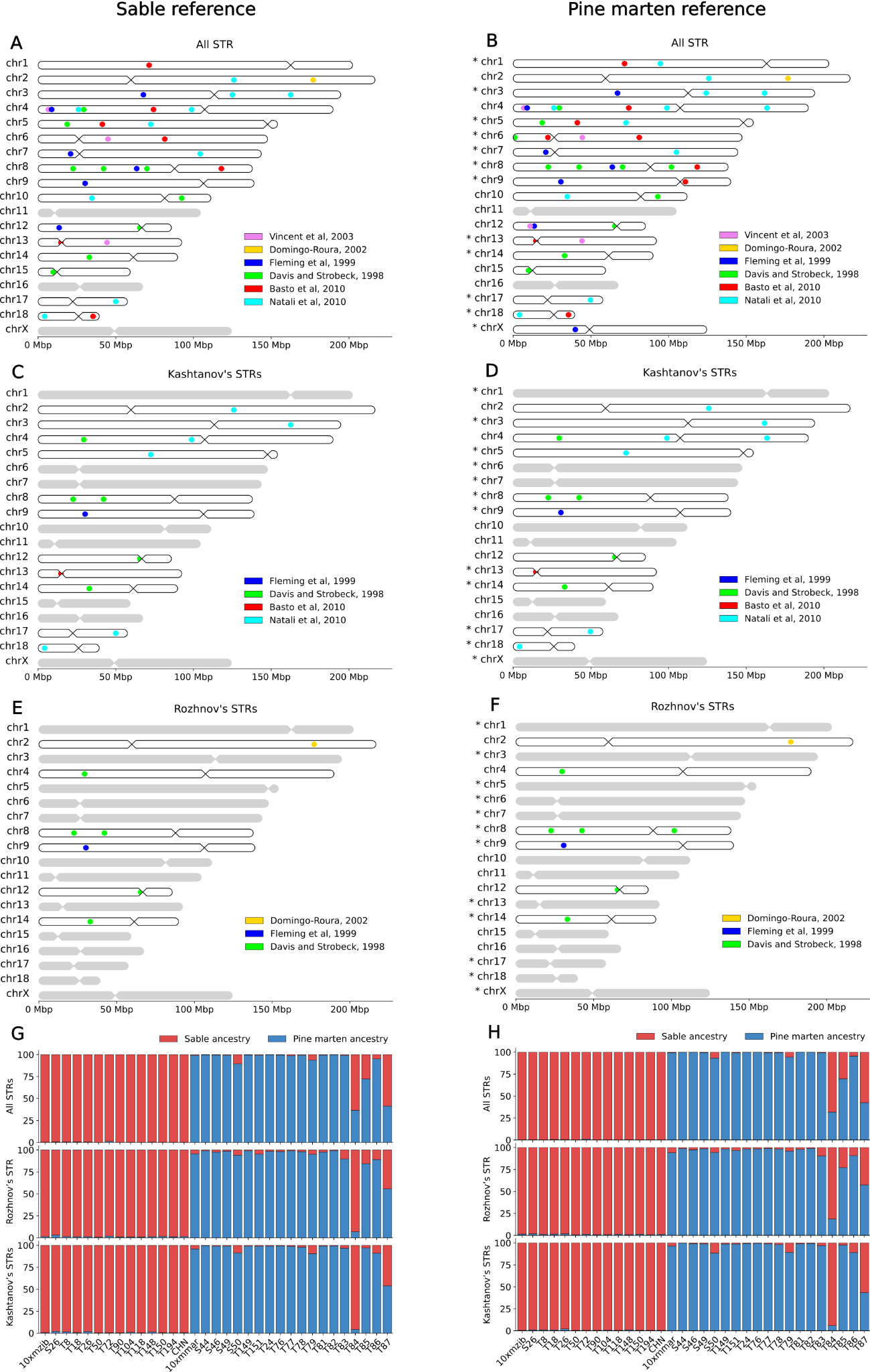


## 4.2. Additional Tables

**Additional Table AT1**. Summary of support metrics for each internal branch in the ASTRAL species tree.

The table reports quartet support values (q1, q2, q3), representing the proportion of gene tree quartets supporting the main topology and two alternatives; the corresponding counts of supporting quartets across all gene trees (f1, f2, f3); local posterior probabilities for each topology (pp1, pp2, pp3); the total number of informative quartets (QC); and the effective number of genes contributing to each branch (EN).

| **Node** | **q1** | **q2** | **q3** | **f1** | **f2** | **f3** | **pp1** | **pp2** | **pp3** | **QC** | **EN** |
| --- | --- | --- | --- | --- | --- | --- | --- | --- | --- | --- | --- |
| 1 | - | - | - | - | - | - | - | - | - | - | - |
| 2 | 0.999786 | 0.000093 | 0.000121 | 5,977.94 | 0.556 | 0.722 | 1 | 0 | 0 | 18 | 5,979.22 |
| 3 | - | - | - | - | - | - | - | - | - | - | - |
| 4 | 0.504337 | 0.238758 | 0.256905 | 1,800.62 | 852.433 | 917.222 | 1 | 0 | 0 | 90 | 3,570.28 |
| 5 | 0.959436 | 0.021889 | 0.018674 | 4,961.98 | 113.206 | 96.579 | 1 | 0 | 0 | 126 | 5,171.76 |
| 6 | 0.989571 | 0.005326 | 0.005103 | 5,617.54 | 30.233 | 28.967 | 1 | 0 | 0 | 90 | 5,676.74 |
| 7 | 0.994269 | 0.002648 | 0.003083 | 5,763.06 | 15.346 | 17.872 | 1 | 0 | 0 | 78 | 5,796.28 |
| 8 | 0.958165 | 0.021062 | 0.020773 | 5,327.61 | 117.111 | 115.5 | 1 | 0 | 0 | 18 | 5,560.22 |
| 9 | 0.397202 | 0.355236 | 0.247562 | 1,018.39 | 910.796 | 634.729 | 0.999999998 | 0.000000002 | 0 | 280 | 2,563.92 |
| 10 | 0.933255 | 0.036117 | 0.030628 | 3,699.09 | 143.154 | 121.4 | 1 | 0 | 0 | 480 | 3,963.64 |
| 11 | 0.973431 | 0.014125 | 0.012444 | 4,774.00 | 69.273 | 61.031 | 1 | 0 | 0 | 256 | 4,904.31 |
| 12 | 0.981458 | 0.009831 | 0.008711 | 5,221.82 | 52.308 | 46.346 | 1 | 0 | 0 | 130 | 5,320.48 |
| 13 | 0.969222 | 0.013683 | 0.017095 | 4,774.03 | 67.397 | 84.205 | 1 | 0 | 0 | 78 | 4,925.63 |
| 14 | 0.846536 | 0.071584 | 0.08188 | 3,030.33 | 256.25 | 293.104 | 1 | 0 | 0 | 48 | 3,579.69 |
| 15 | 0.961569 | 0.01665 | 0.021782 | 4,730.97 | 81.917 | 107.167 | 1 | 0 | 0 | 60 | 4,920.05 |
| 16 | 0.97518 | 0.010772 | 0.014048 | 4,649.03 | 51.353 | 66.971 | 1 | 0 | 0 | 34 | 4,767.35 |
| 17 | 0.922293 | 0.036949 | 0.040758 | 3,980.77 | 159.479 | 175.917 | 1 | 0 | 0 | 48 | 4,316.17 |
| 18 | 0.879455 | 0.064054 | 0.056491 | 3,165.50 | 230.556 | 203.333 | 1 | 0 | 0 | 18 | 3,599.39 |
| 19 | 0.975488 | 0.01443 | 0.010082 | 4,598.82 | 68.029 | 47.529 | 1 | 0 | 0 | 34 | 4,714.38 |
| 20 | 0.762988 | 0.16628 | 0.070733 | 2,227.50 | 485.444 | 206.5 | 1 | 0 | 0 | 18 | 2,919.44 |

**Additional Table AT2**. Dates of species divergence (in millions of years) for correlated, independent and global molecular clocks.

| **Node ID** | **Description** | **Correlated** | | | **Independent** | | | **Global** | | |
| --- | --- | --- | --- | --- | --- | --- | --- | --- | --- | --- |
|  |  | **Date** | **HPD Lower** | **HPD Upper** | **Date** | **HPD Lower** | **HPD Upper** | **Date** | **HPD Lower** | **HPD Upper** |
| 1 | MRCA of *M. martes* and *M. zibellina* | 2.02 | 1.53 | 2.58 | 1.52 | 1.05 | 2.06 | 1.35 | 1.31 | 1.39 |
| 2 | MRCA of *M. foina*, *M. zibellina* and *M. martes* | 3.66 | 2.81 | 4.65 | 2.83 | 2.11 | 3.66 | 2.54 | 2.47 | 2.6 |
| 3 | MRCA of *G. gulo* and *Martes* genus | 7.94 | 6.27 | 9.94 | 6.57 | 5.16 | 8.27 | 5.58 | 5.45 | 5.7 |
| 4 | MRCA of Guloninae | 9.92 | 7.89 | 12.35 | 8.32 | 6.7 | 10.33 | 7.25 | 7.08 | 7.4 |
| 5 | MRCA of *E. lutris* and *L. canadensis* | 7.79 | 6.04 | 9.86 | 7.45 | 5.64 | 9.59 | 6.79 | 6.63 | 6.93 |
| 6 | MRCA of Lutrinae and Melinae | 15.49 | 12.62 | 19.06 | 14.64 | 12.12 | 17.99 | 13.34 | 13.04 | 13.6 |
| 7 | MRCA of Guloninae, Lutrinae and Melinae | 16.17 | 13.18 | 19.87 | 15.34 | 12.68 | 18.8 | 13.53 | 13.22 | 13.78 |
| 8 | MRCA of Ailuridae and Mustelidae | 31.52 | 30.9 | 32.1 | 31.53 | 30.91 | 32.11 | 31.6 | 30.94 | 32.13 |

**Additional Table AT3.** Comparison of whole genome coverage for the pine marten and the sable references.

Note that the coverage was estimated from the alignments of downsampled reads (to 22 ± 2x coverage according to the k-mer distributions).

| **Sample** | **Sable reference** | | **Pine marten reference** | | **Difference** | |
| --- | --- | --- | --- | --- | --- | --- |
|  | **Mean** | **Median** | **Mean** | **Median** | **Mean** | **Median** |
| 10xmzib | 23.34 | 23 | 22.99 | 23 | 0.35 | 0 |
| S26 | 23.71 | 23 | 23.46 | 23 | 0.25 | 0 |
| T8 | 22.44 | 22 | 22.21 | 22 | 0.23 | 0 |
| T26 | 22.57 | 22 | 22.34 | 22 | 0.23 | 0 |
| T50 | 22.32 | 22 | 22.08 | 22 | 0.24 | 0 |
| T72 | 22.68 | 23 | 22.46 | 23 | 0.22 | 0 |
| T90 | 21.57 | 21 | 21.36 | 21 | 0.21 | 0 |
| T104 | 21.34 | 21 | 21.13 | 21 | 0.21 | 0 |
| T118 | 22.22 | 22 | 21.99 | 22 | 0.23 | 0 |
| T148 | 22.05 | 22 | 21.81 | 22 | 0.24 | 0 |
| T150 | 22.56 | 22 | 22.33 | 22 | 0.23 | 0 |
| T194 | 21.02 | 21 | 20.8 | 21 | 0.22 | 0 |
| CHN | 21.07 | 20 | 20.84 | 20 | 0.23 | 0 |
| 10xmmar | 23.91 | 23 | 24 | 23 | -0.09 | 0 |
| S44 | 22.43 | 22 | 22.45 | 22 | -0.02 | 0 |
| S46 | 22.51 | 22 | 22.54 | 22 | -0.03 | 0 |
| S49 | 21.19 | 21 | 21.23 | 21 | -0.04 | 0 |
| T149 | 21.54 | 21 | 21.6 | 21 | -0.06 | 0 |
| T24 | 23.51 | 23 | 23.57 | 23 | -0.06 | 0 |
| T76 | 21.03 | 20 | 21.04 | 21 | -0.01 | -1 |
| T77 | 20.68 | 20 | 20.71 | 20 | -0.03 | 0 |
| T82 | 20.85 | 20 | 20.87 | 21 | -0.02 | -1 |
| T18 | 21.89 | 22 | 21.69 | 22 | 0.2 | 0 |
| S50 | 21.42 | 21 | 21.39 | 21 | 0.03 | 0 |
| T151 | 22.59 | 22 | 22.6 | 22 | -0.01 | 0 |
| T78 | 20.41 | 20 | 20.4 | 20 | 0.01 | 0 |
| T79 | 24.25 | 24 | 24.24 | 24 | 0.01 | 0 |
| T81 | 20.29 | 20 | 20.32 | 20 | -0.03 | 0 |
| T83 | 20.54 | 20 | 20.57 | 20 | -0.03 | 0 |
| T84 | 20.7 | 20 | 20.56 | 20 | 0.14 | 0 |
| T85 | 20.29 | 20 | 20.24 | 20 | 0.05 | 0 |
| T86 | 19.79 | 19 | 19.78 | 19 | 0.01 | 0 |
| T87 | 21.19 | 21 | 21.06 | 21 | 0.13 | 0 |

**Additional Table AT4**. Counts masked bases for the sable and the pine marten references.
High coverage – more than 250% of the median coverage, low coverage – less than 33% of the median coverage.

| **Sample** | **Sable reference, Mbp** | | | **Pine marten reference, Mbp** | | |
| --- | --- | --- | --- | --- | --- | --- |
|  | **All masked, Mbp** | **High coverage, Mbp** | **Low coverage, Mbp** | **All masked, Mbp** | **High coverage, Mbp** | **Low coverage, Mbp** |
| 10xmzib | 70.51 | 5.94 | 64.58 | 100.02 | 5.95 | 94.07 |
| S26 | 63.94 | 6.59 | 57.34 | 86.69 | 6.65 | 80.05 |
| T8 | 65.03 | 5.83 | 58.71 | 87.63 | 5.98 | 81.22 |
| T26 | 65.37 | 5.73 | 59.64 | 88.05 | 5.76 | 82.29 |
| T50 | 64.93 | 5.82 | 58.72 | 88.07 | 5.89 | 81.83 |
| T72 | 65.78 | 5.35 | 60.04 | 88.06 | 5.40 | 82.34 |
| T90 | 62.79 | 5.94 | 56.85 | 85.48 | 5.87 | 79.61 |
| T104 | 64.99 | 6.06 | 58.56 | 86.80 | 6.15 | 80.32 |
| T118 | 66.02 | 5.77 | 59.89 | 88.38 | 5.85 | 82.22 |
| T148 | 70.09 | 5.51 | 64.58 | 93.12 | 5.47 | 87.65 |
| T150 | 63.91 | 5.52 | 58.40 | 86.31 | 5.50 | 80.81 |
| T194 | 63.44 | 5.34 | 58.10 | 86.10 | 5.34 | 80.76 |
| CHN | 119.77 | 9.87 | 109.90 | 142.34 | 9.96 | 132.38 |
| 10xmmar | 87.54 | 7.31 | 80.23 | 77.69 | 7.47 | 70.22 |
| S44 | 75.11 | 6.27 | 68.83 | 69.09 | 6.34 | 62.75 |
| S46 | 76.10 | 6.65 | 69.45 | 69.66 | 6.99 | 62.67 |
| S49 | 75.81 | 6.61 | 68.73 | 70.24 | 7.02 | 62.84 |
| T149 | 76.62 | 6.88 | 69.28 | 70.68 | 7.32 | 63.00 |
| T24 | 75.71 | 6.33 | 68.88 | 69.47 | 6.48 | 62.61 |
| T76 | 76.53 | 7.21 | 69.32 | 69.76 | 7.02 | 62.73 |
| T77 | 76.48 | 7.12 | 68.88 | 70.60 | 7.41 | 62.82 |
| T82 | 75.50 | 6.79 | 68.71 | 68.68 | 6.57 | 62.11 |
| T18 | 67.83 | 5.78 | 61.69 | 85.76 | 5.92 | 79.54 |
| S50 | 72.23 | 6.80 | 65.43 | 69.90 | 7.06 | 62.83 |
| T151 | 73.60 | 6.39 | 66.74 | 72.07 | 6.80 | 64.92 |
| T78 | 74.14 | 6.64 | 67.50 | 71.10 | 7.17 | 63.93 |
| T79 | 71.36 | 6.23 | 65.13 | 69.09 | 6.45 | 62.64 |
| T81 | 76.44 | 6.73 | 69.29 | 71.42 | 7.22 | 63.88 |
| T83 | 75.96 | 6.98 | 68.48 | 71.28 | 7.27 | 63.64 |
| T84 | 65.78 | 6.37 | 58.93 | 78.14 | 6.63 | 71.10 |
| T85 | 69.95 | 6.23 | 63.72 | 71.18 | 6.49 | 64.69 |
| T86 | 73.99 | 7.22 | 66.20 | 75.57 | 7.44 | 67.68 |
| T87 | 65.06 | 6.21 | 58.41 | 75.03 | 6.41 | 68.24 |

**Additional Table AT5**. Pseudoautosomal region (PAR) coordinates for all male samples for the sable and pine marten references.

Note that orientation of the chrX is different between the references, which affects the coordinates.

| **Sample** | **Sable reference** | | | **Pine marten reference** | | |
| --- | --- | --- | --- | --- | --- | --- |
|  | **start (bp)** | **stop (bp)** | **length (bp)** | **start (bp)** | **stop (bp)** | **length (bp)** |
| T8 | 200000 | 6680000 | 6480000 | 118000000 | 124450000 | 6450000 |
| T18 | 200000 | 6680000 | 6480000 | 118000000 | 124450000 | 6450000 |
| T50 | 200000 | 6680000 | 6480000 | 118000000 | 124450000 | 6450000 |
| T72 | 200000 | 6680000 | 6480000 | 118000000 | 124450000 | 6450000 |
| T104 | 200000 | 6680000 | 6480000 | 118000000 | 124450000 | 6450000 |
| T118 | 200000 | 6680000 | 6480000 | 118000000 | 124450000 | 6450000 |
| S49 | 200000 | 6680000 | 6480000 | 118000000 | 124450000 | 6450000 |
| T149 | 200000 | 6680000 | 6480000 | 118000000 | 124450000 | 6450000 |
| T151 | 200000 | 6680000 | 6480000 | 118000000 | 124450000 | 6450000 |
| T24 | 200000 | 6680000 | 6480000 | 118000000 | 124450000 | 6450000 |
| T77 | 200000 | 6680000 | 6480000 | 118000000 | 124450000 | 6450000 |
| T81 | 200000 | 6680000 | 6480000 | 118000000 | 124450000 | 6450000 |
| T83 | 200000 | 6680000 | 6480000 | 118000000 | 124450000 | 6450000 |
| T84 | 200000 | 6680000 | 6480000 | 118000000 | 124450000 | 6450000 |
| T86 | 200000 | 6680000 | 6480000 | 118000000 | 124450000 | 6450000 |
| T87 | 200000 | 6680000 | 6480000 | 118000000 | 124450000 | 6450000 |

**Additional Table AT6.** Comparison of global and local ancestry.

Cells are color-coded as follows: green and dark blue – pure sables and pure pine martens, respectively; yellow and blue – atypical sables and atypical martens, respectively; orange and light blue – backcross-like hybrids, respectively; and dark red – F1-like hybrids.

| **Sample** | **Global ancestry (% of sable)** | | | **Local ancestry (% of pine marten)** | | |
| --- | --- | --- | --- | --- | --- | --- |
|  | **sable**  **reference** | **pine marten**  **reference** | **Difference** | **sable**  **reference** | **pine marten**  **reference** | **Difference** |
| 10xmzib | 100 | 100 | 0 | 96.85 | 96.72 | 0.13 |
| S26 | 100 | 100 | 0 | 99.79 | 99.8 | 0.01 |
| T8 | 100 | 100 | 0 | 99.9 | 99.93 | 0.03 |
| T26 | 100 | 100 | 0 | 99.87 | 99.91 | 0.04 |
| T50 | 100 | 100 | 0 | 99.92 | 99.94 | 0.02 |
| T72 | 100 | 100 | 0 | 99.86 | 99.88 | 0.02 |
| T90 | 100 | 100 | 0 | 99.87 | 99.9 | 0.03 |
| T104 | 100 | 100 | 0 | 99.77 | 99.84 | 0.07 |
| T118 | 100 | 100 | 0 | 99.85 | 99.88 | 0.03 |
| T148 | 100 | 100 | 0 | 99.91 | 99.95 | 0.04 |
| T150 | 100 | 100 | 0 | 98.38 | 98.36 | 0.02 |
| T194 | 100 | 100 | 0 | 99.85 | 99.87 | 0.02 |
| CHN | 100 | 100 | 0 | 99.9 | 99.91 | 0.01 |
| 10xmmar | 0 | 0 | 0 | 3.02 | 2.8 | 0.22 |
| S44 | 0 | 0 | 0 | 3.57 | 3.39 | 0.18 |
| S46 | 0 | 0 | 0 | 1.69 | 1.66 | 0.03 |
| S49 | 0 | 0 | 0 | 4.17 | 3.95 | 0.22 |
| T149 | 0 | 0 | 0 | 2.52 | 2.39 | 0.13 |
| T24 | 0 | 0 | 0 | 3.55 | 3.49 | 0.06 |
| T76 | 0 | 0 | 0 | 2.51 | 2.42 | 0.09 |
| T77 | 0 | 0 | 0 | 3.76 | 3.59 | 0.17 |
| T82 | 0 | 0 | 0 | 2.02 | 1.93 | 0.09 |
| T18 | 84.5 | 83.9 | 0.6 | 83.55 | 83.51 | 0.04 |
| S50 | 12.6 | 12 | 0.6 | 16.21 | 15.76 | 0.45 |
| T151 | 14 | 13.4 | 0.6 | 18.09 | 17.85 | 0.24 |
| T78 | 6 | 5.9 | 0.1 | 9.96 | 9.72 | 0.24 |
| T79 | 12.9 | 12.5 | 0.4 | 17.6 | 17.31 | 0.29 |
| T81 | 0 | 0 | 0 | 5.92 | 5.58 | 0.34 |
| T83 | 0 | 0 | 0 | 7.48 | 7.27 | 0.21 |
| T84 | 67.8 | 66.8 | 1 | 70.14 | 69.19 | 0.95 |
| T85 | 22.5 | 21.6 | 0.9 | 26.64 | 25.83 | 0.81 |
| T86 | 19.6 | 18.7 | 0.9 | 23.1 | 22.51 | 0.59 |
| T87 | 59.6 | 58.5 | 1.1 | 62.62 | 61.55 | 1.07 |

**Additional Table AT7.** Heterozygous SNP counts (autosomes only) for the sable and pine marten references.
 Mean and median heterozygosity densities (hetSNPs/kbp) were calculated in 1 Mbp windows with 100 kbp step.

| **Sample** | **Reference: sable** | | | **Reference: pine marten** | | | **Difference** | | |
| --- | --- | --- | --- | --- | --- | --- | --- | --- | --- |
|  | **hetSNPs*, mln** | **Density, hetSNPs/kbp** | | **hetSNPs*, mln** | **Density, hetSNPs/kbp** | | **hetSNPs*, mln** | **Density, hetSNPs/kbp** | |
|  |  | **Mean** | **Median** |  | **Mean** | **Median** |  | **Mean** | **Median** |
| 10xmzib | 3.14 | 1.39 | 1.32 | 3.04 | 1.35 | 1.27 | 0.1 | 0.04 | 0.05 |
| S26 | 3.87 | 1.71 | 1.67 | 3.79 | 1.68 | 1.64 | 0.08 | 0.03 | 0.03 |
| T8 | 4.23 | 1.87 | 1.82 | 4.13 | 1.83 | 1.78 | 0.1 | 0.04 | 0.04 |
| T26 | 4.45 | 1.97 | 1.87 | 4.34 | 1.93 | 1.84 | 0.11 | 0.04 | 0.03 |
| T50 | 3.8 | 1.69 | 1.66 | 3.71 | 1.65 | 1.62 | 0.09 | 0.04 | 0.04 |
| T72 | 3.66 | 1.63 | 1.61 | 3.57 | 1.6 | 1.58 | 0.09 | 0.03 | 0.03 |
| T90 | 4.1 | 1.83 | 1.74 | 3.99 | 1.79 | 1.7 | 0.11 | 0.04 | 0.04 |
| T104 | 4.48 | 1.99 | 1.88 | 4.36 | 1.95 | 1.84 | 0.12 | 0.04 | 0.04 |
| T118 | 4.43 | 1.96 | 1.86 | 4.32 | 1.92 | 1.82 | 0.11 | 0.04 | 0.04 |
| T148 | 3.89 | 1.73 | 1.69 | 3.79 | 1.69 | 1.65 | 0.1 | 0.04 | 0.04 |
| T150 | 4.44 | 1.96 | 1.79 | 4.34 | 1.92 | 1.76 | 0.1 | 0.04 | 0.03 |
| T194 | 4.24 | 1.88 | 1.81 | 4.13 | 1.83 | 1.76 | 0.11 | 0.05 | 0.05 |
| CHN | 3.63 | 1.61 | 1.54 | 3.45 | 1.53 | 1.46 | 0.18 | 0.08 | 0.08 |
| 10xmmar | 1.86 | 0.8 | 0.55 | 1.92 | 0.83 | 0.57 | -0.06 | -0.03 | -0.02 |
| S44 | 2.28 | 1.01 | 0.65 | 2.33 | 1.03 | 0.67 | -0.05 | -0.02 | -0.02 |
| S46 | 2 | 0.87 | 0.62 | 2.04 | 0.89 | 0.64 | -0.04 | -0.02 | -0.02 |
| S49 | 2.37 | 1.05 | 0.65 | 2.41 | 1.06 | 0.67 | -0.04 | -0.01 | -0.02 |
| T149 | 2.04 | 0.9 | 0.62 | 2.08 | 0.91 | 0.63 | -0.04 | -0.01 | -0.01 |
| T24 | 2.2 | 0.97 | 0.61 | 2.24 | 0.99 | 0.62 | -0.04 | -0.02 | -0.01 |
| T76 | 2.1 | 0.91 | 0.63 | 2.15 | 0.93 | 0.65 | -0.05 | -0.02 | -0.02 |
| T77 | 2.31 | 0.99 | 0.64 | 2.35 | 1.01 | 0.66 | -0.04 | -0.02 | -0.02 |
| T82 | 1.95 | 0.86 | 0.63 | 2 | 0.88 | 0.65 | -0.05 | -0.02 | -0.02 |
| T18 | 5.29 | 2.36 | 2.05 | 5.2 | 2.32 | 2 | 0.09 | 0.04 | 0.05 |
| S50 | 4.46 | 1.96 | 0.86 | 4.48 | 1.98 | 0.89 | -0.02 | -0.02 | -0.03 |
| T151 | 4.75 | 2.08 | 0.87 | 4.77 | 2.1 | 0.91 | -0.02 | -0.02 | -0.04 |
| T78 | 3.43 | 1.52 | 0.71 | 3.47 | 1.55 | 0.74 | -0.04 | -0.03 | -0.03 |
| T79 | 4.5 | 2.01 | 0.98 | 4.52 | 2.02 | 1 | -0.02 | -0.01 | -0.02 |
| T81 | 2.67 | 1.16 | 0.66 | 2.7 | 1.17 | 0.68 | -0.03 | -0.01 | -0.02 |
| T83 | 2.79 | 1.22 | 0.65 | 2.82 | 1.23 | 0.67 | -0.03 | -0.01 | -0.02 |
| T84 | 7.52 | 3.36 | 3.7 | 7.47 | 3.34 | 3.67 | 0.05 | 0.02 | 0.03 |
| T85 | 6.09 | 2.72 | 2.97 | 6.1 | 2.73 | 2.95 | -0.01 | -0.01 | 0.02 |
| T86 | 5.51 | 2.45 | 1.45 | 5.53 | 2.47 | 1.55 | -0.02 | -0.02 | -0.1 |
| T87 | 8.88 | 3.97 | 4.3 | 8.85 | 3.96 | 4.3 | 0.03 | 0.01 | 0 |

* heterozygous SNPs

**Additional Table AT8.** RoH content for the sable and pine marten references.

| **Sample** | **Reference: sable** | | | | | | **Reference: pine marten** | | | | | |
| --- | --- | --- | --- | --- | --- | --- | --- | --- | --- | --- | --- | --- |
|  | **Number of RoHs** | **Total length, Mbp** | **% of genome** | **% of genome *** | | | **Number of RoHs** | **Total length, Mbp** | **% of genome** | **% of genome** | | |
|  |  |  |  | **Short** | **Long** | **Ultra Long** |  |  |  | **Short** | **Long** | **Ultra Long** |
| 10xmzib | 305 | 685.19 | 30.69 | 3.45 | 3.13 | 24.1 | 262 | 664.08 | 29.68 | 2.53 | 3.15 | 24 |
| S26 | 363 | 327.29 | 14.66 | 3.61 | 3.02 | 8.03 | 337 | 318.73 | 14.24 | 3.36 | 2.87 | 8.01 |
| T8 | 202 | 233.1 | 10.44 | 2.15 | 2.62 | 5.67 | 192 | 228.88 | 10.23 | 2 | 2.58 | 5.66 |
| T26 | 147 | 116.74 | 5.23 | 1.51 | 1.31 | 2.41 | 146 | 115.41 | 5.16 | 1.54 | 1.21 | 2.41 |
| T50 | 373 | 352.26 | 15.78 | 3.84 | 4.3 | 7.63 | 347 | 345.88 | 15.46 | 3.59 | 4.1 | 7.78 |
| T72 | 781 | 398.08 | 17.83 | 9.38 | 6.23 | 2.23 | 787 | 387.15 | 17.3 | 9.26 | 5.81 | 2.24 |
| T90 | 270 | 228.06 | 10.21 | 2.57 | 4.19 | 3.45 | 261 | 221.6 | 9.9 | 2.44 | 4.38 | 3.08 |
| T104 | 166 | 116.5 | 5.22 | 1.89 | 2.15 | 1.18 | 171 | 113.77 | 5.08 | 1.88 | 2.48 | 0.73 |
| T118 | 130 | 131.66 | 5.9 | 1.32 | 2.23 | 2.35 | 133 | 131.19 | 5.86 | 1.32 | 2.45 | 2.09 |
| T148 | 269 | 300.45 | 13.46 | 2.6 | 4.88 | 5.97 | 269 | 296.09 | 13.23 | 2.62 | 4.82 | 5.79 |
| T150 | 240 | 139.7 | 6.26 | 2.67 | 2.3 | 1.29 | 241 | 137.2 | 6.13 | 2.52 | 2.3 | 1.3 |
| T194 | 233 | 171.47 | 7.68 | 2.24 | 2.58 | 2.85 | 252 | 170.13 | 7.6 | 2.27 | 2.49 | 2.84 |
| CHN | 194 | 220.48 | 9.87 | 2.04 | 1.85 | 5.98 | 194 | 222.32 | 9.94 | 2.08 | 1.88 | 5.98 |
| 10xmmar | 535 | 483.75 | **21.66** | 5.35 | 4.54 | 11.77 | 622 | 522.4 | **23.35** | 6.58 | 5.04 | 11.72 |
| S44 | 643 | 269.36 | 12.06 | 6.66 | 3.89 | 1.52 | 670 | 284.16 | 12.7 | 6.87 | 4.31 | 1.52 |
| S46 | 679 | 243.34 | 10.9 | 6.86 | 4.04 | 0 | 720 | 260.85 | 11.66 | 7.37 | 4.28 | 0 |
| S49 | 619 | 312.86 | 14.01 | 6.25 | 5.71 | 2.05 | 659 | 329.92 | 14.74 | 6.82 | 5.84 | 2.08 |
| T149 | 660 | 353.12 | 15.81 | 6.85 | 6.33 | 2.63 | 693 | 371.42 | 16.6 | 7.31 | 6.64 | 2.65 |
| T24 | 612 | 390.56 | 17.49 | 5.98 | 6.78 | 4.73 | 656 | 410.92 | 18.36 | 6.71 | 6.93 | 4.73 |
| T76 | 666 | 336.25 | 15.06 | 7.03 | 4.42 | 3.61 | 679 | 348.95 | 15.59 | 7.22 | 4.9 | 3.47 |
| T77 | 640 | 303.38 | 13.59 | 6.99 | 5.79 | 0.81 | 676 | 324.84 | 14.52 | 7.59 | 5.66 | 1.26 |
| T82 | 670 | 283 | 12.67 | 6.59 | 6.08 | 0 | 704 | 303.73 | 13.57 | 6.98 | 6.59 | 0 |
| T18 | 268 | 97.63 | 4.37 | 2.73 | 1.14 | 0.5 | 272 | 98.72 | 4.41 | 2.86 | 1.06 | 0.49 |
| S50 | 445 | 164.18 | 7.35 | 4.37 | 2.98 | 0 | 461 | 175.56 | 7.85 | 4.72 | 3.13 | 0 |
| T151 | 406 | 378.36 | 16.94 | 4.09 | 4.53 | 8.32 | 430 | 390.7 | 17.46 | 4.45 | 4.54 | 8.46 |
| T78 | 535 | 212.31 | 9.51 | 5.48 | 3.5 | 0.52 | 581 | 225.95 | 10.1 | 5.93 | 3.65 | 0.52 |
| T79 | 477 | 244.06 | 10.93 | 5.21 | 3.87 | 1.85 | 497 | 258.04 | 11.53 | 5.49 | 4.19 | 1.85 |
| T81 | 643 | 260.52 | 11.67 | 6.82 | 3.77 | 1.07 | 688 | 279.74 | 12.5 | 7.37 | 4.05 | 1.08 |
| T83 | 597 | 360.3 | 16.14 | 6.38 | 4.16 | 5.6 | 630 | 374.7 | 16.75 | 6.57 | 5.06 | 5.12 |
| T84 | 166 | 61.14 | 2.74 | 1.73 | 1.01 | 0 | 173 | 61.13 | 2.73 | 1.74 | 1 | 0 |
| T85 | 385 | 159.89 | 7.16 | 3.79 | 3.37 | 0 | 395 | 166.64 | 7.45 | 4.05 | 3.4 | 0 |
| T86 | 371 | 147.26 | 6.6 | 3.72 | 2.88 | 0 | 407 | 158.18 | 7.07 | 4.08 | 2.99 | 0 |
| T87 | 130 | 39.01 | 1.75 | 1.25 | 0.5 | 0 | 131 | 38.24 | 1.71 | 1.35 | 0.36 | 0 |

* RoH categories: Short RoH, S (< 1 Mbp), Long RoH, L (>= 1 Mbp and < 10 Mbp) and Ultra Long RoH, UL (>= 10 Mbp).

**Additional Table AT9**. Comparison of ancestry based on STR loci for sable set (sable reference) and pine marten set (pine marten reference).

STR markers provided in Supplementary File 2. Cells are color-coded as follows: green and dark blue – pure sables and pure pine martens, respectively; yellow and blue – atypical sables and atypical martens, respectively; orange and light blue – backcross-like hybrids, respectively; and dark red – F1-like hybrids.

| **Sample** | **All STRs (% of sable)** | | | **Rozhnov’s (% of sable)** | | | **Kashtanov’s (% of sable)** | | |
| --- | --- | --- | --- | --- | --- | --- | --- | --- | --- |
|  | **Sable reference** | **Pine marten reference** | **Difference** | **Sable reference** | **Pine marten reference** | **Difference** | **Sable reference** | **Pine marten reference** | **Difference** |
| 10xmzib | 99.6 | 99.8 | 0.2 | 98.6 | 98.4 | 0.2 | 99.3 | 99.5 | 0.2 |
| S26 | 99.3 | 99.7 | 0.4 | 96.9 | 98.2 | 1.3 | 97.9 | 98.8 | 0.9 |
| T8 | 99.5 | 99.8 | 0.3 | 98.7 | 98.8 | 0.1 | 98.3 | 98.8 | 0.5 |
| T26 | 99.5 | 99.6 | 0.1 | 98.8 | 98.2 | 0.6 | 98.1 | 98.0 | 0.1 |
| T50 | 99.8 | 99.8 | 0 | 99.3 | 99.2 | 0.1 | 99.6 | 99.5 | 0.1 |
| T72 | 98.9 | 98.8 | 0.1 | 98.0 | 98.6 | 0.6 | 99.4 | 99.4 | 0 |
| T90 | 99.8 | 99.8 | 0 | 99.0 | 99.0 | 0 | 99.6 | 99.4 | 0.2 |
| T104 | 99.7 | 99.8 | 0.1 | 99.0 | 99.0 | 0 | 99.0 | 99.2 | 0.2 |
| T118 | 99.8 | 99.8 | 0 | 99.1 | 98.6 | 0.5 | 99.5 | 99.4 | 0.1 |
| T148 | 99.6 | 99.7 | 0.1 | 98.8 | 99.0 | 0.2 | 98.6 | 98.8 | 0.2 |
| T150 | 99.7 | 99.8 | 0.1 | 98.4 | 98.5 | 0.1 | 99.3 | 99.3 | 0 |
| T194 | 99.7 | 99.8 | 0.1 | 98.9 | 99.1 | 0.2 | 99.5 | 99.5 | 0 |
| CHN | 99.7 | 99.8 | 0.1 | 98.7 | 98.8 | 0.1 | 99.3 | 99.4 | 0.1 |
| 10xmmar | 0.9 | 0.6 | 0.3 | 4.4 | 5.8 | 1.4 | 4.0 | 3.4 | 0.6 |
| S44 | 0.3 | 0.2 | 0.1 | 1.0 | 1.1 | 0.1 | 0.4 | 0.4 | 0 |
| S46 | 0.4 | 0.2 | 0.2 | 2.4 | 2.5 | 0.1 | 0.8 | 0.8 | 0 |
| S49 | 0.8 | 0.5 | 0.3 | 1.4 | 1.3 | 0.1 | 0.9 | 1.0 | 0.1 |
| T149 | 0.4 | 0.3 | 0.1 | 1.0 | 1.6 | 0.6 | 0.9 | 1.1 | 0.2 |
| T24 | 0.2 | 0.2 | 0 | 1.7 | 1.5 | 0.2 | 0.7 | 0.6 | 0.1 |
| T76 | 0.4 | 0.2 | 0.2 | 1.8 | 1.4 | 0.4 | 0.6 | 0.5 | 0.1 |
| T77 | 1.4 | 0.6 | 0.8 | 1.0 | 0.9 | 0.1 | 1.1 | 1.0 | 0.1 |
| T82 | 0.2 | 0.2 | 0 | 0.8 | 0.9 | 0.1 | 0.6 | 0.6 | 0 |
| T18 | 99.3 | 99.4 | 0.1 | 98.8 | 98.4 | 0.4 | 99.2 | 99.0 | 0.2 |
| S50 | 10.6 | 6.6 | 4 | 5.8 | 5.6 | 0.2 | 8.6 | 11.5 | 2.9 |
| T151 | 0.8 | 0.7 | 0.1 | 4.9 | 3.2 | 1.7 | 1.0 | 1.1 | 0.1 |
| T78 | 0.8 | 0.5 | 0.3 | 1.9 | 1.6 | 0.3 | 2.1 | 1.8 | 0.3 |
| T79 | 6.1 | 5.7 | 0.4 | 5.0 | 4.0 | 1 | 9.4 | 10.6 | 1.2 |
| T81 | 0.5 | 0.3 | 0.2 | 2.4 | 2.2 | 0.2 | 0.7 | 0.9 | 0.2 |
| T83 | 0.9 | 0.7 | 0.2 | 10.3 | 9.7 | 0.6 | 3.3 | 3.0 | 0.3 |
| T84 | 63.4 | 68.2 | 4.8 | 93.1 | 81.4 | 11.7 | 95.8 | 94.2 | 1.6 |
| T85 | 27.8 | 30.3 | 2.5 | 15.5 | 22.9 | 7.4 | 2.9 | 2.4 | 0.5 |
| T86 | 5.1 | 4.4 | 0.7 | 11.0 | 9.2 | 1.8 | 9.0 | 10.8 | 1.8 |
| T87 | 58.5 | 57.4 | 1.1 | 44.3 | 42.5 | 1.8 | 46.1 | 56.5 | 10.4 |

**Additional Table AT10**. Comparison of ancestry based on STR markers common for the pine marten and sable references

STR markers provided in Supplementary File 2. Cells are color-coded as follows: green and dark blue – pure sables and pure pine martens, respectively; yellow and blue – atypical sables and atypical martens, respectively; orange and light blue – backcross-like hybrids, respectively; and dark red – F1-like hybrids.

| **Sample** | **All STRs (% of sable)** | | | **Rozhnov’s (% of sable)** | | | **Kashtanov’s (% of sable)** | | |
| --- | --- | --- | --- | --- | --- | --- | --- | --- | --- |
|  | **Sable reference** | **Pine marten reference** | **Difference** | **Sable reference** | **Pine marten reference** | **Difference** | **Sable reference** | **Pine marten reference** | **Difference** |
| 10xmzib | 99.6 | 99.7 | 0.1 | 98.6 | 98.5 | 0.1 | 99.3 | 99.4 | 0.1 |
| S26 | 99.3 | 99.5 | 0.2 | 96.9 | 98.4 | 1.5 | 97.9 | 98.4 | 0.5 |
| T8 | 99.5 | 99.5 | 0.0 | 98.7 | 98.4 | 0.3 | 98.3 | 98.5 | 0.2 |
| T26 | 99.5 | 99.4 | 0.1 | 98.8 | 98.4 | 0.4 | 98.1 | 98.0 | 0.1 |
| T50 | 99.8 | 99.8 | 0.0 | 99.3 | 99.2 | 0.1 | 99.6 | 99.6 | 0.0 |
| T72 | 98.9 | 98.9 | 0.0 | 98.0 | 98.2 | 0.2 | 99.4 | 99.3 | 0.1 |
| T90 | 99.8 | 99.8 | 0.0 | 99.0 | 98.9 | 0.1 | 99.6 | 99.5 | 0.1 |
| T104 | 99.7 | 99.7 | 0.0 | 99.0 | 98.8 | 0.2 | 99.0 | 99.0 | 0.0 |
| T118 | 99.8 | 99.8 | 0.0 | 99.1 | 99.0 | 0.1 | 99.5 | 99.4 | 0.1 |
| T148 | 99.6 | 99.5 | 0.1 | 98.8 | 98.6 | 0.2 | 98.6 | 98.5 | 0.1 |
| T150 | 99.7 | 99.7 | 0.0 | 98.4 | 98.1 | 0.3 | 99.3 | 99.2 | 0.1 |
| T194 | 99.7 | 99.7 | 0.0 | 98.9 | 98.9 | 0.0 | 99.5 | 99.5 | 0.0 |
| CHN | 99.7 | 99.7 | 0.0 | 98.7 | 98.5 | 0.2 | 99.3 | 99.3 | 0.0 |
| 10xmmar | 0.9 | 0.7 | 0.2 | 4.4 | 5.3 | 0.9 | 4.0 | 3.5 | 0.5 |
| S44 | 0.3 | 0.3 | 0.0 | 1.0 | 1.1 | 0.1 | 0.4 | 0.5 | 0.1 |
| S46 | 0.4 | 0.4 | 0.0 | 2.4 | 3.0 | 0.6 | 0.8 | 1.0 | 0.2 |
| S49 | 0.8 | 0.8 | 0.0 | 1.4 | 1.6 | 0.2 | 0.9 | 1.0 | 0.1 |
| T149 | 0.4 | 0.5 | 0.1 | 1.0 | 1.8 | 0.8 | 0.9 | 1.3 | 0.4 |
| T24 | 0.2 | 0.2 | 0.0 | 1.7 | 2.0 | 0.3 | 0.7 | 0.7 | 0.0 |
| T76 | 0.4 | 0.4 | 0.0 | 1.8 | 1.5 | 0.3 | 0.6 | 0.5 | 0.1 |
| T77 | 1.4 | 1.4 | 0.0 | 1.0 | 1.1 | 0.1 | 1.1 | 1.2 | 0.1 |
| T82 | 0.2 | 0.3 | 0.1 | 0.8 | 0.9 | 0.1 | 0.6 | 0.6 | 0.0 |
| T18 | 99.3 | 99.2 | 0.1 | 98.8 | 98.5 | 0.3 | 99.2 | 99.1 | 0.1 |
| S50 | 10.6 | 11.4 | 0.8 | 5.8 | 6.5 | 0.7 | 8.6 | 10.3 | 1.7 |
| T151 | 0.8 | 0.7 | 0.1 | 4.9 | 4.1 | 0.8 | 1.0 | 0.9 | 0.1 |
| T78 | 0.8 | 0.8 | 0.0 | 1.9 | 2.0 | 0.1 | 2.1 | 2.1 | 0.0 |
| T79 | 6.1 | 7.1 | 1.0 | 5.0 | 6.0 | 1.0 | 9.4 | 10.8 | 1.4 |
| T81 | 0.5 | 0.5 | 0.0 | 2.4 | 2.7 | 0.3 | 0.7 | 0.8 | 0.1 |
| T83 | 0.9 | 1.0 | 0.1 | 10.3 | 10.1 | 0.2 | 3.3 | 3.7 | 0.4 |
| T84 | 63.4 | 65.0 | 1.6 | 93.1 | 94.3 | 1.2 | 95.8 | 92.3 | 3.5 |
| T85 | 27.8 | 22.9 | 4.9 | 15.5 | 11.0 | 4.5 | 2.9 | 1.9 | 1.0 |
| T86 | 5.1 | 5.9 | 0.8 | 11.0 | 11.6 | 0.6 | 9.0 | 10.3 | 1.3 |
| T87 | 58.5 | 58.5 | 0.0 | 44.3 | 48.7 | 4.4 | 46.1 | 49.7 | 3.6 |

**Additional Table AT11**. Species used for whole-genome phylogenetic reconstruction and dating.

| **Species** | **Level of assembly** | **ID** | **Source** |
| --- | --- | --- | --- |
| *Martes martes* | Chromosome | DNAzoo | [(A.A. Tomarovsky et al. 2025)](https://www.zotero.org/google-docs/?pjKDUE) |
| *Martes zibellina* | Chromosome | DNAzoo | [(A.A. Tomarovsky et al. 2025)](https://www.zotero.org/google-docs/?gOWxrP) |
| *Martes foina* | Chromosome | DNAzoo | [(A. Tomarovsky et al. 2025)](https://www.zotero.org/google-docs/?8PowD9) |
| *Mustela putorius furo* | Chromosome | DNAzoo | [(Peng et al. 2014; Dudchenko et al. 2017; Dudchenko et al. 2018)](https://www.zotero.org/google-docs/?XWQlBG) |
| *Mustela nigripes* | Chromosome | DNAzoo | [(Kliver et al. 2023)](https://www.zotero.org/google-docs/?4gR4Tk) |
| *Mustela nivalis* | Scaffold | GCA_019141155.1 | [(Miranda et al. 2021)](https://www.zotero.org/google-docs/?03ZvMZ) |
| *Mustela erminea* | Chromosome | GCA_009829155.1 | [(Rhie et al. 2021)](https://www.zotero.org/google-docs/?Fv1cjC) |
| *Neogale vison* | Chromosome | GCA_020171115.1 | [(Karimi et al. 2022)](https://www.zotero.org/google-docs/?wO9YMv) |
| *Gulo gulo* | PseudoChromosome | GCA_024510155.1 | [(Lok et al. 2022)](https://www.zotero.org/google-docs/?KA2FUH) |
| *Meles meles* | Chromosome | GCA_922984935.1 | [(Newman et al. 2022)](https://www.zotero.org/google-docs/?t1SMtH) |
| *Ailurus fulgens* | Chromosome | DNAzoo | [(Dudchenko et al. 2017; Hu et al. 2017; Dudchenko et al. 2018)](https://www.zotero.org/google-docs/?2loV8L) |
| *Enhydra lutris* | Chromosome | DNAzoo | [(Dudchenko et al. 2017; Jones et al. 2017; Dudchenko et al. 2018)](https://www.zotero.org/google-docs/?1hxWVV) |
| *Erignathus barbatus* | Chromosome | DNAzoo | [(Dudchenko et al. 2017)](https://www.zotero.org/google-docs/?PZuQzK) |
| *Lontra canadensis* | Chromosome | DNAzoo | [(Dudchenko et al. 2017)](https://www.zotero.org/google-docs/?KNUQO4) |
| *Neomonachus schauinslandi* | Chromosome | GCA_002201575.2 | [(Mohr et al. 2022)](https://www.zotero.org/google-docs/?VDyGR7) |
| *Odobenus rosmarus* | Chromosome | DNAzoo | [(Foote et al. 2015; Dudchenko et al. 2017; Dudchenko et al. 2018)](https://www.zotero.org/google-docs/?71RqPa) |
| *Ursus arctos* | Chromosome | DNAzoo | [(Dudchenko et al. 2017; Dudchenko et al. 2018; Taylor et al. 2018)](https://www.zotero.org/google-docs/?8dGAXM) |
| *Canis lupus familiaris* | Chromosome | DNAzoo | [(Dudchenko et al. 2017; Dudchenko et al. 2018)](https://www.zotero.org/google-docs/?2hOBec) |
| *Mustela eversmanii* | Scaffold | GCA_963422785.1 | [(Derežanin et al. in prep)](https://www.zotero.org/google-docs/?MhvDPx) |
| *Eira barbara* | Scaffold | GCA_020311275.1 | [(Derežanin et al. 2022)](https://www.zotero.org/google-docs/?dzu8KD) |
| *Vulpes lagopus* | Chromosome | GCA_018345385.1 | [(Peng et al. 2021)](https://www.zotero.org/google-docs/?0R2Iwo) |

# 5. Supplementary Files

The supplementary files are attached to the manuscript as a single archive SupplementaryFiles.zip

## Supplementary File 1. HyDe, F3- and D-stastistics

This Excel file contains three sheets: *HyDe*, *F3* and *D*. The sheet *HyDe* contains the results of HyDe analysis for the pure sables and the pure pine martens as parental groups (P1 and P2, respectively) and hybrids as a target. The tests were performed for both all hybrids together as a group and each individual separately. The sheet *F3*  contains tests for all possible permutations of source and target groups for the hybrids (together as a group) and the pure species, as well as tests for each individual as a target (the pure sables as the source 1, the pure pine martens as a source 2). The sheet *D* contains analysis of the D-statistics. For these calculations, *M. foina* was designated as Z (outgroup) for all tests. Like for F3-statistics, we first analyzed all permutations of the pure species and hybrids as W, X, and Y groups. For individual level tests a target individual was always used as W, whereas the pure sables and the pure pine martens were first used as X anY, and then rearranged.

## Supplementary File 2. Description of the genotyped STR loci.

This Excel file contains two sheets: *sable set* and *pine marten set*, representing the STR loci successfully genotyped in the sable and pine marten reference genomes, respectively. Each sheet includes detailed information on the STR markers, including locus ID, coordinates of the whole loci, amplicon length, repeat motif, monomer sequence, number of repeat copies, coordinates of the repeat, and the original repeat motif. Additionally, the table indicates whether each STR locus was previously used in [(Rozhnov et al. 2013)](https://www.zotero.org/google-docs/?i4sxJb) and [(S. Kashtanov et al. 2022)](https://www.zotero.org/google-docs/?ijD4Bs).

## Supplementary File 3. Description of the used mtDNA data.

This Excel file provides information on all mitochondrial DNA sequences used in this study.

## Supplementary File 4. Mitochondrial haplotype network (large scale figure).

This file contains a full-size version of MT Figure 3, showing the median-joining network of mitochondrial genome haplotypes. The circle sizes are proportional to haplotype frequencies, and distances between haplotypes correspond to the number of substitutions between them. Sable subspecies are indicated by different colors. Substitution counts between all haplotypes are shown as numbers. *M. foina* (NC_020643.1) was used as an outgroup (not shown).

## Supplementary File 5. Model fitting statistics for Heterozygosity Component Analysis (HCA).

The table provides the results of Heterozygosity Component Analysis (HCA) for all samples. For each sample, it lists the optimal model, quadratic deviation (QDE) from the empirical heterozygosity distribution, and the mean and mode values for each component (pine marten (P), sable (S), and hybrid (H)).

## Supplementary File 6. Distribution of the heterozygous and homozygous along chromosomes.

SNPs were counted in 1 Mbp windows with a 100 kbp step size and scaled to hetSNPs/kbp (represented by the color scale at the bottom, ranging from dark blue for an extremely low heterozygosity to brown for a very high heterozygosity, from 0 - 0.1 up to over 7 hetSNPs per 1 kbp). The distributions are shown relative to both the pine marten and the sable genome assemblies.

## Supplementary File 7. Local ancestry along chromosomes.

The local ancestry (% of sable) was calculated in 1 Mbp sliding windows with a 100 kbp step (green – sable, blue – pine marten, red – hybrid), relative to both the pine marten and the sable genome assemblies.

## Supplementary File 8. Runs of homozygosity (RoH).

This file contains RoHs plotted along chromosomes for all samples relative to both the pine marten genome and sable assemblies.

## Supplementary File 9. GO-analysis and description of related genes for regions of interest.

This file contains multiple sheets corresponding to various regions of interest: (1) *Inversion*  – inversion between the pine marten and the sable, encompassing the p-arm of chr11*;* (2) *Fst (>=0.9)* - regions showed high Fst values in comparison between the pure pine martens and the pure sables*, (3) Tajima’s D (all samples, >2)* – regions showed Tajima’s D >2 for dataset including all the samples, and (4) *Tajima's D (Pure pine martens, < -2)* – regions showed Tajima’s D < -2 for dataset including only the pure pine martens. Each sheet lists genes located within the respective regions and includes locus ID, scaffold, start and end coordinates, gene name, description, other designations, and a summary. The final sheet, *GO enrichment*, presents the results of Gene Ontology (GO) enrichment analysis for these regions, including GO term, term label, fold enrichment, expected count, raw P-value, and FDR.

## Supplementary File 10. Phylogenetic trees in NEWICK format.

This archive contains phylogenetic trees in NEWICK format obtained using IQ-tree, MrBayes, and Astral. The directory *dating* includes time-calibrated phylogenetic trees using three molecular clock types: global, correlated, and independent.

## Supplementary File 11. In silico PCR of STR loci.

This table contains the results of *in silico* PCR for the genotyped STR loci, including primer pair ID, source, minimum and maximum expected product length, and amplification success for sable and pine marten reference.

## Supplementary File 12. Input data for haplotype network reconstruction (PopArt).

# 6. References

[Abramov A, Puzachenko AY. 2007. Possible hybridization between Meles meles and M. leucurus (Carnivora, Mustelidae) in Western Tien Shan. *Mamm. Mt. Territ.*:4–7.](https://www.zotero.org/google-docs/?F5fpxR)

[Abramov AV, Tumanov IL. 2003. Sexual dimorphism in the skull of the European mink Mustela lutreola from NW part of Russia. *Acta Theriol. (Warsz.)* 48:239–246.](https://www.zotero.org/google-docs/?F5fpxR)

[Bajo P, Drysdale RN, Woodhead JD, Hellstrom JC, Hodell D, Ferretti P, Voelker AHL, Zanchetta G, Rodrigues T, Wolff E, et al. 2020. Persistent influence of obliquity on ice age terminations since the Middle Pleistocene transition. *Science* 367:1235–1239.](https://www.zotero.org/google-docs/?F5fpxR)

[Basto MP, Rodrigues M, Santos-Reis M, Bruford MW, Fernandes CA. 2010a. Isolation and characterization of 13 tetranucleotide microsatellite loci in the Stone marten (Martes foina). *Conserv. Genet. Resour.* 2:317–319.](https://www.zotero.org/google-docs/?F5fpxR)

[Basto MP, Rodrigues M, Santos-Reis M, Bruford MW, Fernandes CA. 2010b. Isolation and characterization of 13 tetranucleotide microsatellite loci in the Stone marten (Martes foina). *Conserv. Genet. Resour.* 2:317–319.](https://www.zotero.org/google-docs/?F5fpxR)

[Behr AA, Liu KZ, Liu-Fang G, Nakka P, Ramachandran S. 2016. pong: fast analysis and visualization of latent clusters in population genetic data. *Bioinformatics* 32:2817–2823.](https://www.zotero.org/google-docs/?F5fpxR)

[Benson G. 1999. Tandem repeats finder: a program to analyze DNA sequences. *Nucleic Acids Res.* 27:573–580.](https://www.zotero.org/google-docs/?F5fpxR)

[Bergeron LA, Besenbacher S, Zheng J, Li P, Bertelsen MF, Quintard B, Hoffman JI, Li Z, St. Leger J, Shao C, et al. 2023. Evolution of the germline mutation rate across vertebrates. *Nature* 615:285–291.](https://www.zotero.org/google-docs/?F5fpxR)

[Cahill JA, Soares AER, Green RE, Shapiro B. 2016. Inferring species divergence times using pairwise sequential Markovian coalescent modelling and low-coverage genomic data. *Philos. Trans. R. Soc. B Biol. Sci.* 371:20150138.](https://www.zotero.org/google-docs/?F5fpxR)

[Carlson CS, Thomas DJ, Eberle MA, Swanson JE, Livingston RJ, Rieder MJ, Nickerson DA. 2005. Genomic regions exhibiting positive selection identified from dense genotype data. *Genome Res.* 15:1553–1565.](https://www.zotero.org/google-docs/?F5fpxR)

[Castresana J. 2000. Selection of Conserved Blocks from Multiple Alignments for Their Use in Phylogenetic Analysis. *Mol. Biol. Evol.* 17:540–552.](https://www.zotero.org/google-docs/?F5fpxR)

[Cheprasov MY, Mordosov II. 2019. Ecology of the Sable in the Middle Kolyma River Basin. *Yakutsk Publ. House NEFU*:143.](https://www.zotero.org/google-docs/?F5fpxR)

[Clark PU, Archer D, Pollard D, Blum JD, Rial JA, Brovkin V, Mix AC, Pisias NG, Roy M. 2006. The middle Pleistocene transition: characteristics, mechanisms, and implications for long-term changes in atmospheric pCO2. *Quat. Sci. Rev.* 25:3150–3184.](https://www.zotero.org/google-docs/?F5fpxR)

[Cook DE, Andersen EC. 2017. VCF-kit: assorted utilities for the variant call format. *Bioinformatics* 33:1581–1582.](https://www.zotero.org/google-docs/?F5fpxR)

[Danecek P, Auton A, Abecasis G, Albers CA, Banks E, DePristo MA, Handsaker RE, Lunter G, Marth GT, Sherry ST, et al. 2011. The variant call format and VCFtools. *Bioinformatics* 27:2156–2158.](https://www.zotero.org/google-docs/?F5fpxR)

[Danecek P, Bonfield JK, Liddle J, Marshall J, Ohan V, Pollard MO, Whitwham A, Keane T, McCarthy SA, Davies RM. 2021. Twelve years of SAMtools and BCFtools. *Gigascience* 10:giab008.](https://www.zotero.org/google-docs/?F5fpxR)

[Davis CS, Strobeck C. 1998a. Isolation, variability, and cross-species amplification of polymorphic microsatellite loci in the family Mustelidae. *Mol. Ecol.* 7:1776–1778.](https://www.zotero.org/google-docs/?F5fpxR)

[Davis CS, Strobeck C. 1998b. Isolation, variability, and cross-species amplification of polymorphic microsatellite loci in the family Mustelidae. *Mol. Ecol.* 7:1776–1778.](https://www.zotero.org/google-docs/?F5fpxR)

[Davison A, Birks JDS, Brookes RC, Messenger JE, Griffiths HI. 2001. Mitochondrial phylogeography and population history of pine martens Martes martes compared with polecats Mustela putorius. *Mol. Ecol.* 10:2479–2488.](https://www.zotero.org/google-docs/?F5fpxR)

[Derežanin L, Blažytė A, Dobrynin P, Duchêne DA, Grau JH, Jeon S, Kliver S, Koepfli K-P, Meneghini D, Preick M, et al. 2022. Multiple types of genomic variation contribute to adaptive traits in the mustelid subfamily Guloninae. *Mol. Ecol.* 31:2898–2919.](https://www.zotero.org/google-docs/?F5fpxR)

[Derežanin L, Safonova Y, Kliver S, Fontsere C, Totikov AA, Tomarovsky AA, Etherington G, Haerty W, Di Palma F, Perelman PL, et al. in prep. Comparative analyses reveal the genomic scars of the severe bottleneck in the endangered black-footed ferret.](https://www.zotero.org/google-docs/?F5fpxR)

[Domingo-Roura X. 2002. Genetic Distinction of Marten Species by Fixation of a Microsatellite Region. *J. Mammal.* 83:907–912.](https://www.zotero.org/google-docs/?F5fpxR)

[Dudchenko O, Batra SS, Omer AD, Nyquist SK, Hoeger M, Durand NC, Shamim MS, Machol I, Lander ES, Aiden AP. 2017. De novo assembly of the Aedes aegypti genome using Hi-C yields chromosome-length scaffolds. *Science* 356:92–95.](https://www.zotero.org/google-docs/?F5fpxR)

[Dudchenko O, Shamim MS, Batra SS, Durand NC, Musial NT, Mostofa R, Pham M, Hilaire BGS, Yao W, Stamenova E, et al. 2018. The Juicebox Assembly Tools module facilitates de novo assembly of mammalian genomes with chromosome-length scaffolds for under $1000. *bioRxiv*:254797.](https://www.zotero.org/google-docs/?F5fpxR)

[Elson E, Perveen R, Donnai D, Wall S, Black GCM. 2002. De novo GLI3 mutation in acrocallosal syndrome: broadening the phenotypic spectrum of GLI3 defects and overlap with murine models. *J. Med. Genet.* 39:804–806.](https://www.zotero.org/google-docs/?F5fpxR)

[Fleming MA, Ostrander EA, Cook JA. 1999. Microsatellite markers for American mink (Mustela vison) and ermine (Mustela erminea). *Mol. Ecol.* 8:1352–1355.](https://www.zotero.org/google-docs/?F5fpxR)

[Fleming, Ostrander, Cook. 1999. Microsatellite markers for American mink (Mustela vison) and ermine (Mustela erminea). *Mol. Ecol.*:1352–1354.](https://www.zotero.org/google-docs/?F5fpxR)

[Foote AD, Liu Y, Thomas GWC, Vinař T, Alföldi J, Deng J, Dugan S, van Elk CE, Hunter ME, Joshi V, et al. 2015. Convergent evolution of the genomes of marine mammals. *Nat. Genet.* 47:272–275.](https://www.zotero.org/google-docs/?F5fpxR)

[Hassanin A, Veron G, Ropiquet A, Vuuren BJ van, Lécu A, Goodman SM, Haider J, Nguyen TT. 2021. Evolutionary history of Carnivora (Mammalia, Laurasiatheria) inferred from mitochondrial genomes. *PLOS ONE* 16:e0240770.](https://www.zotero.org/google-docs/?F5fpxR)

[Hazard SE, Patel SB. 2007. Sterolins ABCG5 and ABCG8: regulators of whole body dietary sterols. *Pflüg. Arch. - Eur. J. Physiol.* 453:745–752.](https://www.zotero.org/google-docs/?F5fpxR)

[Helldin JO. 2000. Seasonal diet of pine marten Martes martes in southern boreal Sweden. *Acta Theriol. (Warsz.)* 45:409–420.](https://www.zotero.org/google-docs/?F5fpxR)

[Heptner V, Naumov N, Yurgenson P, Sludskiy A, Chirkova A, Bannikov A. 1967. Mammals of Soviet Union, Vol. 2 (1) Sea cows and Carnivora. *Vysshaya Shkola Mosc.*](https://www.zotero.org/google-docs/?F5fpxR)

[Herrero J, Kranz A, Skumatov D, Abramov AV, Maran T, Monakhov VG. 2015. IUCN Red List of Threatened Species: Martes martes. *IUCN Red List Threat. Species*.](https://www.zotero.org/google-docs/?F5fpxR)

[Hu Y, Wu Q, Ma S, Ma T, Shan L, Wang X, Nie Y, Ning Z, Yan L, Xiu Y, et al. 2017. Comparative genomics reveals convergent evolution between the bamboo-eating giant and red pandas. *Proc. Natl. Acad. Sci.* 114:1081–1086.](https://www.zotero.org/google-docs/?F5fpxR)

[Huerta-Cepas J, Serra F, Bork P. 2016. ETE 3: Reconstruction, Analysis, and Visualization of Phylogenomic Data. *Mol. Biol. Evol.* 33:1635–1638.](https://www.zotero.org/google-docs/?F5fpxR)

[Jakobsson M, Rosenberg NA. 2007. CLUMPP: a cluster matching and permutation program for dealing with label switching and multimodality in analysis of population structure. *Bioinformatics* 23:1801–1806.](https://www.zotero.org/google-docs/?F5fpxR)

[Jiangzuo Q, Gimranov D, Liu Jinyuan, Liu S, Jin C, Liu Jinyi. 2021. A new fossil marten from Jinyuan Cave, northeastern China reveals the origin of the Holarctic marten group. *Quat. Int.* [Internet] 591:47–58. Available from: https://www.sciencedirect.com/science/article/pii/S1040618220307102](https://www.zotero.org/google-docs/?F5fpxR)

[Jones SJ, Haulena M, Taylor GA, Chan S, Bilobram S, Warren RL, Hammond SA, Mungall KL, Choo C, Kirk H, et al. 2017. The Genome of the Northern Sea Otter (Enhydra lutris kenyoni). *Genes* 8:379.](https://www.zotero.org/google-docs/?F5fpxR)

[Kalff-Suske M, Wild A, Topp J, Wessling M, Jacobsen E-M, Bornholdt D, Engel H, Heuer H, Aalfs CM, Ausems MGEM, et al. 1999. Point Mutations Throughout the GLI3 Gene Cause Greig Cephalopolysyndactyly Syndrome. *Hum. Mol. Genet.* 8:1769–1777.](https://www.zotero.org/google-docs/?F5fpxR)

[Kalyaanamoorthy S, Minh BQ, Wong TKF, von Haeseler A, Jermiin LS. 2017. ModelFinder: fast model selection for accurate phylogenetic estimates. *Nat. Methods* 14:587–589.](https://www.zotero.org/google-docs/?F5fpxR)

[Kargopoulos N, Valenciano A, Abella J, Kampouridis P, Lechner T, Böhme M. 2022. The exceptionally high diversity of small carnivorans from the Late Miocene hominid locality of Hammerschmiede (Bavaria, Germany). *PloS One* 17:e0268968.](https://www.zotero.org/google-docs/?F5fpxR)

[Karimi K, Do DN, Wang J, Easley J, Borzouie S, Sargolzaei M, Plastow G, Wang Z, Miar Y. 2022. A chromosome-level genome assembly reveals genomic characteristics of the American mink (Neogale vison). *Commun. Biol.* 5:1–11.](https://www.zotero.org/google-docs/?F5fpxR)

[Kashtanov S, Zakharov E, Begletsov O, Svishcheva G, Rychkov SY, Filimonov P, Onokhov A, Levenkova E, Meschersky I, Rozhnov V. 2022. Expansion of the sable (Martes zibellina L.) from the north of the Central Siberian Plateau into tundra ecosystems. *Russ. J. Genet.* 58:955–966.](https://www.zotero.org/google-docs/?F5fpxR)

[Kashtanov SN, Zakharov ES, Begletsov OA, Svishcheva GR, Rychkov SYu, Filimonov PA, Onokhov AA, Levenkova ES, Meschersky IG, Rozhnov VV. 2022. Expansion of the Sable (Martes zibellina L.) from the North of the Central Siberian Plateau into Tundra Ecosystems. *Russ. J. Genet.* 58:955–966.](https://www.zotero.org/google-docs/?F5fpxR)

[Kliver S, Houck ML, Perelman PL, Totikov A, Tomarovsky A, Dudchenko O, Omer AD, Colaric Z, Weisz D, Aiden EL, et al. 2023. Chromosome-length genome assembly and karyotype of the endangered black-footed ferret (Mustela nigripes). *J. Hered.* 114:539–548.](https://www.zotero.org/google-docs/?F5fpxR)

[Koepfli K-P, Deere KA, Slater GJ, Begg C, Begg K, Grassman L, Lucherini M, Veron G, Wayne RK. 2008. Multigene phylogeny of the Mustelidae: Resolving relationships, tempo and biogeographic history of a mammalian adaptive radiation. *BMC Biol.* 6:10.](https://www.zotero.org/google-docs/?F5fpxR)

[Law CJ, Slater GJ, Mehta RS. 2018. Lineage Diversity and Size Disparity in Musteloidea: Testing Patterns of Adaptive Radiation Using Molecular and Fossil-Based Methods. *Syst. Biol.* 67:127–144.](https://www.zotero.org/google-docs/?F5fpxR)

[Li B, Wolsan M, Wu D, Zhang W, Xu Y, Zeng Z. 2014. Mitochondrial genomes reveal the pattern and timing of marten (Martes), wolverine (Gulo), and fisher (Pekania) diversification. *Mol. Phylogenet. Evol.* 80:156–164.](https://www.zotero.org/google-docs/?F5fpxR)

[Li H, Durbin R. 2009. Fast and accurate short read alignment with Burrows–Wheeler transform. *Bioinformatics* 25:1754–1760.](https://www.zotero.org/google-docs/?F5fpxR)

[Li H, Handsaker B, Wysoker A, Fennell T, Ruan J, Homer N, Marth G, Abecasis G, Durbin R. 2009. The Sequence Alignment/Map format and SAMtools. *Bioinformatics* 25:2078–2079.](https://www.zotero.org/google-docs/?F5fpxR)

[Lok S, Lau TNH, Trost B, Tong AHY, Wintle RF, Engstrom MD, Stacy E, Waits LP, Scrafford M, Scherer SW. 2022. Chromosomal-level reference genome assembly of the North American wolverine (Gulo gulo luscus): a resource for conservation genomics. *G3 GenesGenomesGenetics* 12:jkac138.](https://www.zotero.org/google-docs/?F5fpxR)

[Löytynoja A. 2014. Phylogeny-aware alignment with PRANK. In: Russell DJ, editor. Multiple Sequence Alignment Methods. Totowa, NJ: Humana Press. p. 155–170.](https://www.zotero.org/google-docs/?F5fpxR)

[Macdonald P. 2018. mixdist: Finite Mixture Distribution Models. Available from: https://CRAN.R-project.org/package=mixdist](https://www.zotero.org/google-docs/?F5fpxR)

[Manni M, Berkeley MR, Seppey M, Zdobnov EM. 2021. BUSCO: Assessing Genomic Data Quality and Beyond. *Curr. Protoc.* 1:e323.](https://www.zotero.org/google-docs/?F5fpxR)

[Marciszak A, Lipecki G, Spassov N. 2024. Martes wenzensis Stach, 1959 within the early history of the genus Martes Pinel, 1792. *Acta Geol. Pol.* 74:e4.](https://www.zotero.org/google-docs/?F5fpxR)

[McKenna A, Hanna M, Banks E, Sivachenko A, Cibulskis K, Kernytsky A, Garimella K, Altshuler D, Gabriel S, Daly M, et al. 2010. The Genome Analysis Toolkit: A MapReduce framework for analyzing next-generation DNA sequencing data. *Genome Res.* 20:1297–1303.](https://www.zotero.org/google-docs/?F5fpxR)

[Minh BQ, Schmidt HA, Chernomor O, Schrempf D, Woodhams MD, von Haeseler A, Lanfear R. 2020. IQ-TREE 2: New Models and Efficient Methods for Phylogenetic Inference in the Genomic Era. *Mol. Biol. Evol.* 37:1530–1534.](https://www.zotero.org/google-docs/?F5fpxR)

[Miranda I, Giska I, Farelo L, Pimenta J, Zimova M, Bryk J, Dalén L, Mills LS, Zub K, Melo-Ferreira J. 2021. Museomics Dissects the Genetic Basis for Adaptive Seasonal Coloration in the Least Weasel. *Mol. Biol. Evol.* 38:4388–4402.](https://www.zotero.org/google-docs/?F5fpxR)

[Mohr DW, Gaughran SJ, Paschall J, Naguib A, Pang AWC, Dudchenko O, Aiden EL, Church DM, Scott AF. 2022. A Chromosome-Length Assembly of the Hawaiian Monk Seal (Neomonachus schauinslandi): A History of “Genetic Purging” and Genomic Stability. *Genes* 13:1270.](https://www.zotero.org/google-docs/?F5fpxR)

[Monakhov V. 2020. Species-specific structure of the frontal part of the skull in the sable (Martes zibellina) and the pine marten (Martes martes). *Zool. ZHURNAL* 99:1298–1306.](https://www.zotero.org/google-docs/?F5fpxR)

[Monakhov VG. 2011. Martes zibellina (Carnivora: Mustelidae). *Mamm. Species* 43:75–86.](https://www.zotero.org/google-docs/?F5fpxR)

[Monakhov VG. 2015. IUCN Red List of Threatened Species: Martes zibellina. *IUCN Red List Threat. Species*.](https://www.zotero.org/google-docs/?F5fpxR)

[Monakhov VG. 2016. Winter diet in sable (Martes zibellina) and pine marten (Martes martes, Carnivora, Mustelidae) from the Urals. *Zool. Zhurnal* 95:1087–1095.](https://www.zotero.org/google-docs/?F5fpxR)

[Monakhov VG. 2021a. Species-Specific Structure of the Frontal Part of the Skull in the Sable (Martes zibellina) and the European Pine Marten (Martes martes). *Biol. Bull.* 48:1434–1441.](https://www.zotero.org/google-docs/?F5fpxR)

[Monakhov VG. 2021b. Tracing size distribution patterns in two Martes species across Eurasia: which populations should be protected? *Biodivers. Conserv.* 30:37–53.](https://www.zotero.org/google-docs/?F5fpxR)

[Monakhov VG. 2022. Martes martes (Carnivora: Mustelidae). *Mamm. Species* 54:seac007.](https://www.zotero.org/google-docs/?F5fpxR)

[Monakhov VG, Uspenskaya OD. 2013. On the morphological distinctness of the hybrid between the sable and pine marten. *Dokl. Biol. Sci.* 448:52–56.](https://www.zotero.org/google-docs/?F5fpxR)

[Morgulis A, Gertz EM, Schäffer AA, Agarwala R. 2006. WindowMasker: window-based masker for sequenced genomes. *Bioinforma. Oxf. Engl.* 22:134–141.](https://www.zotero.org/google-docs/?F5fpxR)

[Nagel D, Stefen C, Morlo M. 2009. The carnivoran community from the Miocene of Sandelzhausen (Germany). *Paläontol. Z.* 83:151–174.](https://www.zotero.org/google-docs/?F5fpxR)

[Natali C, Banchi E, Ciofi C, Manzo E, Bartolommei P, Cozzolino R. 2010. Characterization of 13 polymorphic microsatellite loci in the European pine marten Martes martes. *Conserv. Genet. Resour.* 2:397–399.](https://www.zotero.org/google-docs/?F5fpxR)

[Neph S, Kuehn MS, Reynolds AP, Haugen E, Thurman RE, Johnson AK, Rynes E, Maurano MT, Vierstra J, Thomas S, et al. 2012. BEDOPS: high-performance genomic feature operations. *Bioinformatics* 28:1919–1920.](https://www.zotero.org/google-docs/?F5fpxR)

[Newman C, Tsai M, Buesching CD, Holland PW, Macdonald DW, Darwin Tree of Life Consortium. 2022. The genome sequence of the European badger, Meles meles (Linnaeus, 1758). *Wellcome Open Res.* 7:239.](https://www.zotero.org/google-docs/?F5fpxR)

[Okamoto N, Tamura D, Nishimura G, Shimojima K, Yamamoto T. 2011. Submicroscopic deletion of 12q13 including *HOXC* gene cluster with skeletal anomalies and global developmental delay. *Am. J. Med. Genet. A.* 155:2997–3001.](https://www.zotero.org/google-docs/?F5fpxR)

[Partanen J, Aspi J, Kvist L, Lansink G, Henttonen H, Malinen J, Saveljev A. 2020. Escaped From Fur Farms-Now Not Only The American Mink And Blue Artic Fox, But Also The Russian Sable. *Vestn. Okhotovedeniya* 17:293–299.](https://www.zotero.org/google-docs/?F5fpxR)

[Pavlinin VN. 1963. Tobol sable. *Ural Branch Acad. Sci. USSR* 34:1–112.](https://www.zotero.org/google-docs/?F5fpxR)

[Peng X, Alföldi J, Gori K, Eisfeld AJ, Tyler SR, Tisoncik-Go J, Brawand D, Law GL, Skunca N, Hatta M, et al. 2014. The draft genome sequence of the ferret (Mustela putorius furo) facilitates study of human respiratory disease. *Nat. Biotechnol.* 32:1250–1255.](https://www.zotero.org/google-docs/?F5fpxR)

[Peng Y, Li H, Liu Z, Zhang C, Li K, Gong Y, Geng L, Su J, Guan X, Liu L, et al. 2021. Chromosome-level genome assembly of the Arctic fox (Vulpes lagopus) using PacBio sequencing and Hi-C technology. *Mol. Ecol. Resour.* 21:2093–2108.](https://www.zotero.org/google-docs/?F5fpxR)

[Pisias NG, Moore TC. 1981. The evolution of Pleistocene climate: A time series approach. *Earth Planet. Sci. Lett.* 52:450–458.](https://www.zotero.org/google-docs/?F5fpxR)

[Posłuszny M, Pilot M, Goszczyński J, Gralak B. 2007. Diet of sympatric pine marten (Martes martes) and stone marten (Martes foina) identified by genotyping of DNA from faeces. In: Annales Zoologici Fennici. JSTOR. p. 269–284.](https://www.zotero.org/google-docs/?F5fpxR)

[Pritchard JK, Stephens M, Donnelly P. 2000. Inference of Population Structure Using Multilocus Genotype Data. *Genetics* 155:945–959.](https://www.zotero.org/google-docs/?F5fpxR)

[Quinlan AR, Hall IM. 2010. BEDTools: a flexible suite of utilities for comparing genomic features. *Bioinformatics* 26:841–842.](https://www.zotero.org/google-docs/?F5fpxR)

[Ranallo-Benavidez TR, Jaron KS, Schatz MC. 2020. GenomeScope 2.0 and Smudgeplot for reference-free profiling of polyploid genomes. *Nat. Commun.* 11:1432.](https://www.zotero.org/google-docs/?F5fpxR)

[Rhie A, McCarthy SA, Fedrigo O, Damas J, Formenti G, Koren S, Uliano-Silva M, Chow W, Fungtammasan A, Kim J, et al. 2021. Towards complete and error-free genome assemblies of all vertebrate species. *Nature* 592:737–746.](https://www.zotero.org/google-docs/?F5fpxR)

[Rozhnov VV, Pishchulina SL, Meschersky IG, Simakin LV. 2013. On the ratio of phenotype and genotype of sable and pine marten in sympatry zone in the Northern Urals. *Mosc. Univ. Biol. Sci. Bull.* 68:178–181.](https://www.zotero.org/google-docs/?F5fpxR)

[Sadedin SP, Oshlack A. 2019. Bazam: a rapid method for read extraction and realignment of high-throughput sequencing data. *Genome Biol.* 20:78.](https://www.zotero.org/google-docs/?F5fpxR)

[Salesa MJ, Antón M, Siliceo G, Pesquero MD, Morales J, Alcalá L. 2013. A non-aquatic otter (Mammalia, Carnivora, Mustelidae) from the Late Miocene (Vallesian, MN 10) of La Roma 2 (Alfambra, Teruel, Spain): systematics and functional anatomy. *Zool. J. Linn. Soc.* 169:448–482.](https://www.zotero.org/google-docs/?F5fpxR)

[Samuels JX, Cavin J. 2013. The earliest known fisher (Mustelidae), a new species from the Rattlesnake Formation of Oregon. *J. Vertebr. Paleontol.* 33:448–454.](https://www.zotero.org/google-docs/?F5fpxR)

[Schibler L, Gibbs L, Benoist-Lasselin C, Decraene C, Martinovic J, Loget P, Delezoide A-L, Gonzales M, Munnich A, Jais J-P, et al. 2009. New Insight on FGFR3-Related Chondrodysplasias Molecular Physiopathology Revealed by Human Chondrocyte Gene Expression Profiling. *PLOS ONE* 4:e7633.](https://www.zotero.org/google-docs/?F5fpxR)

[Smit A, Hubley R, Green P. 2013. 2015 RepeatMasker Open-4.0.](https://www.zotero.org/google-docs/?F5fpxR)

[Stach J. 1959. On some Mustelinae from the Pliocene bone breccia of Węże. *Acta Palaeontol. Pol.* 4.](https://www.zotero.org/google-docs/?F5fpxR)

[StatSoft. 2001. Statistica: Software, Release 6.0.](https://www.zotero.org/google-docs/?F5fpxR)

[Stojak J, Jędrzejewska B. 2022. Extinction and replacement events shaped the historical biogeography of Arctic mammals in Europe: new models of species response. *Mammal Rev.* 52:507–518.](https://www.zotero.org/google-docs/?F5fpxR)

[Taylor GA, Kirk H, Coombe L, Jackman SD, Chu J, Tse K, Cheng D, Chuah E, Pandoh P, Carlsen R, et al. 2018. The Genome of the North American Brown Bear or Grizzly: Ursus arctos ssp. horribilis. *Genes* 9:598.](https://www.zotero.org/google-docs/?F5fpxR)

[Tomarovsky A, Khan R, Dudchenko O, Totikov A, Serdyukova NA, Weisz D, Vorobieva NV, Bulyonkova T, Abramov AV, Nie W, et al. 2025. Chromosome-length genome assembly of the stone marten (Martes foina, Mustelidae): A new view on one of the cornerstones in carnivore cytogenetics. *J. Hered.* 116:548–557.](https://www.zotero.org/google-docs/?F5fpxR)

[Tomarovsky AA, Khan R, Dudchenko O, Beklemisheva VR, Perelman PL, Totikov AA, Serdyukova NA, Bulyonkova TM, Pobedintseva M, Abramov AV, et al. 2025. Novel chromosome-length genome assemblies of three distinct subspecies of pine marten, sable, and yellow-throated marten (genus Martes, family Mustelidae). :2025.09.22.677678. Available from: https://www.biorxiv.org/content/10.1101/2025.09.22.677678v1](https://www.zotero.org/google-docs/?F5fpxR)

[Totikov AA, Tomarovsky AA, Perelman PL, Bulyonkova TM, Serdyukova NA, Yakupova AR, Mohr D, Foerster DW, Grau Jipoulou JH, Beklemisheva VR, et al. 2025. Comparative genomics and phylogenomics of the Mustelinae lineage (Mustelidae, Carnivora). *bioRxiv*:2025.07.01.662473.](https://www.zotero.org/google-docs/?F5fpxR)

[Twining JP, Montgomery I, Fitzpatrick V, Marks N, Scantlebury DM, Tosh DG. 2019. Seasonal, geographical, and habitat effects on the diet of a recovering predator population: the European pine marten (Martes martes) in Ireland. *Eur. J. Wildl. Res.* 65:51.](https://www.zotero.org/google-docs/?F5fpxR)

[Vincent IR, Farid A, Otieno CJ. 2003a. Variability of thirteen microsatellite markers in American mink (Mustela vison). *Can. J. Anim. Sci.* 83:597–599.](https://www.zotero.org/google-docs/?F5fpxR)

[Vincent IR, Farid A, Otieno CJ. 2003b. Variability of thirteen microsatellite markers in American mink ( *Mustela vison* ). *Can. J. Anim. Sci.* 83:597–599.](https://www.zotero.org/google-docs/?F5fpxR)

[Wang R-Y, Xiong Q, Chang S-H, Jin J-Y, Xiang R, Zeng L, Yu F. 2024. Identification of truncated variants in GLI family zinc finger 3 (GLI3) associated with polydactyly. *J. Orthop. Surg.* 19:449.](https://www.zotero.org/google-docs/?F5fpxR)

[Wang X, McKenna MC, Dashzeveg D. 2005. Amphicticeps and Amphicynodon (Arctoidea, Carnivora) from Hsanda Gol Formation, central Mongolia and phylogeny of basal arctoids with comments on zoogeography. *Am. Mus. Novit.* 2005:1–60.](https://www.zotero.org/google-docs/?F5fpxR)

[Willems T, Zielinski D, Yuan J, Gordon A, Gymrek M, Erlich Y. 2017. Genome-wide profiling of heritable and de novo STR variations. *Nat. Methods* 14:590–592.](https://www.zotero.org/google-docs/?F5fpxR)

[Wolsan M. 1990. Lower Pleistocene carnivores of Poland. *Quarterpäläntologie* 8:277–280.](https://www.zotero.org/google-docs/?F5fpxR)

[Yakupova A, Tomarovsky A, Totikov A, Beklemisheva V, Logacheva M, Perelman PL, Komissarov A, Dobrynin P, Krasheninnikova K, Tamazian G, et al. 2023. Chromosome-Length Assembly of the Baikal Seal (Pusa sibirica) Genome Reveals a Historically Large Population Prior to Isolation in Lake Baikal. *Genes* [Internet] 14:619. Available from: https://www.mdpi.com/2073-4425/14/3/619](https://www.zotero.org/google-docs/?F5fpxR)

[Yu L, Hammer RE, Li-Hawkins J, Von Bergmann K, Lutjohann D, Cohen JC, Hobbs HH. 2002. Disruption of *Abcg5* and *Abcg8* in mice reveals their crucial role in biliary cholesterol secretion. *Proc. Natl. Acad. Sci.* 99:16237–16242.](https://www.zotero.org/google-docs/?F5fpxR)

[Yu L, Li-Hawkins J, Hammer RE, Berge KE, Horton JD, Cohen JC, Hobbs HH. 2002. Overexpression of ABCG5 and ABCG8 promotes biliary cholesterol secretion and reduces fractional absorption of dietary cholesterol. *J. Clin. Invest.* 110:671–680.](https://www.zotero.org/google-docs/?F5fpxR)

[Yurgenson P. 1947. Sexual dimorphism in feeding as an ecological adaptation of a species. *Byulleten Mosk. Obshchestva Ispyt. Prir.* 52:33–35.](https://www.zotero.org/google-docs/?F5fpxR)

[Zakharov ES, Safronov VM, Pavlova AI. 2016. Winter diet of sable (Martes zibellina L.) in Yakutia. *Achiev. Sci. Technol. AIC* 30:82–87.](https://www.zotero.org/google-docs/?F5fpxR)

[Zalewski A. 2005. Geographical and Seasonal Variation in Food Habits and Prey Size of European Pine Martens.Harrison DJ, Fuller AK, Proulx G, editors. *Martens Fish. Martes Hum.-Altered Environ. Int. Perspect.*:77–98.](https://www.zotero.org/google-docs/?F5fpxR)

[Zalewski A, Jedrzejewski W, Jedrzejewski B. 2004. Mobility and home range use by pine martens ( *Martes martes* ) in a Polish primeval forest. *Écoscience* 11:113–122.](https://www.zotero.org/google-docs/?F5fpxR)

[Zhang C, Rabiee M, Sayyari E, Mirarab S. 2018. ASTRAL-III: polynomial time species tree reconstruction from partially resolved gene trees. *BMC Bioinformatics* 19:153.](https://www.zotero.org/google-docs/?F5fpxR)

[Žliobaitė I, Fortelius M, Bernor RL, Van den Hoek Ostende LW, Janis CM, Lintulaakso K, Säilä LK, Werdelin L, Casanovas-Vilar I, Croft DA. 2023. The NOW database of fossil mammals. In: Evolution of Cenozoic Land Mammal Faunas and Ecosystems: 25 Years of the NOW Database of Fossil Mammals. Springer. p. 33–42.](https://www.zotero.org/google-docs/?F5fpxR)
