# Supplementary figures and images for: Genomics of Sable (Martes zibellina) × Pine Marten (Martes martes) Hybridization
Source: Genome Biol Evol. 2026 Mar 5;18(3):evag018. doi: 10.1093/gbe/evag018 (PMC12960073; doi:10.1093/gbe/evag018)

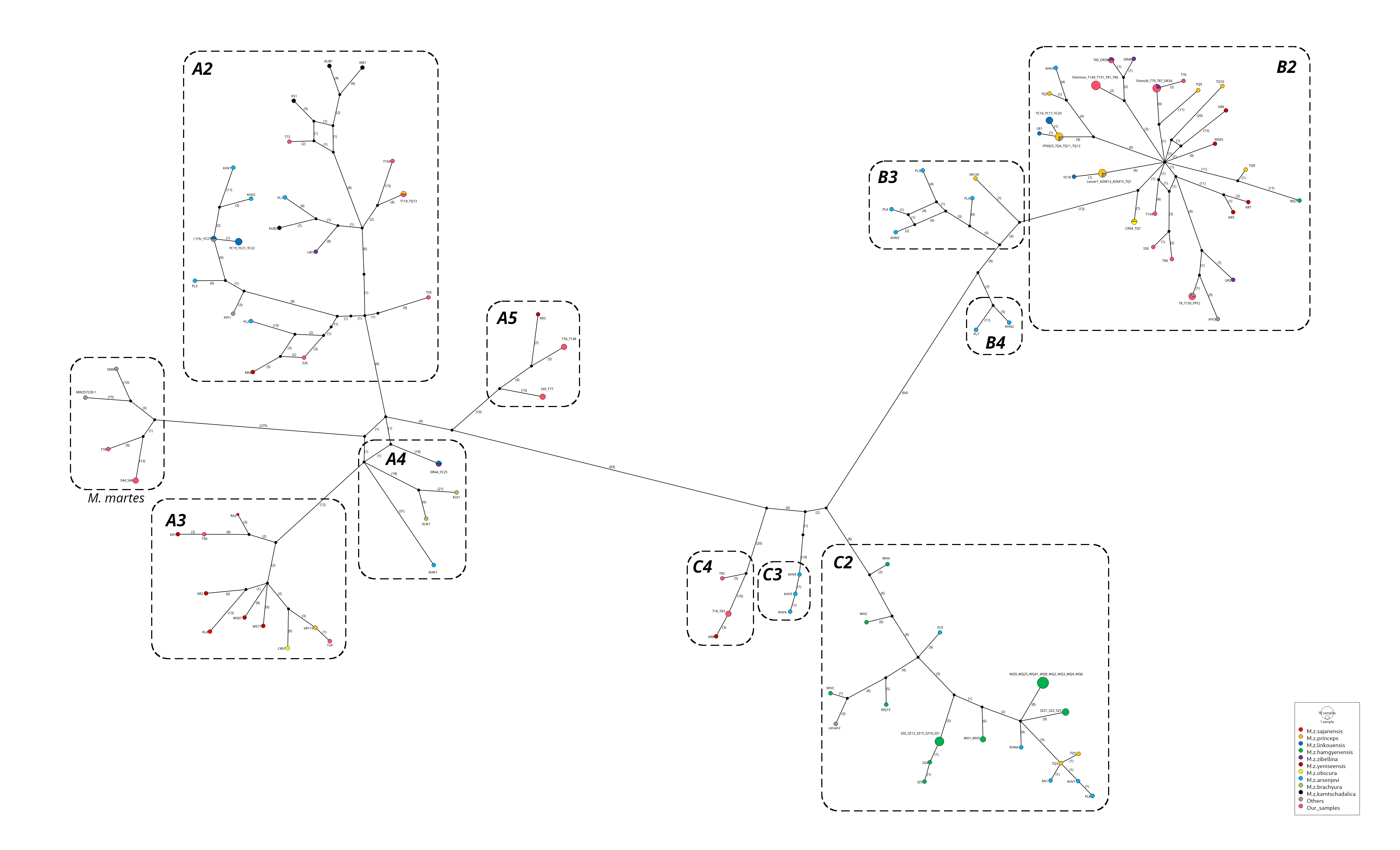

Supplement: evag018_Supplementary_Data [file evag018_supplementary_data.zip › SupplementaryFiles/SupplementaryFile_4.Mitochondrial _haplotype_network.png]
